# Carboranyl‐Curcuminoids for the Neutron Capture‐Based Treatment of Amyloid Aggregates in Alzheimer's Disease

**DOI:** 10.1002/advs.202521701

**Published:** 2026-04-16

**Authors:** Sebastiano Micocci, Stefano Parisotto, Diego Alberti, Alberto Lanfranco, Valeria Bitonto, Polyssena Renzi, Martina Salmi, Andrea Magnani, Federica Dal Bello, Katiuscia Pagano, Laura Ragona, Nicoletta Protti, Annamaria Deagostino, Simonetta Geninatti Crich

**Affiliations:** ^1^ Department of Molecular Biotechnology and Health Sciences University of Torino Torino Italy; ^2^ Department of Chemistry University of Torino Torino Italy; ^3^ Istituto Di Scienze e Tecnologie Chimiche "Giulio Natta" (SCITEC) National Council of Research (CNR) Milano Italy; ^4^ Department of Physics “A. Volta” University of Pavia Pavia Italy; ^5^ Nuclear Physics National Institute (INFN) Pavia Unit Pavia Italy

**Keywords:** Alzheimer's disease, amyloid‐β, BNCT, monocarbonyl curcumin derivatives, protein oxidation

## Abstract

Alzheimer's disease (AD) is a neurodegenerative disorder characterized by progressive cognitive decline. The aggregation of amyloid‐beta (Aβ) peptides into oligomers and fibrils is central to its pathogenesis. While oligomers represent the most neurotoxic species, larger aggregates serve as reservoirs, maintaining pathological Aβ levels. To our knowledge, this study is the first to investigate Boron Neutron Capture Therapy (BNCT) as a method to selectively destabilize Aβ aggregates. This is achieved by inducing structural modifications in the Aβ peptide, aiming to convert fibrils into innocuous species. The approach utilizes ^10^B‐enriched monocarbonyl analogs of curcumin (BMACs), a novel molecule that binds to Aβ fibrils and enables the site‐specific release of high‐linear‐energy‐transfer (LET) α particles and lithium ions upon neutron exposure. In vitro, Aβ aggregates were characterized using FESEM and Thioflavin T staining. The binding affinities of BMACs were determined through competition assays, with inhibition constants calculated using the Cheng‐Prusoff equation. Post‐irradiation analysis by ^1^H‐NMR and mass spectrometry demonstrated selective oxidation of histidine residues, a chemical modification capable of inducing fibril destabilization. This study provides proof of concept that not only offers future perspectives for Alzheimer's treatment but also enhances the understanding of radiation effects on proteins, particularly within the context of amyloidosis.

## Graphical abstract

The ^10^B‐enriched monocarbonyl analog of curcumin (BMAC) **^10^B‐9** enables site‐specific Boron Neutron Capture Therapy (BNCT) on amyloid‐β (Aβ) fibrils. Neutron irradiation induces histidine oxidation and fibril destabilization, as revealed by ^1^H‐NMR and FESEM analyses. This provides a proof of concept for Alzheimer's disease treatment and advances understanding of radiation effects on proteins.

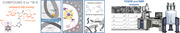

## 1 Introduction

Alzheimer's disease (AD) is a complex and heterogeneous progressive disorder of the central nervous system. As the leading cause of dementia, AD severely affects patients, families, and caregivers, and imposes major economic costs [1]. The number of people with dementia is expected to rise from 55 million in 2019 to 139 million in 2050. In addition, the financial burden of dementia on global health systems is estimated to more than double from an estimated US$1.3 trillion per year in 2019 to US$2.8 trillion by 2030 [2].

Several hypotheses have been proposed over the years to explain the etiology of AD, including genetic factors, the amyloid‐β (Aβ) cascade, and tau hyperphosphorylation. However, the pathogenesis of AD remains elusive. Among these, the amyloid cascade hypothesis suggests that the accumulation of amyloid fibrils within extracellular neuritic plaques in the cerebral cortex represents the initiating event of the disease [3], offering a biochemical perspective. According to this hypothesis, Aβ fibril deposition triggers a cascade of pathological consequences, ultimately leading to axonal disruption, synaptic loss, and neuronal death [4]. However, several investigations indicate that the most cytotoxic Aβ species are not the bigger aggregates, but soluble oligomers of varying size shapes [5], with plaques acting primarily as reservoirs. Oligomers are thought to damage cells by penetrating and disrupting membranes, forming ion channel‐like pores, and interfering with normal cellular processes [6, 7, 8, 9]. As key intermediates, these oligomers assemble into β‐sheet‐rich fibrils (hundreds of µm long, a few nm in diameter), which then aggregate into plaques (spherical assemblies of ca. 10 µm in diameter).

Most preclinical AD treatments have been designed to target amyloidogenic pathways [10, 11, 12], including small‐molecule Aβ inhibitors [13, 14, 15, 16] and anti‐Aβ monoclonal antibodies [17, 18, 19]. However, a direct correlation between plaque disaggregation and cognitive improvement has not been convincingly demonstrated, and harmful oligomeric species can form during plaque breakdown. Aducanumab, a monoclonal antibody that was approved by the FDA in 2021 (but not by the EMA) as the first treatment for AD, targeting soluble oligomers, was later withdrawn due to adverse effects [20]. Despite considerable progress in the field of understanding disease mechanisms and the recent approval of different monoclonal antibodies (Lecanemab, Donanemab) [21], there is still no cure for AD. The most common strategy for managing symptoms continues to be a combination of pharmacological and non‐pharmacological treatments, which do not address the causative agents [22].

Recently, whole‐brain radiotherapy has been suggested as a potential approach for Aβ disaggregation [23, 24, 25]. The mechanism of action of radiotherapy (RT) on systemic amyloid deposits is not well understood, but it has been proposed that RT affects the β‐sheet structure of amyloid fibrils by disrupting H‐bond interactions and depolymerizing glucoaminoglycans, highly radiation‐sensitive polysaccharides associated with amyloid fibrils. Nonetheless, its efficacy remains uncertain, and the selectivity is insufficient [24, 26, 27, 28].

**SCHEME 1:**
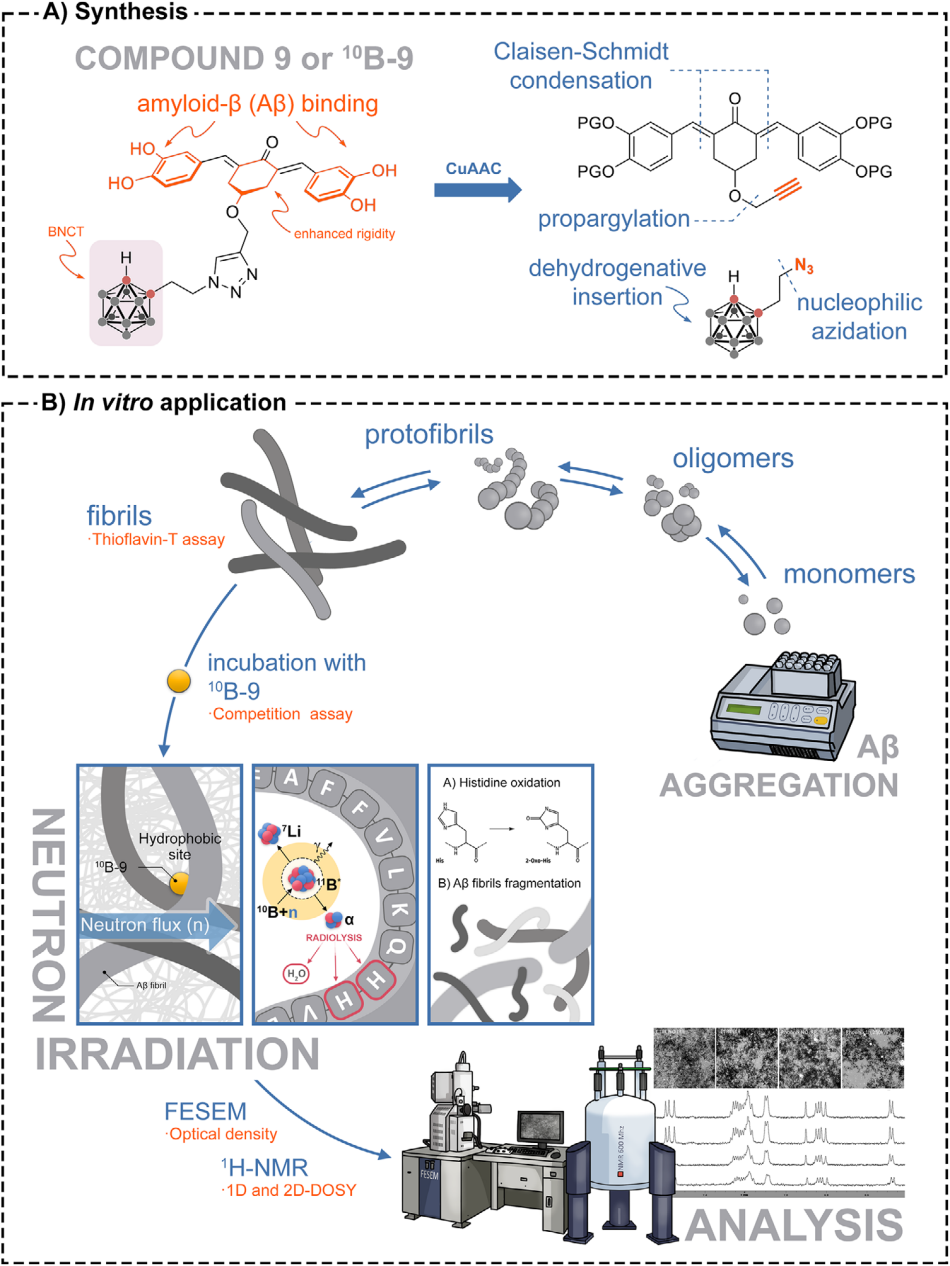
Overview of the study: (A) Design and synthesis of 2,6‐bis((*E*)‐3,4‐bis((tetrahydroxy)benzylidene)‐4‐((1‐(2‐ethylcarboranyl)‐1*H*‐1,2,3‐triazol‐4‐yl)methoxy)cyclohexan‐1‐one (9, ^10^B‐9) (B) Preparation and characterization of Amyloid‐β (Aβ) aggregates, neutron irradiation at the T.R.I.G.A. Mark II research nuclear reactor (Pavia University), and subsequent analysis of the samples by ^1^H‐NMR and FESEM microscopy.

In this context, this study introduces, for the first time to our knowledge, the use of Boron Neutron Capture Therapy (BNCT) as an alternative strategy to treat AD. BNCT enables the local release of high‐linear‐energy‐transfer (LET) α particles and lithium ions, with high selectivity, potentially inducing chemical modifications in aggregating protein fragments forming amyloid plaques rather than simply disaggregating them. BNCT is a two‐step RT modality: non‐radioactive ^10^B nuclides capture epithermal neutrons, generating high‐LET short‐range particles, which release their energy over distances of 5–9 µm, comparable to the size of a mammalian cell, confining their effect locally (Scheme 1). When boron carriers selectively deliver ^10^B to the pathological site, neutron irradiation can provide a therapeutic dose without damaging surrounding healthy tissue. The efficacy and selectivity of BNCT critically depend on the bio‐distribution of ^10^B, making it attractive not only for disseminated metastases but also for focal pathologies embedded in normal tissues, such as Alzheimer plaques.

To date, BNCT has been mostly explored for cancer [29, 30], particularly for brain tumors such as glioblastoma and glioma [31, 32, 33], with numerous preclinical studies supporting its efficacy [29, 34, 35, 36, 37, 38, 39, 40]. Although clinical applications have primarily relied on low molecular weight boron‐containing agents, polynuclear boronated derivatives have also been investigated [41, 42, 43]. Functionalized carboranes, in particular, are promising boron‐delivery vehicles due to their high boron content, chemical versatility, and in vivo stability [43, 44, 45, 46, 47, 48, 49, 50, 51, 52, 53, 54, 55]. Moreover, they can also enhance the pharmacological properties of biologically active compounds by mimicking the steric volume of a rotating phenyl ring [56]. Numerous carborane‐containing ligands targeting different receptors and proteins have been reported [43, 57, 58, 59, 60, 61, 62, 63], some for the treatment of cancer [64, 65, 66, 67, 68, 69] and others coupled with enzymatic inhibitors with improved efficacy [70, 71].

Aiming at combining the therapeutic potential of curcuminoids with NCT, we reported the synthesis of a small library of boronated monocarbonyl analogues of curcumin (BMAC), incorporating the carborane cage within the dienoyl framework [71]. Curcumin is known for its neuroprotective effects and for its affinity for Aβ fibrils [72, 73, 74, 75, 76, 77, 78, 79, 80, 81, 82, 83], but its clinical application has been limited by its poor stability and solubility in aqueous solutions [80]. Curcumin analogues with improved stability, while retaining similar bioactivity and pharmacokinetics, are therefore considered very promising scaffolds, not only as anticancer agents [84, 85, 86], but also for AD therapy [87, 88]. Among the reported BMACs, compound Chimera, showed superior activity compared to curcumin in reducing lysozyme fibril formation [71]. To overcome the limited modularity of the first generation BMAC synthesis and streamline their preparation, we designed a second family of boronated curcuminoids aimed at modulating amyloid fibril aggregation in combination with neutron irradiation (Scheme 1), which is the focus of this work.

## 2 Results and Discussion

### 2.1 Synthesis

Building on our previous work, we replaced the carborane motif in the conjugated dienone with a second catechol group to enhance amyloid fibrils affinity [71]. Additionally, to increase the rigidity of the dienone system and potentially improve the pharmacodynamic profile, we employed a curcuminoid scaffold based on a cyclic ketone [89].

**SCHEME 2:**
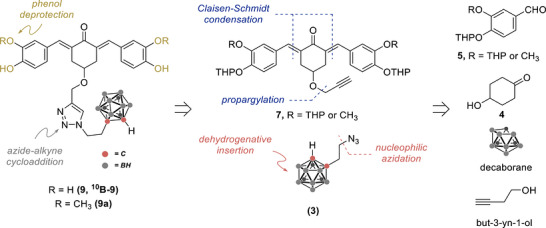
Retrosynthetic approach toward BMACs second generation.

To evaluate the role of phenol groups on the activity and toxicity of the active compounds, two agents were prepared: compounds **9** and **9a**, as described in Scheme 2, including four and two free OH groups, respectively. To introduce the carborane group, this second BMAC generation features a hydroxyl group that serves as a versatile linchpin. Leveraging the broad applicability of the copper‐catalyzed azide‐alkyne cycloaddition (CuAAC) and building on our previous work on developing Boron Neutron Capture Therapy (BNCT) theranostic agents [71], we designed a click reaction between alkyne‐functionalized curcuminoid **7** and azido‐carborane **3** (Scheme 2).

**SCHEME 3:**
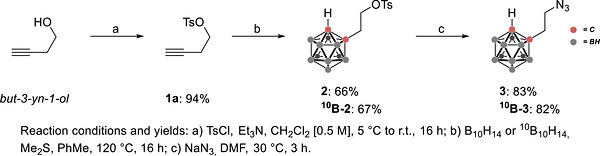
Preparation of 2‐(*C*‐*o*‐carboranyl)‐1‐azidoethane 3 and ^10^B‐3.

The azido derivative, 2‐(*C*‐*o*‐carboranyl)‐1‐azidoethane **3**, was synthesized from commercially available but‐3‐yn‐1‐ol (Scheme 3). This synthesis followed a dehydrogenative insertion reaction of tosylate **1** and decaborane in the presence of Me_2_S as the Lewis base. The corresponding *closo*‐carborane (compound **2**) was isolated in 66% yield, consistent with similar reactions reported in the literature [43, 90]. Subsequent nucleophilic displacement of the sulfonyl group with sodium azide in DMF at 30°C led to the complete conversion of compound **2** to the desired azide **3** in 83% yield within three hours. The same synthetic protocol was employed with ^10^B‐enriched decaborane, resulting in the preparation of ^10^B‐enriched 2‐(*C*‐*o*‐carboranyl)‐1‐azidoethane (**^10^B‐3**), which was obtained in a combined yield of 55% from tosylate **1**, like the non‐enriched analogue (55%).

**SCHEME 4:**
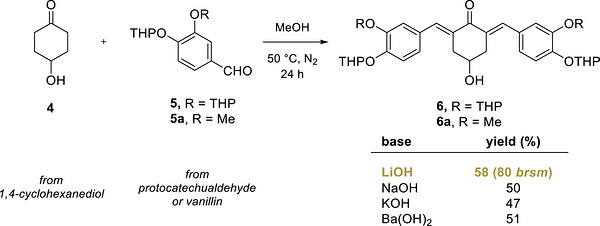
Claisen‐Schmidt condensation between cyclohexanone 4 and aldehyde 5/5a.

For the curcuminoid core structure, 4‐hydroxycyclohexan‐1‐one **4** was synthesized by selective monoxidation of 1,4‐cyclohexanediol using a combination of sodium bromate and cerium ammonium nitrate as oxidizing agents [91]. A subsequent base‐catalyzed Claisen‐Schmidt condensation with protected protocatechualdehyde **5** [71], or vanillin **5a** afforded the corresponding dienone **6** and **6a** on a gram scale. After screening various bases, lithium hydroxide (LiOH) was identified as the most efficient base, yielding 2,6‐bis((*E*)‐3,4‐ditetrahydropyranylbenzylidene)‐4‐hydroxycyclohexan‐1‐one **6** in 58% (80% yield based on the recovered, unreacted aldehyde), Scheme 4. The optimized protocol was then applied to tetrahydropyranyl vanillin **5a,** affording 2,6‐bis((*E*)‐3‐methoxy‐4‐tetrahydropyranylbenzylidene)‐4‐hydroxycyclohexan‐1‐one **6a** with an overall yield of 47%.

**SCHEME 5:**
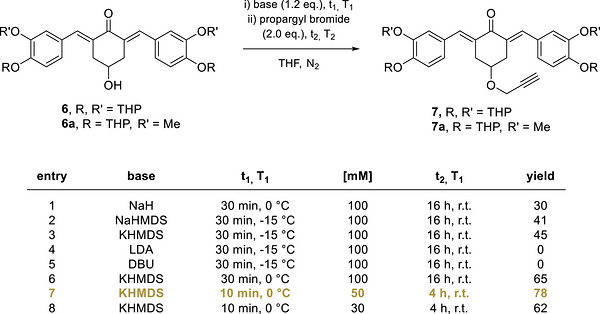
Propargylation of cyclohexanol 6/6a.

To synthesize the target alkyne **7**, suitable for ligation to azido‐carborane **3**, a propargylation reaction using propargyl bromide was employed. Optimization of this step was necessary, as the standard procedure using NaH gave a poor yield of only 30%. The results of the optimization are summarized in Scheme 5. Various bases were screened, and hexamethyldisilazides (HMDS) showed promise. Specifically, NaHMDS yielded 41% of the propargyl ether **7**. In order to generate a more reactive potassium alkoxide intermediate, KHMDS was tested resulting in a slight improvement in yield to 45%. Further optimization involved deprotonating alcohol **6** at 0°C instead of −15°C, which raised the yield to 65%. Finally, reducing the reaction concentration from 100 to 50 mm improved the yield to 78% for 2,6‐bis((*E*)‐3,4‐ditetrahydropyranylbenzylidene)‐4‐(3‐propargyloxy)cyclohexan‐1‐one 7. Again, the optimized conditions were extended to **6a** to obtain 2,6‐bis((*E*)‐3‐methoxy‐4‐tetrahydropyranylbenzylidene)‐4‐(3‐propargyloxy)cyclohexan‐1‐one **7a** in 47% yield.

**SCHEME 6:**
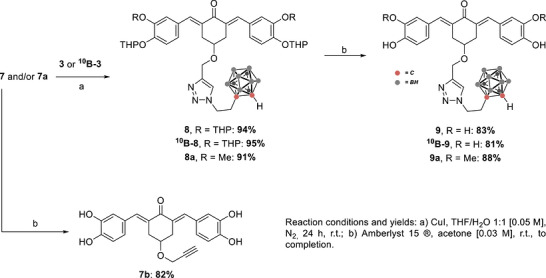
CuAAC followed by acidic treatment for 9, ^10^B‐9, 9a, and 7b syntheses.

The two fragments were successfully joined via copper‐catalyzed azide‐alkyne cycloaddition (CuAAC) in the presence of stoichiometric cuprous iodide in aqueous THF. The triazole formation proceeded with excellent efficiency, yielding both THP protected 2,6‐bis((*E*)‐3,4‐bis((tetrahydro‐2*H*‐pyran‐2‐yl)oxy)benzylidene)‐4‐((1‐(2‐ethylcarboranyl)‐1*H*‐1,2,3‐triazol‐4‐yl)methoxy)cyclohexan‐1‐one (**8**) and 2,6‐bis((*E*)‐3‐methoxy‐4‐(tetrahydro‐2*H*‐pyran‐2‐yl)oxy)benzylidene)‐4‐((1‐(2‐ethylcarboranyl)‐1*H*‐1,2,3‐triazol‐4‐yl)methoxy)cyclohexan‐1‐one (**8a**) were obtained in high yields of 94% and 91%, respectively. In the case of **9**, the ^10^B‐enriched derivative (**^10^B‐8**), necessary for an effective neutron irradiation, was successfully prepared in 95% yield. Lastly, acid‐promoted hydrolysis of the acetal protecting groups liberated the catechol residues, affording the natural abundant and ^10^B enriched compounds 2,6‐bis((*E*)‐3,4‐bis((tetrahydroxy)benzylidene)‐4‐((1‐(2‐ethylcarboranyl)‐1*H*‐1,2,3‐triazol‐4‐yl)methoxy)cyclohexan‐1‐one **(9** and **^10^B‐9)** in good yields of 83% and 81%, respectively, and 2,6‐bis((*E*)‐3‐methoxy‐4‐(tetrahydroxy)benzylidene)‐4‐((1‐(2‐ethylcarboranyl)‐1*H*‐1,2,3‐triazol‐4‐yl)methoxy)cyclohexan‐1‐one **(9a)** in 88% yield (Scheme 6). The same protocol has been followed for the synthesis of 2,6‐bis((*E*)‐3,4‐dihydroxybenzylidene)‐4‐(prop‐2‐yn‐1‐yloxy)cyclohexan‐1‐one **(7b)**, in comparable yields.

### 2.2 Interaction With Amyloid‐β (Aβ) Aggregates

The rate at which Aβ aggregates form is well‐known to be influenced by various factors, such as the composition of the buffer, the presence of small aggregates, the methods for dissolution, and the conditions during incubation (concentration, temperature, and mixing). In fact, many different preparation protocols can be found in the literature. Among them, we followed the protocol described by De Simone et al. [92] and Bartolini et al. [93] where HFIP‐pretreated Aβ samples, freshly resolubilized in an CH_3_CN/Na_2_CO_3_/NaOH aqueous mixture, showed a good level of stability and a relatively low percentage of oligomeric/fibrillar conformation. Aβ (1‐42) peptide was selected due to its high aggregation propensity and greater toxicity compared to other Aβ fragments [94]. The aggregation kinetics of Aβ were monitored by Thioflavin‐T (ThT) fluorescence enhancement, which is a commonly used stain to monitor in vitro amyloid fibril formation [95].

Previous studies [96, 97] indicate that stirring Aβ samples may speed up the formation of fibrils by enhancing the nucleation and reducing the lag time. Accordingly, we observed a significant increase in ThT fluorescence enhancement (Figure S1A) at the highest stirring rate (600 rpm) compared to the lower (400 rpm). The different nucleation and morphology obtained under the two conditions were confirmed by FESEM (Figure S1B). It is worth noting that fibrils prepared with the commercial Aβ peptide exhibit considerable intrinsic variability, largely due to partial oligomerization of the peptide, which can occur even during storage. This variability affects both the fibril aggregation and maturation rates, as demonstrated by the high standard deviations observed in ThT fluorescence of Figure S1A [98].

### 2.3 Thioflavin‐T Fluorescence Binding and Competition Assay

**FIGURE 1:**
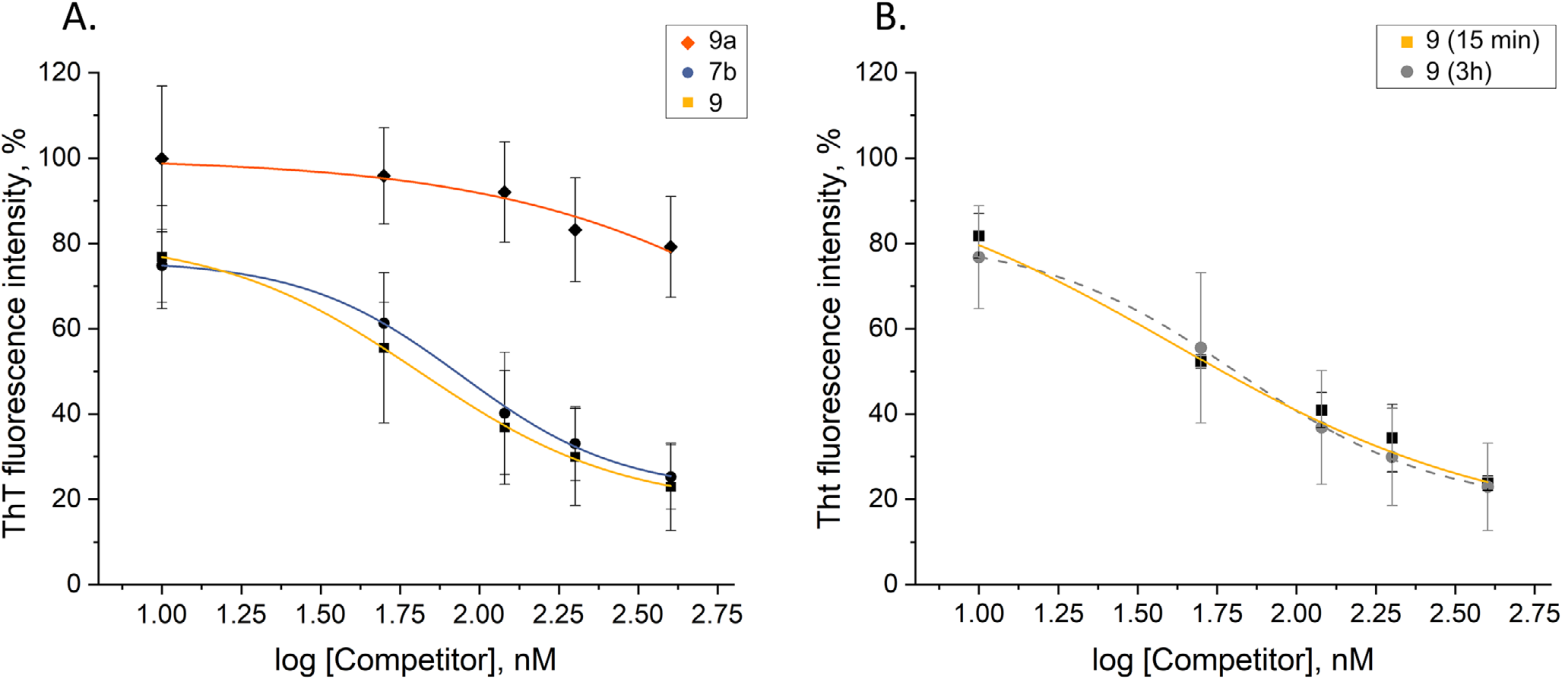
(A) Plot of ThT fluorescence (100 nm, *λ*_ex._ 450 nm, *λ*_em._ 476 nm) in the presence of Aβ (200 nm), as a function of the concentration logarithm of **7b**, **9**, and **9a** after 3 h pre‐incubation. (B) Comparison of ThT fluorescence (measured under the same conditions) for **9** after pre‐incubation for 3 h or 15 min, respectively. Data are presented as mean ± SD (A: *n* = 4; B: *n* = 2 independent experiments). Dose‐response curves were fitted using the DoseResp function in OriginLab to determine IC_50_ values.

The binding affinity of **9** and its derivatives to Aβ fibrils was assessed using a fluorescent competition assay exploiting ThT as a reporter. In fact, a direct titration was not possible as the compounds themselves do not produce any detectable fluorescence when bound to Aβ fibrils. Instead, the thermodynamic affinity constants were indirectly determined by measuring the fluorescence emission spectra of ThT in the presence of increasing concentrations of Aβ fibrils (Figure 1A). Finally, K_i_ values were calculated from the Cheng‐Prusoff equation [99], with the dissociation constants of the reporter ligands in the assay determined as K_d (ThT)_ = 7.2 µm and K_d (curcumin)_ = 4.4 µm. This competition assay was feasible because the absorption spectra of **9** and its derivatives do not overlap with the ThT fluorescence emission, which was monitored at 476 nm (Figure S2D–F). Moreover, upon excitation at 450 nm, the compounds showed negligible fluorescence in the absence or presence of amyloid fibrils (Figure S2A–C). The ThT thermodynamic dissociation constant (K_d_) to preformed Aβ fibrils was determined by plotting the fluorescence emission at 476 nm as a function of Aβ concentration. (Figure S3) The fitting of the obtained behavior with Equation 1 [100] yielded a K_d_ of 7.2 ± 1.8 µm, assuming a 1:1 interaction with Aβ aggregates. This value is within the range of K_d_ (0.5–20 µm) reported in the literature [101], as determined by fluorescence measurements.

**FIGURE 2:**
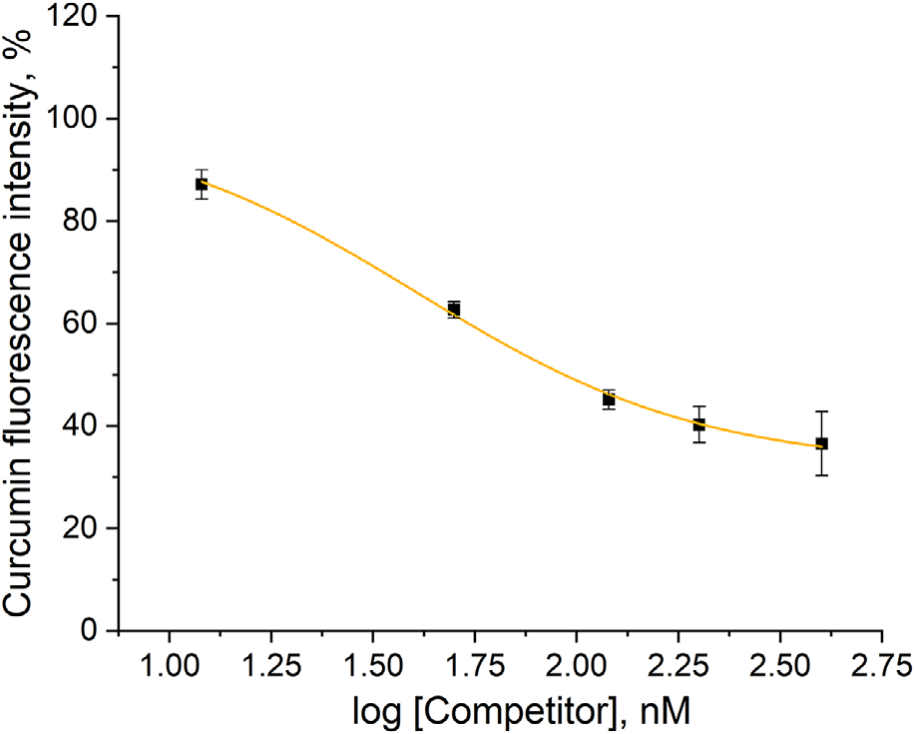
Plot of curcumin fluorescence emission (100 nm, λ_ex._ 450 nm, λ_em._ 495 nm) in the presence of Aβ (200 nm), as a function of logarithm of **9** concentration. Experimental points were fitted using the DoseResp fitting function in OriginLab to determine IC_50_ values_._ Data are presented as mean ± SD (*n* = 2 independent experiments). Dose‐response curves were fitted using the DoseResp function in OriginLab to determine IC_50_ values.

Table 1 shows that **9** exhibits a strong binding affinity for Aβ fibrils, with a K_i_ value of 50 nm, as determined through competition experiments with ThT. In all experiments, fibrils were incubated with the compounds for 3 h and subsequently with ThT for 15 min before fluorescence measurements. To rule out the possibility that the competitive effect resulted from partial fibril dissociation induced by the compounds, the titration with compound **9** was repeated using a shorter incubation time (15 min), yielding comparable results (K_i_ = 39 ± 4 nm, Figure 1B). This finding was further validated by repeating the competition assay, using curcumin as the fluorescent probe instead of ThT (Figure 2). In fact, curcumin is also known to exhibit a marked increase in its fluorescence emission (measured at 490 nm) upon binding to Aβ fibrils, along with a red shift of its absorption maximum to 450 nm. Thus, using for curcumin a K_d_ = 3.85 µm previously determined by our group employing Aβ fibrils prepared with the same protocol [100], a K_i_ of 41 nm was obtained, in agreement with the value found using ThT (Table 2). This result confirms that ThT and curcumin share a common binding site on fibrils surface, in agreement with literature data [102]. ThT was proposed to bind to primarily hydrophobic grooves (involving residues V18, F20, E22), with its long axis oriented almost parallel to the fibril axis. Additionally, Hotsumi et al. [103] demonstrated that a C5‐monoketone variant of curcumin, which features hydroxyl groups at the 2, 3, 2′, and 5′ positions, exhibits a comparable binding mechanism.

**TABLE 1:** Inhibition constants of the tested compounds relative to the ThT competition assay, assuming 1:1 interaction with the substrate.

Compound	Structure	IC_50_ ± SD [nM]	K_i_ ± SD [nM]
**9**	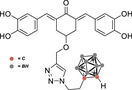	51 ± 28	50 ± 28
**7b**	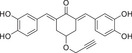	76 ± 17	75 ± 17
**9a**	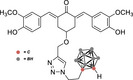	> 600	n.d.

**TABLE 2:** Inhibition constants of the tested compounds relative to the curcumin competition assay, assuming 1:1 interaction with the substrate.

Compound	C_50_ ± SD [nM]	K_i_ ± SD [nM]
**9**	42 ± 15	41 ± 15

Based on the results shown in Table 1, we can conclude that: (1) the affinity of **9** for the fibrils is primarily attributed to the curcumin‐derived portion of the molecule; (2) the presence of the carborane moiety enhances the molecule affinity; and (3) the catechol group is essential to obtain this high affinity.

### 2.4 Neutron Irradiation on Amyloid‐β Aggregates

The first assessment of the effect of neutron irradiation was carried out on mature Aβ fibrils (see Experimental section 4.4.1), exploiting the thermal neutron uncollimated and locally isotropic field available at the modified thermal column of the research nuclear reactor T.R.I.G.A. Mark II of Pavia University. The irradiation position is characterized by an unperturbed, in‐air total neutron flux Φ = 1.2×10^10^ cm^−2^s^−1^. Samples have been irradiated for 15 or 60 min, at two different reactor power (30 or 250 kW) leading to total neutron fluences Ψ of 1.3×10^12^ cm^−2^ (Ψ_1_), 1.1×10^13^ cm^−2^ (Ψ_2_), and 4.3×10^13^ cm^−2^ (Ψ_3_), respectively [104]. After irradiation, Aβ fibrils integrity in the irradiated controls (CI) was assessed by ThT fluorescence. No significant changes were observed compared to the non‐irradiated controls (CNI) (Figure S4), suggesting that neutron interactions in the absence of ^10^B do not provide sufficient energy to trigger disaggregation. Unfortunately, competition between **^10^B‐9** and ThT for the same binding sites prevents the use of this dye to monitor Aβ fibril disaggregation induced by neutron irradiation in the presence of **^10^B‐9**.

### 2.5 FESEM Analysis of ^10^B‐9 Treated Aβ Samples

Disaggregation analysis of all irradiated and non‐irradiated samples, including the Aβ controls, was initially assessed by field emission scanning electron microscopy (FESEM). To compare non‐irradiated controls (CNI: Aβ alone; 9NI: Aβ with **^10^B‐9**) with irradiated samples in the absence (CI: Aβ alone) or presence of **^10^B‐9** (9I: Aβ with **^10^B‐9**), a semiquantitative evaluation of fibril content was performed by analyzing 15 randomly selected images per experimental condition. (Figures S5–S7).

**FIGURE 3:**
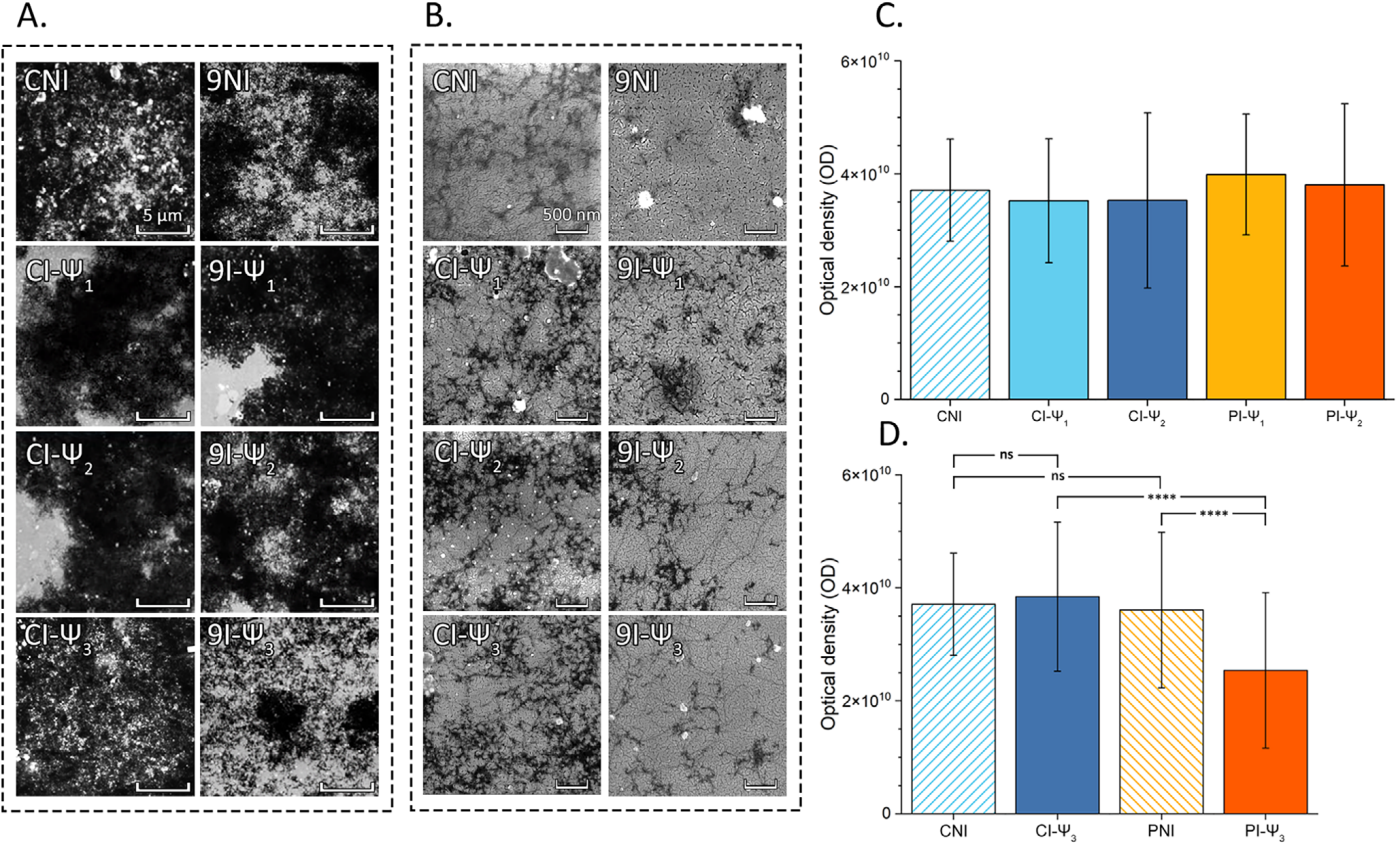
(A) Representative FESEM images of non‐irradiated (CNI) and irradiated Aβ (CI) samples, as well as non‐irradiated (9NI) and irradiated (9I) Aβ samples treated with **^10^B‐9** at a neutron fluence of 1.3 × 10^12^ cm^−2^ (Ψ_1_), 1.1 × 10^13^ cm^−2^ (Ψ_2_), and 4.3 × 10^13^ cm^−2^ (Ψ_3_). Scalebar: 5 µm; (B) Representative FESEM images of the same sample groups acquired at higher magnification. Scale bar: 500 nm; (C) Average optical densities measured from different irradiation sessions at neutron fluences Ψ_1_, Ψ_2_, *n* = 5 each. Data are presented as mean ± SD. (D) Average optical densities measured across different Ψ_3_ irradiation sessions, *n* = 7; Statistical comparisons between two independent groups were performed using a two‐tailed unpaired Student's *t*‐test (see Table S1). ns, *p* ≥ 0.05; *p < 0.05; **p < 0.01; ***p < 0.001; ****p < 0.0001.

**FIGURE 4:**
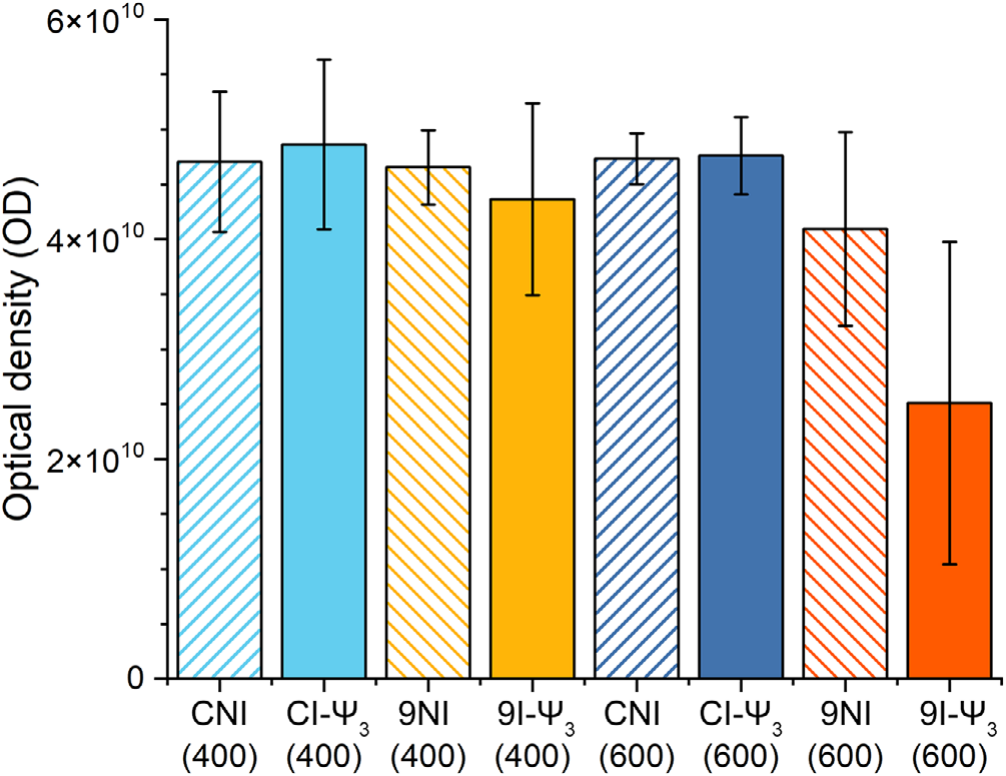
Average optical densities from different Ψ_3_ irradiation sessions. Aggregates were prepared at 400 or 600 rpm stirring over 4 days incubation, followed by 1 day incubation with **^10^B‐9**. *n* = 2. Data are presented as mean ± SD.

Figure 3A,B shows representative FESEM images (at 15 000× and 1 00 000× magnification, respectively) of the residual fibrils present in the different samples irradiated at three different neutron fluences (Ψ_1_, Ψ_2_, Ψ_3_). To further explore the fibrils' morphology at a higher level of detail, additional high‐resolution FESEM images (200,000× magnification) of a Ψ_3_ irradiation session were acquired using chromium‐coated glass coverslips (Figure S8). In Figure 3C,D, the comparison of the semiquantitative analysis of the different samples evidenced a significant reduction of the Aβ aggregates (‐34%) only in samples irradiated at Ψ_3_ in the presence of **^10^B‐9** whereas the observed differences are not significant incubating Aβ with **^10^B‐9** alone or exposed to a lower neutron flux. Despite, the relatively high SD of the data due to the well‐known high intrinsic variability related to a partial oligomerization of the commercial Aβ (1‐42) peptides occurring also under storage (−20°C), able to change the aggregation and maturation rate of fibrils, a significant disaggregation was observed only in samples treated with neutrons in the presence of boron. Interestingly, by repeating the same experiment using non‐mature proto‐fibrils (400 rpm condition), showing a low ThT binding (and presumably also a low **^10^B‐9** affinity), no disaggregating effect was observed (Figure 4). This confirms the hypothesis that, to be effective, the boronated compounds must be bound to the protein aggregates and not simply be dissolved in the same solution.

### 2.6 ^1^H‐Nuclear Magnetic Resonance (NMR) Analysis of Amyloid‐β Aggregates

The occurrence of chemical modifications in Aβ amino acid residues induced by neutron irradiation (with or without **^10^B‐9**) and the effect on equilibria between monomer and aggregated species was evaluated by ^1^H‐NMR spectroscopy. The high LET ionizing radiations generated by the boron neutron capture reaction can exert a ionization effect and may release their energy via two different pathways when interacting with biological matter: 1) a direct effect, consisting of direct energy transfer to biomolecules, leading to a loss of one electron and, consequently, to the formation of radical cations; 2) an indirect effect, in which energy is transferred to water, causing its radiolysis and the generation of reactive oxygen species (ROS), among which the hydroxyl radical (HO•) is the most reactive. While the consequences of α particles interactions are well known for DNA, this study represents, to our knowledge, the first attempt to investigate their impact on Aβ fibrils.

NMR spectroscopy is a valuable tool to study the structure and assembly of Aβ fibrils and oligomers derived from different protein precursors. Solution‐state NMR is particularly suitable for analysing small, water‐soluble species such as monomers and small oligomers, which exhibit rapid tumbling rates characteristic of early aggregation stages [105]. To enable NMR analysis, the Aβ fibrils, collected after neutron irradiation, with the neutron fluences already reported in the previous section, were solubilized in 60 mM aqueous NaOH, because a highly basic pH promotes the disassembling of bigger aggregates into monomeric forms. As reported by Pilkington et al., [106], this concentration (60 mm) effectively solubilizes the maximum amount of monomeric species (Figures S9B and S10), lower concentrations resulted in reduced solubilization efficiency (Figures S9A and S10). Increasing the concentration to 100 mm does not further enhance the ^1^H‐NMR signals of dissolved monomers; instead, it leads to peak broadening, likely due to the increased viscosity of the solution (Figures S9C and S10). On each sample, a combination of 1D, ^1^H quantitative NMR spectra together with diffusion edited spectra (2D‐DOSY) was recorded to detect the effect of the irradiation on the chemical integrity of the Aβ peptide and on the equilibria between aggregated species.

**FIGURE 5:**
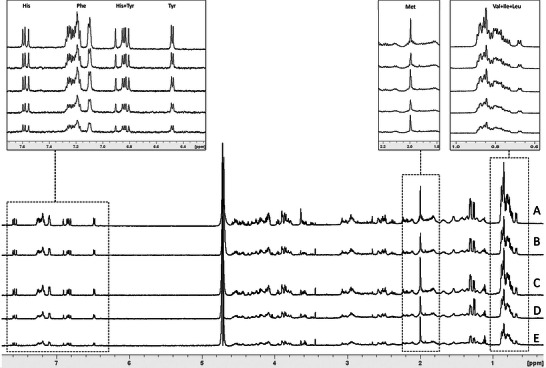
Representative ^1^H‐NMR spectra (1D‐NOESY, 600 MHz) of Aβ fibril samples after Ψ_3_ irradiation session. (A) non‐irradiated Aβ monomers; (B) non‐irradiated Aβ control (CNI); (C) neutron‐irradiated Aβ control at Ψ_3_ (CI‐Ψ_3_); (D) non‐irradiated Aβ with **^10^B‐9** (9NI); (E) neutron‐irradiated Aβ with **^10^B‐9** (9I‐Ψ_3_). The overall spectral pattern is conserved across conditions, while a marked decrease in signal intensity is observed in **^10^B‐9**‐treated samples.

Figure 5 reports ^1^H‐NMR spectra of Aβ fibrils, comparing non‐irradiated control samples (CNI, 9NI) with those undergone to neutron irradiation at the highest fluence (CI‐Ψ_3_, 9I‐Ψ_3_).

**FIGURE 6:**
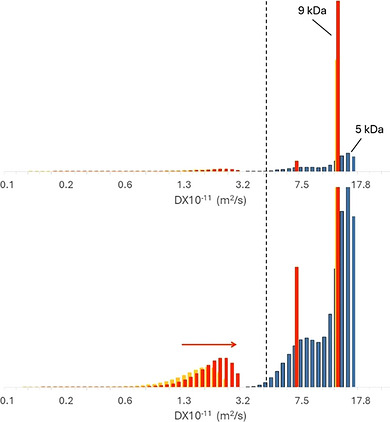
Overlay of the diffusivity distribution derived from the Inverse Laplace Transform (ILT) analysis of the non‐exponential decays measured from 2D‐DOSY recorded for not irradiated Aβ control (CNI, blue), not irradiated Aβ with **^10^B‐9** (9NI, yellow), irradiated Ab with **^10^B‐9** at Ψ_3_ (9I, orange, color code as in Figure 7). Samples were acquired at 600 MHz, 25°C. The “apparent MW” (see Experimental section 4.4.5) of the most populated visible species is labelled. ILT analysis also reveals the presence in the sample of NMR invisible large assemblies (D lower than < 5.5×10^−11^ m^2^ s^−1^, dashed line) in rapid exchange with the NMR‐visible low molecular species, characterized by diffusion values that cannot however, be quantitatively translated into reliable “apparent MWs”. An arrow indicates the shift of this distribution toward higher D values (lower apparent MWs) upon irradiation.

1D ^1^H data analysis indicates that the overall spectral pattern remained unchanged across all conditions, suggesting that **^10^B‐9** does not interact with the NMR visible Aβ species. However, the remarkable decrease in Aβ signal intensity, together with the absence of **^10^B‐9** signals in the spectra of the Aβ samples treated with the boronated curcuminoid, supported its high‐affinity binding to invisible large Aβ assemblies, in agreement with the nanomolar K_d_ measured from fluorescence data. This hypothesis is validated by Inverse Laplace Transform (ILT) analysis of NMR diffusion spectra (2D‐DOSY) recorded on all the samples, giving hints on the distribution of species in equilibrium with monomers in solution [107]. In the Aβ peptide samples, the predominant species is monomer, although oligomeric forms are also present (Figure 6, blue), even under basic pH conditions. Upon addition of the curcuminoid compound, the emergence of higher molecular weight species in exchange with visible monomers and/or small oligomers is observed (Figure 6, red), suggesting that **^10^B‐9** can redirect Aβ peptides into larger assemblies and justifying the overall decrease of the Aβ peptide signal intensity. Interestingly, diffusion data further indicate that when **^10^B‐9** is bound to large assemblies, neutron irradiation perturbs them, supporting a disaggregating effect (Figure 6, orange).

**FIGURE 7:**
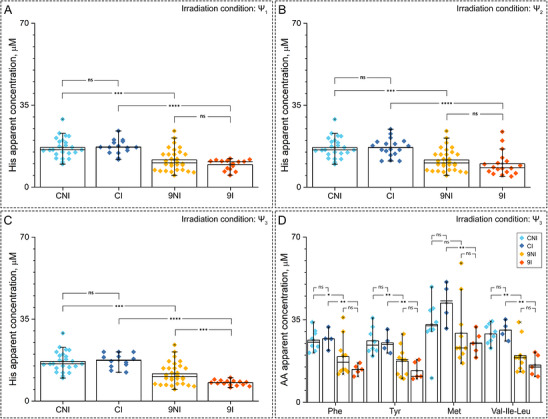
^1^H‐qNMR analysis of selected amino acid residues. Quantification was performed using the ERETIC method with an external standard. Only histidine signals at 6.4 ppm were considered. (A) Histidine apparent concentration in samples irradiated with a neutron fluence of 1.3 × 10^12^ cm^−2^ (Ψ_1_) *n* = 8 (CNI), 10 (9NI), 5 (CI), 5 (9I). (B) Histidine apparent concentration in samples irradiated with a neutron fluence of 1.1 × 10^13^ cm^−2^ (Ψ_2_) *n* = 8 (CNI), 10 (9NI), 6 (CI), 6 (9I). (C) Histidine apparent concentration in samples irradiated with neutron fluence of 4.3×10^13^ cm^−2^ (Ψ_3_), *n* = 8 (CNI), 10 (9NI), 4 (CI), 5 (9I). (D) Apparent concentration of other oxidizable amino acids in samples irradiated with a neutron fluence of 4.3×10^13^ cm^−2^ (Ψ_3_), n = 8 (CNI), 10 (9NI), 4 (CI), 5 (9I). Data are shown as mean ± SD. Statistical comparisons between two independent groups were performed using a two‐tailed unpaired Student's t‐test (see Table S2). ns, *P* ≥ 0.05; *p < 0.05; **p < 0.01; ***p < 0.001; ****p < 0.0001.

The effect of neutron irradiation on chemical modifications of Aβ residues was investigated by quantitative analysis of ^1^H 1D NMR spectra. The NMR signals of selected amino acid residues of Aβ (histidine, phenylalanine, tyrosine, methionine, valine, isoleucine, and leucine) were integrated using ERETIC (see Experimental section, Section 4.4.5) with an external standard (Figure 7).

This analysis allowed to conclude that:

1. In the not‐irradiated Aβ control (CNI), the apparent concentration of all analyzed amino acids remained unchanged upon neutron exposure. This finding is consistent with the ThT assay and FESEM analyses, both confirming a negligible effect of neutron irradiation on Aβ aggregation in absence of the boronated curcuminoid.
2. All samples treated with **^10^B‐9** exhibited a statistically significant reduction in the apparent concentration of the analyzed aminoacids compared to the respective control samples (Table S2), with no observed selectivity. This decrease may result from the interaction of **^10^B‐9** with Aβ fibrils, leading to the stabilization of high molecular weight aggregates that remain intact even under basic conditions, thereby reducing the NMR‐visible fraction. After irradiation a further, statistically not significant, decrease is observed in the presence of **^10^B‐9** (Table S2). Metionine CH_3_ ε peak is too encumbered to obtain a clear evaluation of the apparent concentration.
3. Histidine showed a similar trend, but it was the only residue that show a significant decrease in the apparent concentration after the highest neutron fluence (Table S2), suggesting a selective oxidation of this amino acid. The corresponding oxidation product (oxo‐histidine) was likely not detected due to its poor stability under basic pH conditions [108]. Notably, since histidine plays a crucial role in fibril formation and stabilization, its oxidation may initiate Aβ fibril disaggregation [109, 110, 111, 112, 113, 114]. Interestingly, the histidine oxidation was already observed with lower neutron fluence (Ψ_1_ and Ψ_2_) but became significant at the highest (Ψ_3_). The difficulty of observing histidine oxidation in the absence of metals (particularly Cu) [115, 116], further supports the conclusion that the high LET ionizing radiations generated from the reaction between neutrons and **^10^B‐9** possess sufficient energy to directly ionize this residue.

To exclude the formation of stable soluble oligomers upon irradiation, the NMR analysis of Aβ fibrils subjected to neutron irradiation (at the highest fluence Ψ3), was carried out on samples dissolved in phosphate buffer (pH = 7.4). ^1^H‐NMR spectra (Figure S11) did not reveal the stabilization of soluble, stable off‐pathway oligomers in the range 4–100 kDa. However, the formation of transient oligomeric species cannot be excluded by a spectroscopic approach.

**FIGURE 8:**
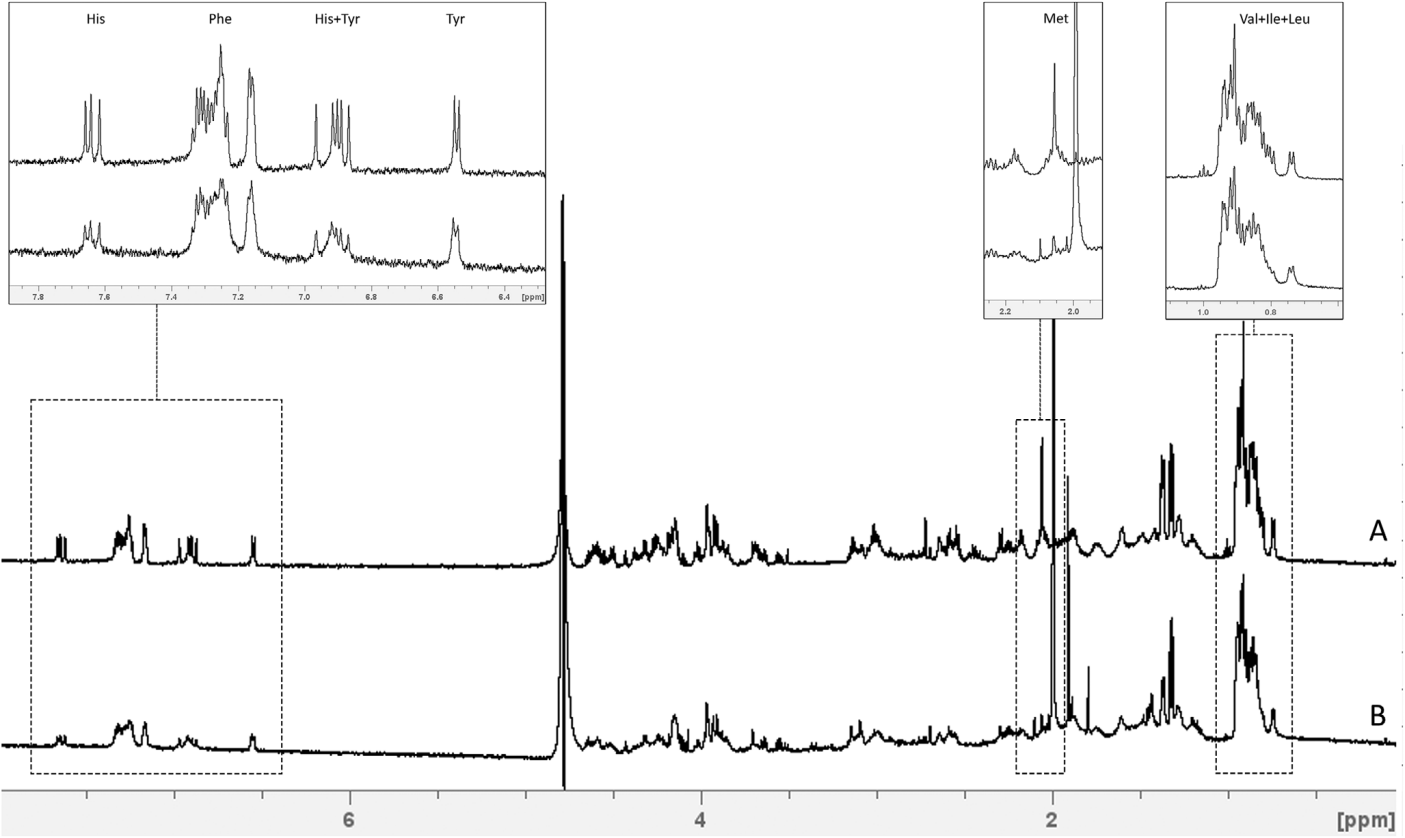
Representative ^1^H‐NMR spectra (1D‐NOESY, 600 MHz, 25°C, D_2_O, 256 scans, d1 = 11 s) of Aβ fibrils before and after oxidation with 0.5% H_2_O_2_ for 2 h. (A) non‐oxidized control (Aβ CTRL); (B) oxidized control by 0.5% H_2_O_2_ (Aβ OX). It's observed a marked decrease in the Met signal (*δ* = 2.06), together with the appearance of a peak at *δ* = 1.93 corresponding to the oxidation of acetonitrile to acetamide.

To investigate more deeply the oxidation mechanism and distinguish between direct and indirect effects of high LET ionizing radiations, a parallel ^1^H‐NMR study was carried out after the addition of 0.5% H_2_O_2_ to the Aβ fibrils suspension, to mimic oxidation resulting from the indirect effect of α particle exposure. As shown in Figure 8, 9 the hydroxyl radicals generated by H_2_O_2_ oxidised both methionine and histidine, in agreement with literature reports [106].

**FIGURE 9:**
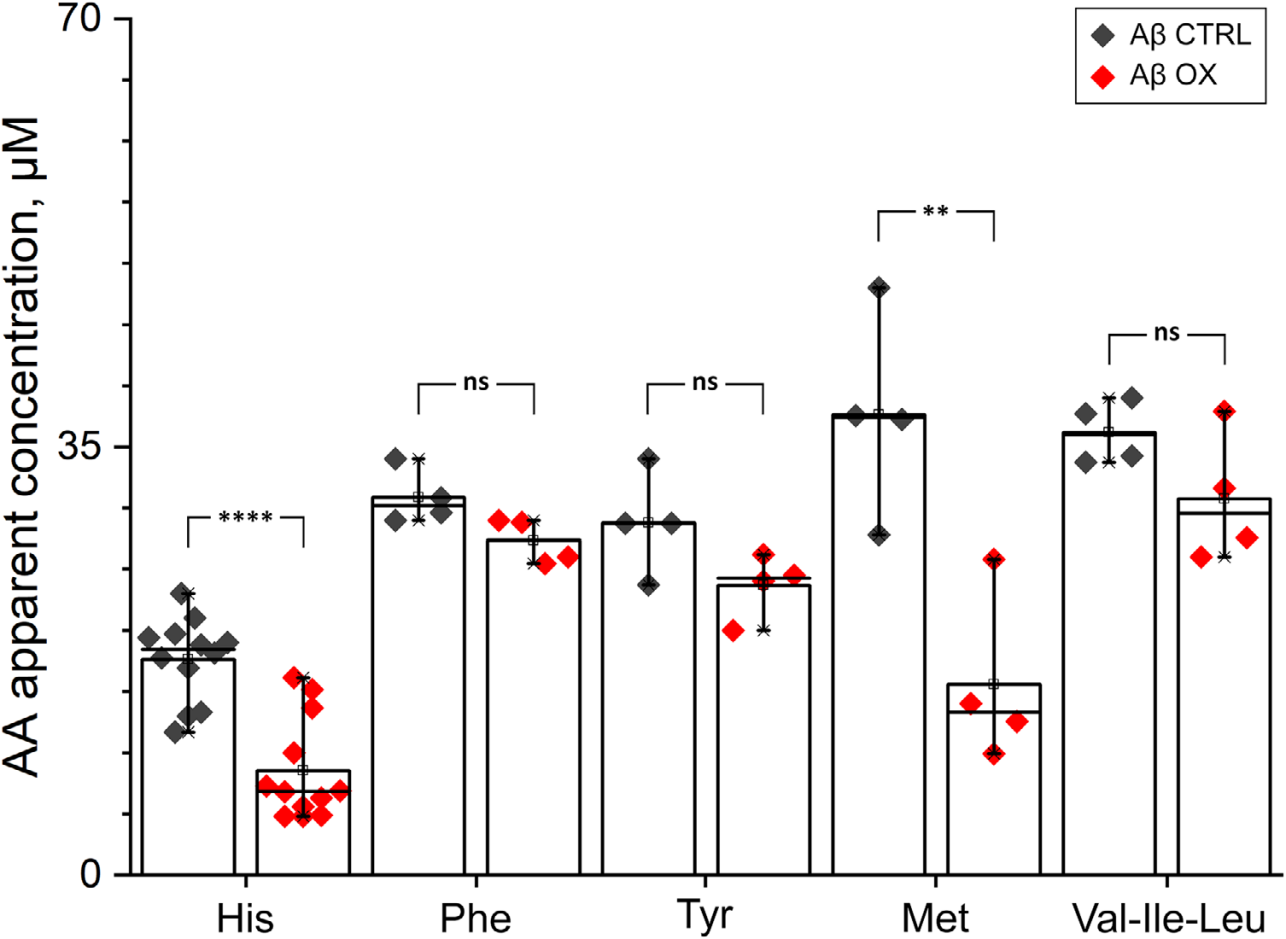
^1^H‐NMR analysis of Aβ fibrils after treatment with 0.5% H_2_O_2_ to mimic the indirect effect of high LET radiation exposure via water radiolysis. Oxidation of both methionine and histidine residues is observed, consistent with literature reports. Since methionine oxidation was not detected in neutron‐irradiated samples under any condition, these results support the hypothesis that histidine oxidation induced by BNCT occurs via a direct and residue‐specific mechanism. *n* = 4 (Aβ CTRL), 10 (Aβ OX). Data are shown as mean ± SD. Statistical comparisons between two independent groups were performed using a two‐tailed unpaired Student's t‐test (see Table S3). ns, *P* ≥ 0.05; *p < 0.05; **p < 0.01; ***p < 0.001; ****p < 0.0001.

Along this line, DOSY measurements showed the clear shift of the Met35 CH_3_γ protons upon H_2_O_2_ treatment, due to oxidation (Figure S12). At variance, Met sidechain is unaffected by neutron irradiation. The absence of NMR signals indicating methionine oxidation under any irradiation condition supports the hypothesis that the observed histidine oxidation is both direct and highly specific, highlighting the selective effect of high LET ionizing radiations generated during the neutron capture reaction. This interpretation is corroborated by the location of the curcumin binding site in the Aβ fibrils. In fact, curcumin recognizes repetitive β‐sheet structures, specifically involving residues 12 and 17–21, as demonstrated by solid‐state ^13^C‐NMR [117], which are in close proximity to the His13‐His14 pair within the flexible loop region connecting the two β‐sheets. This loop is critical for Aβ ion channel activity, metal ion binding (particularly Zn and Cu), and interactions with ganglioside GM1. Literature indicates that histidine is the key amino acid for the interaction of Aβ oligomers with neuronal cell membranes [118, 119, 120]. Consequently, the oxidation of histidine reduces the peptide affinity for gangliosides or other negatively charged groups on membranes, thereby decreasing the formation of Ca^2+^ permeable pores responsible for Aβ‐associated neurotoxicity. Therefore, histidine oxidation can be considered a potential therapeutic target for Alzheimer's Disease.

### 2.7 NanoHPLC‐HRMS

The histidine oxidation was confirmed by nanoHPLC‐HRMS (nanoHigh Performance Liquid Chromatography‐High Resolution Mass Spectrometry).

Tryptic digestion of Aβ protein led to four peptides in a mass range between 319 and 669 *m/z* with z = 2. Each peptide, after high energy collision induced fragmentation, gives a peculiar tandem MS spectrum characterized by the presence of the so‐called b and y ions [121]. All the fragmentation patterns of the peptides are reported in Figures S15–S17. Oxidation of histidine residues was observed in the peptide His6–Lys16, potentially affecting His6, His13, and His14, and giving rise to mono‐, di‐, or tri‐oxidized forms.

**FIGURE 10:**
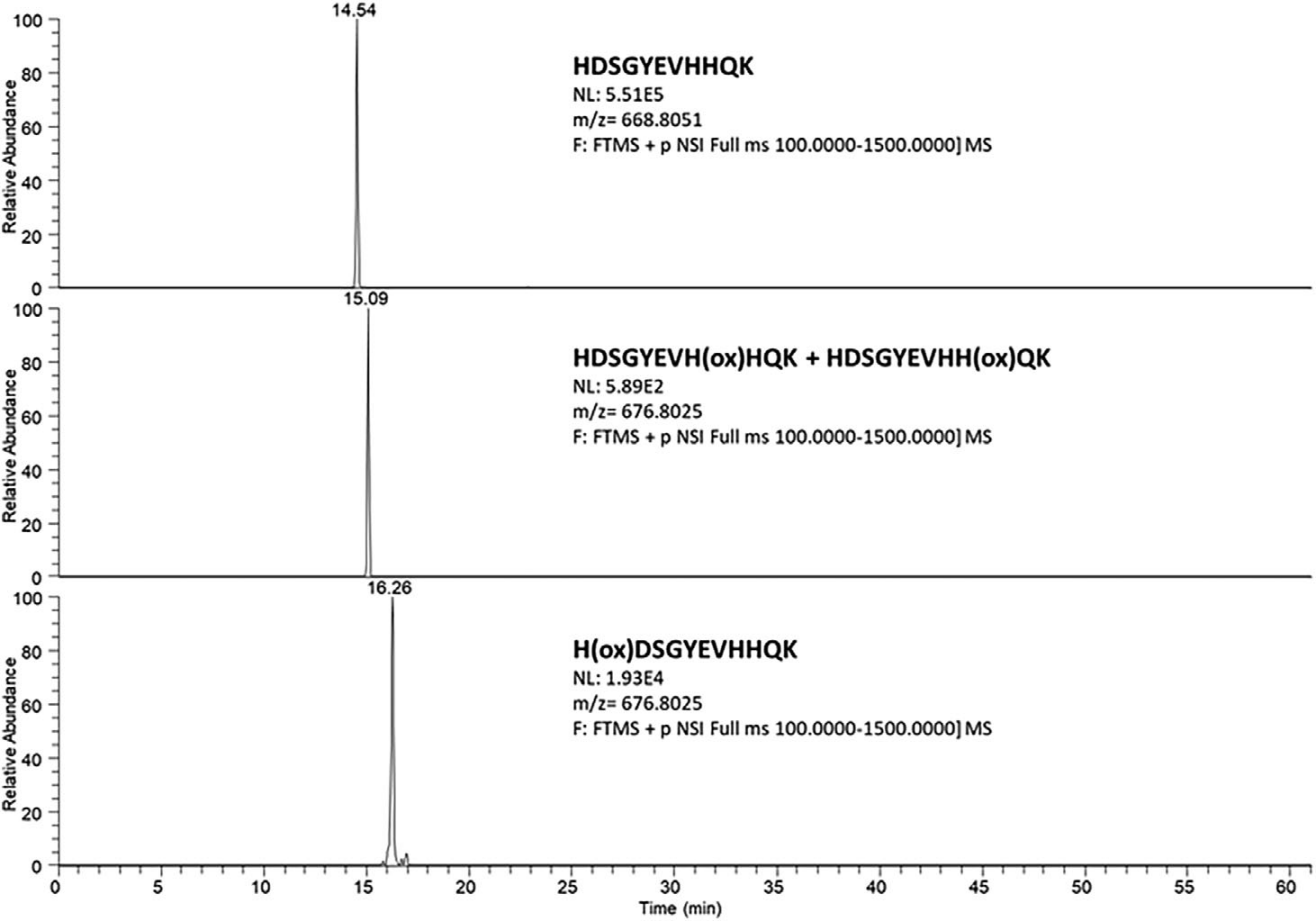
Chromatographic separation of peptide HDSGYEVHHQK unmodified (Rt = 14.54 min) and mono oxidated. At Rt = 15.09 min peptides with the oxidation on His13 and His14 co‐eluted; at Rt = 16.26 min peptide with the oxidation on His6 eluted.

For the recognition of the oxidation site, the manual interpretation and amino acids sequence of the MS/MS spectra were performed. The manual interpretation is based on the comparison between the obtained product ions vs. in silico simulated ones. For the in silico simulation, we used MS‐Digest, a tool of the free software Protein Prospector [122]. The results confirmed the presence of the oxidated forms together with unmodified peptide. We found that mono‐oxidation occurred both on His6, His13 and His14. As Figure 10 shows, the peptide H(ox)DSGYEVHHQK resulted in a chromatographic peak eluted with retention time Rt = 16.26 min, while peptides HDSGYEVH(ox)HQK and HDSGYEVHH(ox)QK co‐eluted at Rt = 15.09 min. The unmodified compound eluted at 14.54 min. The detection of diagnostic b and y ions in the MS/MS spectra corroborated the assignment of the oxidation sites (Figure S13). A similar evaluation was carried out for the di‐oxidized peptide H(ox)DSGYEVH(ox)HQK, eluting at Rt = 15.07 min.

For a semi‐quantitative assessment of the oxidation level induced by **^10^B‐9** treatment, the area under the curve (AUC) of the oxidized peptides was compared (Table 3).

**TABLE 3:** Area under the curve (AUC) of HDSGYEVHHQK peptide containing different oxidations on histidine.

Sample name	No oxidation ^a^	1 oxidation ^b^	2 oxidations ^c^
9NI	5.8 × 10^5^	6.8 × 10^7^	n.d.
9I‐Ψ_3_	3.9 × 10^5^	1.1 × 10^8^	n.d.

^a)^ AUC of HDSGYEVHHQK.

^b)^ AUC of H(ox)DSGYEVHHQK + HDSGYEVH(ox)HQK + HDSGYEVHH(ox)QK.

^c)^ AUC of H(ox)DSGYEVH(ox)HQK.

The total abundance of oxidized peptides (mono‐ and di‐oxidized) was higher in sample 9I‐Ψ_3_ than in 9NI. This observation was consistent with the other results, showing that one hour of **^10^B‐9** irradiation at Ψ_3_ enhanced the formation of oxidized Aβ species.

Despite these findings, a fraction of the Aβ protein appeared to remain hidden from tryptic digestion. Even though the trypsin‐to‐protein ratio was sufficient for complete digestion, intact Aβ species were still detected in the chromatographic run (Figure S14).

The amount of undigested protein in the **^10^B‐9**‐treated Aβ sample was higher than in the control sample (CNI; data not shown), indicating that the **^10^B‐9** treatment made Aβ less accessible, possibly due to the formation of larger and more stable aggregates, thus confirming the NMR results.

## 3 Conclusion

This study represents the first example of targeted in situ high LET ionizing radiations, generated by neutron capture, being used to induce structural modifications in the Aβ peptide, with the goal of converting aggregated fibrils into innocuous species. This establishes a proof of concept that NCT can be exploited for future therapeutic applications in Alzheimer's disease. Using a carborane‐containing curcuminoid derivative, 2,6‐bis((*E*)‐3,4‐bis((tetrahydroxy)benzylidene)‐4‐((1‐(2‐ethylcarboranyl)‐1*H*‐1,2,3‐triazol‐4‐yl)methoxy)cyclohexan‐1‐one (**^10^B‐9**), a 34% reduction in aggregation was demonstrated by FESEM upon exposure to the highest neutron fluence (Ψ_3_). Furthermore, the study demonstrated that both the carborane moiety and the presence of –OH groups are crucial for achieving the nanomolar affinity of for Aβ fibrils, which is fundamental for this type of targeted therapy. It should be noted, however, that in vitro Aβ fibril constructs, lacking metals and other redox agents, exhibit greater stability than those encountered under physiological conditions. Additionally, limited solubility and variability in sample preparation reproducibility significantly hinder a fully quantitative characterization. Converging evidence from ^1^H‐NMR and mass spectrometry suggests that the high LET ionizing radiations induce histidine oxidation. Interestingly, literature reports [114, 123, 124] the critical role of histidine residues in amyloid‐like aggregation, and the oxidation of even one of the three histidines in the Aβ sequence can alter the peptide architecture and biological behavior, particularly its membrane interaction properties, which are fundamental to its pathogenicity. While the biological effects of such high LET radiations have been extensively studied in the context of DNA damage, their impact on other pathological biological assemblies, such as amyloid fibrils, remains largely unexplored. Our results, therefore, open a new avenue for investigating controlled radiation chemistry as a strategy to modulate pathogenic protein assemblies, with potential therapeutic implications for Alzheimer's disease. Moreover, recognizing AD as a new target for NCT is crucial, given the growing clinical interest in this therapy and the advent of new accelerator‐based neutron sources worldwide. Historically, reliance on reactor‐based neutron sources has limited NCT's broader integration into clinical practice [125, 126, 127]. For clinical translation, both the design of more selective delivery agents and the optimization of epithermal neutron beams with defined characteristics will be essential. In this context, compound **9,** showing nanomolar affinity for Aβ fibrils, emerges as the most promising candidate for future studies aimed at mitigating the neuronal toxicity of Aβ plaques in through this alternative therapeutic strategy. A further advantage of employing a small‐sized ligand for the delivering of carboranes into Aβ fibrils and plaques, is its capacity to generally bind all fibrillar aggregates formed by other proteins, including alpha‐synuclein (αS) condensates [128]. Indeed, recent reports indicate that interactions between Aβ and αS are implicated in the pathogenesis of AD and other neurodegenerative protein‐related co‐pathologies [129, 130, 131]. This broadens the therapeutic potential of compound **9** as boron delivery system, compared to antibodies or peptides that specifically bind only Aβ (1‐42) or Aβ (1‐40), thereby rendering this approach essential for the development of effective therapeutic strategies to combat neurodegeneration.

## 4 Experimental Section

### 4.1 Materials and Instruments for Synthesis and Characterization

*Materials*. Flasks and all equipment used for the generation and reaction of moisture‐sensitive compounds were dried by an electric heat gun under N_2_. All commercially available reagents and solvents were used as received. Anhydrous solvents were purchased by Sigma‐Aldrich and Acros. THF was distilled from Na/benzophenone and CH_2_Cl_2_ from CaH_2_ before use. Decaborane was bought from KATCHEM spol. s r. o. Products were purified by preparative column chromatography either on Macherey‐Nagel silica gel for flash chromatography, 0.04–0.063 mm/230–400 mesh, or for non‐flash chromatography, 0.063–0.2 mm/70–230 mesh. When specified, silica gel was deactivated with 1% of Et_3_N. Reactions were monitored by TLC using Silica gel on TLC‐PET foils Merck, layer thickness 0.2 mm, medium pore diameter 60 Å. Carboranes and their derivatives were visualized on TLC plates using a 5% PdCl_2_ aqueous solution in HCl.

*Instruments*. ^1^H NMR spectra were recorded at NMR Jeol ECZR 600 MHz and Bruker NeoAvance 400, ^13^C NMR spectra at 150 and 100 MHz, ^11^B NMR spectra at 192.5 MHz, in CDCl_3_, CD_3_OD, acetone‐*d*_6_ or DMSO‐*d*_6_. Data were reported as follows: chemical shifts in ppm from tetramethylsilane as the internal standard, integration, multiplicity (*s* = singlet, *d* = doublet, *t* = triplet, *q* = quartet, dd = double‐doublet, *m* = multiplet, br = broad), coupling constants (Hz), and assignment. ^13^C NMR and ^11^B NMR spectra were measured with complete proton decoupling. DEPT experiments were carried out with a DEPT‐135 sequence. Chemical shifts were reported in ppm from the residual solvent as an internal standard. The MS flow‐injection analyses were run on a “Orbitrap IQ‐X high resolution” mass spectrometer (Thermo Fisher Scientific, Rodano, Italy), equipped with a heated electrospray ionization source (HESI). Diluted samples (1:10 in ACN/MeOH) were injected into the flowing solvent (MeOH or ACN:H_2_O, 90:10 (v/v) acidified with 0.1% formic acid) at a flow rate of 300–400 µL/min and delivered directly to the ESI source by a syringe pump at constant flow (15‐30 µL/min). The tuning parameters adopted for the ESI source were: ion spray voltage 3.3 kV (ESI+) and 3.0 kV (ESI‐), and tube lens voltage 60%. The ion transfer tube and the vaporizer temperature were maintained at 275°C and 290°C, respectively. Sheath and auxiliary gases were set at 40 and 10 arb (arbitrary unit), respectively. The mass accuracy of the recorded ions (*vs*. the calculated ones) was <5 mmu (milli‐mass units). Analyses were run using full MS at 150‐2000 *m/z* in either positive or negative ion modes.IR spectra were recorded on a PerkinElmer BX FT‐IR. Melting point analyses were carried out with an SMP3 Bibbi Stuart Scientific system. UHPLC‐MS analyses were performed using a UHPLC Acquity H‐Class coupled with Waters 3100 Mass Detector. Samples were dissolved in ACN or MeOH at a concentration of 1 mg/mL and injected into an ACQUITY UHPLC BEH C18 Column, pore size 130 Å, particle size 1.7 µm, 2.1 mm × 50 mm, following a gradient elution from H_2_O (+ TFA 0.05% v/v):ACN 95:5 to ACN 100% in 8 min. Mass spectra were recorded at an ion spray voltage of 3.0 kV (ESI+), source temperature of 120°C, and desolvation temperature of 250°C.

### 4.2 Materials and Instruments for In Vitro Applications

*Materials* Amyloid‐beta (1–42) was purchased from Anaspec (AS‐20276)/DBA Italia. All reagents, if not specified, were purchased by Sigma‐Aldrich.

*Instruments*. FESEM analyses were carried out using a FEG‐SEM S9000 by Tescan. The measurements were performed with a Schotty emitter (resolution 0.7 nm at 15 KeV and 1.4 nm at 1 KeV), the probe set at 100 pA, and the electron beam energy at 5KeV. The analysis was carried out with an in‐beam SE detector. The microanalysis has been carried out with OXFORD—Detector Ultim Max—Software AZTEC. Fluorometric assays were performed with a Horiba Jobin Yvon spectrofluorometer Fluoromax‐4 (Kyoto, Japan). Spectrophotometric analyses were conducted with a Jenway 6715 UV‐Vis spectrophotometer. Thermal neutron irradiations were carried out at the T.R.I.G.A. Mark II nuclear research reactor of the Applied Nuclear Energy Laboratory (L.E.N.A.) of the University of Pavia, Italy. 1H NMR were recorded at a NMR Bruker Avance 14T (proton frequency, 600 MHz) equipped with a Double Resonance Broadband Probe (BBI). Sample (D_2_O) were analysed using 1D‐NOESY, at 25°C, 256 scans, d1 = 11 s. NanoHPLC‐HRMS was carried out using a Dionex Ultimate 3000 coupled with an Orbitrap Tribrid Fusion mass analyzer (Thermo Scientific, Milan, Italy) was used for the analysis. Purification of the samples for nanoHPLC‐HRMS was performed using Pierce C18 Spin Tips (Thermo Fischer Scientific, Milan, Italy). A Dionex Ultimate 3000 coupled with an Orbitrap Tribrid Fusion mass analyzer (Thermo Scientific, Milan, Italy) was used for the nanoHPLC‐HRMS analysis. Chromatographic separation was achieved with a nano‐C18 column (PepMap 2 µm, 100 Å, 75 µm × 15 cm; Thermo Scientific, Milan, Italy) preceded by a nano‐preconcentration column (C18 PepMap trap cartridge 100 Å, 5 µm, 0.3 mm × 5 mm; Thermo Scientific, Milan, Italy) both operated at 45°C.

Statistical analysis and graphs were obtained using OriginLab software (OriginLab Corporation, Northampton, MA, USA).

### 4.3 Synthesis and Characterization

#### 4.3.1 Preparation of 2‐(C‐o‐Carboranyl)‐1‐Azidoethane (3) and ^10^B‐Enriched 2‐(C‐o‐Carboranyl)‐1‐Azidoethane (**^10^B‐3**)

**SCHEME 7:**
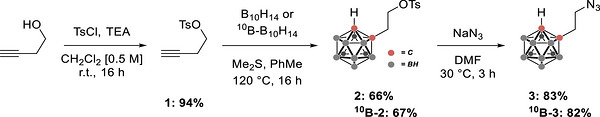
Synthesis of 2‐(*C*‐*o*‐carboranyl)‐1‐azidoethane **(3)** and ^10^B‐Enriched 2‐(*C*‐*o*‐carboranyl)‐1‐azidoethane **(^10^B‐3)**.

*But‐3‐yn‐1‐yl 4‐methylbenzenesulfonate* ***(1)***: A solution of but‐3‐yn‐1‐ol (0.76 mL, 10.0 mmol) and triethylamine (2.8 mL, 20.0 mmol) in 20 mL CH_2_Cl_2_ was cooled to 5°C, and 4‐methylbenzenesulfonyl chloride (2.10 g, 11.0 mmol) was added in small portions. The reaction was allowed to warm up to room temperature and further reacted overnight. 1M HCl was added, and the reaction was vigorously stirred for 10 min before being transferred into a separating funnel. The organic layer was separated, and the aqueous phase was extracted with CH_2_Cl_2_ (2 × 20 mL). The collected organic phases were washed with water and brine, dried on anhydrous Na_2_SO_4_, and concentrated under reduced pressure. The crude mixture was purified using petroleum ether/ethyl acetate 9:1, affording but‐3‐yn‐1‐yl 4‐methylbenzenesulfonate **1** as a colorless oil (2.40 g, 9.40 mmol, 94% yield). The NMR data agree with the literature [132]. ^1^H NMR (600 MHz, CDCl_3_, Me_4_Si) δ: 7.83–7.78 (m, 2H), 7.38–7.33 (m, 2H), 4.11 (t, *J* = 7.1 Hz, 2H), 2.56 (td, *J* = 7.1, 2.7 Hz, 2H), 2.46 (s, 3H), 1.97 (t, *J* = 2.7 Hz, 1H) (Scheme 7).

*Dehydrogenative insertion on tosylate* ***(2)***: In a dried 100 mL round‐bottom flask equipped with a reflux condenser under nitrogen atmosphere, but‐3‐yn‐1‐yl 4‐methylbenzenesulfonate (**1**) (1.1 eq.) and the appropriate borane (1.0 eq.) were suspended in anhydrous toluene. Dimethylsulfide (2.5 eq.) was added, then the mixture was stirred at 120°C for 16 h. After cooling to room temperature, the solvent was evaporated under reduced pressure, and the crude was purified by flash chromatography on silica gel (petroleum ether/dichloromethane 3:2), affording the carborane.

*2‐(C‐o‐carboranyl)‐ethyl‐4‐methylbenzenesulfonate* ***(2)***: According to the general procedure, but‐3‐yn‐1‐yl 4‐methylbenzenesulfonate **1** (1.12 g, 5.5 mmol) was reacted with decaborane (0.61g, 5.0 mmol) and dimethylsulfide (0.78 g, 0.91 mL, 12.5 mmol), affording 2‐(*C*‐*o*‐carboranyl)‐ethyl‐4‐methylbenzenesulfonate **2** (1.13 g, 3.3 mmol, 66% yield) as a white solid. r.f.: 0.33 (petroleum ether/ethyl acetate 6:1). Weakly active under UV light, grey spot with PdCl_2_ staining solution. melting point: 106 – 108°C. ^1^H NMR (600 MHz, CDCl_3_, Me_4_Si) δ: 7.78 (d, *J* = 8.5 Hz, 2H, Ar‐*H*), 7.39 (dm, *J* = 7.9 Hz, 2H, Ar‐H), 4.10 (t, *J* = 6.2 Hz, 2H, TsO‐CH_2_), 3.66 (bs, 1H, C─CH), 2.62 (t, *J* = 6.2 Hz, 2H, TsO‐CH_2_‐CH_2_), 2.48 (s, 3H, CH_3_), 2.81‐1.62 (bm, 10H, B‐H). ^13^C{^1^H} NMR (150 MHz, CDCl_3_, Me_4_Si) δ: 145.7 (Cq), 132.2 (Cq), 130.2 (2xCH), 127.9 (2xCH), 71.1 (Cq), 66.8 (CH_2_), 60.4 (CH), 36.8 (CH_2_), 21.7 (CH_3_). ^11^B NMR (192.5 Hz, CDCl_3_, Me_4_Si) *δ*: ‐2.78 (bs, 1B), ‐5.99 (bs, 1B), ‐10.21 (bs, 2B), ‐12.31 (bs, 2B), ‐13.19 (bs, 2B), −13.70 (bs, 2B). HRMS (ESI) *m/z*: Calcd for C_11_H_22_B_10_O_3_S 389.2202 [M+FA‐H]^−^, found 389.2200 [M+FA‐H]^−^. IR ν_max_ (neat)/ cm^−1^: 3058, 2604, 2575, 1173.

*^10^B‐enriched 2‐(C‐o‐carboranyl)‐ethyl‐4‐methylbenzenesulfonate (* ***^10^B‐2*** *)*: According to the general procedure, but‐3‐yn‐1‐yl 4‐methylbenzenesulfonate **1** (0.49 g, 2.2 mmol) was reacted with ^10^B‐enriched decaborane (0.23 g, 2.0 mmol) and dimethylsulfide (0.31 g, 0.37 mL, 5.0 mmol), affording 2‐(*C*‐*o*‐carboranyl)‐ethyl‐4‐methylbenzenesulfonate **2** (0.45 g, 1.34 mmol, 67% yield) as a white solid. r.f.: 0.33 (petroleum ether/ethyl acetate 6:1). Weakly active under UV light, grey spot with PdCl_2_ staining solution. Melting point: 105°C–106°C. ^1^H NMR (600 MHz, CDCl_3_, Me_4_Si) δ: 7.78 (*d*, *J* = 8.5 Hz, 2H, Ar‐H), 7.39 (dm, *J* = 7.9 Hz, 2H, Ar‐H), 4.10 (t, *J* = 6.2 Hz, 2H, TsO‐CH_2_), 3.66 (bs, 1H, C─CH), 2.62 (t, *J* = 6.2 Hz, 2H, TsO─CH_2─_CH_2_), 2.48 (s, 3H, CH_3_), 2.81‐1.62 (bm, 10H, B‐H). ^13^C{^1^H} NMR (150 MHz, CDCl_3_, Me_4_Si) δ: 145.8 (Cq), 132.3 (Cq), 130.3 (2xCH), 128.1 (2xCH), 71.3 (Cq), 66.9 (CH_2_), 60.5 (CH), 36.9 (CH_2_), 21.9 (CH_3_). HRMS (ESI) *m/z*: Calcd for C_11_H_22_^10^B_10_O_3_S 379.2565 [M+FA‐H]^−^, found 379.2560 [M+FA‐H]^−^. IR ν_max_ (neat)/ cm^−1^: 3061, 2598, 2575, 1165.

*Nuclephilic azidation*: In a dried scintillation vial under nitrogen atmosphere, sodium azide (4.0 eq.) was added to a solution of tosylate **2** or **^10^B‐2** (1.0 eq.) in anhydrous DMF (4.0 mL, 0.1 m). The reaction was stirred at 30°C to completion (usually achieved in 3–5 h). DMF was removed under reduced pressure, and the crude residue was purified by flash chromatography on silica gel (petroleum ether/CH_2_Cl_2_ 4:1), affording the desired azide.

*2‐(C‐o‐carboranyl)‐1‐azidoethane (* ***3*** *)*: According to the general procedure, 2‐(*C*‐*o*‐carboranyl)‐ethyl‐4‐methylbenzenesulfonate **2** (0.34 g, 1.0 mmol) was treated with sodium azide (0.26 g, 4.0 mmol), affording 2‐(*C*‐*o*‐carboranyl)‐1‐azidoethane **3** (0.18 g, 0.83 mmol, 83% yield). Appearance: colorless oil. r.f.: 0.31 (petroleum ether/CH_2_Cl_2_ 4:1). UV inactive, grey spot with PdCl_2_ staining solution. ^1^H NMR (600 MHz, CDCl_3_, Me_4_Si) δ: 3.75 (bs, 1H, C‐CH), 3.48 (t, *J* = 6.9 Hz, 2H, N_3_─CH_2_), 2.47 (t, *J* = 6.9 Hz, 2H, N_3_─CH_2_─CH_2_), 2.88‐1.62 (bm, 10H, B─H). ^13^C{^1^H} NMR (150 MHz, CDCl_3_, Me_4_Si) δ: 72.2 (Cq), 60.8 (CH), 49.6 (CH_2_), 36.8 (CH_2_). ^11^B NMR (192.5 Hz, CDCl_3_, Me_4_Si) δ: ‐2.82 (bs, 1B), −6.15 (bs, 1B), ‐10.25 (bs, 2B), −12.30 (bs, 2B), −13.12 (bs, 2B), ‐13.77 (bs, 2B). HRMS (ESI) *m/z*: Calcd for C_4_H_15_B_10_N_3_ 214.2124 [M+H]^−^, found 214.2123 [M+H]^−^. IR ν_max_ (neat)/ cm^−1^: 3066, 2933, 2575, 2094,1265.

*^10^B‐enriched 2‐(C‐o‐carboranyl)‐1‐azidoethane (* ***^10^B‐3*** *)*: According to the general procedure, ^10^B‐enriched 2‐(*C*‐*o*‐carboranyl)‐ethyl‐4‐methylbenzenesulfonate **^10^B‐2** (0.34 g, 1.0 mmol) was treated with sodium azide (0.26 g, 4.0 mmol), affording ^10^B‐enriched 2‐(*C*‐*o*‐carboranyl)‐1‐azidoethane **^10^B‐3** (0.17 g, 0.82 mmol, 82% yield). Appearance: colorless oil. r.f.: 0.31 (petroleum ether/CH_2_Cl_2_ 4:1). UV inactive, grey spot with PdCl_2_ staining solution. ^1^H NMR (600 MHz, CDCl_3_, Me_4_Si) δ: 3.75 (bs, 1H, C─CH), 3.48 (*t*, *J* = 6.9 Hz, 2H, N_3_─CH_2_), 2.47 (*t*, *J* = 6.9 Hz, 2H, N_3_─CH_2_─CH_2_), 2.88‐1.62 (bm, 10H, B‐H). ^13^C{^1^H} NMR (150 MHz, CDCl_3_, Me_4_Si) δ: 72.2 (Cq), 60.8 (CH), 49.6 (CH_2_), 36.8 (CH_2_). HRMS (ESI) *m/z*: Calcd for C_4_H_15_^10^B_10_N_3_ 204.2487 [M+H]^−^, found 204.2483 [M+H]^−^. IR ν_max_ (neat)/ cm^−1^: 3066, 2928, 2574, 2094, 1258.

#### 4.3.2 Preparation of Alkynes (7 and 7a)

**SCHEME 8:**
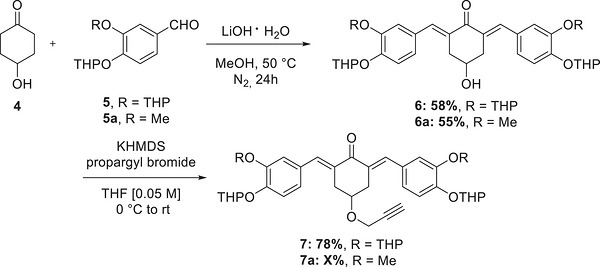
Syntheses of 2,6‐bis((E)‐3,4‐bis((tetrahydro‐2H‐pyran‐2‐yl)oxy)benzylidene)‐4‐(prop‐2‐yn‐1‐yloxy) cyclohexan‐1‐one (7).

*General procedure for the Claisen‐Schmidt condensation*: In a 100 mL round‐bottom flask, 4‐hydroxycyclohexan‐1‐one **4** [91], (1.0 eq.) and the appropriate aldehyde **5** or **5a** [71], (2.0 eq.) were dissolved in MeOH. The solution was heated to 50°C, and then lithium hydroxide monohydrate (3.0 eq.) was added. The solution quickly turned pale yellow and after a few hours became red. The reaction was stirred at 50°C, for 24 h, under an inert atmosphere, then cooled down to room temperature, and methanol was removed under reduced pressure. The residue was added to 75 mL deionized water and extracted with diethyl ether (5×50 mL). The organic phases were washed with brine (1 x 50 mL) and dried on anhydrous Na_2_SO_4_. The volatiles were removed under reduced pressure, and the crude product was purified on deactivated silica gel, affording the desired product **6** or **6a** as a mixture of stereoisomers (Scheme 8).

*2,6‐bis((E)‐3,4‐bis((tetrahydro‐2H‐pyran‐2‐yl)oxy)benzylidene)‐4‐hydroxycyclohexan‐1‐one* ***(6)***: According to the general procedure, 4‐hydroxycyclohexan‐1‐one **4** (0.34 g, 3.0 mmol) and 3,4‐bis((tetrahydro‐2H‐pyran‐2‐yl)oxy)benzaldehyde **5** (2.08 g, 6.0 mmol) were treated with LiOH (0.39 g, 9.0 mmol) in 60 mL of MeOH. The crude was purified on deactivated silica gel using diethyl ether/petroleum ether 4:1, affording 2,6‐bis((*E*)‐3,4‐bis((tetrahydro‐2*H*‐pyran‐2‐yl)oxy)benzylidene)‐4‐hydroxycyclohexan‐1‐one **6** as a mixture of stereoisomers (1.20 g, 1.74 mmol, 58% yield). *Purification: silica gel 70–230 mesh (100 g) packed in a Ø 40 mm column. The crude product was dissolved in a minimal amount of diethyl ether and loaded in the column. The elution afforded unreacted* ***5*** *(which could be reused) first, and product* ***6 s*** *. The progress of purification can be easily followed due to the bright yellow color of chalcone* ***6***. Appearance: foamy bright yellow solid. r.f.: 0.25 (diethyl ether/petroleum ether 4:1). UV active, blue spot with cerium‐molybdate staining solution. ^1^H NMR (600 MHz, CDCl_3_, Me_4_Si) δ: 7.80 (bs, 2H, 2 x C═CH), 7.32‐7.26 (m, 2H, 2 x Ar‐H), 7.18‐7.12 (m, 2H, 2 x Ar‐H), 7.12‐7.06 (m, 2H, 2 x Ar‐H), 5.51 (bs, 1H, O‐CH‐O), 5.48 (bs, 1H, O─CH─O), 5.43 (bs, 1H, O─CH─O), 5.42 (bs, 1H, O‐CH‐O), 4.10 (bs, 1H, CH─OH), 4.05–3.98 (m, 2H, CH_2_‐O), 3.98‐3.91 (m, 2H, CH_2_─O), 3.67–3.55 (m, 4H, 2 x CH_2_─O), 3.24‐3.19 (bs, 1H, HO─CH─CH_2_), 3.19–3.15 (bs, 1H, HO─CH─CH_2_), 3.01–2.90 (m, 4H, 2 x HO─CH─CH_2_), 2.18‐1.55 (m, 25H, CH_2_─CH_2_─CH_2_ + OH). ^13^C{^1^H} NMR (150 MHz, CDCl_3_, Me_4_Si) δ: 188.8 (Cq), 148.5 (Cq), 148.4 (Cq), 148.3 (Cq), 148.2 (Cq), 147.1 (Cq), 146.9 (Cq), 139.1 (CH), 139.1 (CH), 131.2 (Cq), 131.1 (Cq), 130.2 (Cq), 130.0 (Cq), 125.8 (CH), 125.8 (CH), 125.7 (CH), 125.6 (CH), 121.4 (CH), 121.3 (CH), 121.0 (CH), 121.0 (CH), 117.8 (CH), 117.7 (CH), 117.5 (CH), 117.4 (CH), 98.1 (CH), 98.1 (CH), 97.7 (CH), 97.6 (CH), 97.4 (CH), 97.0 (CH), 96.9 (CH), 66.0 (CH), 62.2 (CH_2_), 62.2 (CH_2_), 62.1 (CH_2_), 62.0 (CH_2_), 62.0 (CH_2_), 61.9 (CH_2_), 36.7 (CH_2_), 36.7 (CH_2_), 30.5 (CH_2_), 30.4 (CH_2_), 30.4 (CH_2_), 30.3 (CH_2_), 25.4 (CH_2_), 25.3 (CH_2_), 18.8 (CH_2_), 18.7 (CH_2_), 18.7 (CH_2_), 18.5 (CH_2_). HRMS (ESI) *m/z*: Calcd for C_40_H_50_O_10_ 713.3296 [M+Na]^+^, found 713.3293 [M+Na]^+^. IR ν_max_ (neat)/ cm^−1^: 3466, 2940, 2870, 1711, 1597, 1502, 1356, 1238, 1182, 1069, 1048, 954, 915, 871, 815.

*4‐hydroxy‐2,6‐bis((E)‐3‐methoxy‐4‐((tetrahydro‐2H‐pyran‐2‐yl)oxy)benzylidene) cyclohexan‐1‐one* ***(6a)***: According to the general procedure, 4‐hydroxycyclohexan‐1‐one **4** (0.19 g, 1.67 mmol) and 3‐methoxy‐4‐((tetrahydro‐2H‐pyran‐2‐yl)oxy)benzaldehyde **5a** (0.79 g, 3.34 mmol) were treated with LiOH (0.12 g, 5.01 mmol) in 30 mL of MeOH. The crude was purified on deactivated silica gel using petroleum ether/acetone 2:1, affording 4‐hydroxy‐2,6‐bis((*E*)‐3‐methoxy‐4‐((tetrahydro‐2*H*‐pyran‐2‐yl)oxy)benzylidene)cyclohexan‐1‐one **6a** as a mixture of stereoisomers (0.43 g, 0.79 mmol, 47% yield). Appearance: foamy bright yellow solid. r.f.: 0.24 (petroleum ether/ethyl acetate 1:1). UV active, blue spot with cerium‐molybdate staining solution. ^1^H NMR (400 MHz, CDCl_3_, Me_4_Si) δ: 7.84 (bs, 2H, 2 x C≐CH), 7.16 (*d*, *J* = 8.4 Hz, 2H, 2 x Ar‐H), 7.06 (*d*, *J* = 8.5 Hz, 2H, 2 x Ar‐H), 7.02 (s, 2H, 2 x Ar‐H), 5.47 (*t*, *J* = 3.0 Hz, 2H, 2 x O‐CH‐O), 4.21–4.11 (m, 1H, CH─OH), 3.97 (dt, *J* = 9.8 Hz, 2.7 Hz, 2H, 2 x CH^a^H^b^─O), 3.89 (s, 6H, 2 x CH_3_‐O), 3.62 (dt, *J* = 10.2 Hz, 3.5 Hz, 2H, 2 x CH^a^H^b^‐O), 3.23 (*d*, *J* = 15.0 Hz, 2H, 2 x HO‐CH─CH^a^H^b^), 3.01 (dd, *J* = 15.3 Hz, 8.3 Hz, 2H, HO─CH─CH^a^H^b^), 2.13‐1.58 (m, 13H, CH_2_‐CH_2_‐CH_2_ + OH). ^13^C{^1^H} NMR (100 MHz, CDCl_3_, Me_4_Si) δ: 188.7 (Cq), 150.0 (Cq), 147.3 (Cq), 139.3 (CH), 131.1 (Cq), 130.0 (Cq), 123.9 (CH), 117.0 (CH), 115.1 (CH), 97.4 (CH), 66.0 (CH), 62.3 (CH_2_), 56.4 (CH_3_), 36.8 (CH_2_), 36.8 (CH_2_), 30.4 (CH_2_), 25.3 (CH_2_), 18.9 (CH_2_). HRMS (ESI) *m/z*: Calcd for C_32_H_38_O_8_ 551.2639 [M‐H]^+^, found 551.2642 [M‐H]^+^. IR ν_max_ (neat)/ cm^−1^: 3466, 2940, 2871, 1596, 1578, 1507, 1465, 1453, 1240, 1201, 1138, 1120, 1034, 957, 919.

*General procedure for the propargylation reaction*: In a Schlenk flask under an inert nitrogen atmosphere, the appropriate alcohol **6** or **6a** (1.0 eq.) was dissolved in anhydrous THF (0.05 m). The solution was cooled to 0°C, and 1.0 m KHMDS solution (1.2 eq.) was added dropwise. The solution was vigorously stirred for 15 min, then 80% w/w propargyl bromide in toluene (2.0 eq.) was added, and the reaction was further stirred for 3.5 h at room temperature. The excess base was quenched by the addition of 500 µL of deionized water, followed by anhydrous Na_2_SO_4_. After vigorously stirring for 10 min, the content of the flask was filtered and the volatiles removed under removed pressure. The crude product was purified on deactivated silica gel, affording the desired product **7** or **7a** as a mixture of stereoisomers.

*2,6‐bis((E)‐3,4‐bis((tetrahydro‐2H‐pyran‐2‐yl)oxy)benzylidene)‐4‐(prop‐2‐yn‐1‐yloxy)cyclohexan‐1‐one* ***(7)***: According to the general procedure, 2,6‐bis((*E*)‐3,4‐bis((tetrahydro‐2*H*‐pyran‐2‐yl)oxy)benzylidene)‐4‐hydroxycyclohexan‐1‐one **6** (0.69 g, 1.0 mmol) was treated with 1.0 m KHMDS solution (1.2 mL, 1.2 mmol) in anhydrous THF (20.0 mL, 0.05 M). Then 80% w/w propargyl bromide in toluene (0.23 mL, 2.0 mmol) was added. The crude was purified on deactivated silica gel using diethyl ether/petroleum ether 2:1, affording 2,6‐bis((*E*)‐3,4‐bis((tetrahydro‐2*H*‐pyran‐2‐yl)oxy)benzylidene)‐4‐(prop‐2‐yn‐1‐yloxy) cyclohexan‐1‐one **7** as a mixture of stereoisomers (0.39 g, 0.53 mmol, 53% yield). *Note*: Highly pure alcohol **6** produced a yellow solution upon the addition of the base. In the presence of impurities, the solution turned orange to brown, resulting in a drop in the yield of product **7**. According to this reaction, **7** is usually recovered in a 40%–55% range of yield. *Purification*: silica gel 70–230 mesh (100 g) packed in a Ø 40 mm column. The crude product was dissolved in a minimal amount of diethyl ether and loaded into the column. The progress of the purification can be easily followed due to the bright yellow color of product **7**. Appearance: foamy bright yellow solid. r.f.: 0.3 (diethyl ether/petroleum ether 2:1). UV active, blue spot with cerium‐molybdate staining solution. ^1^H NMR (600 MHz, CDCl_3_, Me_4_Si) δ: 7.80 (bs, 2H, 2 x C═C*H*), 7.30–7.28 (m, 2H, 2 x Ar‐*H*), 7.17‐7.15 (m, 2H, 2 x Ar‐*H*), 7.12‐7.10 (m, 2H, 2 x Ar‐*H*), 5.51 (bs, 1H, O─C*H─*O), 5.49 (bs, 1H, O─C*H─*O), 5.44 (bs, 1H, O─C*H─*O), 5.42 (bs, 1H, O─C*H─*O), 4.09‐4.07 (m, 1H, C*H*‐OH), 4.04‐3.93 (m, 2H, C*H_2_*‐O), 3.98‐3.91 (m, 2H, C*H_2_*‐O), 3.64‐3.60 (m, 6H, 3 x C*H_2_* ─O), 3.24‐3.18 (m, 2H, 2 x C*H^a^* H‐O), 3.07‐3.00 (m, 2H, 2 x CH^a^ *H^b^*‐O), 2.35–2.33 (m, 1H, C≡CH), 2.06‐1.85 (m, 12H, C*H_2_* ─C*H_2_* ─C*H_2_*), 1.75‐1.60 (m, 12H, C*H_2_* ─C*H_2_* ─C*H_2_*). ^13^C{^1^H} NMR (150 MHz, CDCl_3_, Me_4_Si) δ: 188.7 (Cq), 148.5 (Cq), 148.3 (Cq), 147.1 (Cq), 147.1 (Cq), 146.9 (Cq), 146.9 (Cq), 138.9 (CH), 138.8 (CH), 131.0 (Cq), 131.0 (Cq), 130.9 (Cq), 130.2 (Cq), 130.0 (Cq), 125.8 (CH), 125.7 (CH), 125.6 (CH), 125.5 (CH), 121.4 (CH), 121.1 (CH), 121.02 (CH), 117.7 (CH), 117.4 (CH), 98.1 (CH), 98.1 (CH), 97.7 (CH), 97.6 (CH), 97.4 (CH), 97.4 (CH), 97.0 (CH), 97.0 (CH), 79.7 (Cq), 74.6 (CH), 71.8 (CH), 71.7 (CH), 71.6 (CH), 62.2 (CH_2_), 62.1 (CH_2_), 62.0 (CH_2_), 62.0 (CH_2_), 61.9 (CH_2_), 55.6 (CH_2_), 33.5 (CH_2_), 33.5 (CH_2_), 33.4 (CH_2_), 30.5 (CH_2_), 30.4 (CH_2_), 30.4 (CH_2_), 30.4 (CH_2_), 25.4 (CH_2_), 25.3 (CH_2_), 18.8 (CH_2_), 18.7 (CH_2_), 18.7 (CH_2_), 18.5 (CH_2_). HRMS (ESI) *m/z*: Calcd for C_43_H_52_O_10_ 751.3453 [M‐Na]^+^, found 751.3456 [M‐Na]^+^. IR *ν*_max_ (neat)/ cm^−1^: 2940, 2870, 1598, 1504, 1453, 1355, 1240, 1201, 1119, 1035, 1021, 965, 916, 872.

*2,6‐bis((E)‐3‐methoxy‐4‐((tetrahydro‐2H‐pyran‐2‐yl)oxy)benzylidene)‐4‐(prop‐2‐yn‐1‐yloxy) cyclohexan‐1‐one* ***(7a)***: According to the general procedure, 4‐hydroxy‐2,6‐bis((*E*)‐3‐methoxy‐4‐((tetrahydro‐2*H*‐pyran‐2‐yl)oxy)benzylidene)cyclohexan‐1‐one **6a** (0.17 g, 0.30 mmol) was treated with 1.0 m KHMDS solution (0.36 mL, 0.36 mmol) in anhydrous THF (6.0 mL, 0.05 m). Then 80% w/w propargyl bromide in toluene (0.067 mL, 0.6 mmol) was added. The crude was purified on deactivated silica gel using petroleum ether/acetone 4:1, affording 2,6‐bis((*E*)‐3‐methoxy‐4‐((tetrahydro‐2*H*‐pyran‐2‐yl)oxy)benzylidene)‐4‐(prop‐2‐yn‐1‐yloxy) cyclohexan‐1‐one **7a** as a mixture of stereoisomers (0.083 g, 0.14 mmol, 47% yield). Appearance: foamy bright yellow solid. r.f.: 0.57 (petroleum ether/acetone 7:3). UV active, blue spot with cerium‐molybdate staining solution. ^1^H NMR (400 MHz, acetone‐d6) δ: 7.72 (bs, 2H, 2 x C═CH), 7.19 (*d*, *J* = 8.4 Hz, 2H, 2 x Ar‐H), 7.17 (*d*, *J* = 1.6 Hz, 2H, 2 x Ar‐H), 7.11 (dd, *J* = 8.5 Hz, 1.6 Hz, 2H, 2 x Ar‐H), 5.49 (t, *J* = 2.8 Hz, 2H, 2 x O─CH─O), 4.12–4.09 (m, 1H, CH─O─CH_2_), 4.09 (*d*, *J* = 2.4 Hz, 2H, CH_2_─O), 3.95–3.86 (m, 2H, 2 x CH^a^H‐O), 3.90 (s, 6H, 2 x CH_3_‐O), 3.57 (dt, *J* = 11 Hz, 4.1 Hz, 2H, 2 x CH^a^H─O), 3.21 (*d*, *J* = 2.4 Hz, 4H, 2 x CH_2_‐CH‐O‐CH_2_), 2.81 (t, *J* = 2.2 Hz, 1H, C≡CH), 2.04–1.94 (m, 2H, 2 x O‐CH_2_‐CH^a^H^b^‐CH_2_), 1.89‐1.81 (m, 4H, 2 x O‐CH‐CH_2_), 1.71‐1.54 (m, 6H, 2 x O‐CH_2_‐CH^a^H^b^‐CH_2_). ^13^C{^1^H} NMR (100 MHz, acetone‐d6) δ: 188.3 (Cq), 151.1 (Cq), 151.0 (Cq), 148.2 (Cq), 138.4 (CH), 138.4 (CH), 132.3 (Cq), 130.9 (Cq), 124.3 (CH), 124.3 (CH), 117.9 (CH), 117.9 (CH), 115.8 (CH), 115.8 (CH), 97.9 (CH), 97.8 (CH), 80.9 (CH), 75.7 (CH), 71.9 (CH), 71.9 (CH), 71.9 (CH), 62.4 (CH_2_), 56.4 (CH_3_), 55.8 (CH_2_), 33.8 (CH_2_), 31.0 (CH_2_), 26.0 (CH_2_), 19.5 (CH_2_). HRMS (ESI) *m/z*: Calcd for C_35_H_40_O_8_ 589.2796 [M‐H]^+^, found 589.2800 [M‐H]^+^. IR ν_max_ (neat)/ cm^−1^: 3264, 2938, 2872, 1596, 1579, 1508, 1465, 1453, 1417, 1356, 1260, 1240, 1201, 1138, 1120, 1034, 1021, 956, 918, 872, 737.

#### 4.3.3 Preparation of 2,6‐Bis((E)‐3,4‐Bis((Tetrahydroxy)Benzylidene)‐4‐((1‐(2‐Ethylcarboranyl)‐1H‐1,2,3‐triazol‐4‐yl)Methoxy)Cyclohexan‐1‐one (**9**, **^10^B‐9**), 2,6‐Bis((E)‐3‐Methoxy‐4‐(Tetrahydroxy)Benzylidene)‐4‐((1‐(2‐Ethylcarboranyl)‐1H‐1,2,3‐triazol‐4‐yl)Methoxy)Cyclohexan‐1‐One (**9a**) and 2,6‐Bis((E)‐3,4‐Dihydroxybenzylidene)‐4‐(Prop‐2‐yn‐1‐yloxy)Cyclohexan‐1‐One (**7b**)

**SCHEME 9:**
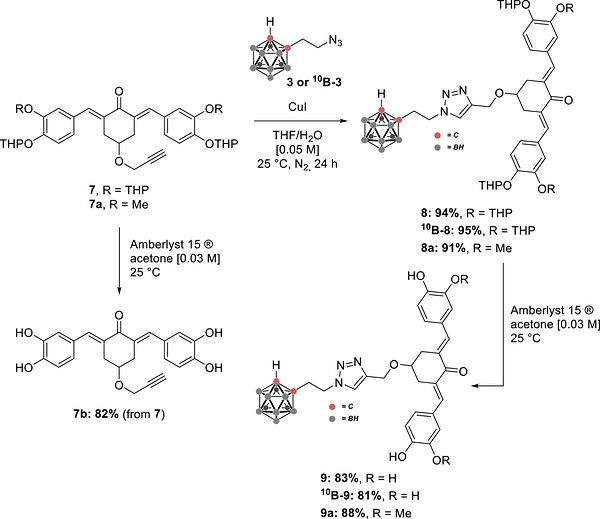
Syntheses of 9,^10^B‐9, 9a and 7b.

*CuCAAC between alkyne* ***7*** *and azide* ***3*** *or* ***^10^B‐3***: In a flask under a nitrogen atmosphere, the appropriate alkyne **7** or **7a** (1.0 eq.) and azide **3** or **^10^B‐3** (1.0 eq.) were suspended in an equimolar mixture of THF and deionized water. Oxygen was removed by bubbling nitrogen or argon for 10 min, then anhydrous copper iodide (1.0 eq.) was added in one single portion. The reaction was vigorously stirred for 24 h, at 25°C and under an inert atmosphere (nitrogen of argon). Upon removal of the volatiles, the crude product was purified by flash chromatography on deactivated silica gel, affording the corresponding triazole. *Purification*: silica gel 230–400 mesh (25 g) packed in a Ø 55 mm column and deactivated with Et_3_N (1% w/w). The crude product was adsorbed on silica gel and loaded in the column (Scheme 9).

2,6‐bis((E)‐3,4‐bis((tetrahydro‐2H‐pyran‐2‐yl)oxy)benzylidene)‐4‐((1‐(2‐ethylcarboranyl)‐1H‐1,2,3‐triazol‐4‐yl)methoxy)cyclohexan‐1‐one (**8**): According to the general procedure, 2,6‐bis((E)‐3,4‐bis((tetrahydro‐2H‐pyran‐2‐yl)oxy)benzylidene)‐4‐(prop‐2‐yn‐1‐yloxy) cyclohexan‐1‐one **7** (0.38 g, 0.52 mmol) was reacted with 2‐(C‐o‐carboranyl)‐1‐azidoethane **3** (0.11 g, 0.52 mmol) in 10 mL of a THF:H_2_O 1:1 mixture in the presence of CuI (0.099 g, 0.52 mmol). The crude was purified on deactivated silica gel using dichlorometane/methanol 75:1 as the eluent, affording triazole **8** as a mixture of diastereomers (0.45 g, 0.48 mmol, 94% yield). Appearance: foamy bright yellow solid. r.f.: 0.24 (dichloromethane/methanol 75:1). UV active, grey spot with PdCl_2_ staining solution. ^1^H NMR (600 MHz, CDCl_3_, Me_4_Si) δ: 7.76‐7.69 (bs, 2H, 2 x C═CH), 7.33‐7.27 (m, 1H, triazole Ar‐H), 7.25–7.20 (m, 2H, 2 x Ar‐H), 7.14–7.09 (m, 2H, 2 x Ar‐H), 7.08‐7.01 (m, 2H, 2 x Ar‐H), 5.50–5.44 (m, 2H, 2 x O─CH─O), 5.42‐5.39 (m, 1H, O─CH─O), 5.37 (dt, 1H, *J* = 9.2, 3.2 Hz, O‐CH‐O), 4.46‐4.41 (m, 2H, triazole‐CH_2_─O), 4.28–4.18 (m, 2H, triazole‐CH_2_‐CH_2_), 4.06–3.82 (m, 6H, 2 x O‐CH_2_ + O‐CH + carborane CH), 3.66‐3.53 (m, 4H, 2 x O─CH_2_), 3.15‐3.01 (m, 4H, CH_2_‐CH‐CH_2_), 2.70–2.61 (m, 2H triazole‐CH_2_─CH_2_), 2.08‐1.49 (m, 24H, 4 x CH_2_─CH_2_─CH_2_), 2.85‐1.40 (bs, 10H, 10 x BH). ^13^C{^1^H} NMR (150 MHz, CDCl_3_, Me_4_Si) δ: 207.0 (Cq), 188.8 (Cq), 148.5 (Cq), 148.4 (Cq), 148.3 (Cq), 148.2 (Cq), 147.1 (Cq), 147.0 (Cq), 146.9 (Cq), 146.9 (Cq), 145.6 (Cq), 145.6 (Cq), 138.6 (CH), 138.5 (CH), 130.9 (Cq), 130.9 (Cq), 130.8 (Cq), 129.9 (Cq), 129.9 (Cq), 129.9 (Cq), 129.8 (Cq), 125.5 (CH), 125.3 (CH), 123.2 (CH), 123.1 (CH), 123.1 (CH), 121.4 (CH), 121.1 (CH), 121.0 (CH), 120.8 (CH), 117.7 (CH), 117.6 (CH), 117.4 (CH), 117.4 (CH), 117.4 (CH), 98.1 (CH), 98.1 (CH), 97.7 (CH), 97.6 (CH), 97.4 (CH), 97.3 (CH), 97.0 (CH), 97.0 (CH), 72.2 (CH), 71.2 (Cq), 62.2 (CH_2_), 62.2 (CH_2_), 62.08 (CH_2_), 62.0 (CH_2_), 62.0 (CH_2_), 62.0 (CH_2_), 61.9 (CH_2_), 61.9 (CH_2_), 61.9 (CH_2_), 48.3 (CH_2_), 36.8 (CH_2_), 36.8 (CH_2_), 33.4 (CH_2_), 33.3 (CH_2_), 30.4 (CH_2_), 30.4 (CH_2_), 30.3 (CH_2_), 30.3 (CH_2_), 25.3 (CH_2_), 25.2 (CH_2_), 18.8 (CH_2_), 18.8 (CH_2_), 18.7 (CH_2_), 18.7 (CH_2_), 18.6 (CH_2_), 18.5 (CH_2_). ^11^B NMR (192.5 MHz, CDCl_3_, Me_4_Si) δ: ‐3.07 (bs, 1B), ‐6.02 (bs, 1B), ‐10.10 (bs, 2B), ‐13.09 (bs, 6B). HRMS (ESI) m/z: Calcd for C_47_H_67_B_10_N_3_O_10_ 944.5830 [M+H]^+^, found 944.5830 [M+H]^+^. IR ν_max_ (neat)/ cm^−1^: 2941, 2872, 2583 (BH), 1711, 1579, 1507, 1465, 1452, 1415, 1356, 1241, 1201, 1136, 1072, 1032, 1021, 953, 918, 871.

^10^B‐enriched 2,6‐bis((E)‐3‐methoxy‐4‐(tetrahydro‐2H‐pyran‐2‐yl)oxy)benzylidene)‐4‐((1‐(2‐ethylcarboranyl)‐1H‐1,2,3‐triazol‐4‐yl)methoxy)cyclohexan‐1‐one (**^10^B‐8**): According to the general procedure, 2,6‐bis((E)‐3,4‐bis((tetrahydro‐2H‐pyran‐2‐yl)oxy)benzylidene)‐4‐(prop‐2‐yn‐1‐yloxy) cyclohexan‐1‐one **7** (0.38 g, 0.52 mmol) was reacted with ^10^B‐enriched 2‐(C‐o‐carboranyl)‐1‐azidoethane **^10^B‐3** (0.11 g, 0.52 mmol), in 10 mL of a THF:H_2_O 1:1 mixture in the presence of CuI (0.099 g, 0.52 mmol). The crude was purified on deactivated silica gel using dichlorometane/methanol 75:1 as the eluent, affording ^10^B‐enriched triazole **^10^B‐8** as a mixture of diastereomers (0.46 g, 0.49 mmol, 95% yield). Appearance: foamy bright yellow solid. r.f.: 0.24 (dichloromethane/methanol 75:1). UV active, grey spot with PdCl_2_ staining solution. ^1^H NMR (400 MHz, CDCl_3_, Me_4_Si) δ: 7.76‐7.69 (bs, 2H, 2 x C═CH), 7.33‐7.27 (m, 1H, triazole Ar‐H), 7.25–7.20 (m, 2H, 2 x Ar‐H), 7.14–7.09 (m, 2H, 2 x Ar‐H), 7.08–7.01 (m, 2H, 2 x Ar‐H), 5.52‐5.48 (m, 2H, 2 x O─CH─O), 5.43–5.39 (m, 2H, O─CH─O), 4.52 (bs, 2H, triazole‐CH_2_─O), 4.33–4.29 (m, 2H, triazole‐CH_2_‐CH_2_), 4.04‐3.92 (m, 6H, 2 x O─CH_2_ + O─CH), 3.83 (bs, 1H, carborane CH), 3.65‐3.59 (m, 4H, 2 x O─CH_2_), 3.15–3.04 (m, 4H, CH_2_‐CH‐CH_2_), 2.79‐2.65 (m, 2H triazole‐CH_2_─CH_2_), 2.08‐1.49 (m, 24H, 4 x CH_2_─CH_2_─CH_2_), 2.85–1.40 (bs, 10H, 10 x BH). ^13^C{^1^H} NMR (150 MHz, CDCl_3_, Me_4_Si) δ: 207.1 (Cq), 188.8 (Cq), 148.5 (Cq), 148.4 (Cq), 148.3 (Cq), 148.2 (Cq), 147.2 (Cq), 147.1 (Cq), 147.0 (Cq), 146.9 (Cq), 145.7 (Cq), 145.7 (Cq), 138.6 (CH), 138.6 (CH), 130.9 (Cq), 130.9 (Cq), 130.9 (Cq), 130.0 (Cq), 129.9 (Cq), 129.8 (Cq), 129.8 (Cq), 125.5 (CH), 125.4 (CH), 123.2 (CH), 123.2 (CH), 123.1 (CH), 121.4 (CH), 121.1 (CH), 121.1 (CH), 120.8 (CH), 117.8 (CH), 117.6 (CH), 117.5 (CH), 117.5 (CH), 117.4 (CH), 98.2 (CH), 98.1 (CH), 97.7 (CH), 97.7 (CH), 97.4 (CH), 97.3 (CH), 97.0 (CH), 97.0 (CH), 72.3 (CH), 71.3 (Cq), 62.2 (CH_2_), 62.2 (CH_2_), 62.1 (CH_2_), 62.0 (CH_2_), 62.0 (CH_2_), 62.0 (CH_2_), 61.9 (CH_2_), 61.9 (CH_2_), 61.9 (CH_2_), 48.3 (CH_2_), 36.9 (CH_2_), 36.8 (CH_2_), 33.4 (CH_2_), 33.3 (CH_2_), 30.4 (CH_2_), 30.4 (CH_2_), 30.3 (CH_2_), 30.3 (CH_2_), 25.3 (CH_2_), 25.2 (CH_2_), 18.8 (CH_2_), 18.8 (CH_2_), 18.7 (CH_2_), 18.7 (CH_2_), 18.6 (CH_2_), 18.5 (CH_2_). HRMS (ESI) m/z: Calcd for C_47_H_67_^10^B_10_N_3_O_10_ 934.6193 [M+H]^+^, found 934.6193 [M+H]^+^. IR ν_max_ (neat)/ cm^−1^: 2940, 2871, 2581 (BH), 1710, 1579, 1506, 1464, 1452, 1415, 1357, 1240, 1201, 1136, 1072, 1033, 1021, 953, 917, 871.

2,6‐bis((E)‐3‐methoxy‐4‐(tetrahydro‐2H‐pyran‐2‐yl)oxy)benzylidene)‐4‐((1‐(2‐ethylcarboranyl)‐1H‐1,2,3‐triazol‐4‐yl)methoxy)cyclohexan‐1‐one (**8a**): According to the general procedure, 2,6‐bis((E)‐3‐methoxy‐4‐((tetrahydro‐2H‐pyran‐2‐yl)oxy)benzylidene)‐4‐(prop‐2‐yn‐1‐yloxy) cyclohexan‐1‐one **7a** (0.052 g, 0.087 mmol) was reacted with 2‐(C‐o‐carboranyl)‐1‐azidoethane **3** (0.019 g, 0.087 mmol), in 1.7 mL of a THF:H_2_O 1:1 mixture in the presence of CuI (0.017 g, 0.087 mmol). The crude was purified on deactivated silica gel using dichlorometane/methanol 75:1 as the eluent, affording triazole **8a** as a mixture of diastereomers (0.063 g, 0.079 mmol, 91% yield). Appearance: foamy bright yellow solid. r.f.: 0.41 (dichloromethane/methanol 98:2). UV active, grey spot with PdCl_2_ staining solution. ^1^H NMR (600 MHz, CDCl_3_, Me_4_Si) δ: 7.79 (bs, 2H, 2 x C═CH), 7.38 (bs, 1H, triazole Ar‐H), 7.15 (dd, *J* = 8.3 Hz, 2.1 Hz, 2H, 2 x Ar‐H), 7.04 (*d*, *J* = 8.0 Hz, 2H, 2 x Ar‐H), 6.99 (s, 2H, 2 x Ar‐H), 5.49‐5.45 (m, 2H, 2 x O─CH─O), 4.55 (bs, 2H, triazole─CH_2_─O), 4.37‐4.28 (m, 2H, triazole─CH_2_─CH_2_), 4.02–3.92 (m, 3H, 2 x O‐CH^a^H^b^ + O‐CH), 3.88 (s, 6H, 2 x O─CH_3_) 3.78 (bs, 1H, carborane CH), 3.66‐3.58 (m, 2H, 2 x O─CH^a^H^b^), 3.17‐3.07 (m, 4H, CH_2_─CH‐O), 2.84‐2.76 (m, 2H triazole‐CH_2_‐CH_2_), 2.13‐1.54 (m, 12H, 2 x CH_2_─CH_2_─CH_2_), 2.75‐1.50 (bs, 10H, 10 x BH). ^13^C{^1^H} NMR (100 MHz, acetone‐d6) δ: 188.4 (Cq), 151.1 (Cq), 151.1 (Cq), 148.2 (Cq), 145.8 (Cq), 138.3 (CH), 132.5 (Cq), 130.9 (Cq), 130.9 (Cq), 124.3 (CH), 124.3 (CH), 118.0 (CH), 118.0 (CH), 115.8 (CH), 115.8 (CH), 97.9 (CH), 76.3 (CH), 72.3 (CH), 63.7 (CH), 62.5 (CH_2_), 62.4 (CH_2_), 62.2 (CH_2_), 62.1 (CH_2_), 56.5 (CH_3_), 48.8 (CH_2_), 37.5 (CH_2_), 37.5 (CH_2_), 34.0 (CH_2_), 26.0 (CH_2_), 19.5 (CH_2_). ^11^B NMR (128 MHz, acetone‐d6) δ: ‐2.03 (bs, 1B), ‐4.89 (bs, 1B), ‐8.82 (bs, 2B), ‐11.05 (bs, 4B), ‐12.25 (bs, 2B). HRMS (ESI) m/z: Calcd for C_39_H_55_B_10_N_3_O_8_ 804.4992 [M+H]^+^, found 804.4994 [M+H]^+^. IR νmax (neat)/ cm^−1^: 2941, 2870, 2585 (BH), 1710, 1578, 1507, 1464, 1453, 1416, 1357, 1239, 1202, 1136, 1072, 1032, 1021, 954, 917, 871.

*Catechol deprotection*: In a round‐bottom flask, protected catechol (**7**, **8**, **^10^B‐8** or **8a**) (1.0 eq.) was dissolved in acetone (0.03 M). Amberlyst 15 (10% w/w) was added in one single portion, and the suspension was vigorously stirred at 25°C until completion (as confirmed by ESI‐MS analysis on the reaction mixture). Amberlyst was filtered off, and the volatiles were removed affording a crude product. Trituration using dichloromethane, followed by centrifugation or filtration of the solid residue afforded the desired product. Alternatively, purification of can also be achieved by flash chromatography on silica gel. *Note*: The amount of Amberlyst might be different depending on its water content. If complete deprotection is not achieved, extra 40%–50% w/w should be added.

*2,6‐bis((E)‐3,4‐bis((tetrahydroxy)benzylidene)‐4‐((1‐(2‐ethylcarboranyl)‐1H‐1,2,3‐triazol‐4‐yl)methoxy)cyclohexan‐1‐one (* ***9*** *)*: According to the general procedure, compound **8** (0.47 g, 0.5 mmol) was treated with Amberlyst 15 (0.047 mg, 10% w/w) in 15 mL of acetone. The crude was purified on silica gel using dichloromethane/methanol 93:7 as the eluent, affording the final product **9** as a bright orange powder (0.25g, 0.42 mmol, 83% yield). Appearance: bright orange solid. r.f.: 0.28 (dichloromethane/methanol 93:7). UV active, grey spot with PdCl_2_ staining solution. Melting point: 103°C decomposition. ^1^H NMR (600 MHz, acetone‐*d6*) δ: 8.21 (bs, 4H, 4 x Ar‐O*H*), 7.78 (s, 1H, triazole Ar‐*H*), 7.49 (s, 2H, 2 x C═C*H*), 6.96 (s, 2H, 2 x Ar‐*H*), 6.83 (d, *J* = 8.2 Hz, 2H, 2 x Ar‐*H*), 6.78–6.77 (d, *J* = 8.2 Hz, 2H, 2 x Ar‐*H*), 4.66 (bs, 1H, carborane C*H*), 4.45‐4.43 (m, 2H, N‐C*H*_2_), 4.41 (s, 2H, O─C*H_2_*), 3.88‐3.83 (m, 1H, O─C*H*), 3.11‐2.93 (m, 4H, C*H_2─_* CH─C*H_2_*), 2.92–2.84 (m, 2H, N─CH_2─_C*H_2_*), 2.56‐1.41 (bs, 10, 10 x BH). ^13^C{^1^H} NMR (150 MHz, acetone‐*d6*) δ: 188.3 (Cq), 147.2 (Cq), 145.8 (Cq), 138.4 (2 x CH), 131.3 (Cq), 128.9 (Cq), 124.6 (CH), 124.6 (2 x CH), 118.1 (2 x CH), 116.3 (CH), 73.6 (Cq), 72.6 (CH), 63.7 (CH), 62.0 (CH_2_), 49.0 (CH_2_), 37.4 (CH_2_), 34.1 (2 x CH_2_). ^11^B NMR (192.5 Hz, acetone‐*d6*) δ: ‐3.67 (bs, 1B), ‐6.51 (bs, 1B), ‐10.46 (bs, 2B), ‐12.67 (bs, 4B), ‐13.90 (bs, 2B). HRMS (ESI) *m/z*: Calcd for C_27_H_35_B_10_N_3_O_6_ 608.3530 [M+H]^+^, found 608.3530 [M+H]^+^. IR *ν*_max_ (neat)/ cm^−1^: 3100 (bs, OH), 2578 (BH), 1584 (C═O), 1509, 1232, 996.

^10^B‐enriched 2,6‐bis((E)‐3,4‐bis((tetrahydroxy)benzylidene)‐4‐((1‐(2‐ethylcarboranyl)‐1H‐1,2,3‐triazol‐4‐yl)methoxy)cyclohexan‐1‐one (**^10^B‐9**): According to the general procedure, **^10^B‐8** (0.47 g, 0.5 mmol) was treated with Amberlyst 15 (0.047 g, 10% w/w) in 15 mL of acetone. The crude was purified on silica gel using dichloromethane/methanol 93:7 as the eluent, affording the final product ^10^B‐**9** as a bright orange powder (0.24 g, 0.41 mmol, 81% yield). Appearance: bright orange solid. r.f.: 0.28 (dichloromethane/methanol 93:7). UV active, grey spot with PdCl_2_ staining solution. Melting point: 100°C decomposition. ^1^H NMR (400 MHz, acetone‐d6) δ: 8.31 (bs, 2H, 2 x Ar‐OH), 8.17 (bs, 2H, 2 x Ar‐OH), 7.85 (s, 1H, triazole Ar‐H), 7.62 (s, 2H, 2 x C═CH), 7.09 (s, 2H, 2 x Ar‐H), 6.95 (d, *J* = 8.2 Hz, 2H, 2 x Ar‐H), 6.91 (d, *J* = 8.2 Hz, 2H, 2 x Ar‐H), 4.78 (bs, 1H, carborane CH), 4.57–4.54 (m, 2H, N‐CH_2_), 4.53 (s, 2H, O‐CH_2_), 3.99‐3.96 (m, 1H, O‐CH), 3.18‐3.07 (m, 4H, CH_2_‐CH‐CH_2_), 3.03‐2.99 (m, 2H, N‐CH_2_‐CH_2_), 2.56‐1.41 (bs, 10, 10 x BH). ^13^C{^1^H} NMR (100 MHz, acetone‐d6) δ: 188.3 (Cq), 147.2 (Cq), 145.9 (Cq), 138.4 (2 x CH), 131.4 (Cq), 128.9 (Cq), 124.5 (CH), 124.3 (2 x CH), 118.1 (2 x CH), 116.3 (CH), 73.7 (Cq), 72.6 (CH), 63.7 (CH), 62.1 (CH_2_), 48.9 (CH_2_), 37.4 (CH_2_), 34.1 (2 x CH_2_). HRMS (ESI) m/z: Calcd for C_27_H_35_^10^B_10_N_3_O_6_ 598.3892 [M+H]^+^, found 598.3894 [M+H]^+^. IR ν_max_ (neat)/ cm^−1^: 3105 (bs, OH), 2583 (BH), 1581 (C═O), 1500, 1242.

2,6‐bis((E)‐3‐methoxy‐4‐(tetrahydroxy)benzylidene)‐4‐((1‐(2‐ethylcarboranyl)‐1H‐1,2,3‐triazol‐4‐yl)methoxy)cyclohexan‐1‐on (**9a**): According to the general procedure, **8a** (0.057 g, 0.071 mmol) was treated with Amberlyst 15 (0.0057 g, 10% w/w) in 2.5 mL of acetone. The crude was purified on silica gel using dichloromethane/methanol 99:1 as the eluent, affording the final product **9a** as a yellow powder (0.040 g, 0.063 mmol, 88% yield). Appearance: yellow solid. r.f.: 0.32 (dichloromethane/methanol 98:2). UV active, grey spot with PdCl_2_ staining solution. Melting point: 118‐119°C. ^1^H NMR (400 MHz, acetone‐d6) δ: 8.05 (bs, 2H, 2 x Ar‐OH), 7.82 (s, 1H, triazole Ar‐H), 7.69 (s, 2H, 2 x C═CH), 7.15 (s, 2H, 2 x Ar‐H), 7.09 (dd, *J* = 8.2 Hz, 2.0 Hz, 2H, 2 x Ar‐H) 6.92 (*d*, *J* = 8.2 Hz, 2H, 2 x Ar‐H), 4.77 (bs, 1H, carborane CH), 4.56‐4.48 (m, 2H, N‐CH_2_), 4.50 (s, 2H, O‐CH_2_), 4.05–3.98 (m, 1H, O‐CH), 3.92 (s, 6H, OCH_3_), 3.24‐3.12 (m, 4H, CH_2_‐CH‐CH_2_), 3.03‐2.95 (m, 2H, N‐CH_2_‐CH_2_), 2.75‐1.50 (bs, 10, 10 x BH).^13^C{^1^H} NMR (100 MHz, acetone‐d6) δ: 188.4 (Cq), 148.6 (Cq), 148.3 (Cq), 145.9 (Cq), 138.5 (CH), 131.6 (Cq), 128.7 (Cq), 125.1 (CH), 124.3 (CH), 116.0 (CH), 115.0 (CH), 73.7 (Cq), 72.4 (CH), 63.7 (CH), 62.1 (CH_2_), 56.4 (CH_3_), 48.8 (CH_2_), 37.5 (CH_2_), 34.0 (CH_2_). ^11^B NMR (192.5 Hz, acetone‐d6) δ: −3.69 (bs, 1B), −6.55 (bs, 1B), ‐10.50 (bs, 2B), −12.75 (bs, 4B), ‐13.99 (bs, 2B). HRMS (ESI) m/z: Calcd for C_29_H_39_B_10_N_3_O_6_ 636.3842 [M+H]^+^, found 636.3843 [M+H]^+^. IR ν_max_ (neat)/ cm^−1^: 3049 (bs, OH), 2923, 2850, 2585 (BH), 1584 (C═O), 1511, 1425, 1253, 1211, 1150, 1125, 1032, 735.

*2,6‐bis((E)‐3,4‐dihydroxybenzylidene)‐4‐(prop‐2‐yn‐1‐yloxy)cyclohexan‐1‐one (* ***7b*** *)*: According to the general procedure, 2,6‐bis((*E*)‐3,4‐bis((tetrahydro‐2*H*‐pyran‐2‐yl)oxy)benzylidene)‐4‐(prop‐2‐yn‐1‐yloxy) cyclohexan‐1‐one **7** (0.030 g, 0.041 mmol) was treated with Amberlyst 15 (0.0030 g, 10% w/w) in 1.5 mL of acetone. The crude was purified on silica gel using dichloromethane/methanol 95:5 as the eluent, affording **7b** as an ocher to olive green powder (0.013 mg, 0.034 mmol, 82% yield). Appearance: ocher solid. r.f.: 0.28 (dichloromethane/methanol 95:5). UV active, blue spot with cerium‐molybdate staining solution. Melting point: 195‐196°C decomposition. ^1^H NMR (400 MHz, acetone‐d6) δ: 8.29 (bs, 1H, Ar‐OH), 8.16 (bs, 1H, Ar‐OH), 7.64 (s, 2H, 2 x C═CH), 7.09 (d, J = 2.1 Hz, 2H, 2 x Ar‐H), 6.97 (dd, J = 8.2 Hz, 2.0 Hz, 2H, 2 x Ar‐H) 6.91 (d, J = 8.2 Hz, 2H, 2 x Ar‐H), 4.11 (d, J = 2.4 Hz, 2H, CH_2_‐C≡CH), 4.09‐4.02 (m, 1H, O‐CH), 3.20 (dt, *J* = 15.7 Hz, 3.0 Hz, 2H, 2 x CH‐CH^a^H^b^), 3.09 (dd, *J* = 16.5 Hz, 6.8 Hz, 2H, 2 x CH‐CH^a^H^b^), 2.83 (t, J = 2.5 Hz 1H, C≡CH). ^13^C{^1^H} NMR (100 MHz, acetone‐d6) δ: 188.2 (Cq), 147.3 (Cq), 145.8 (Cq), 138.6 (CH), 131.2 (Cq), 128.9 (Cq), 124.5 (CH), 118.1 (CH), 116.3 (CH), 80.9 (Cq), 75.7 (Cq), 72.1 (CH), 55.8 (CH_2_), 34.0 (CH_2_). HRMS (ESI) *m/z*: Calcd for C_23_H_20_O_6_ 393.1333 [M+H]^+^, found 393.1333 [M+H]^+^. IR ν_max_ (neat)/ cm^−1^: 3287 (bs, OH), 2921, 2852, 1697, 1586 (C═O), 1519, 1440, 1368, 1279, 1238, 1196, 1154, 1115, 1078, 1001, 926, 819.

### 4.4 In Vitro Applications

#### 4.4.1 General Procedure for the Preparation of Amyloid‐Beta (Aβ) Aggregates

Aβ (1–42) was aliquoted in HFIP (Hexafluoro‐2‐propanol), evaporated overnight at room temperature, and stored at −80°C. For the experiments, aliquots were solubilized in a CH_3_CN/Na_2_CO_3_ (300 µm)/NaOH (250 mm) (48.3%/48.3%/3.4% v/v/v) mixture, sonicated and diluted in 10 mm phosphate buffer containing 11 mm NaCl (pH 7.4) to a final concentration of 50 µm [92]. Samples were then incubated at 37°C with stirring (400 or 600 rpm) for 4 days to obtain Aβ aggregates in protofibrillar and fibrillar states. Aggregation kinetics were monitored using thioflavin T (ThT) assay: Aβ (300 nm) and ThT (50 nm) were incubated for 15 min in 2 mL of 10 mm phosphate buffer containing 11 mm NaCl (pH 7.4), and fluorescence emission at 476 nm (*λ*_ex._ 450 nm) was recorded.

#### 4.4.2 Field Emission Scanning Electron Microscopy (FESEM) Analysis of Aβ Aggregates

Each sample of 50 µL was diluted to 2 mL with distilled H_2_O to remove the excess salts that could interfere with the analysis. The diluted samples were centrifuged at 10 300×g for 20 min, and then the supernatant was discarded, leaving 100 µL of sample. The pellet was resuspended, and 10 µL of this solution was spotted over a gold‐coated glass coverslip for semiquantitative analysis or onto a chromium‐coated glass coverslip for morphological studies. Chromium coatings form a continuous, fine‐grained surface that enables high‐magnification FESEM imaging of biological samples with minimal interference. However, chromium is prone to oxidation, and samples must therefore be viewed shortly after preparation, making it less suitable for prolonged semiquantitative analyses. In contrast, gold coatings are chemically stable and resistant to oxidation, making them more suited for extended analyses, although their larger grain size limits imaging at high magnification [133]. Acquired images were analyzed using ImageJ software (version 1.53t). After opening the 16‐bit image files, the black‐and‐white values were inverted to visualize Aβ fibrils in white against a black background. A manual threshold was applied to segment fibrils from the background. The selected area was analyzed to extract three parameters: (1) area (square pixels), (2) mean gray value (sum of gray values of all pixels in the selection divided by the number of pixels), and (3) integrated density (area × mean gray value). To obtain a semiquantitative evaluation of fibril number, 15 images were analyzed for each experimental condition.

#### 4.4.3 Thioflavin‐T (ThT) Fluorescence Binding and Competition Assay

For both assays, stock solutions were prepared in 99% (v/v) ethanol: ThT (450 µm) and compounds **7b**, **9** and **9a** (2.5 mm). The concentration of ThT was confirmed spectrophotometrically using its molar extinction coefficient in ethanol at 416 nm (26 600 M^−1^cm^−1^) [134].

*ThT binding assay*: A fixed concentration of ThT reporter ligand (5 nm) and varying concentrations of Aβ (1–42) fibrils (0‐4.7 µm) were incubated at 37°C for 15 min in 10 mm phosphate buffer containing 11 mm NaCl (pH 7.4) and 0.5% ethanol in a final volume of 2 mL. Fluorescence emission was recorded at 476 nm (*λ*_ex._ 450 nm, excitation and emission slit = 5 nm). From the plot of fluorescence intensity at 476 nm, fitted using Equation 1 [100], the thermodynamic dissociation constant (*K*_d_) for ThT was determined as 7.2×10^−6^ M, assuming a 1:1 interaction with Aβ (1–42) fibrils.

$$\begin{array}{ccc} y & = & \frac{\left(K_{a}C_{t}+nxK_{a}+1\right)-\sqrt{{\left(K_{a}C_{t}+nxK_{a}+1\right)}^{2}-4{K_{a}}^{2}C_{t}nx}}{2K_{a}}\\ & & \times \;\left(F_{b}-F_{f}\right)+F_{f}\times C_{t}+F_{pr}x+BKG \end{array}$$

**Equation 1**: K_a_ = association constant, *x* = Aβ concentration, *C*_t_ = ThT fixed concentration, *n* = number of binding sites, *F*_b_ = molar fluorescence intensity observed at the peak of the emission spectra when Tht is bound to fibrils, F_f_ = molar fluorescence intensity observed at the peak of the emission spectra when Tht is free, *F*_pr_ = molar fluorescence emission observed at the peak of the emission spectra for Aβ aggregates, BKG = background fluorescence (buffer plus 0.5% ethanol).

*Competition assay*: The assay was performed using a fixed concentration of Aβ aggregates (200 nM) and either ThT or curcumin (100 nm), while varying the concentration of competitor ligands (10–572 nm). Incubations were carried out in 10 mM phosphate buffer containing 11 mm NaCl (pH 7.4) and 0.25% ethanol, in a final volume of 1995 µL for 3 h or 15 min at 37°C with stirring at 400 rpm. Subsequently, 5 µL of ThT or curcumin (40 µm in ethanol) was added and incubated, and the mixture was further incubated 15 min at 37°C under stirring. Fluorescence emission spectra were acquired by exciting samples at 450 nm and recording the emission spectra from 460 to 800 nm (slit width = 5 nm). Data was analyzed with OriginLab software using the DoseResp fitting function to determine IC_50_ values. Finally, inhibition constants (*K*_i_) were calculated using the Cheng‐Prusoff equation [99].

#### 4.4.4 Neutron Irradiation on Aβ Aggregates

Aβ (50 µm) samples were incubated for 4 days at 37°C with stirring at 600 rpm to obtain mature fibrils. Compound **^10^B‐9** was then added at 1:1 ratio, reaching a final concentration of 5% (v/v) ethanol, and incubation was continued overnight at 37°C with stirring at 400 rpm to allow binding. Non‐irradiated Aβ samples, incubated under the same conditions with or without **^10^B‐9**, were used as controls. Two reactor powers (250 and 30 kW) and two irradiation times (1 h and 15 min) were tested. The 1 h irradiation at 250 kW was selected as the reference condition and repeated in seven separate irradiation sessions to assess reproducibility. Disaggregation of irradiated and non‐irradiated samples, including Aβ controls, was analyzed using Field Emission Scanning Electron Microscopy (FESEM). Additionally, as a further control, both irradiated and non‐irradiated Aβ samples were analyzed using the ThT assay, as described in the Aβ aggregate preparation section, but without the addition of **^10^B‐9**.

#### 4.4.5 ^1^H‐Nuclear Magnetic Resonance (NMR) Analysis of Aβ Aggregates

Aβ aggregates were prepared as stated in Section 4.4.1. (90 µg, 50 µm). Irradiated and non‐irradiated samples were prepared according to Section 4.4. Oxidized Aβ aggregates were obtained by incubating 4‐day aggregated samples with 150 mm hydrogen peroxide (0.5% v/v) for 2 h or overnight at 37°C under stirring. All the samples were lyophilized overnight prior to NMR analysis.

*1D NMR experiments*: Lyophilized samples were solubilized with slight modifications to the protocol of Pilkington et al., [106] in 60 mm NaOD (final Aβ concentration: 100 µm), allowed to stand for 10 min for disaggregation, and then diluted to 40 µm with D_2_O. A 500 µL aliquot was placed in a 5 mm NMR tube (Wilmad WG‐1000–7600 Mhz, purchased from Sigma), and the ^1^H NMR spectra were acquired using a 1D‐NOESY sequence at 25°C, D1 relaxation delay time 11 s, acquisition time 3.4 s, SW = 15.78 ppm and NS = 256.

*Diffusion NMR experiments*: NMR experiments were performed at 25°C on a Bruker NEO equipped with a Prodigy cryoprobe operating at 600 MHz. 2D‐DOSY spectra were acquired with a stimulated echo pulse sequence, and matrices of 2048 (t2) by 80 points (t1) were collected. The *z*‐axis gradient strength varied linearly from 2% to 98% of its maximum value (65.7 G cm^−1^), the gradient pulse duration was 4.0 ms, and the time period between the two gradient pulses was optimized to 80 ms for Aβ NMR visible species. The relaxation delay D1 was set to 1 s. Water suppression was achieved using a 3‐9‐19 pulse sequence with gradients. All spectra were manually phased and baseline corrected using TOPSPIN 4.3.0 (Bruker, Karlsruhe, Germany). Diffusion data were obtained through the Dynamic Centre tool implemented in Topspin 4 software and processed by using the inverse Laplace transform (ILT) routine of the Bruker Dynamics Center software suite, version 2.6.1. Starting from the measured diffusion (D) value an “apparent MW” of the small oligomers can be calculated by the combination of Stokes−Einstein equation with the relationship between molar radius and formula weight [135].

*Quantitative NMR*: Samples were analyzed using the ERETIC 2 (Electronic Reference To access In vivo Concentrations) methodology [136]. ERETIC 2 is an experimental quantitative NMR technique based on PULCON (pulse length based concentration determination) [137], an internal standard method which correlates absolute intensities of two different spectra. In 1D NMR experiments, a 3‐(Trimethylsilyl)‐1‐propanesulfonic acid‐d6 sodium salt (63.4 µm) was used as reference. Spectra acquiring at 25°C, D1 relaxation delay time 11 s, acquisition time 3.4 s, SW = 15.78 ppm, and NS = 256. In diffusion NMR experiments a sucrose sample of known concentration (29.2 mM), under “quantitative” conditions, was employed as reference: probe was exactly tuned and matched, 90° pulse calibrated, D1 relaxation delay time 14 s, acquisition time 5 s, SW = 29.7 ppm, and NS = 256 were employed.

#### 4.4.6 NanoHPLC‐HRMS Settings

The histidine oxidation was confirmed by nanoHPLC‐HRMS (nanoHigh Performance Liquid Chromatography‐High Resolution Mass Spectrometry). A dedicated sample preparation protocol was established before mass spectrometry analysis. Because the study focused on amino acid oxidation and the protein doesn't have cysteine residues, reduction and alkylation steps were intentionally omitted before the tryptic digestion of the Aβ (1‐42) protein. For each sample, trypsin was added at an enzyme‐to‐protein ratio of 1:50, corresponding to 80 µg of total protein. Aβ was first solubilized in 50 mm ammonium bicarbonate and subjected to sonication in an ultrasonic bath for 30 min to disrupt fibrillar aggregates. The sample was then incubated with trypsin (100 ppm) overnight at 37°C. Digestion was stopped by acidification, and a subsequent spin tip purification step was performed following the manufacture instructions. The samples were reconstituted with 10 µL of 0.1% aqueous formic acid for nanoHPLC‐HRMS analysis. The described procedure was applied to non‐irradiated Aβ samples with **^10^B‐9** (9NI), and to irradiated Aβ samples with **^10^B‐9** exposed to a neutron fluence of 4.3 × 10^13^ cm^−2^ (9I‐Ψ_3_).

The gradient elution for chromatographic separation started from 95%–5% of 0.1% formic acid in water (solvent A) and acetonitrile:0.1% formic acid in water 8:2 (solvent B) maintained for 8 min. These first 8 min were mandatory to complete the preconcentration step on the C18 trap by using trifluoroacetic acid 0.05% in water/acetonitrile 98/2 as eluent (flow rate 5 µL min^−1^). From minute 8 to minute 50, gradient increased up to 99% of solvent B; then the column went back to the initial conditions for a total run time of 63 min. Flow rate and injection volume were 300 nL min^−1^ and 5 µL, respectively. The nano column was interfaced with a nano‐ESI source, operated under the following conditions: spray positive voltage 1900 V and ion transfer tube temperature 275°C. Full mass spectra were acquired in the range between 100 and 1500 *m/z*. For the dedicated tandem MS experiments we used quadrupole as isolation mode, with an isolation window of 1.6 *m/z*, and HCD (High Energy Collision Induced Dissociation) activation mode customized for each peptide (Table S4). The resolving power was set at 60 000 both for full mass and MS/MS spectra.

#### 4.4.7 Statistical Analysis

Statistical analyses were performed using OriginPro (OriginLab, Northampton, MA, USA). No data points were excluded from the analyses. For each experiment, values obtained from independent replicates were averaged before statistical testing. Data are presented as mean ± standard deviation (SD), unless otherwise stated. Comparisons between two independent groups were performed using a two‐tailed unpaired Student's t‐test. A *p* value < 0.05 was considered statistically significant. The statistical comparisons were pre‐specified and limited to the indicated group pairs; therefore, *p* values were reported without adjustment for multiple comparisons. Sample sizes (n) for each analysis are reported in the corresponding figure legends. Statistical significance is indicated as follows: ns: not significant (*P* ≥ 0.05); *: *p* < 0.05; **: *p* < 0.01; ***: *p* < 0.001; ****: *p* < 0.0001.

## Funding

European Union's Horizon 2020 research and innovation programme (grant agreement no. 964934). Fondazione Compagnia di San Paolo (CUP: D13C25000220007). Project CH4.0 under the MUR program “Dipartimenti di Eccellenza 2023–2027” (CUP: D13C22003520001).

## Conflicts of Interest

The authors declare no conflicts of interest.

## Supporting information




**Supporting File**: advs75256‐sup‐0001‐SuppMat.pdf

## Data Availability

The data that support the findings of this study are available from the corresponding author upon reasonable request.
