# Supplementary material for: Carboranyl‐Curcuminoids for the Neutron Capture‐Based Treatment of Amyloid Aggregates in Alzheimer's Disease
Source: Adv Sci (Weinh). 2026 Apr 16;13(39):e21701. doi: 10.1002/advs.202521701 (PMC13335006; doi:10.1002/advs.202521701)
Supplement: Supplementary file 1 — Supporting File: advs75256‐sup‐0001‐SuppMat.pdf [file ADVS-13-e21701-s001.pdf]

Supporting informations

Carboranyl-Curcuminoids for the Neutron Capture-based Treatment of Amyloid Aggregates in Alzheimer's Disease

Sebastiano Micocci†, Stefano Parisotto†, Diego Alberti, Alberto Lanfranco, Valeria Bitonto, Polyssena Renzi, Martina Salmi, Andrea Magnani, Federica Dal Bello, Katiuscia Pagano, Laura Ragona, Nicoletta Protti, Annamaria Deagostino*, and Simonetta Geninatti Crich**

Supplementary Figures and Tables

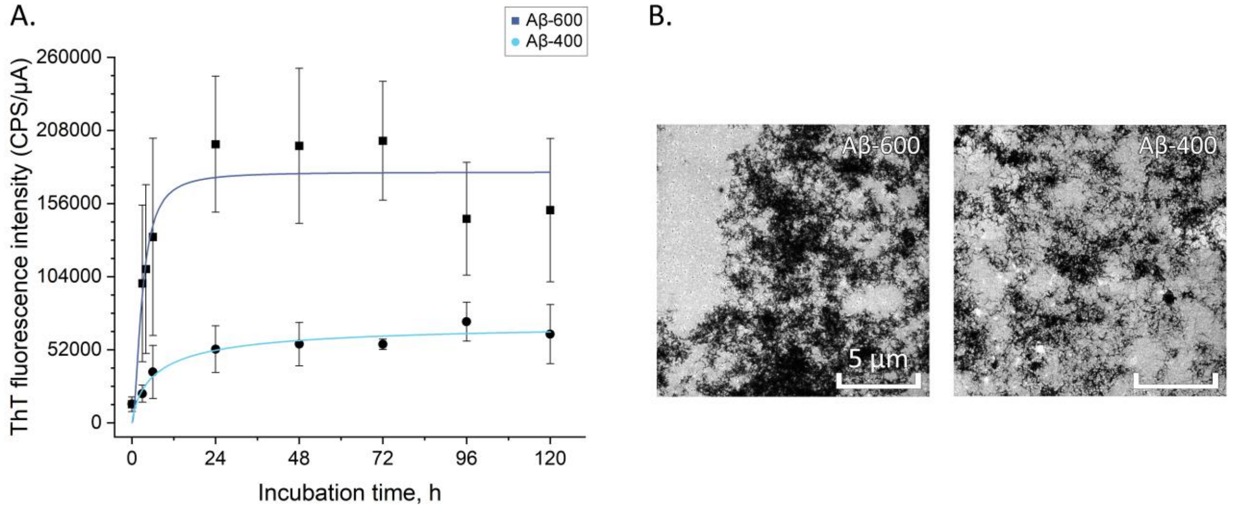

Figure S1. (A) ThT fluorescence enhancement at different times of incubation at two different stirrings,400 rpm (Aβ-400) and 600 rpm (Aβ-600) (37°C, under stirring, [Aβ] 300 nM, [ThT] 50 nM, ex. 450 nm, em. 476 nm). The curves were fitted with an exponential trend solely to illustrate the overall kinetic behavior. (B) Representative FESEM images of Aβ samples prepared with different stirring. n = 13 (Aβ-600), 4 (Aβ-400). Data are presented as mean ± SD.

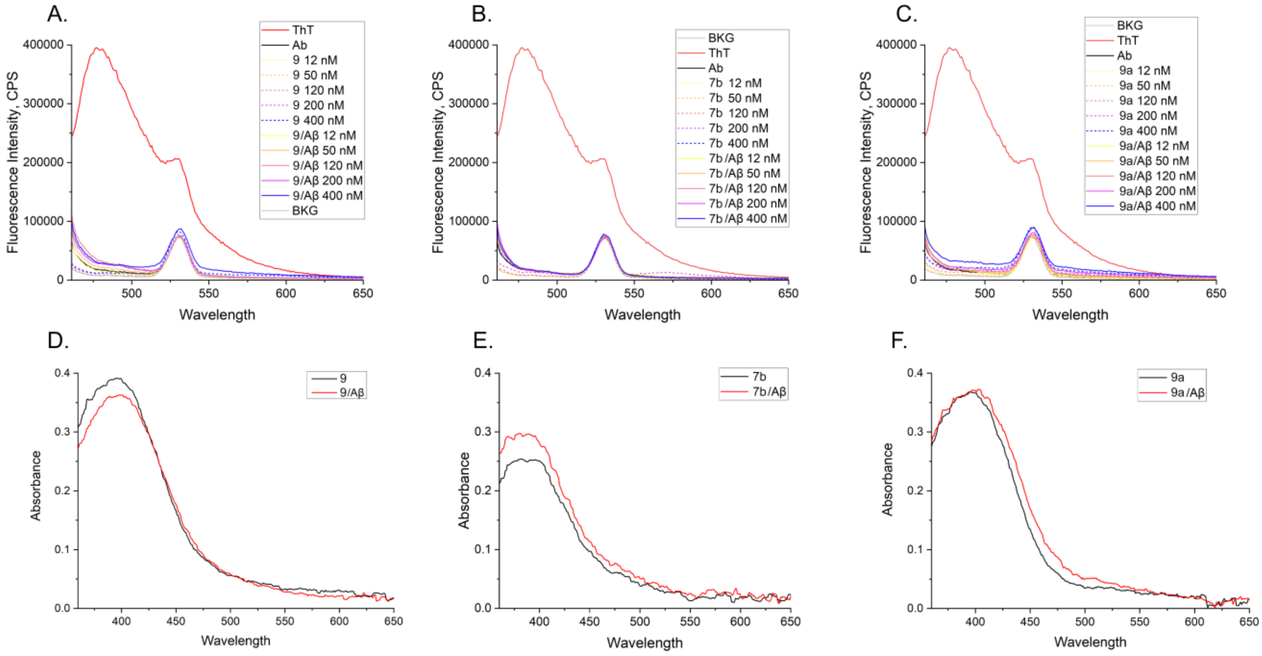

Figure S2. (A-C) Fluorescence emission ($\lambda_{ex.}$ 450 nm, $\lambda_{em.}$ 476 nm) and (D-F) UV–Vis absorption spectra of compounds **9**, **7b** and **9a**, acquired in the presence or absence of Aβ fibrils. Spectra correspond to: (A, D) **9** alone and with Aβ (**9/Aβ**); (B, E) **7b** alone and with Aβ (**7b/Aβ**); (C, F) **9a** alone and with Aβ (**9a/Aβ**). Fluorescence intensities measured at the highest concentration of each

competitor, either in the presence or absence of Aβ fibrils are < 9% respect to the Thioflavin T emission peak. n= 2 independent experiments for (A–C). Data are presented as mean ± SD.

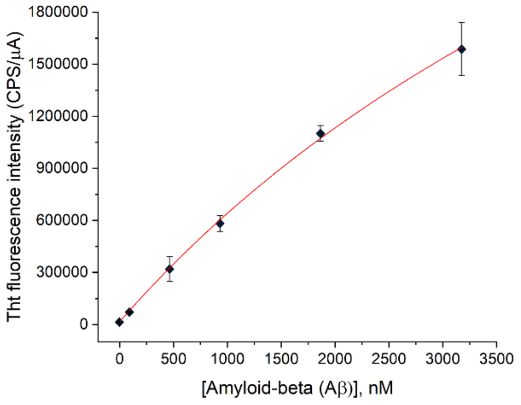

Figure S3. ThT binding Plot of ThT fluorescence emission at 476nm ($\lambda_{ex.}$ 450 nm). The red line is the result of the fitting of the experimental points by Equation 1. n = 2 independent experiments per condition. Data are presented as mean ± SD.

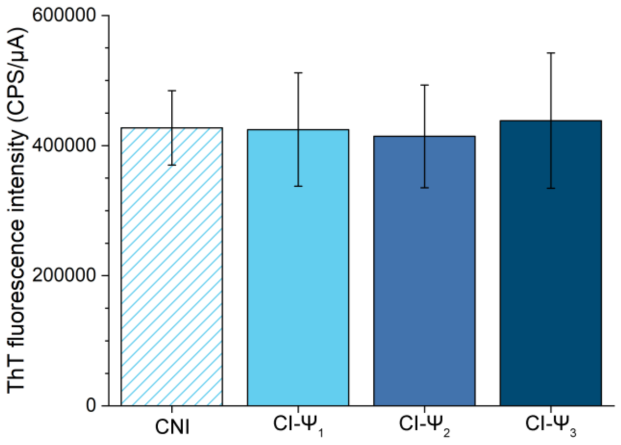

Figure S4. Fluorescence intensity of Thioflavin T (ThT) of irradiated and non-irradiated Aβ samples, in absence of the compounds ([Aβ] 300 nM, [ThT] 50 nM, $\lambda_{ex.}$ 450 nm, $\lambda_{em.}$ 476 nm). n = 4 independent experiments per condition. Data are presented as mean ± SD. Statistical comparisons between two independent groups were performed using a two-tailed unpaired Student's t-test; all comparisons were not significant (ns, $P \geq 0.05$).

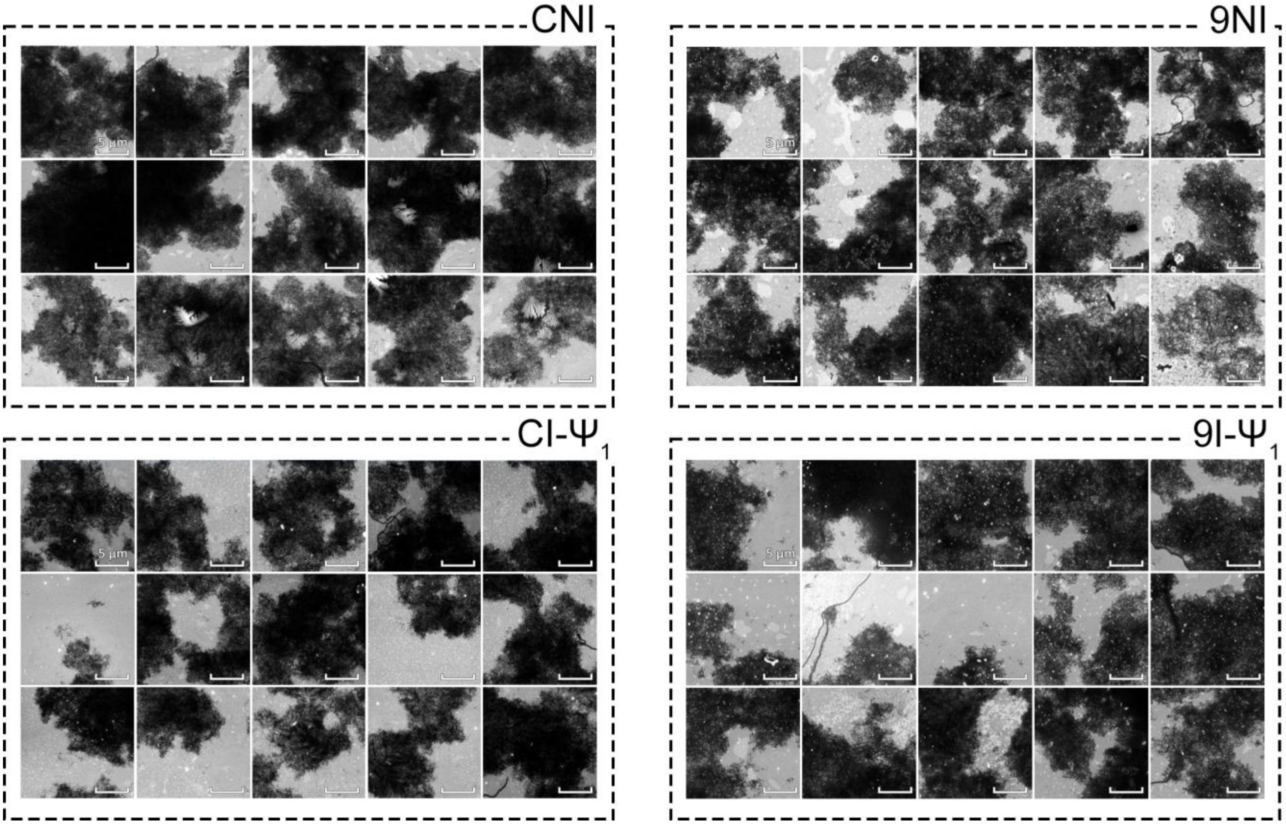

Figure S5. Example of semiquantitative analysis from one irradiation session (Ψ_1) comparing non-irradiated Aβ without (CNI) or with ^{10}**B-9** (9NI) and the respective irradiated counterparts (CI, 9I), 15 images were acquired. Scalebar in each image corresponds to 5 μm. Statistical data reported in **Table S1.**

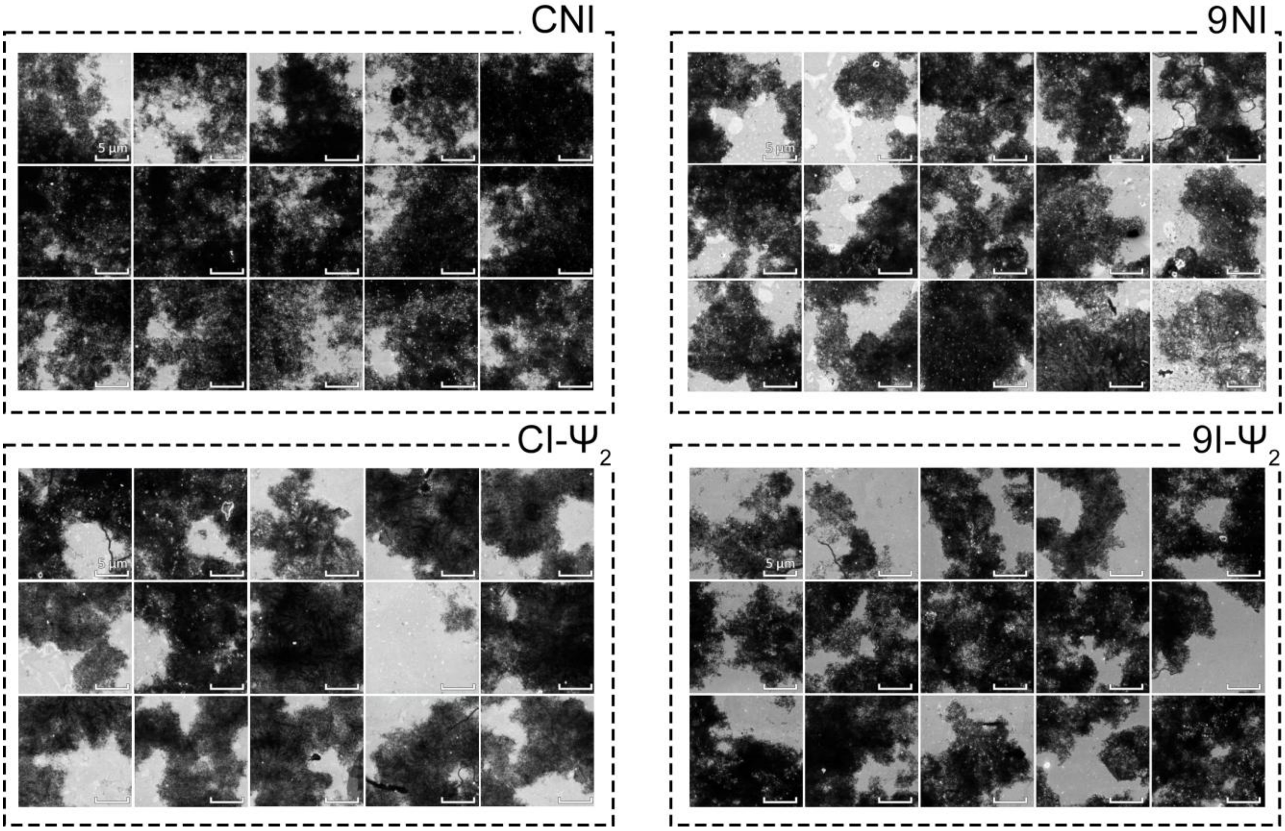

Figure S6: Example of semiquantitative analysis from one irradiation session (Ψ_2) comparing non-irradiated Aβ without (CNI) or with **^{10}B-9** (9NI) and the respective irradiated counterparts (CI, 9I), 15 images were acquired. Scalebar in each image corresponds to 5 µm. Statistical data reported in Table S1.

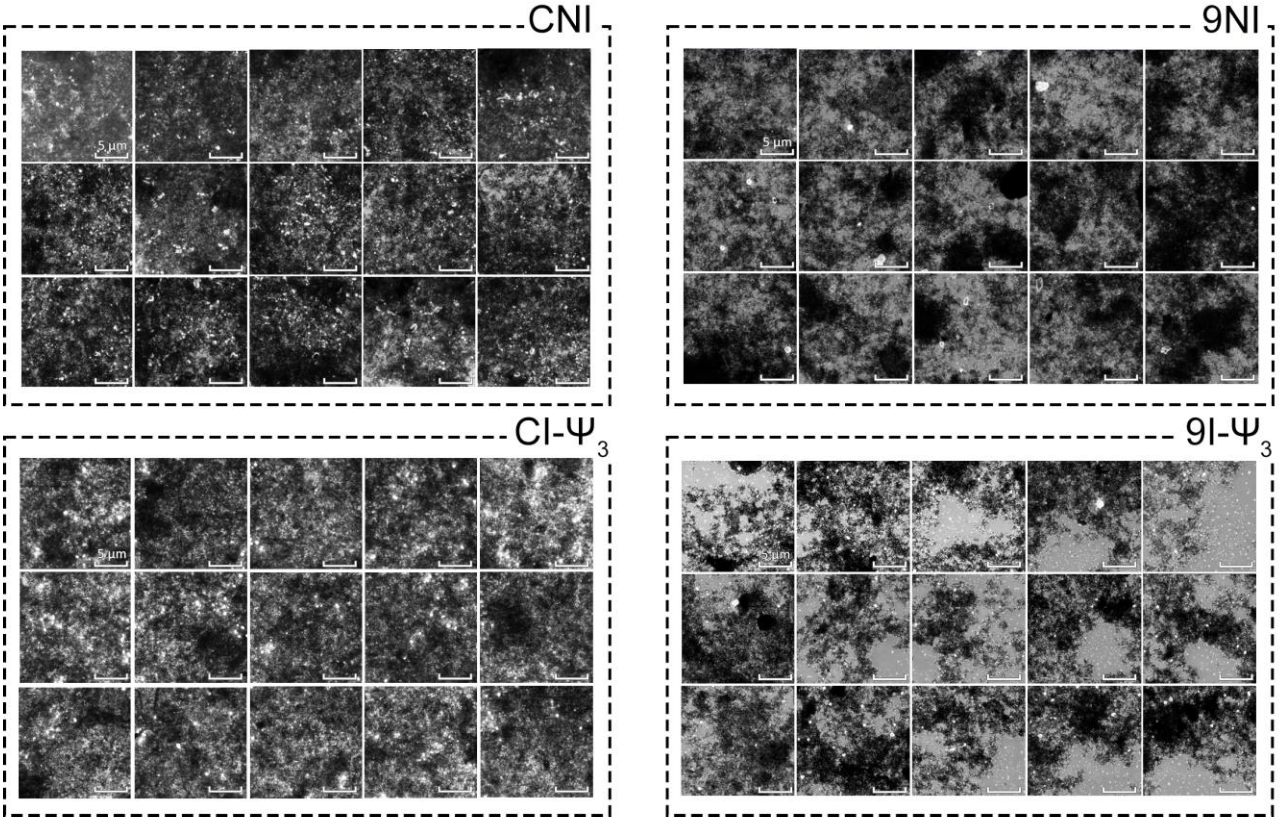

Figure S7: Example of semiquantitative analysis from one irradiation session (Ψ_3) comparing non-irradiated Aβ without (CNI) or with **[10]B-9** (9NI) and the respective irradiated counterparts (CI, 9I), 15 images were acquired. Scalebar in each image corresponds to 5 µm. Statistical data reported in Table S1.

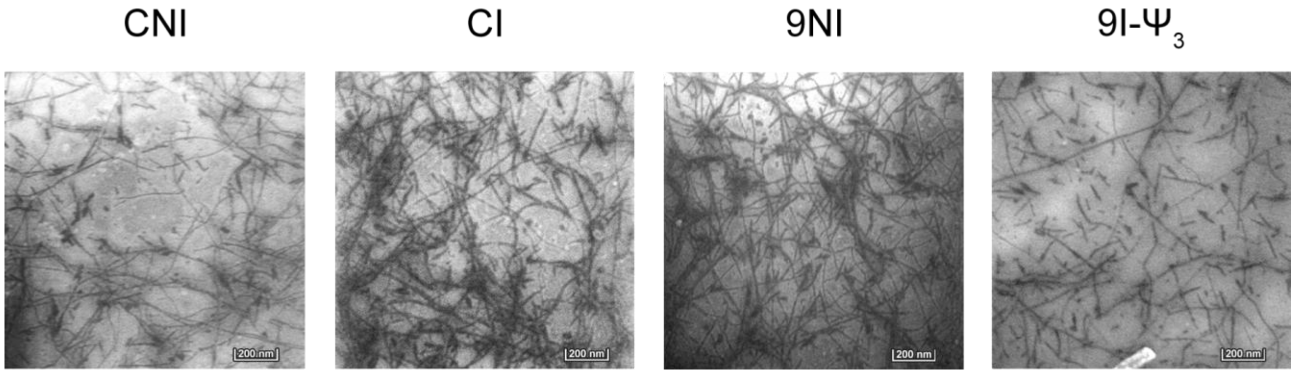

Figure S8. Representative high-resolution FESEM morphological details from one irradiation session (Ψ_3) comparing non-irradiated Aβ without (CNI) or with **[10]B-9** (9NI) and the respective irradiated counterparts (CI, 9I), acquired at 200,000× magnification. Samples were prepared with a thin chromium coating to bypass the nanometric grain artifacts of gold, allowing for an optimized morphological assessment of the fibrillar structures.

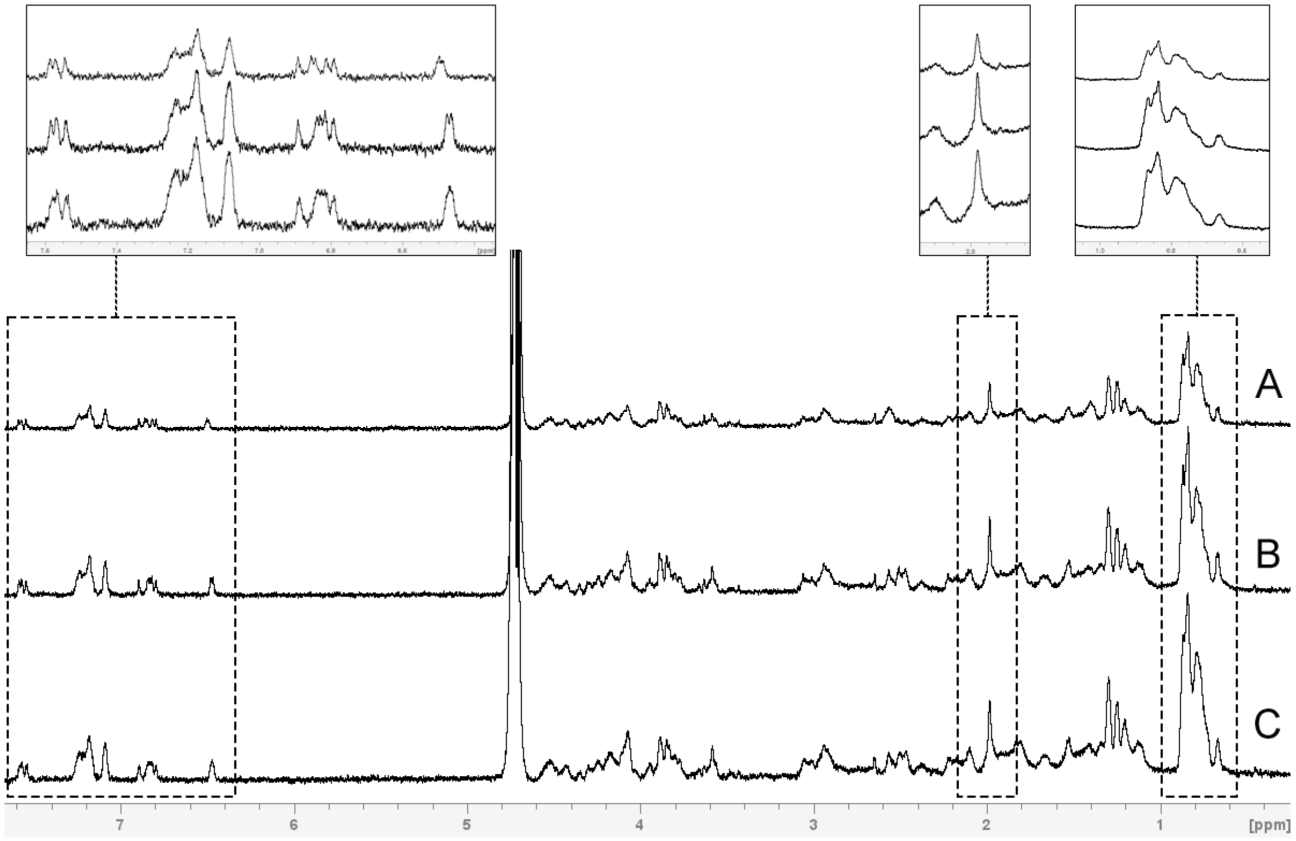

Figure S9. Representative ^{1}H-NMR spectra (1D-NOESY, 600 MHz) of Aβ fibril samples dissolved in NaOH at three different concentrations: (A)10 mM (B) 60 mM; (C) 100 mM. At this highest concentration (100mM) peaks appears broader and with lower resolution.

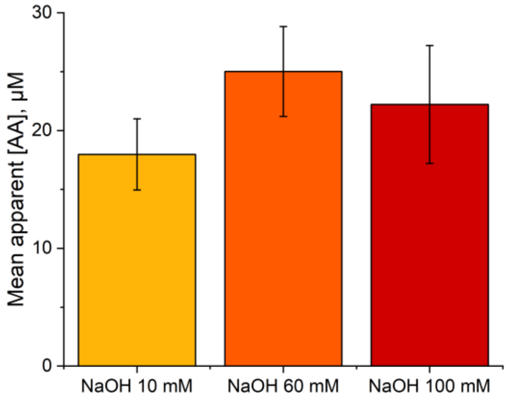

Figure S10. Apparent amino acid concentrations after Aβ fibrils dissolution in NaOH at 10, 60, and 100 mM: the 60 mM condition shows the highest mean values (+39% vs 10 mM and + 11% vs 100mM). Bars represent the mean across measured amino acid NMR signals; error bars indicate SD (n=5 amino acids).

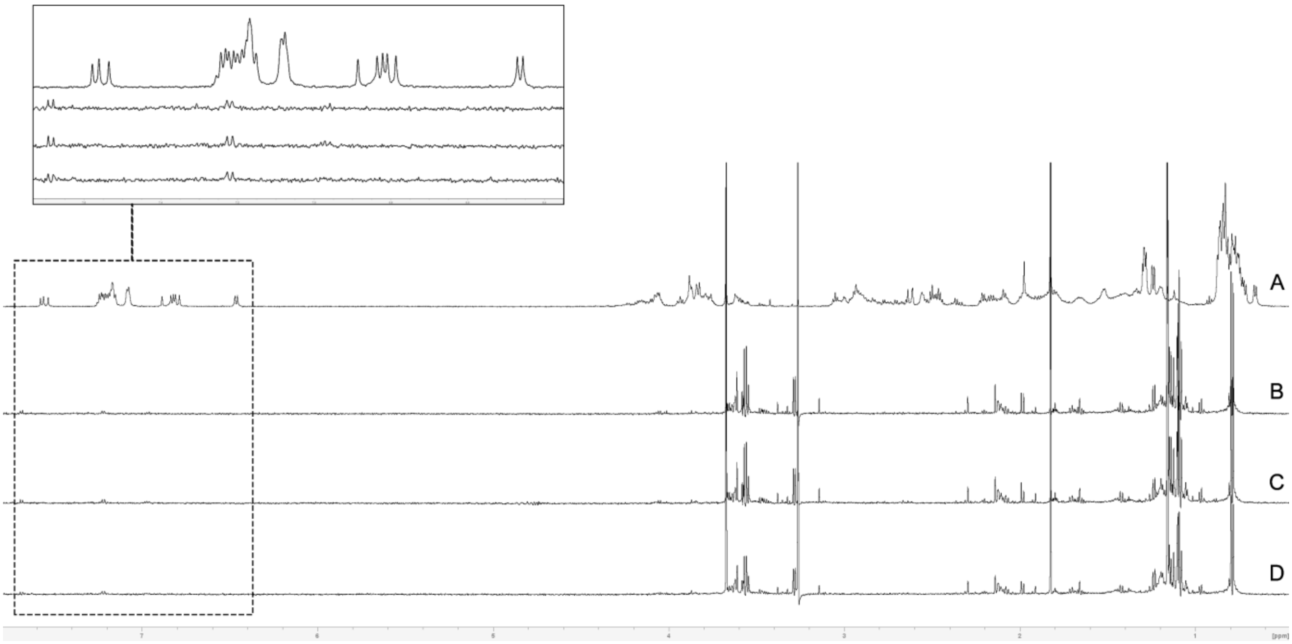

Figure S11. Representative ^{1}H-NMR spectra (1D-NOESY, 600 MHz) of Aβ fibril samples after Ψ_3 irradiation session dissolved in PBS (phosphate buffer saline, pH = 7.4). (A) non-irradiated Aβ monomers; (B) non-irradiated Aβ control (CNI); (C) non-irradiated Aβ with ^{10}**B-9** (9NI); (D) neutron-irradiated Aβ with ^{10}**B-9** (9I-Ψ_3).

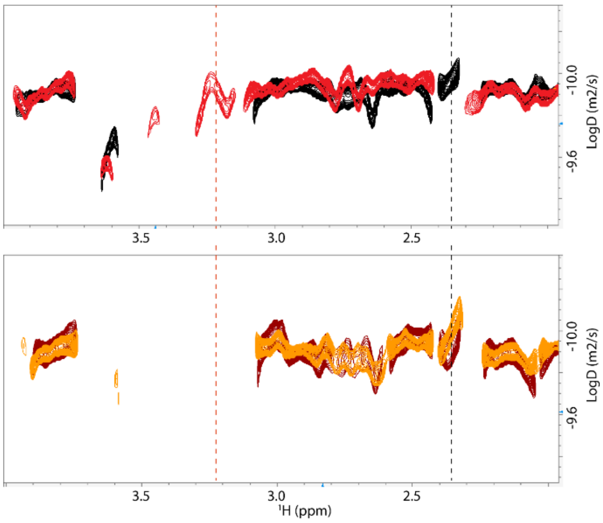

Figure S12. Overlay of the 2D-DOSY spectra of Aβ peptide (black) and Aβ+H_2O_2 (red) in upper panel; Ab+^{10}**B-9** (dark red) and irradiated Aβ+^{10}**B-9** (orange) in lower panel. Samples were acquired at 600 MHz, 25°C. The chemical shifts of CH3γ protons of reduced and oxidized M35 are highlighted by black and red dashed lines, respectively. Methionine oxidation, clearly observable upon H_2O_2 treatment, does not occur upon neutron irradiation.

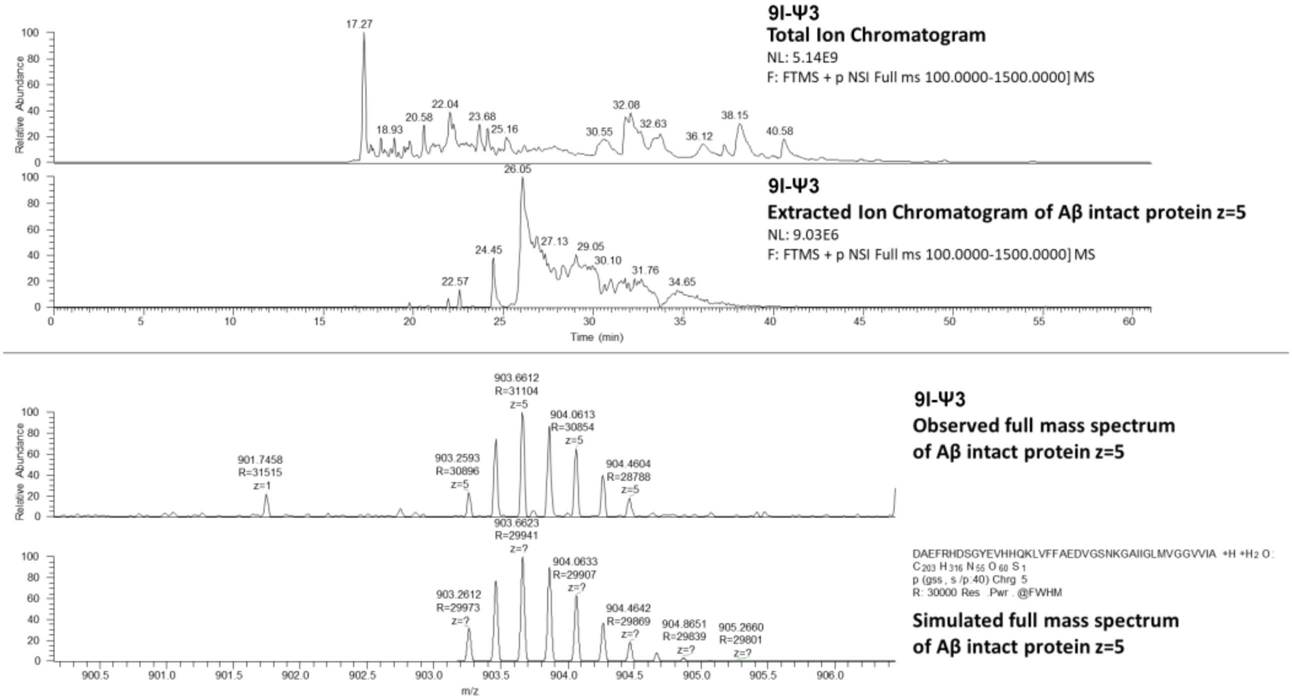

Figure S13. Total ion chromatogram of the sample 9I-Ψ_3 analyzed with the nanoHPLC-HRMS method. Intact Aβ species were still detected (*m/z* 903.6612, z=5) in the chromatographic run (R_t= 26.05-34.65 min) despite a sufficient trypsin-to-protein ratio for complete digestion. The peak shape suggests that the use of a C18 column is not appropriate for intact protein analysis due to inefficient retention mechanisms.

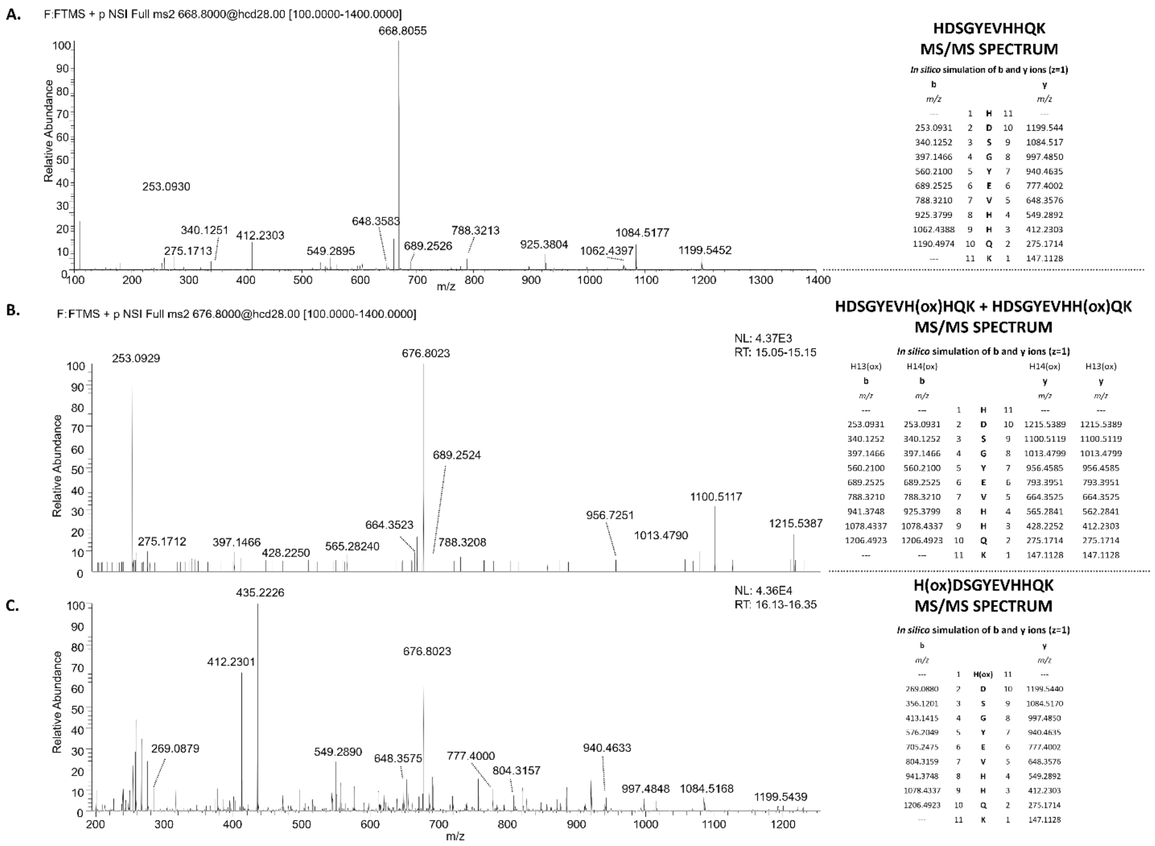

Figure S14. MS/MS spectra of: A. peptide HDSGYEVHHQK unmodified; B. peptide HDSGYEVHHQK di-oxidated on His13 and His14; C. peptide HDSGYEVHHQK mono-oxidated on His6.

Table S1: Student t-test statistical analysis (P values) performed with OriginLab software, based on results shown in Figure 3. 15 different images were acquired per sample in each irradiation session. Comparisons between two independent groups were performed using a two-tailed unpaired Student's t-test. A P value < 0.05 was considered statistically significant.

Neutron Fluence $[cm^{-2}]$	CNI vs CI[a)] (Δ%)[b)]	CNI vs 9NI[a)] (Δ%)[b)]	CI vs 9I[a)] (Δ%)[b)]	9NI vs 9I[a)] (Δ%)[b)]
Ψ_1	ns	P ≤ 0.05	P ≤ 0.01 (+13%)	ns
Ψ_2	P ≤ 0.05	P ≤ 0.05	P ≤ 0.05	P ≤ 0.05

Ψ_3	P ≤ 0.05	P ≤ 0.05	P ≤ 0.0001 (-34%)	P ≤ 0.0001 (-30%)

[a] P value; [b] Percentage decrease (Δ%)

Table S2: Student t-test statistical analysis (P values) performed with OriginLab software, based on results shown in Figure 7. Comparisons between two independent groups were performed using a two-tailed unpaired Student's t-test. A P value < 0.05 was considered statistically significant.

Neutron Fluence [cm^{-2}]	Histidine (His)			
	CNI vs CI[a] (Δ%)[b]	CNI vs 9NI[a] (Δ%)[b]	CI vs 9I[a] (Δ%)[b]	9NI vs 9I[a] (Δ%)[b]
Ψ_1	ns	P ≤ 0.001 (-31%)	P ≤ 0.0001 (-44%)	ns
Ψ_2	ns	P ≤ 0.001 (-31%)	P ≤ 0.0001 (-41%)	ns
Ψ_3	ns	P ≤ 0.001 (-31%)	P ≤ 0.0001 (-55%)	P ≤ 0.001 (-33%)
Neutron Fluence [cm^{-2}]	Phenylalanine (Phe)			
	CNI vs CI[a] (Δ%)[b]	CNI vs 9NI[a] (Δ%)[b]	CI vs 9I[a] (Δ%)[b]	9NI vs 9I[a] (Δ%)[b]
Ψ_1	ns	P ≤ 0.05	P ≤ 0.05	ns
Ψ_2	P ≤ 0.05	ns	P ≤ 0.05	ns
Ψ_3	ns	P ≤ 0.05	P ≤ 0.05	ns
	Tyrosine (Tyr)			

Neutron Fluence [cm^{-2}]	CNI vs CI[a)] (Δ%)[b)]	CNI vs 9NI[a)] (Δ%)[b)]	CI vs 9I[a)] (Δ%)[b)]	9NI vs 9I[a)] (Δ%)[b)]
Ψ_1	ns	P ≤ 0.01 (-33%)	P ≤ 0.05	ns
Ψ_2	ns	P ≤ 0.01 (-33%)	P ≤ 0.05	ns
Ψ_3	ns	P ≤ 0.01 (-33%)	P ≤ 0.05	ns

Neutron Fluence [cm^{-2}]	Methionine (Met)			
	CNI vs CI[a)] (Δ%)[b)]	CNI vs 9NI[a)] (Δ%)[b)]	CI vs 9I[a)] (Δ%)[b)]	9NI vs 9I[a)] (Δ%)[b)]
Ψ_1	ns	ns	P ≤ 0.05	ns
Ψ_2	ns	ns	P ≤ 0.05	ns
Ψ_3	ns	ns	P ≤ 0.05	ns

Neutron Fluence [cm^{-2}]	Valine-Isoleucine-Leucine (Val-Ile-Leu)			
	CNI vs CI[a)] (Δ%)[b)]	CNI vs 9NI[a)] (Δ%)[b)]	CI vs 9I[a)] (Δ%)[b)]	PNI vs 9I[a)] (Δ%)[b)]
Ψ_1	P ≤ 0.05	P ≤ 0.01 (-32%)	P ≤ 0.01 (-42%)	ns
Ψ_2	P ≤ 0.05	P ≤ 0.01 (-32%)	P ≤ 0.05	ns
Ψ_3	ns	P ≤ 0.01 (-32%)	P ≤ 0.01 (-48%)	ns

[a)] P value; [b)] Percentage decrease (Δ%)

Table S3. Student t-test statistical analysis (P values) performed with OriginLab software, based on results shown in Figure 9. Comparisons between two independent groups were performed using a two-tailed unpaired Student's t-test. A P value < 0.05 was considered statistically significant.

AA	His	Phe	Tyr	Met	Val-Ile-Leu
CNO vs CO[b] (Δ%)[c]	P ≤ 0.0001 (-51,7%)	ns	ns	P ≤ 0.01 (-58,7%)	ns

a) P value; b) Percentage decrease (Δ%)

Table S4. Amino acids sequences, exact $[M+H]^+$ masses, precursor ion *m/z* values, HCD collision energies (%) and tandem MS scan range (*m/z*) of tryptic peptides generated from Aβ. All peptides exhibited a charge state (*z*) of 2^+. Ox, oxidation; n.d., not detected.

Aminoacids sequence	Retention time Rt [min]	Exact mass[a)]	HCD collision energy [%]	MS/MS scan range[b)]
DAEFR	16.53	319.1506	23	70-750
HDSGYEVHHQK	14.54	668.8051	28	100-1400
H(ox)DSGYEVHHQK	16.26	676.8025	28	100-1400
HDSGYEVH(ox)HQK	15.10	676.8025	28	100-1400
HDSGYEVHH(ox)QK	15.10	676.8025	28	100-1400
H(ox)DSHYEVHH(ox)QK	n.d.	684.8000	28	100-1400
H(ox)DSGYEVH(ox)HQK	15.07	684.8000	28	100-1400

HDSGYEVH(ox)H(ox)QK	n.d	684.8000	28	100-1400
H(ox)DSGYEVH(ox)H(ox)QK	n.d.	692.7975	28	100-1400
LVFFAEDVGSNK	22.47	663.3404	23	100-1400
GAIIGLMVGGVVIA	29.17	635.3836	23	100-1300

[a] $[M+H]^{2+}$; [b] *m/z*

HRMS spectra

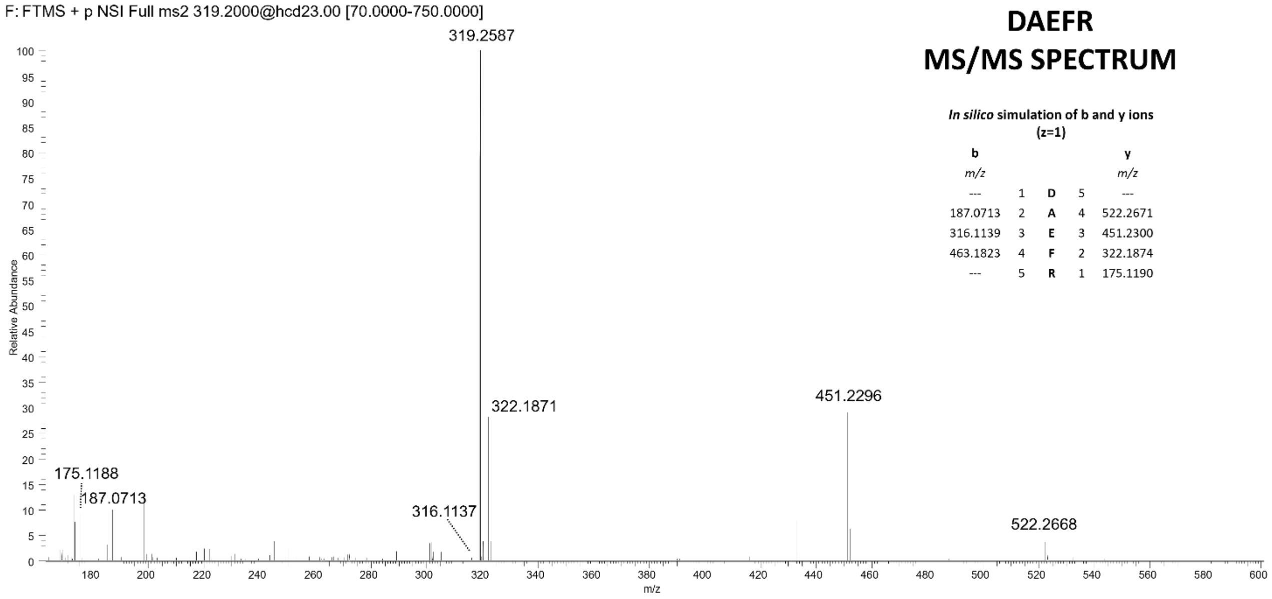

Figure S15. MS/MS spectra of peptide DAEFR *m/z* = 319.1506 (z = 2).

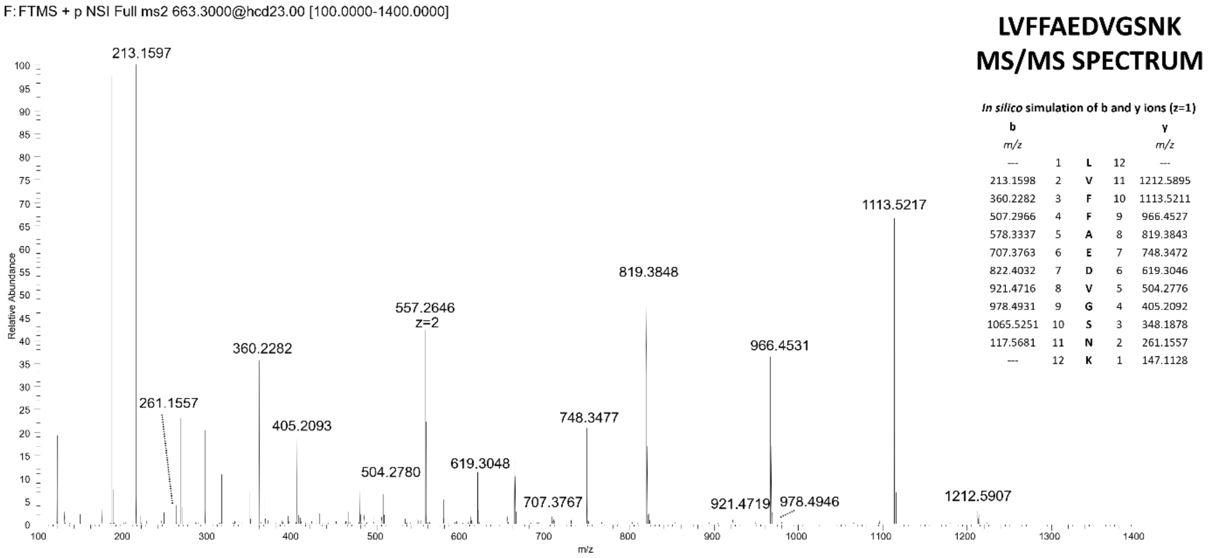

Figure S16. MS/MS spectra of peptide LVFFAEDVGSNK *m/z* = 663.3404 (z = 2).

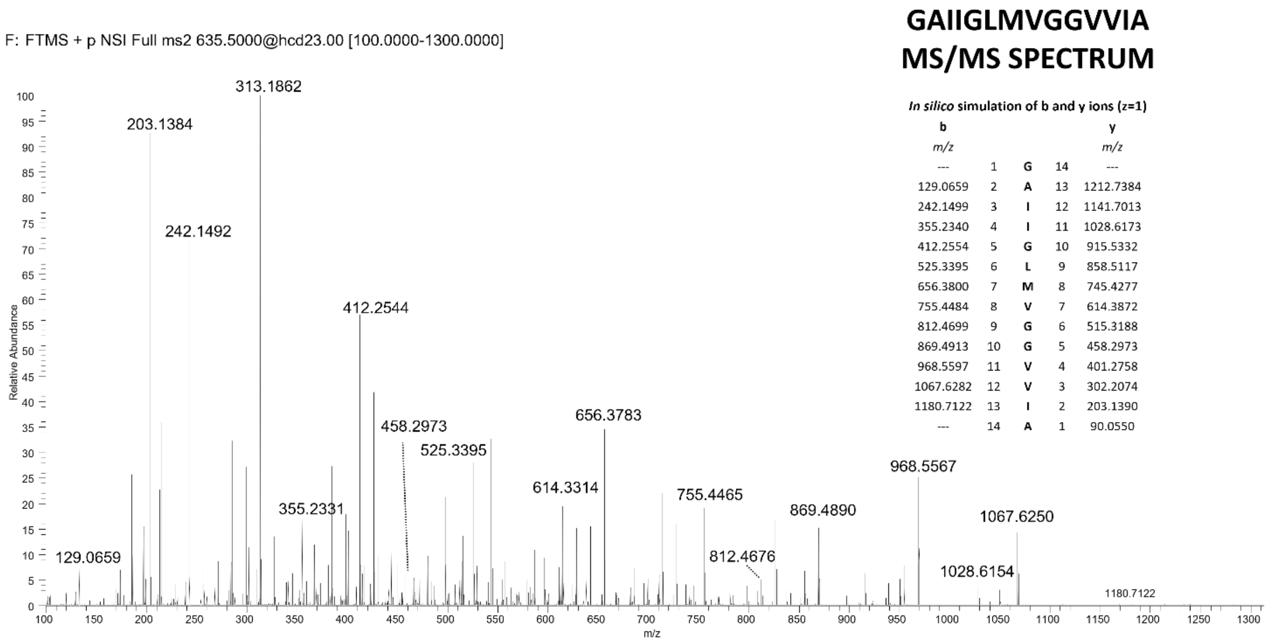

Figure S17. MS/MS spectra of peptide GAIIGLMVGGVVIA *m/z* = 663.3404 (z = 2).

NMR spectra

*2-(C-o-carboranyl)-ethyl-4-methylbenzenesulfonate (**2**)*

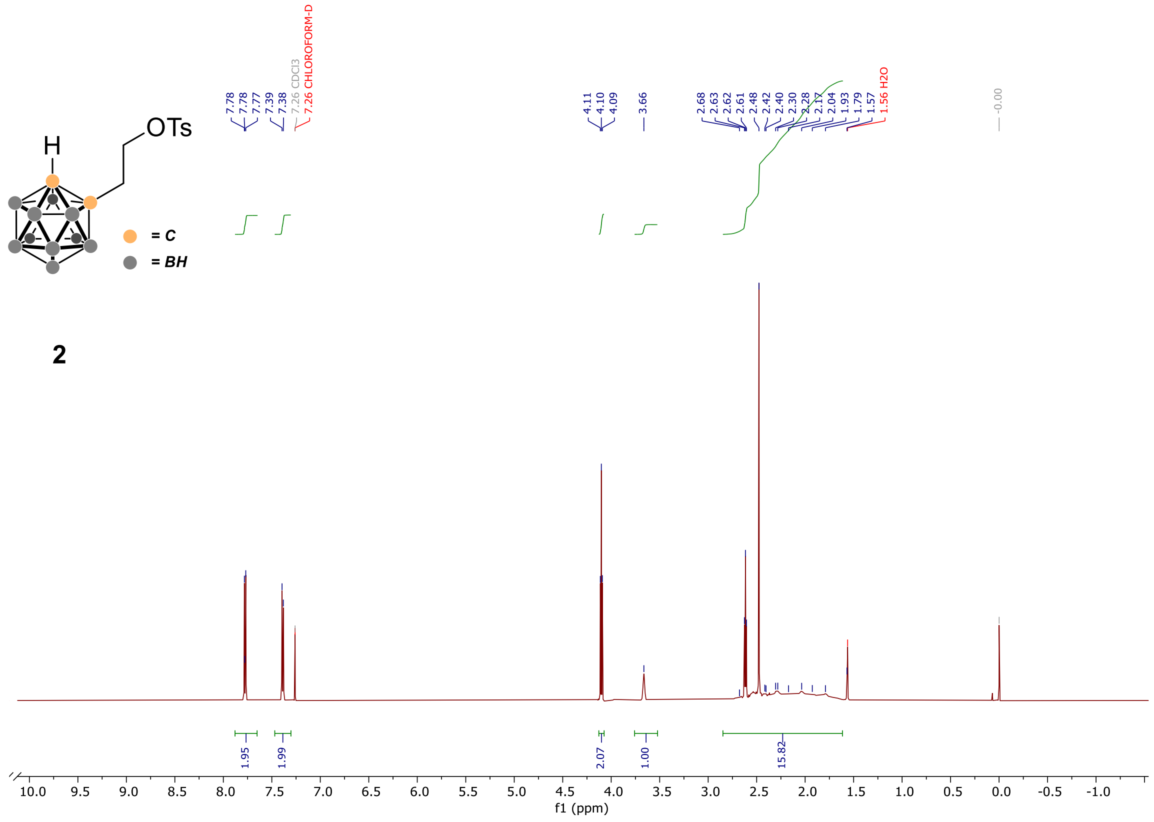

Figure S18. ^{1}H-NMR 2-(C-o-carboranyl)-ethyl-4-methylbenzenesulfonate (**2**)

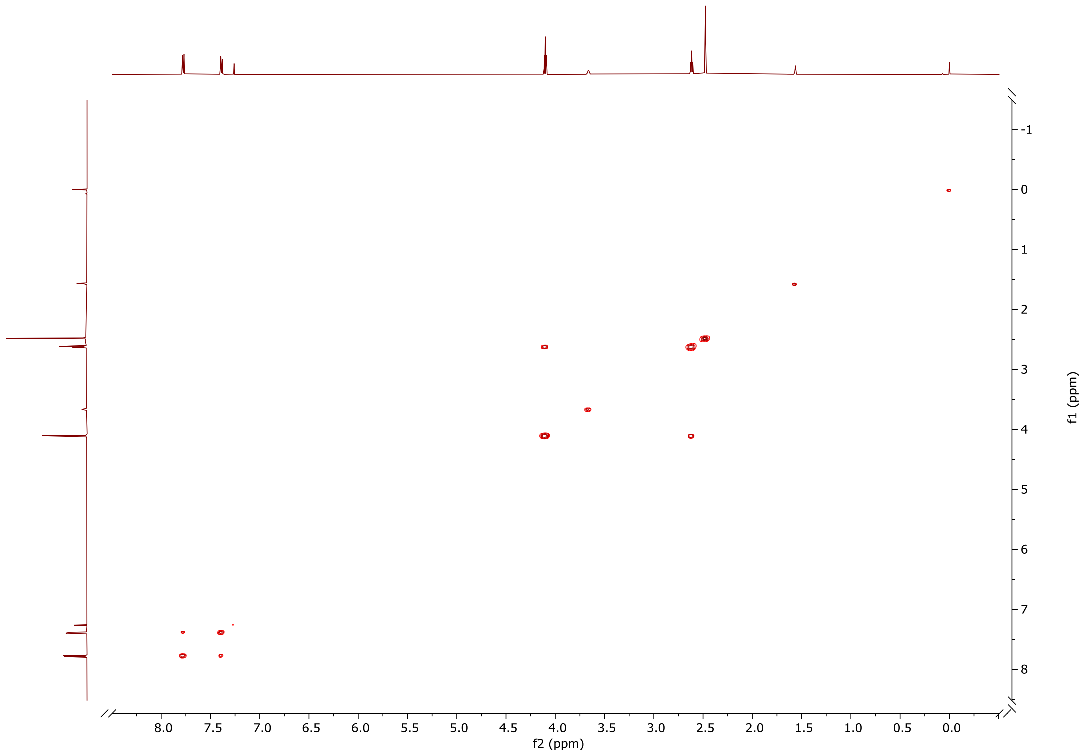

Figure S19. COSY 2-(C-*o*-carboranyl)-ethyl-4-methylbenzenesulfonate (**2**)

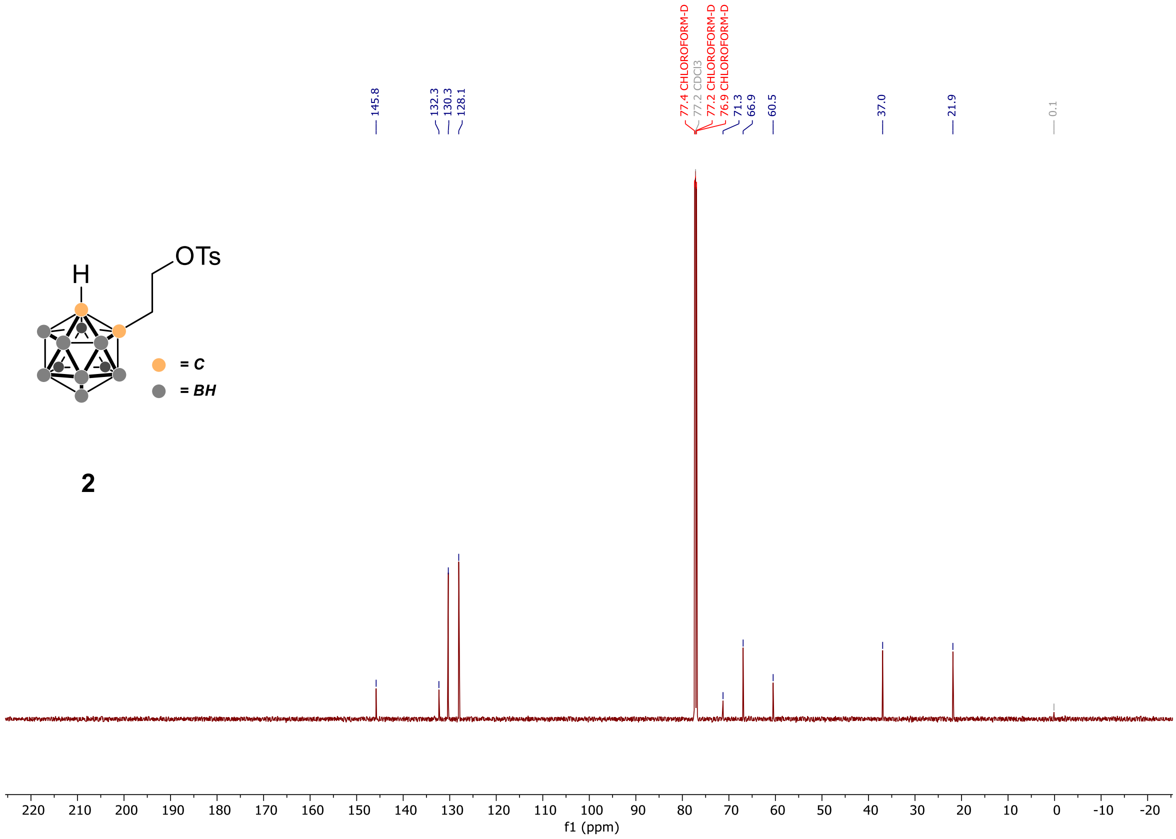

Figure S20. ^{13}C NMR 2-(C-*o*-carboranyl)-ethyl-4-methylbenzenesulfonate (**2**)

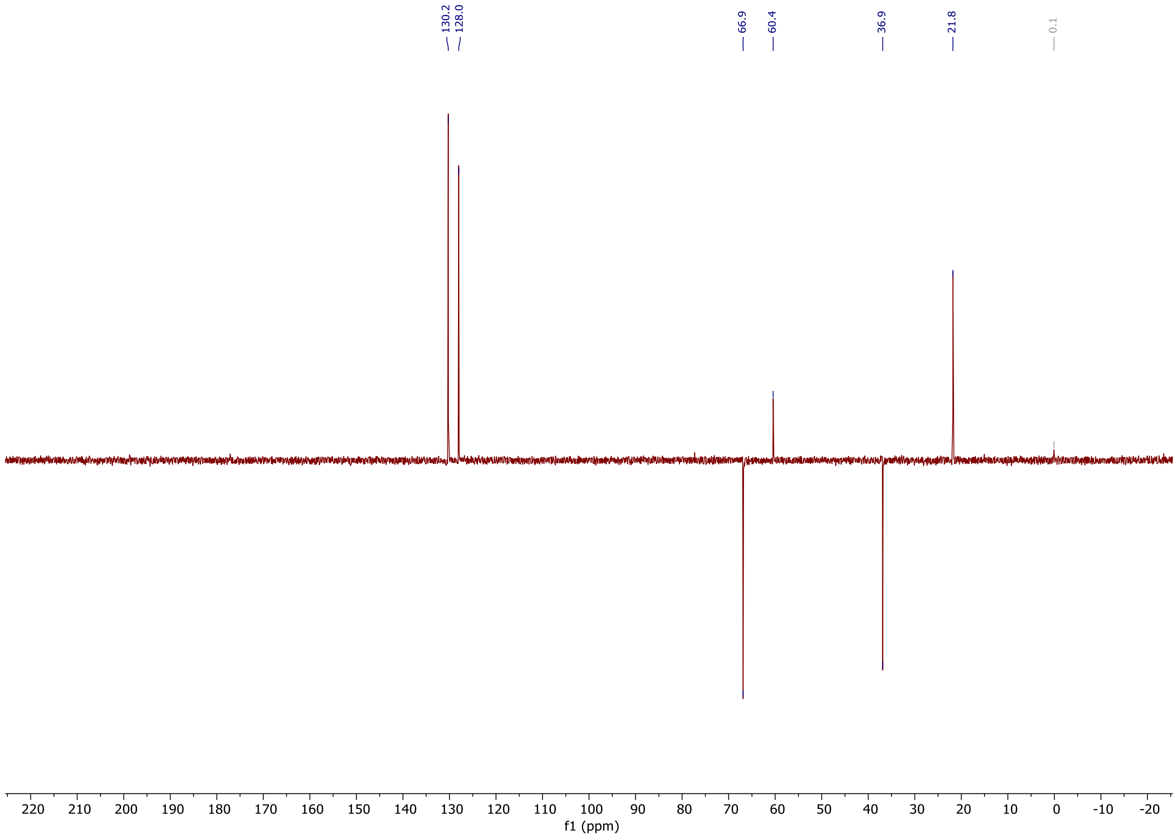

Figure S21. DEPT 135 2-(C-*o*-carboranyl)-ethyl-4-methylbenzenesulfonate (**2**)

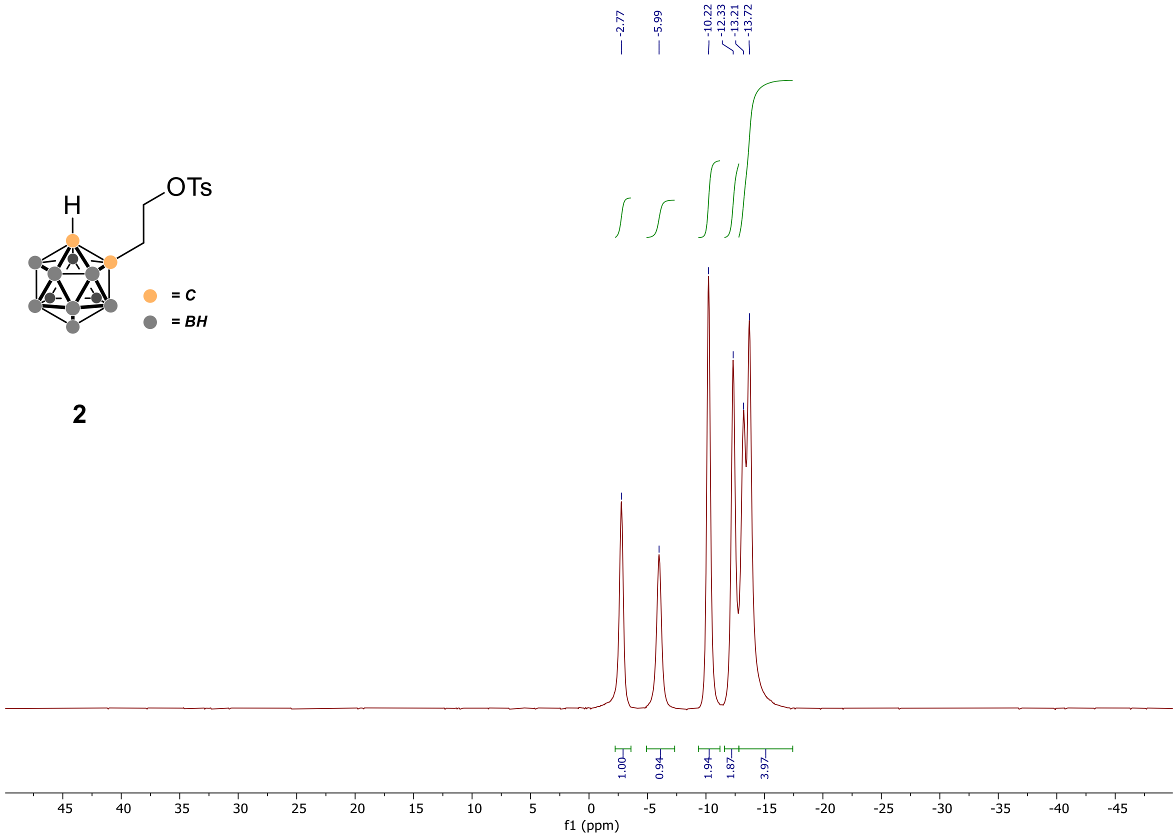

2

Figure S22. ^{11}B-NMR 2-(C-*o*-carboranyl)-ethyl-4-methylbenzenesulfonate (**2**)

*^{10}B-enriched 2-(C-o-carboranyl)-ethyl-4-methylbenzenesulfonate (^{10}**B-2**)*

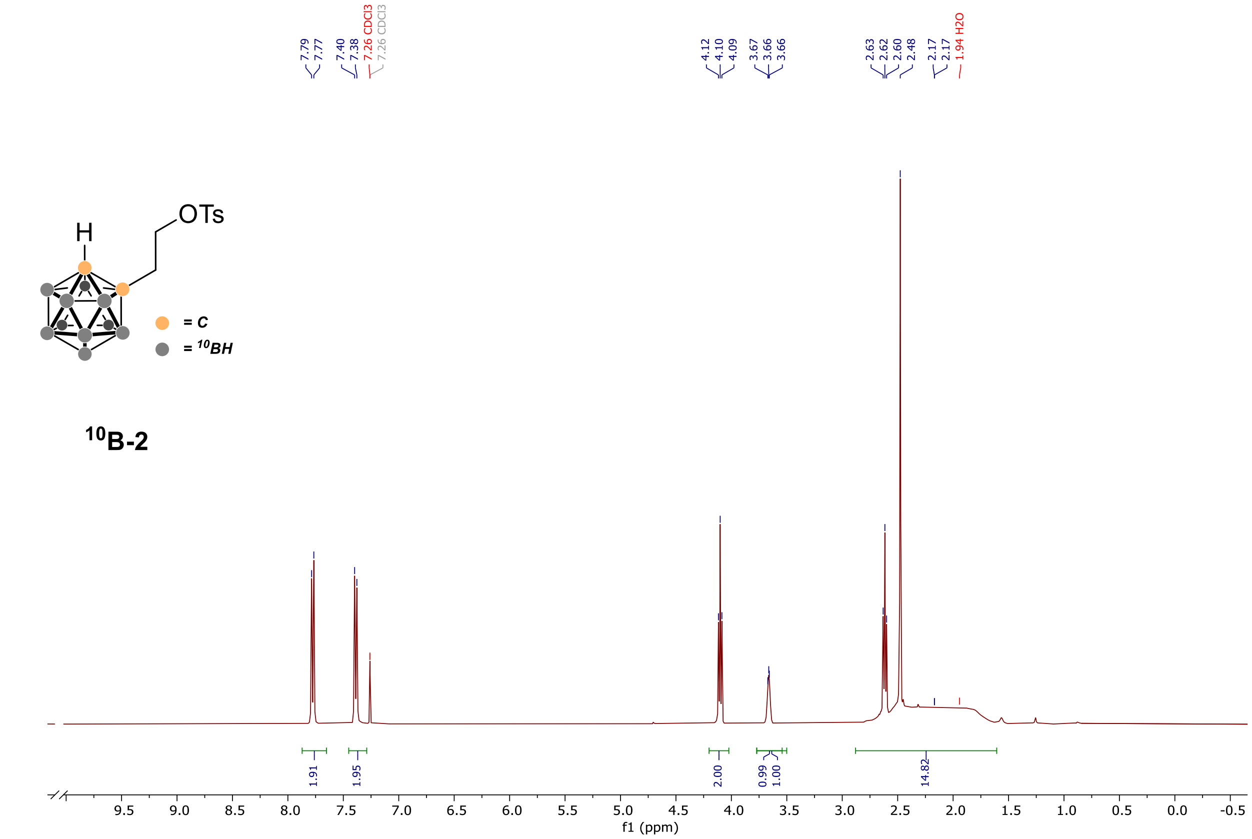

Figure S23. ^{1}H-NMR 2-(C-o-carboranyl)-ethyl-4-methylbenzenesulfonate (^{10}**B-2**)

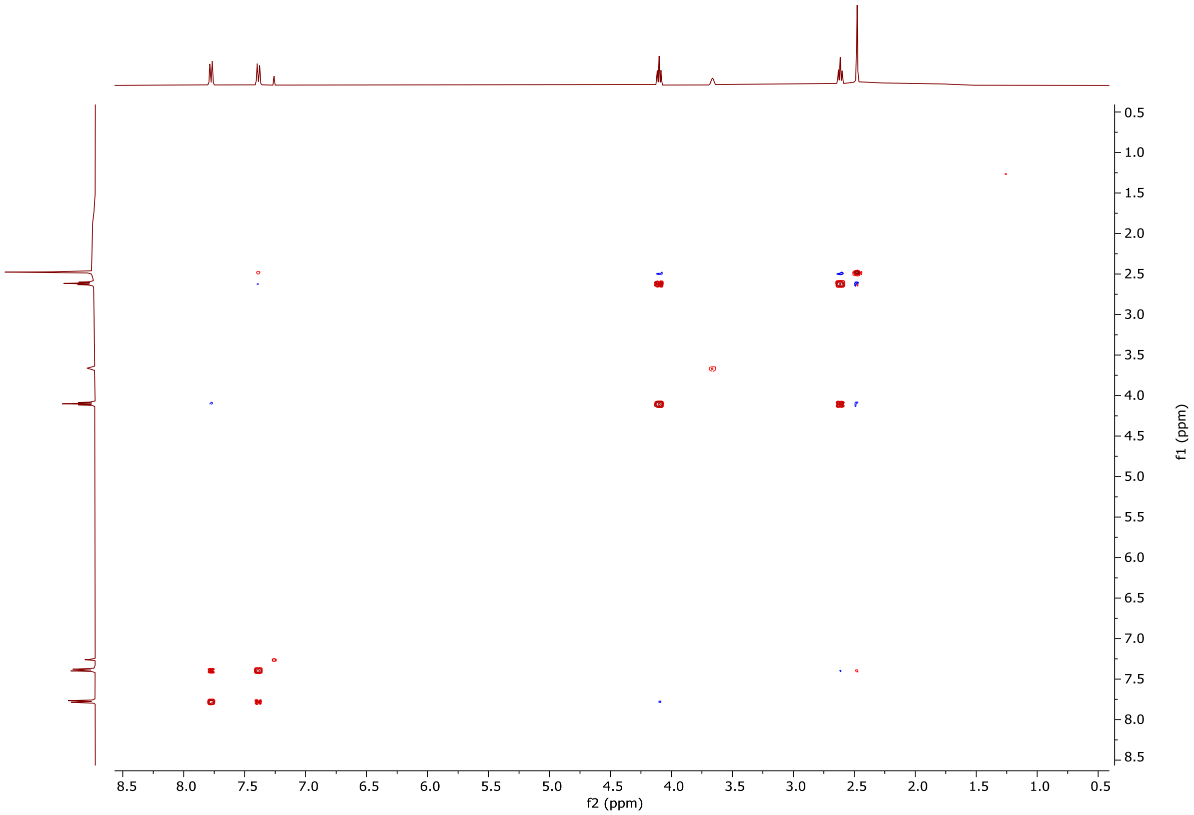

Figure S24. COSY 2-(C-o-carboranyl)-ethyl-4-methylbenzenesulfonate (^{10}**B-2**)

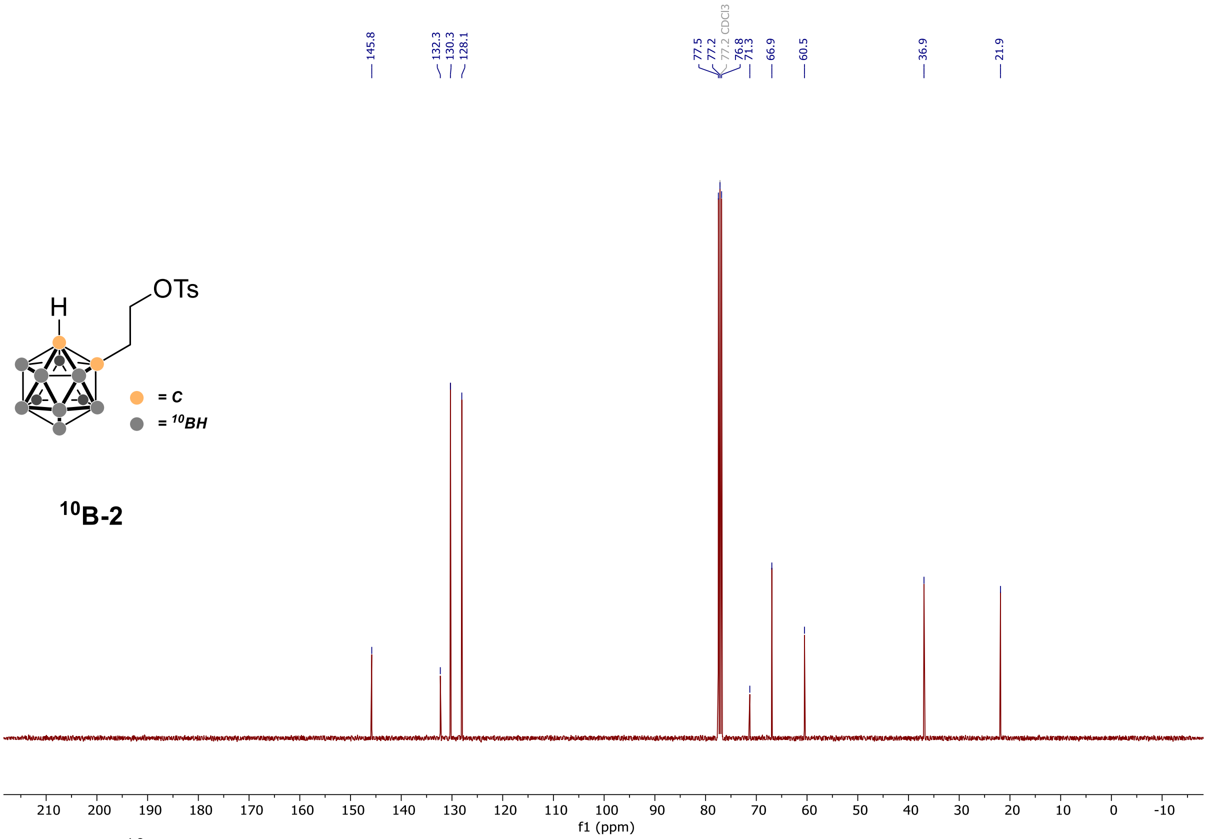

Figure S25. ^{13}C NMR 2-(C-o-carboranyl)-ethyl-4-methylbenzenesulfonate (**^{10}B-2**)

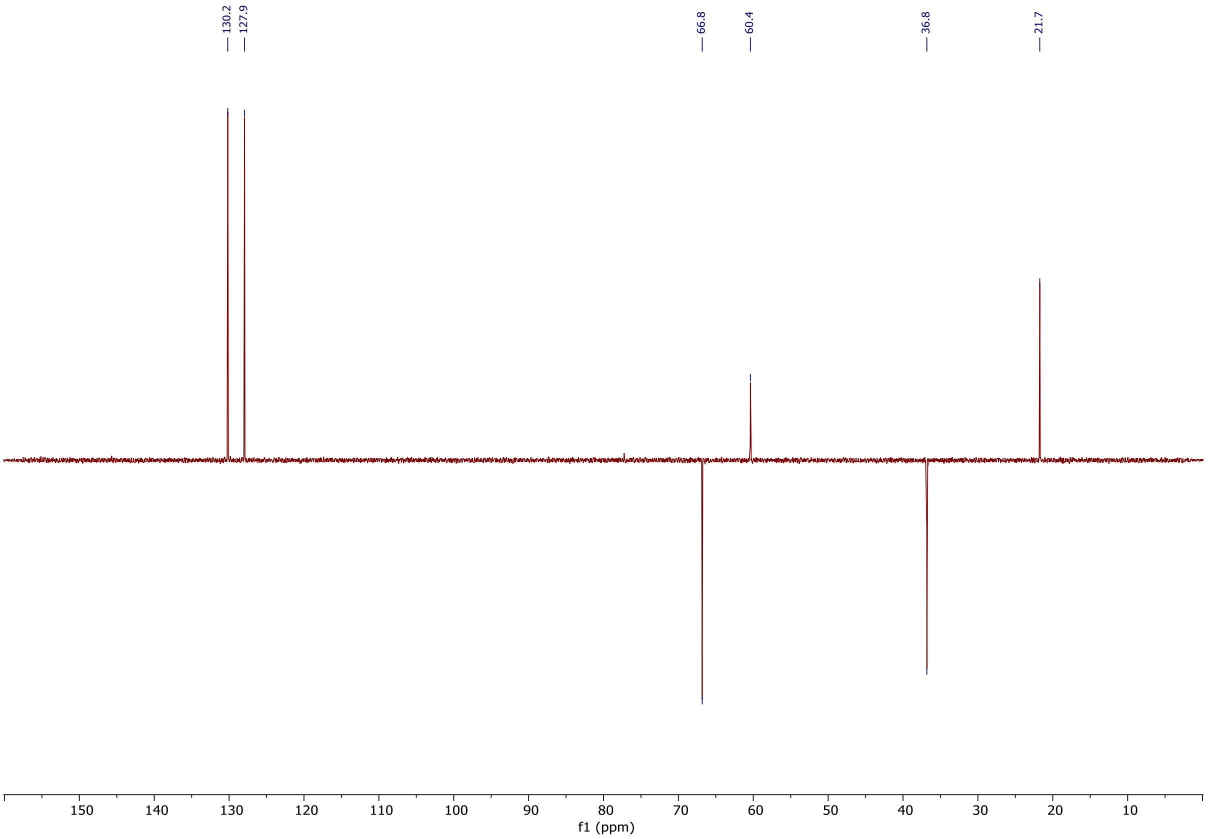

Figure S26. DEPT 135 2-(C-o-carboranyl)-ethyl-4-methylbenzenesulfonate (**^{10}B-2**)

*2-(C-o-carboranyl)-1-azidoethane (**3**)*

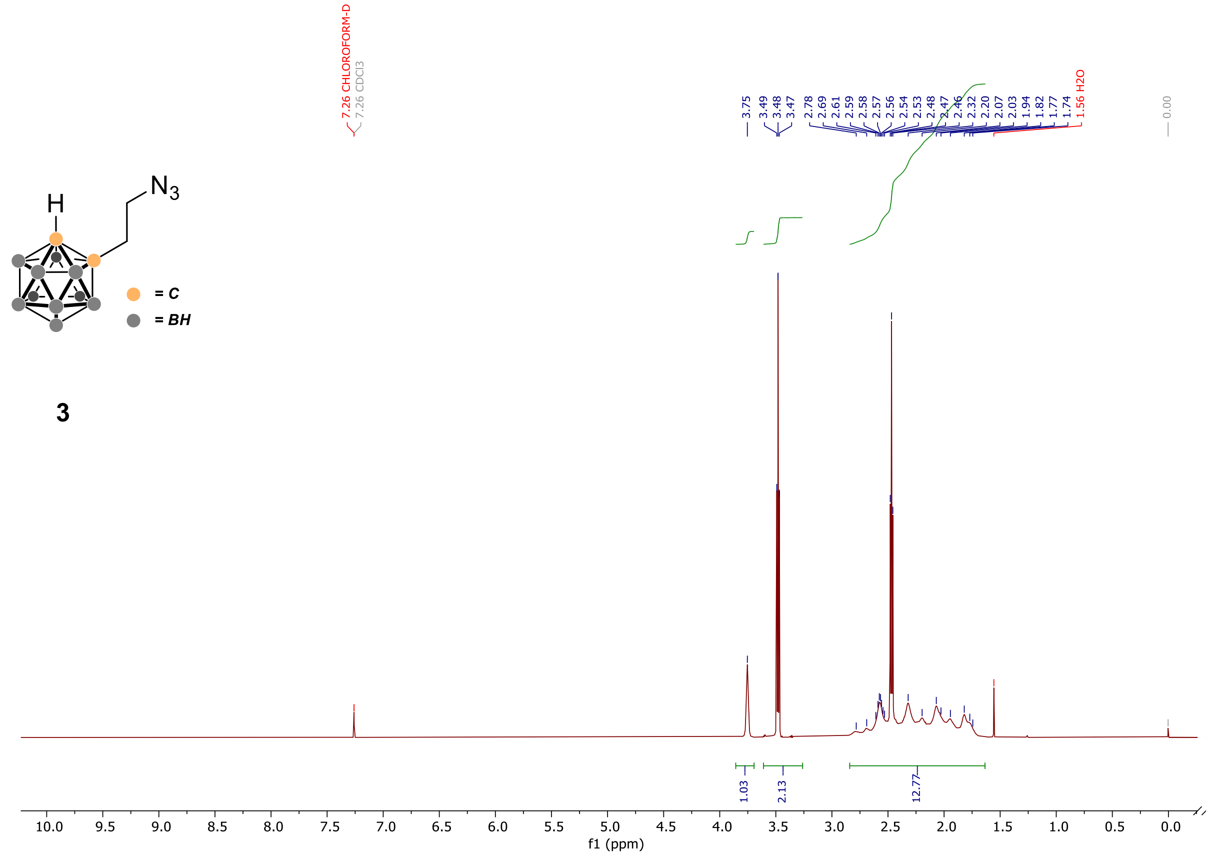

Figure S27. ^{1}H-NMR 2-(C-*o*-carboranyl)-1-azido ethane (**3**)

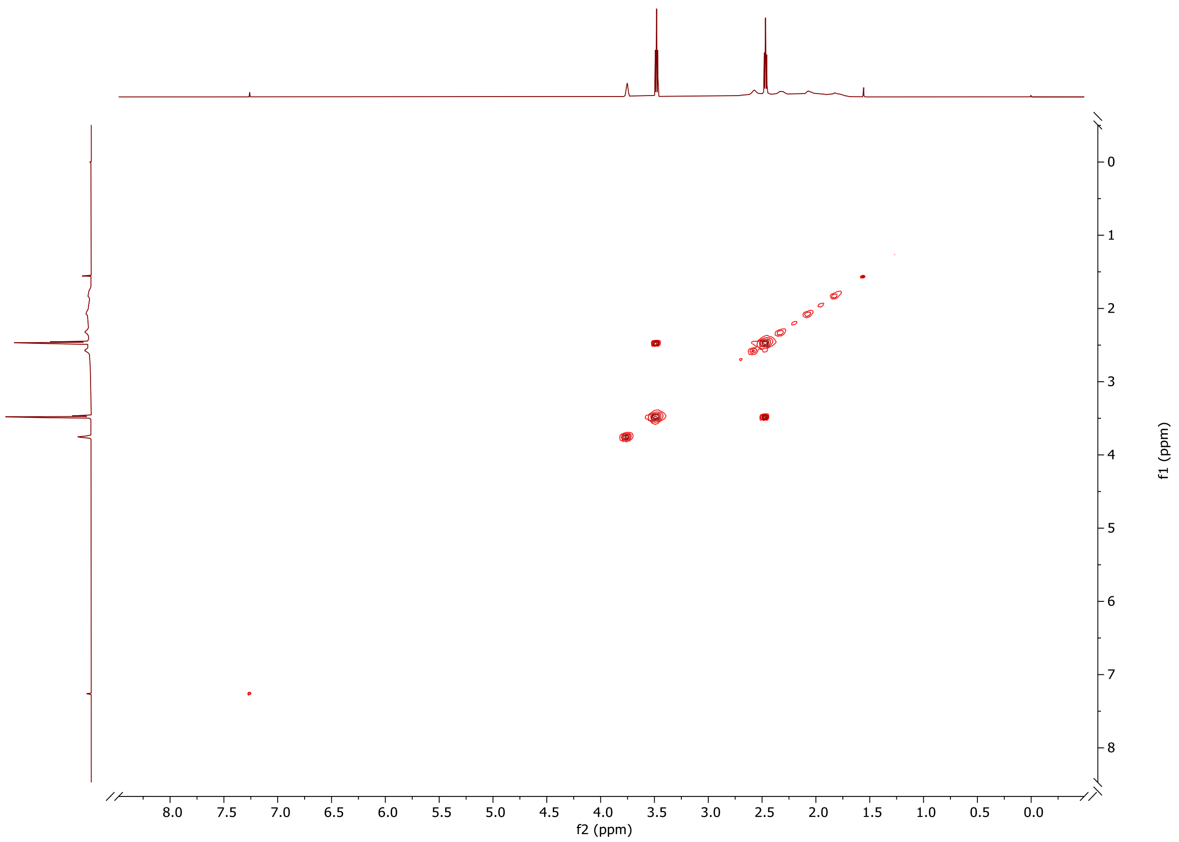

Figure S28. COSY 2-(C-*o*-carboranyl)-1-azido ethane (**3**)

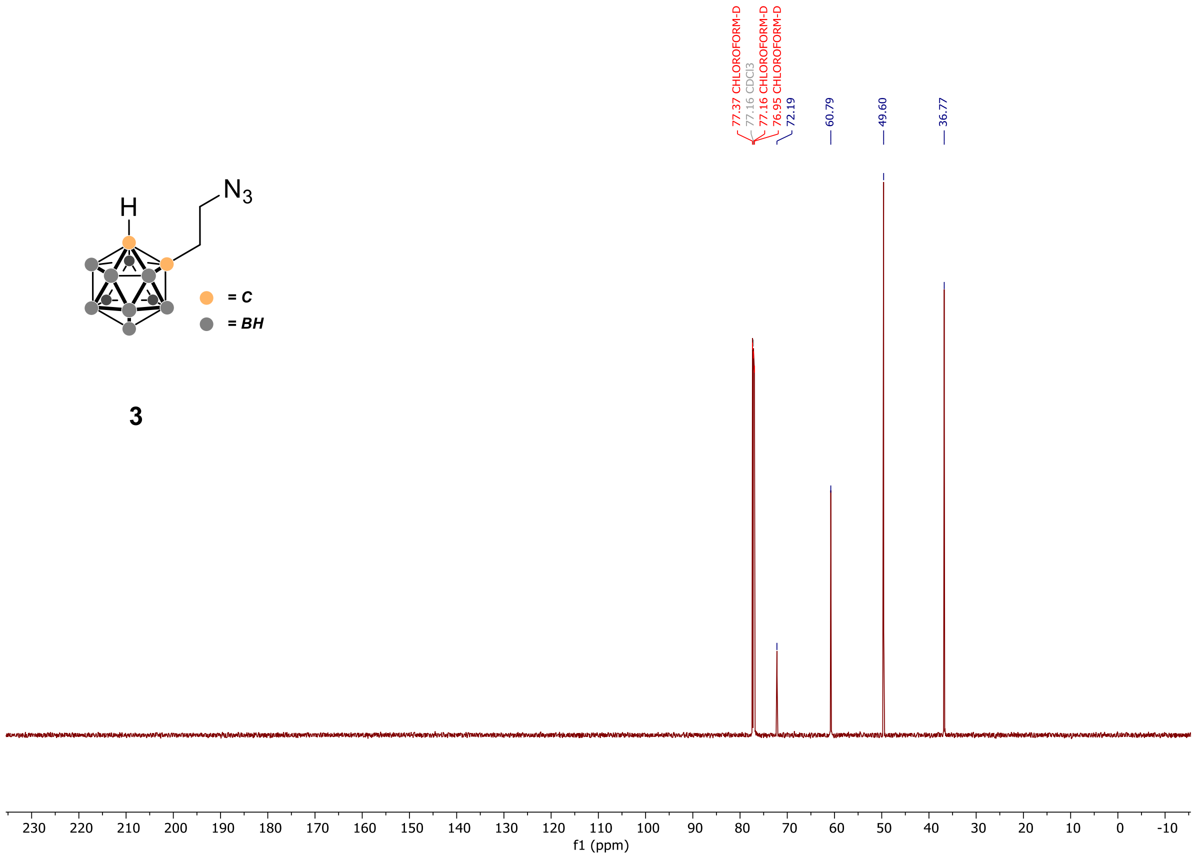

Figure S29. ^{13}C NMR 2-(C-*o*-carboranyl)-1-azido ethane (**3**)

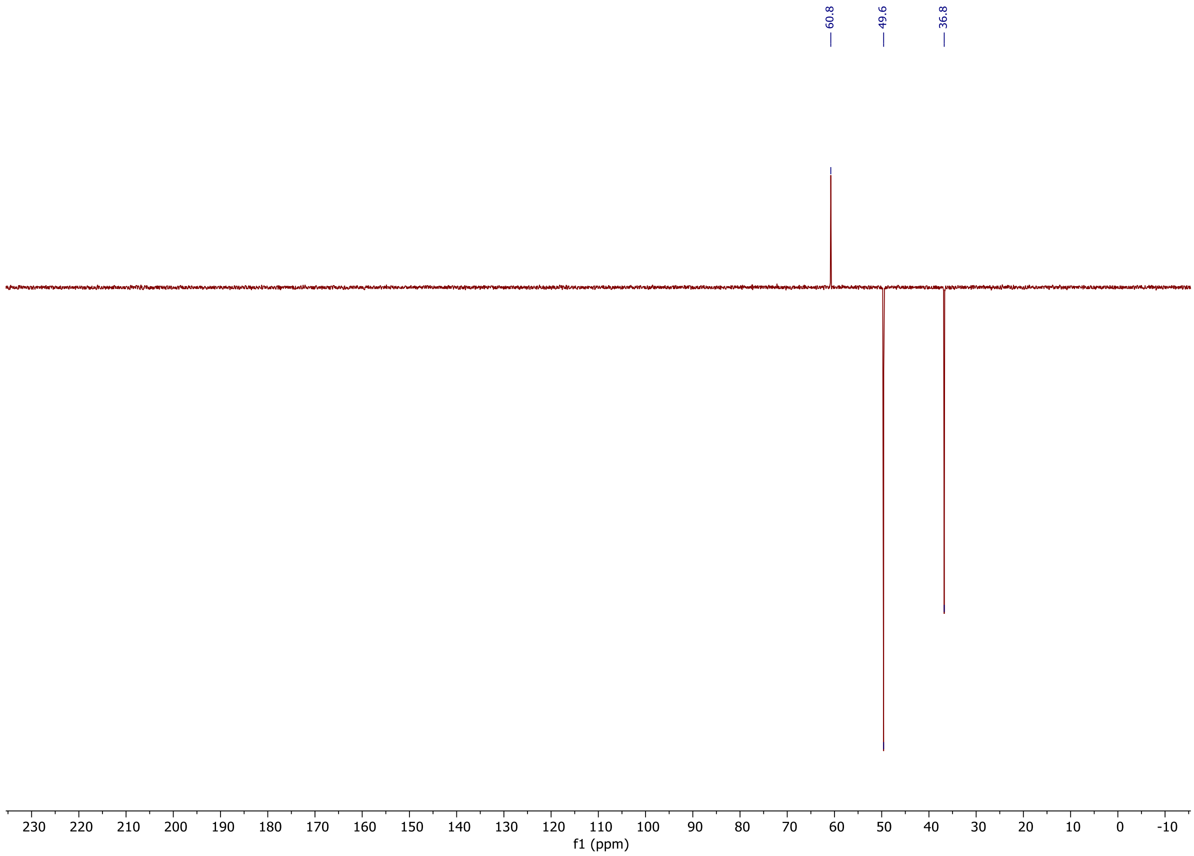

Figure S30. DEPT 135 2-(C-*o*-carboranyl)-1-azido ethane (**3**)

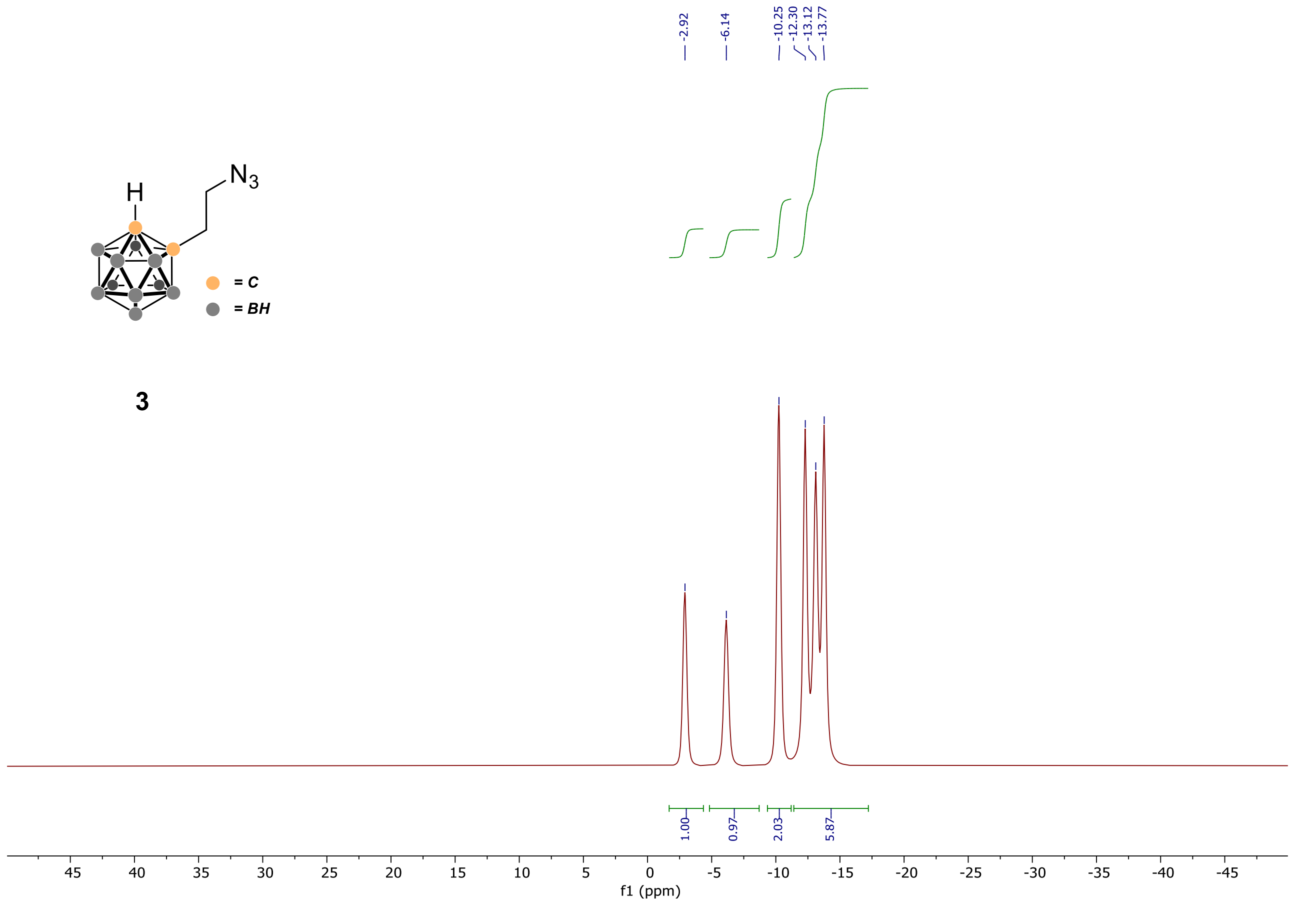

Figure S31: ^{11}B-NMR 2-(C-*o*-carboranyl)-1-azido ethane (**3**)

*[10]B-enriched 2-(C-o-carboranyl)-1-azidoethane (**[10]B-3**)*

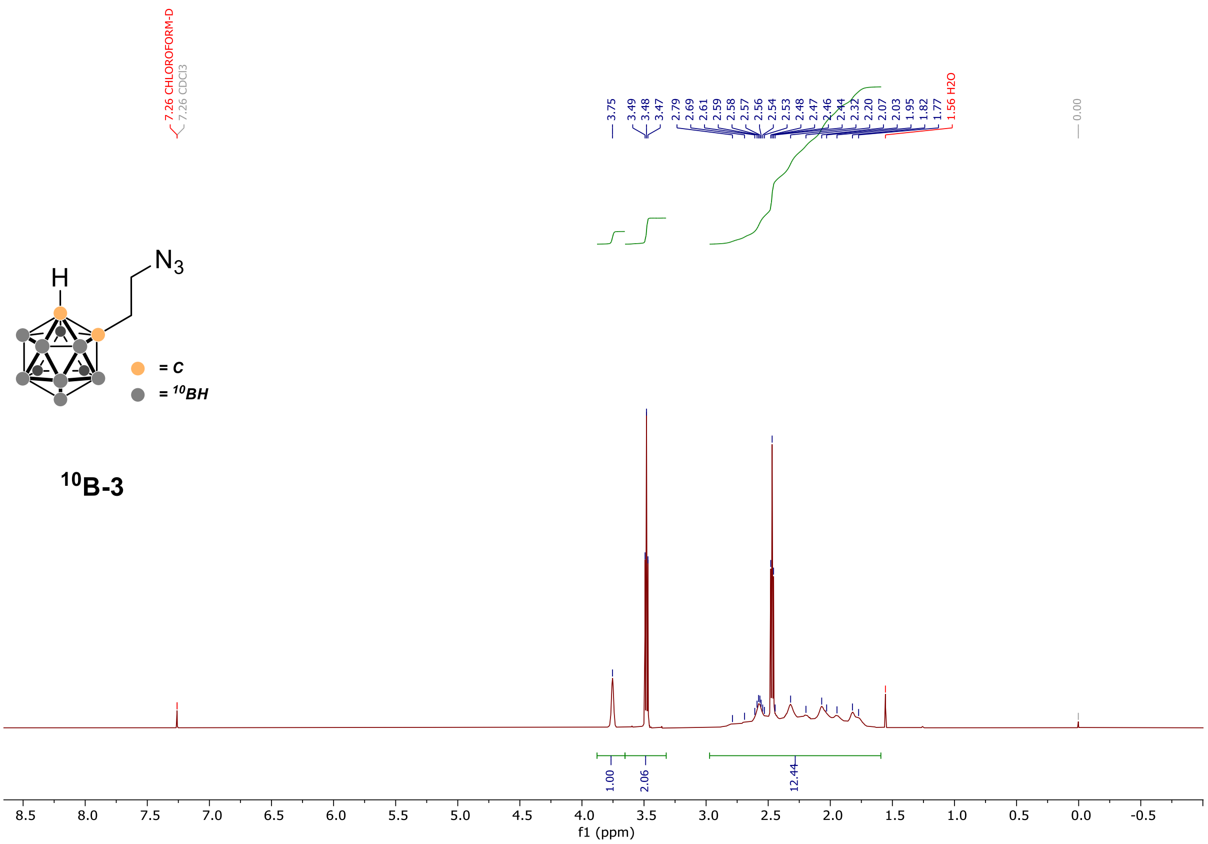

Figure S32. ^{1}H-NMR ^{10}B-enriched 2-(C-o-carboranyl)-1-azidoethane (**^{10}B-3**)

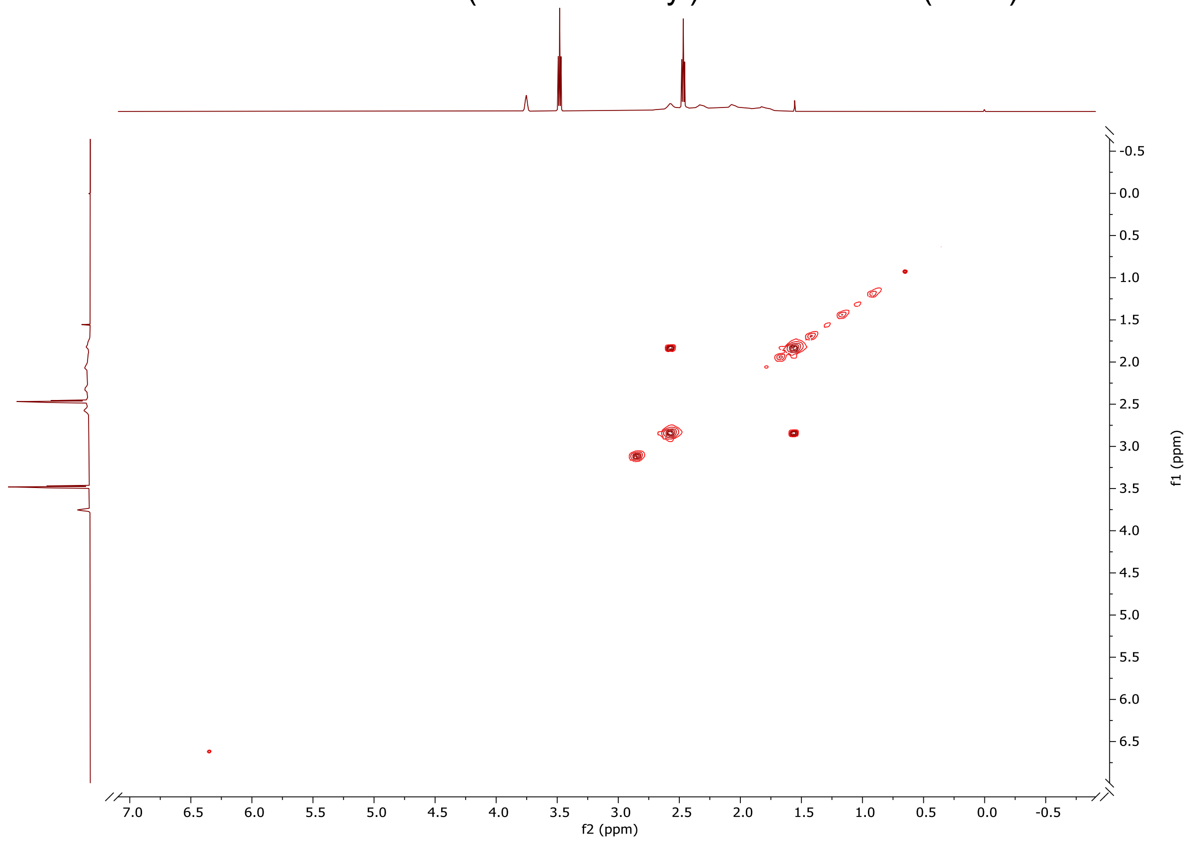

Figure S33. COSY ^{10}B-enriched 2-(C-o-carboranyl)-1-azidoethane (**^{10}B-3**)

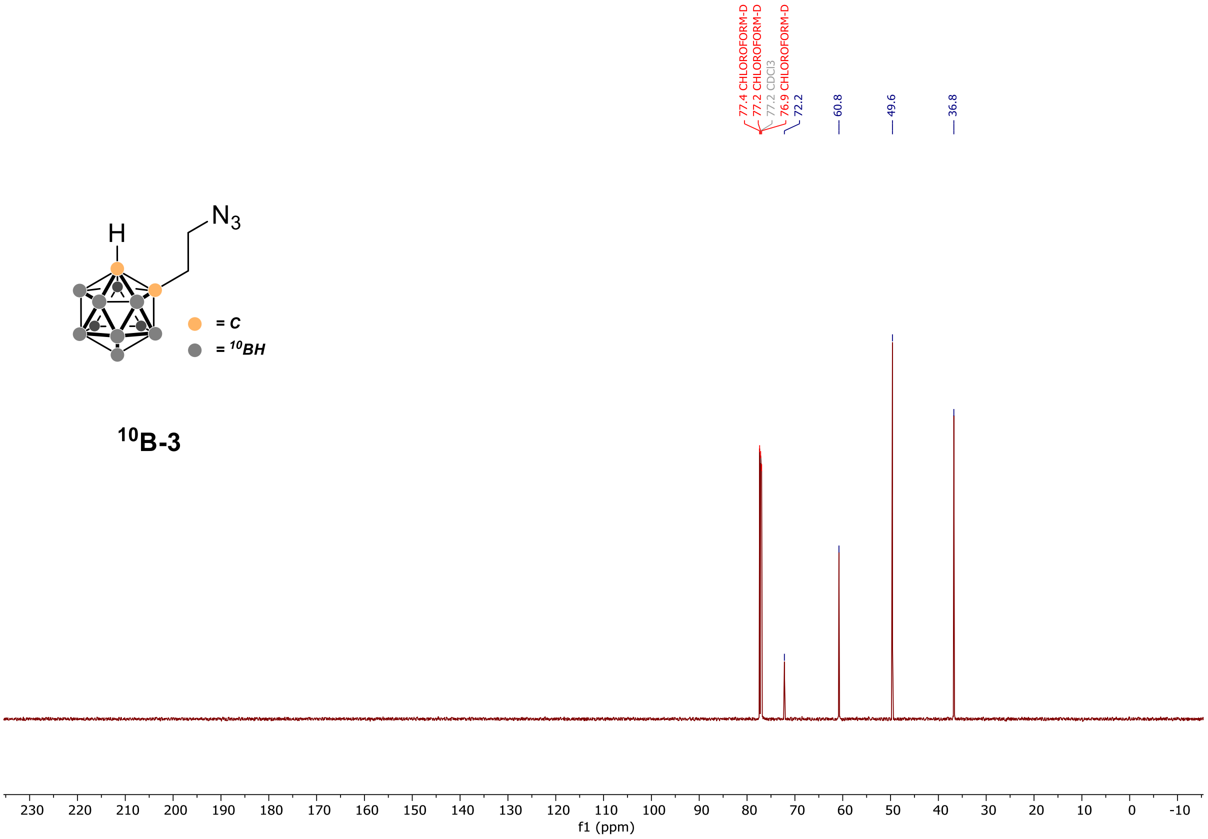

Figure S34. ^{13}C NMR ^{10}B-enriched 2-(C-o-carboranyl)-1-azidoethane (**^{10}B-3**)

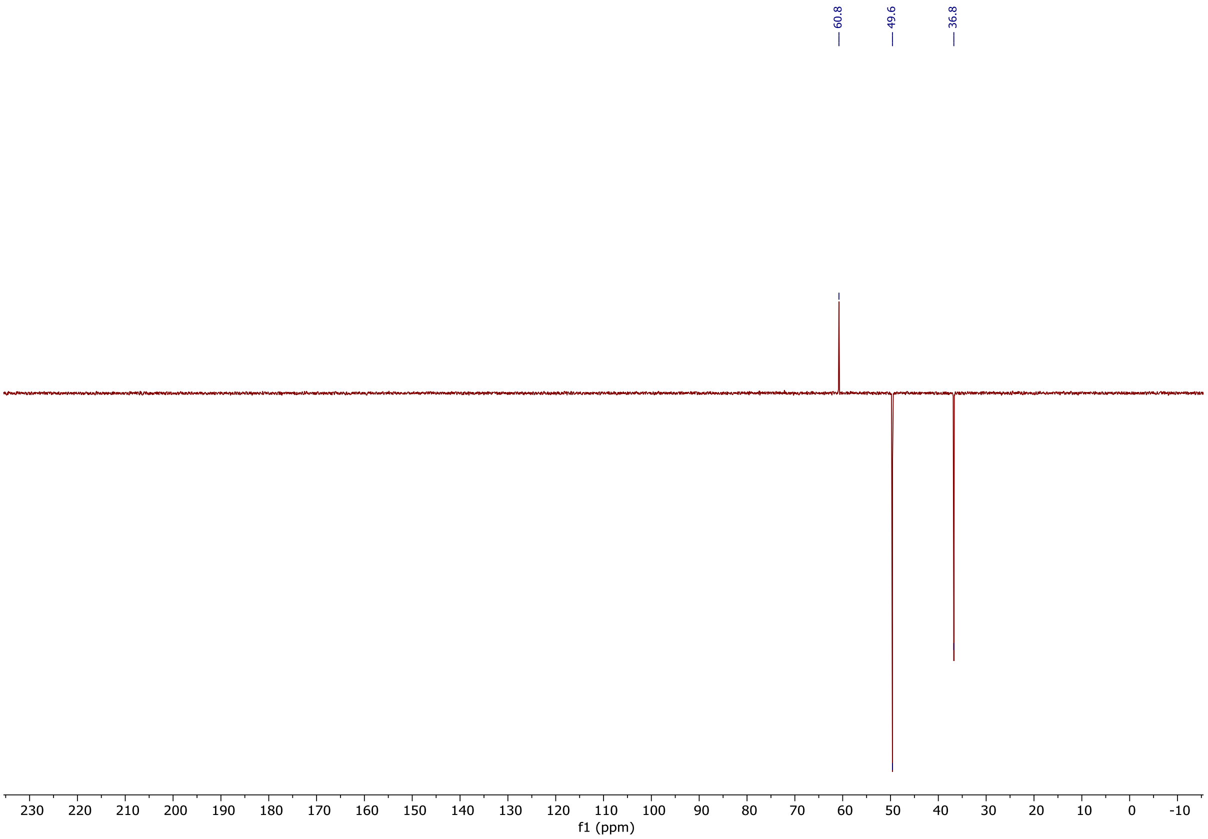

Figure S35. DEPT 135 ^{10}B-enriched 2-(C-o-carboranyl)-1-azidoethane (**^{10}B-3**)

*2,6-bis((E)-3,4-bis((tetrahydro-2H-pyran-2-yl)oxy)benzylidene)-4-hydroxycyclohexan-1-one (**6**)*

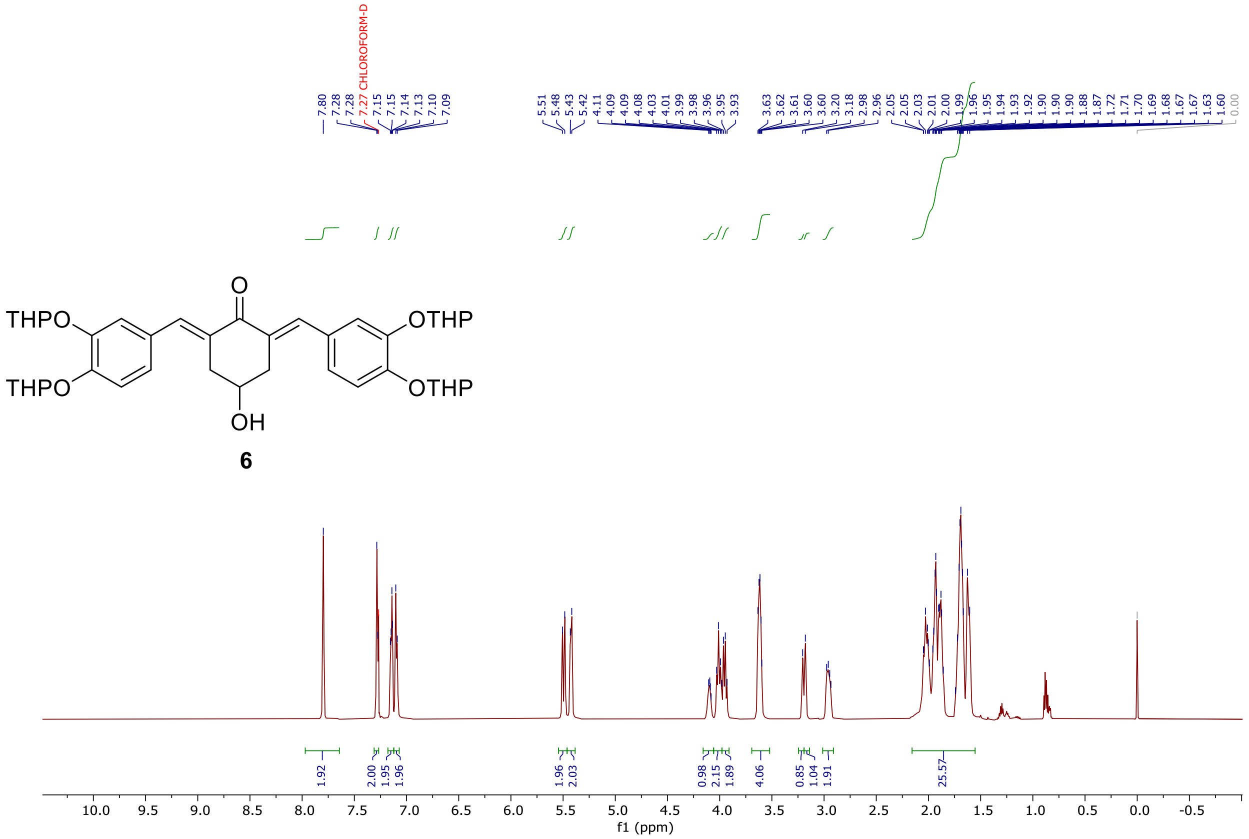

Figure S36. ^{1}H-NMR 2,6-bis((*E*)-3,4-bis((tetrahydro-2*H*-pyran-2-yl)oxy)benzylidene)-4-hydroxycyclohexan-1-one (**6**)

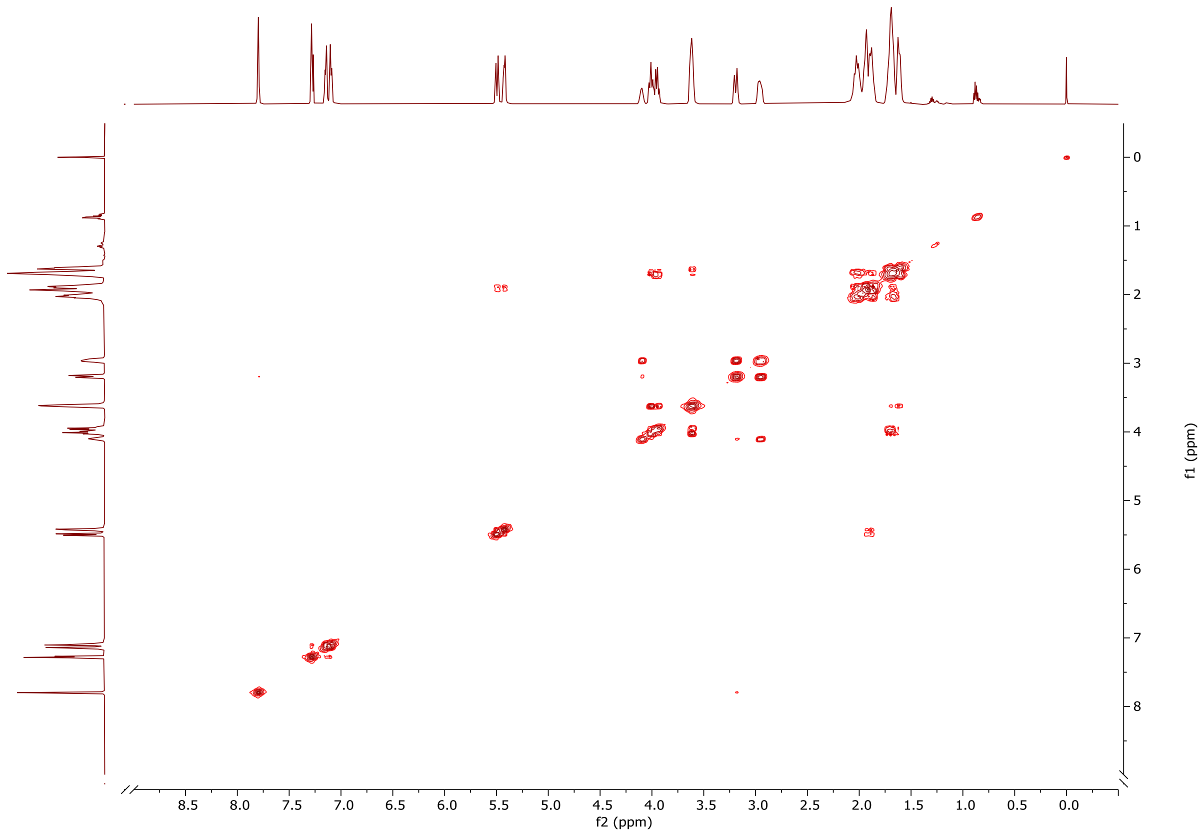

Figure S37. COSY 2,6-bis((*E*)-3,4-bis((tetrahydro-2*H*-pyran-2-yl)oxy)benzylidene)-4-hydroxycyclohexan-1-one (**6**)

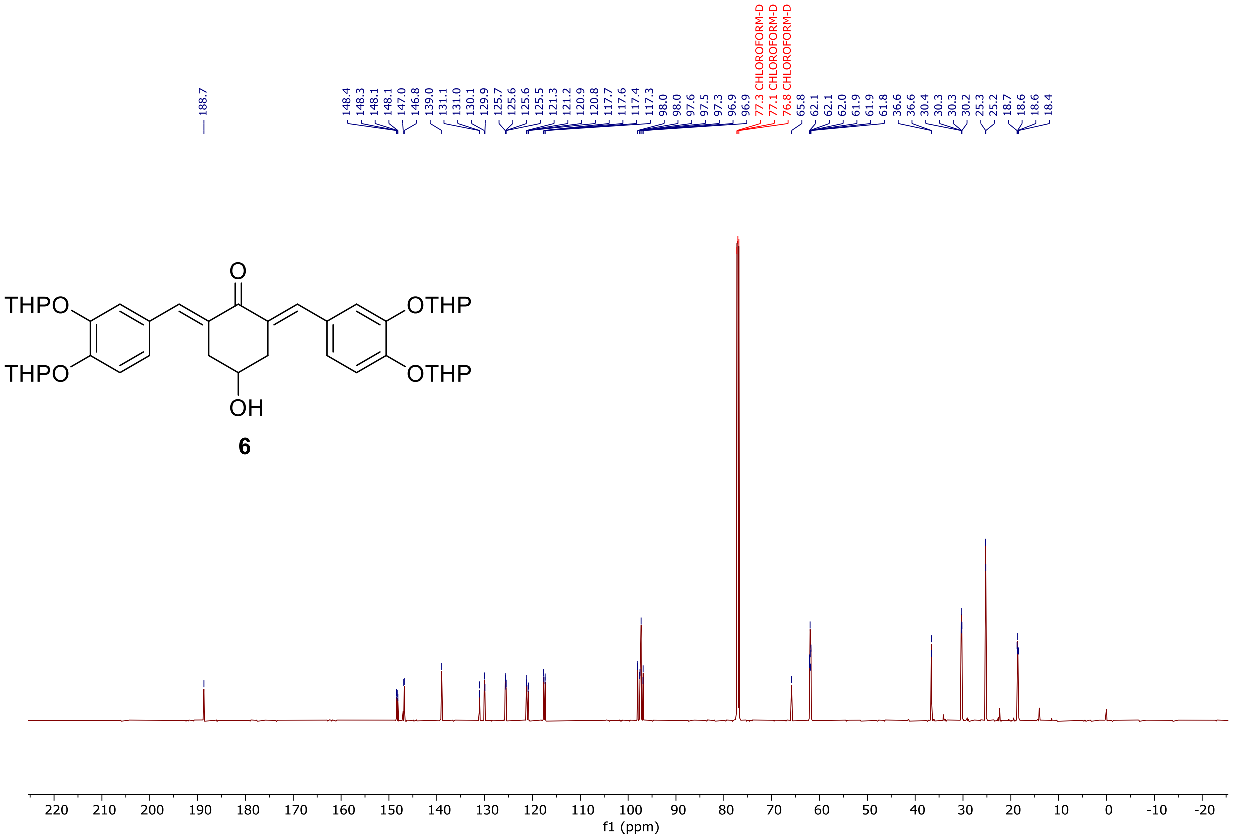

Figure S38. ^{13}C NMR 2,6-bis((*E*)-3,4-bis((tetrahydro-2H-pyran-2-yl)oxy)benzylidene)-4-hydroxycyclohexan-1-one (**6**)

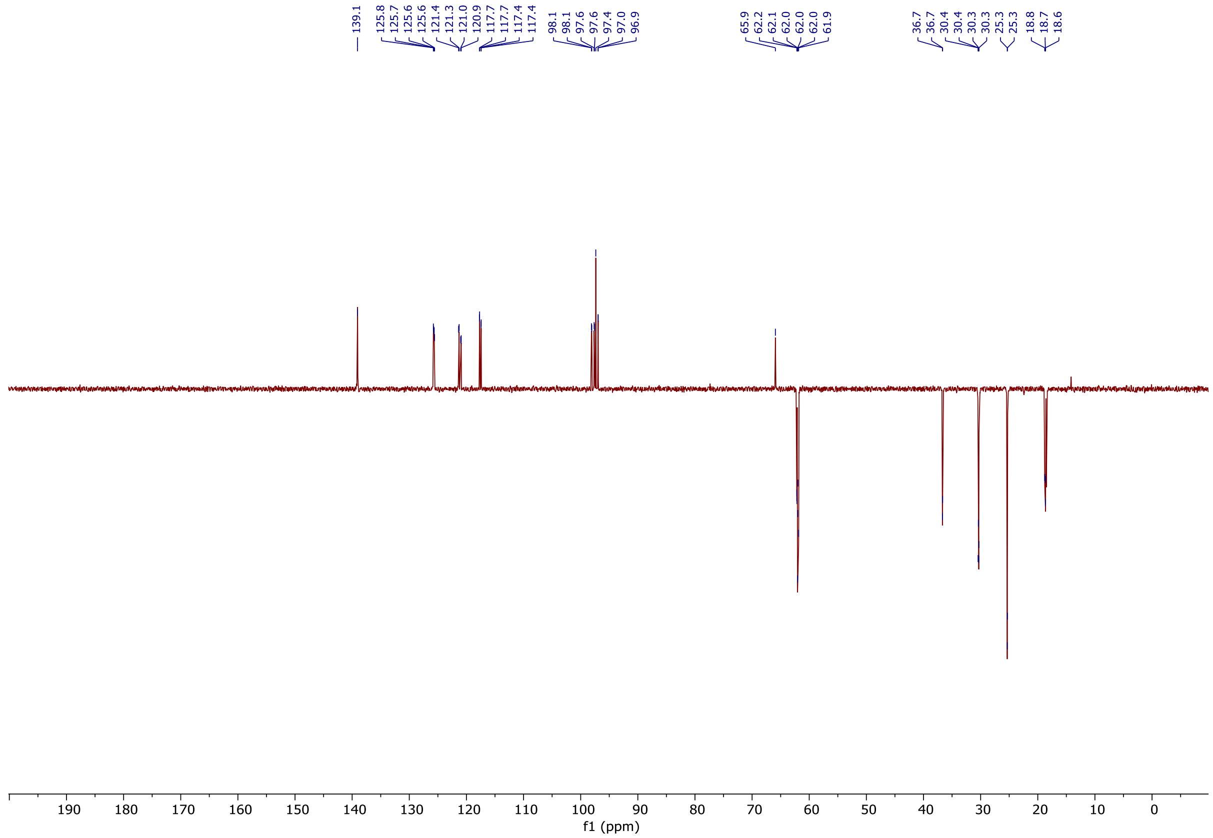

Figure S39. DEPT 135 2,6-bis((*E*)-3,4-bis((tetrahydro-2H-pyran-2-yl)oxy)benzylidene)-4-hydroxycyclohexan-1-one (**6**)

*4-hydroxy-2,6-bis((E)-3-methoxy-4-((tetrahydro-2H-pyran-2-yl)oxy)benzylidene) cyclohexan-1-one (**6a**)*

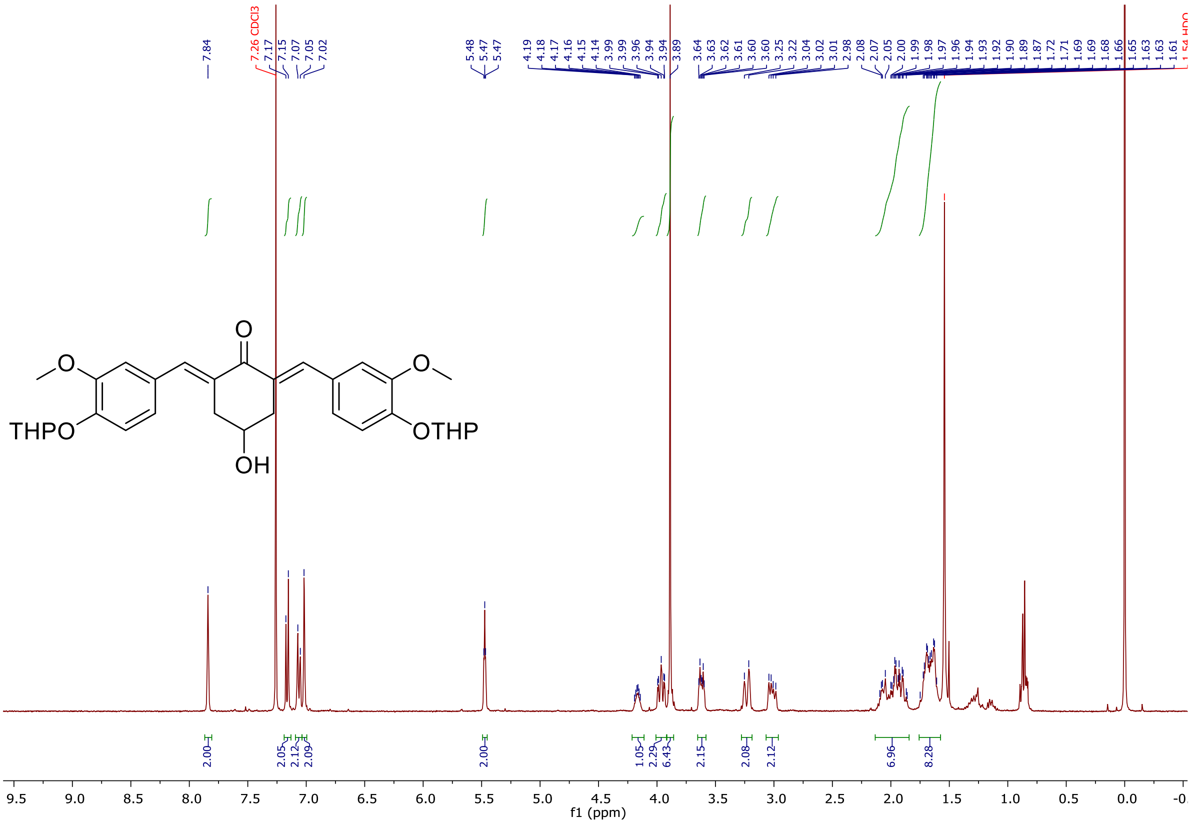

Figure S40. ^{1}H-NMR 4-hydroxy-2,6-bis((*E*)-3-methoxy-4-((tetrahydro-2*H*-pyran-2-yl)oxy)benzylidene)cyclohexan-1-one (**6a**)

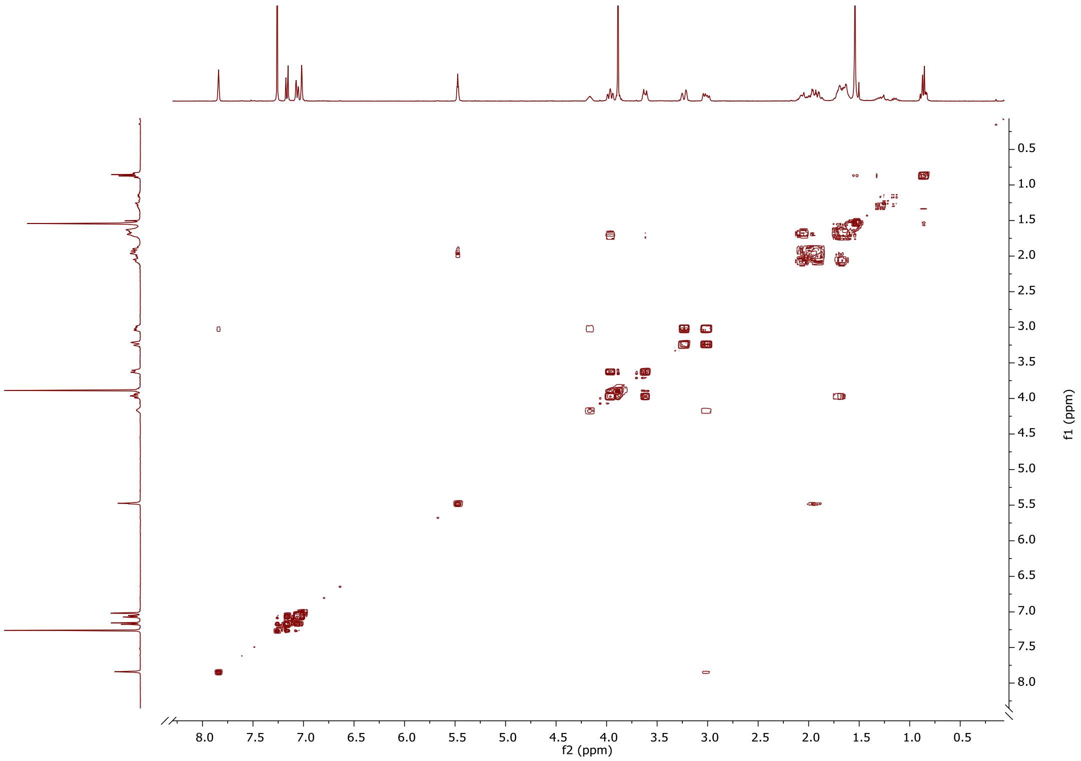

Figure S41. COSY 4-hydroxy-2,6-bis((*E*)-3-methoxy-4-((tetrahydro-2*H*-pyran-2-yl)oxy)benzylidene)cyclohexan-1-one (**6a**)

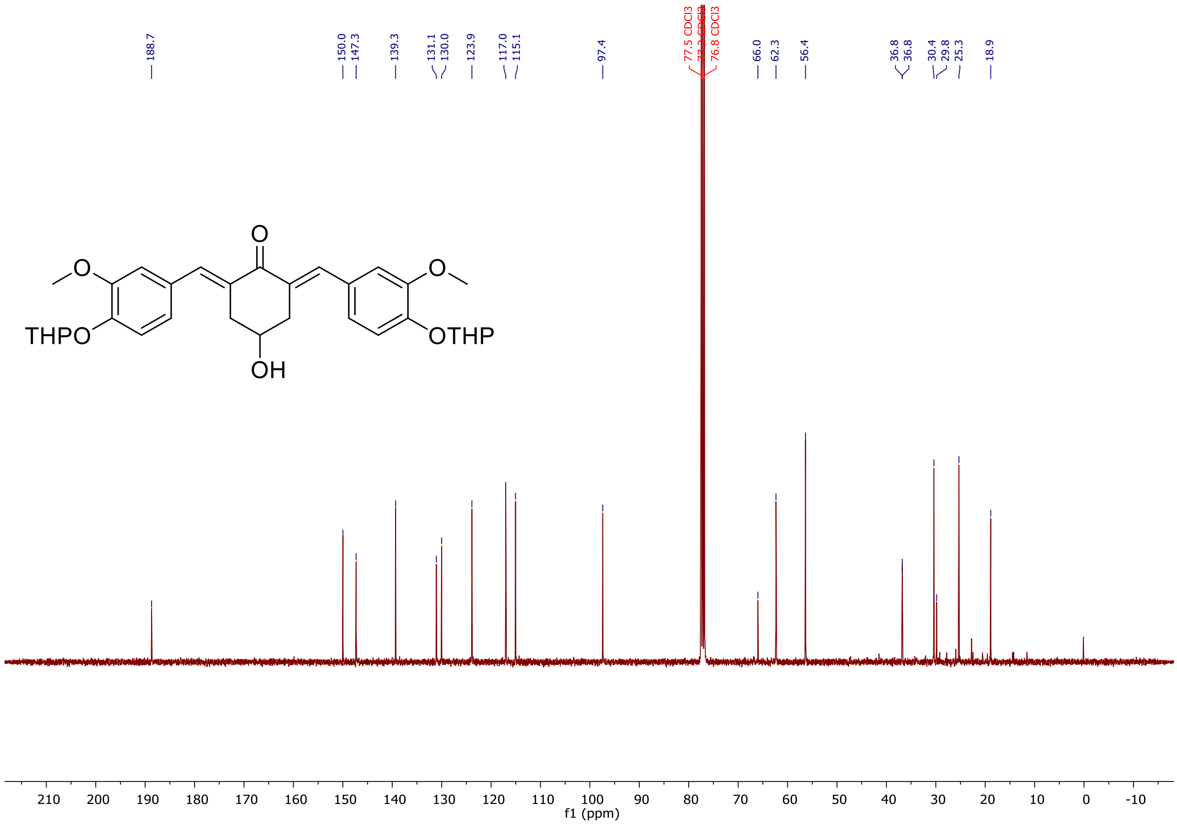

Figure S42. ^{13}C NMR 4-hydroxy-2,6-bis((*E*)-3-methoxy-4-((tetrahydro-2*H*-pyran-2-yl)oxy)benzylidene)cyclohexan-1-one (**6a**)

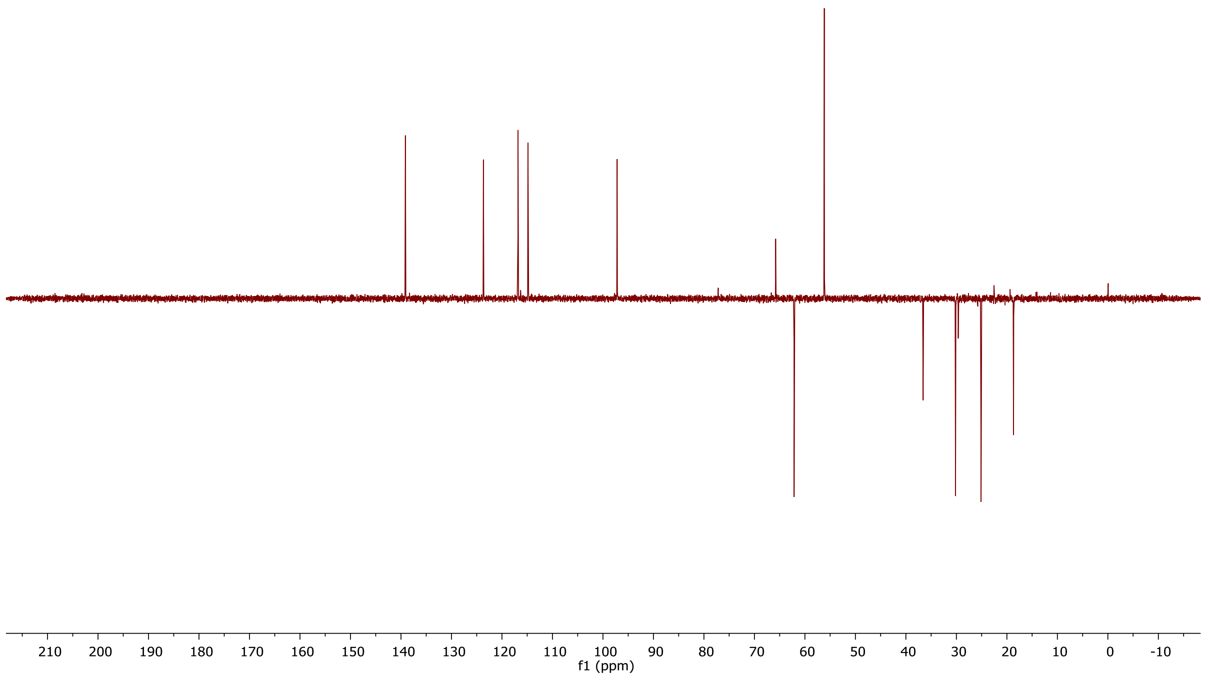

Figure S43. DEPT 135 4-hydroxy-2,6-bis((*E*)-3-methoxy-4-((tetrahydro-2*H*-pyran-2-yl)oxy)benzylidene)cyclohexan-1-one (**6a**)

2,6-bis((E)-3,4-bis((tetrahydro-2H-pyran-2-yl)oxy)benzylidene)-4-(prop-2-yn-1-yloxy) cyclohexan-1-one (7)

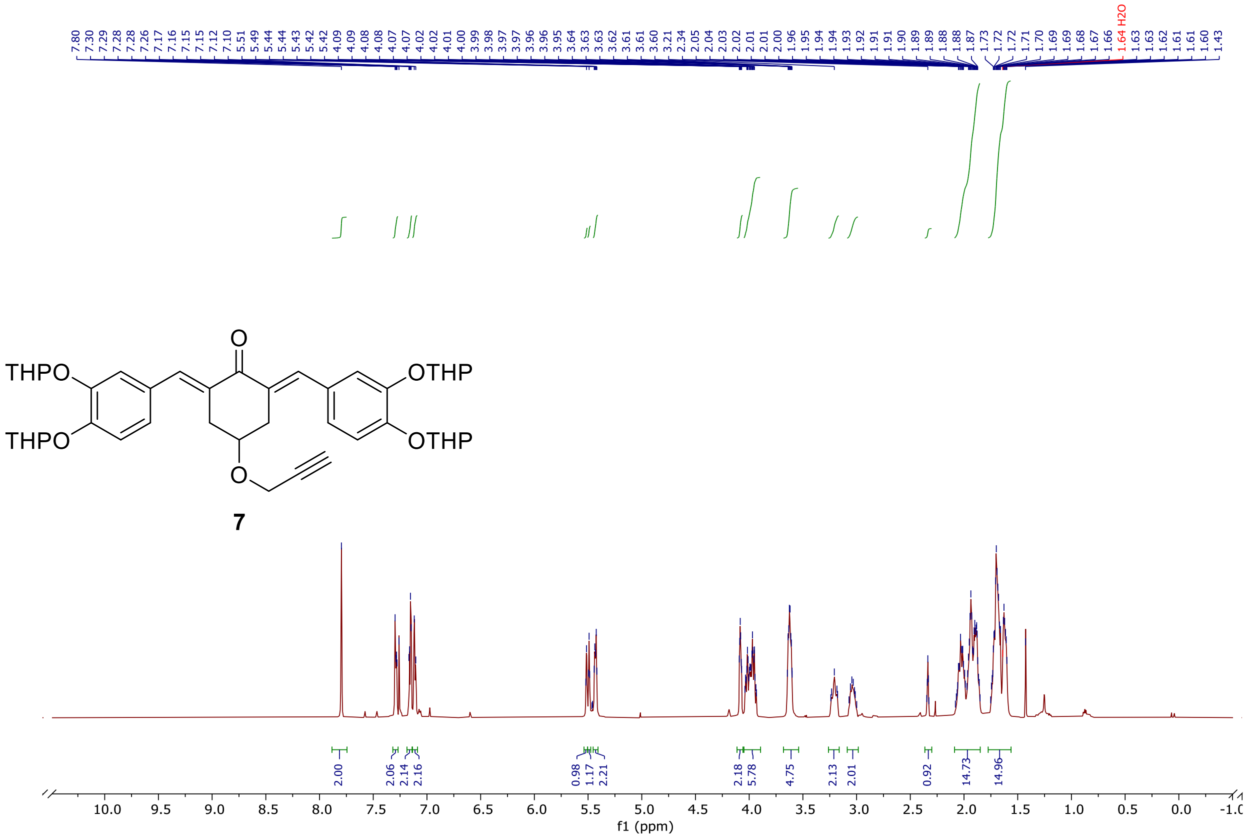

Figure S44. ^{1}H-NMR 2,6-bis((*E*)-3,4-bis((tetrahydro-2H-pyran-2-yl)oxy)benzylidene)-4-(prop-2-yn-1-yloxy) cyclohexan-1-one (**7**)

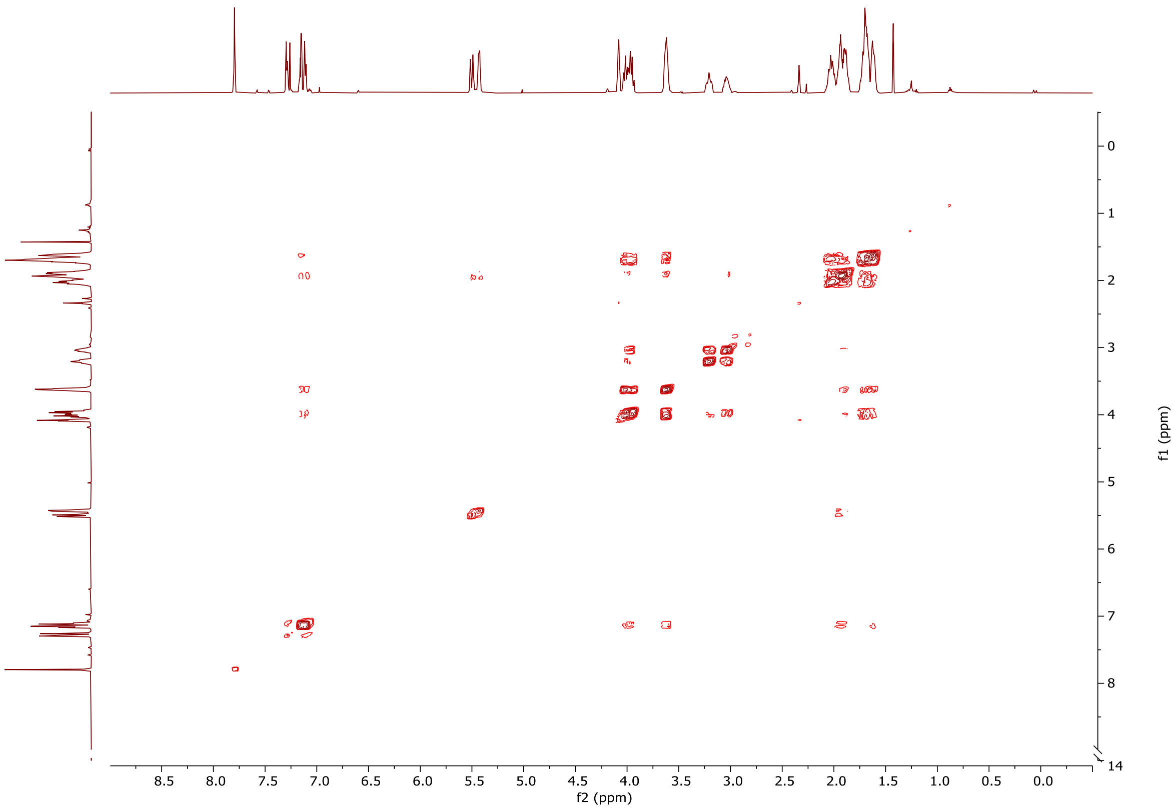

Figure S45. COSY 2,6-bis((*E*)-3,4-bis((tetrahydro-2H-pyran-2-yl)oxy)benzylidene)-4-(prop-2-yn-1-yloxy) cyclohexan-1-one (**7**)

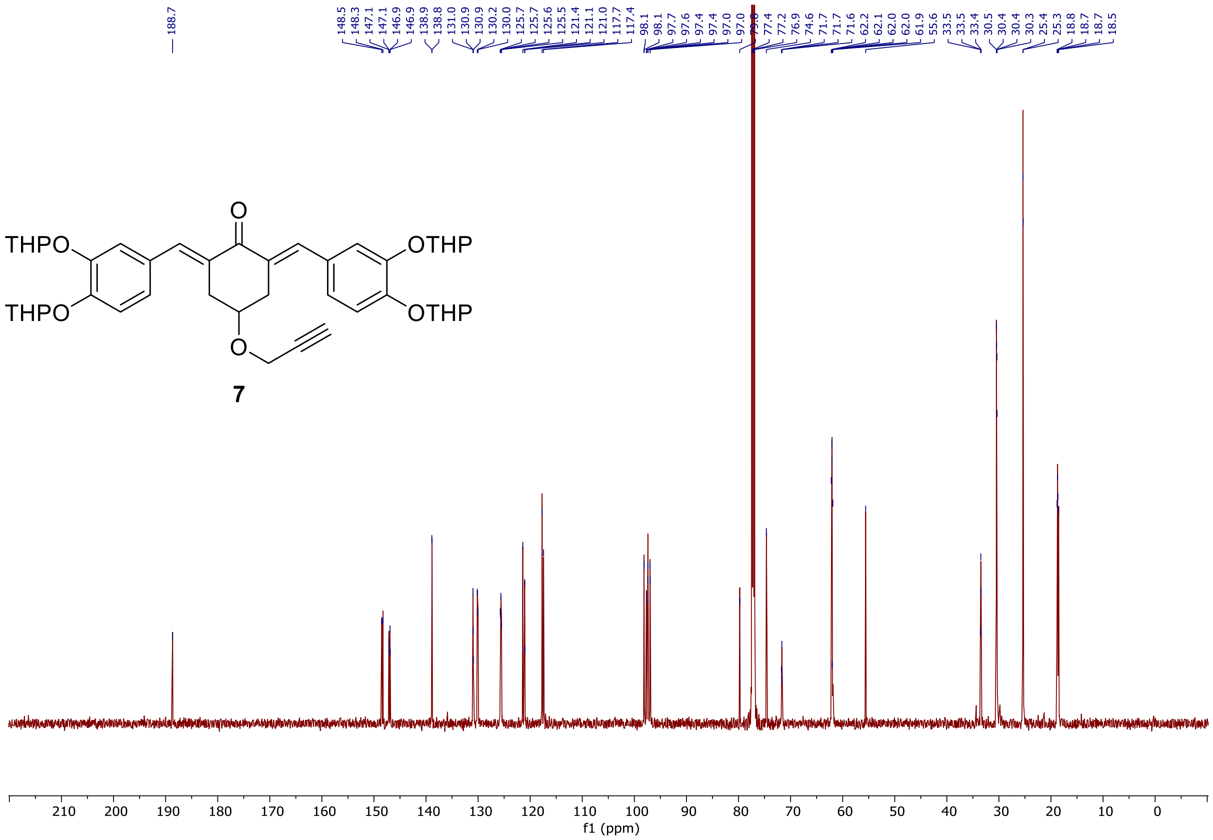

Figure S46. ^{13}C NMR 2,6-bis((*E*)-3,4-bis((tetrahydro-2H-pyran-2-yl)oxy)benzylidene)-4-(prop-2-yn-1-yloxy) cyclohexan-1-one (**7**)

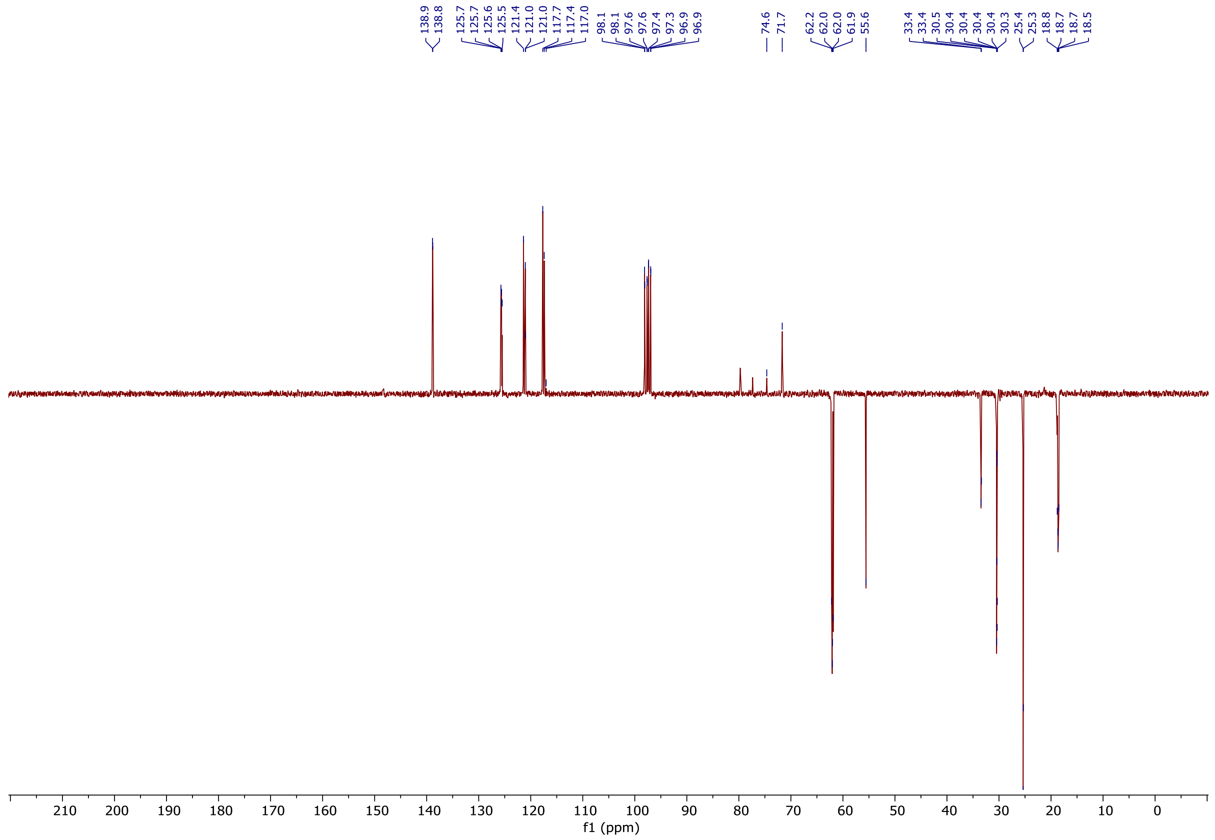

Figure S47. DEPT 135 2,6-bis((*E*)-3,4-bis((tetrahydro-2H-pyran-2-yl)oxy)benzylidene)-4-(prop-2-yn-1-yloxy) cyclohexan-1-one (**7**)

*2,6-bis((E)-3-methoxy-4-((tetrahydro-2H-pyran-2-yl)oxy)benzylidene)-4-(prop-2-yn-1-yloxy) cyclohexan-1-one (7**a**)*

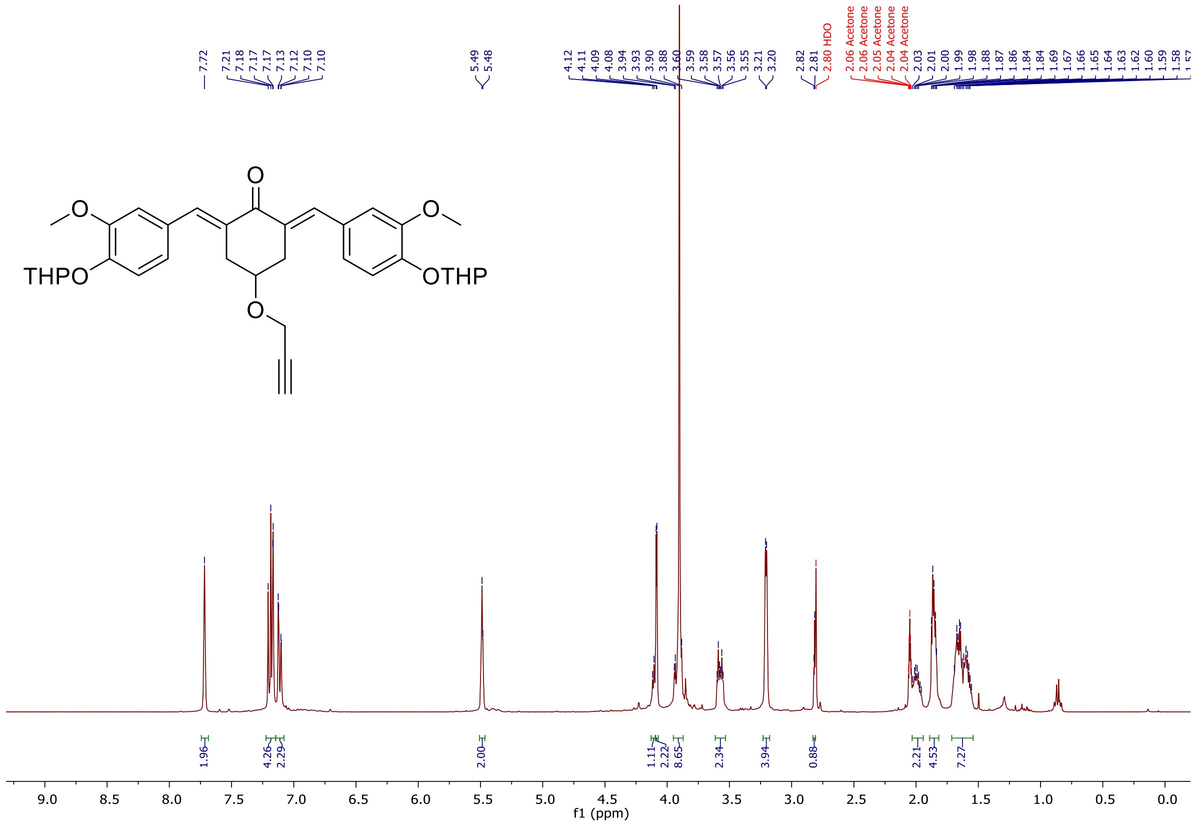

Figure S48: ^{1}H-NMR 2,6-bis((*E*)-3-methoxy-4-((tetrahydro-2*H*-pyran-2-yl)oxy)benzylidene)-4-(prop-2-yn-1-yloxy) cyclohexan-1-one (**7a**)

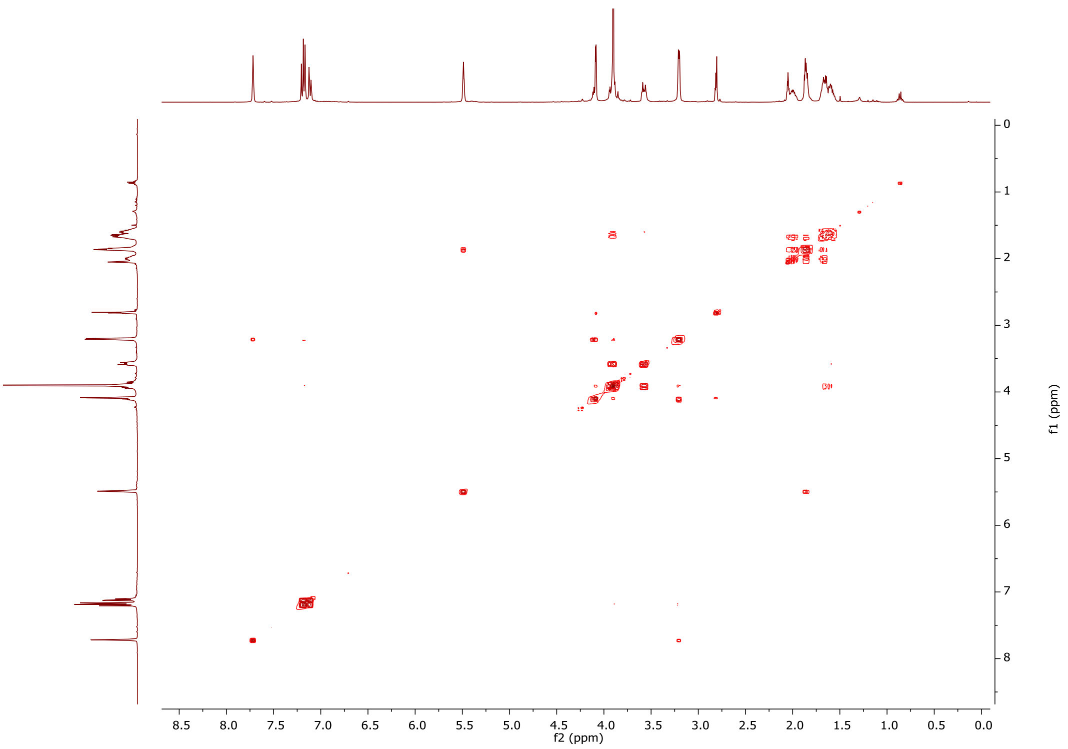

Figure S49. COSY 2,6-bis((E)-3-methoxy-4-((tetrahydro-2H-pyran-2-yl)oxy)benzylidene)-4-(prop-2-yn-1-yloxy) cyclohexan-1-one (**7a**)

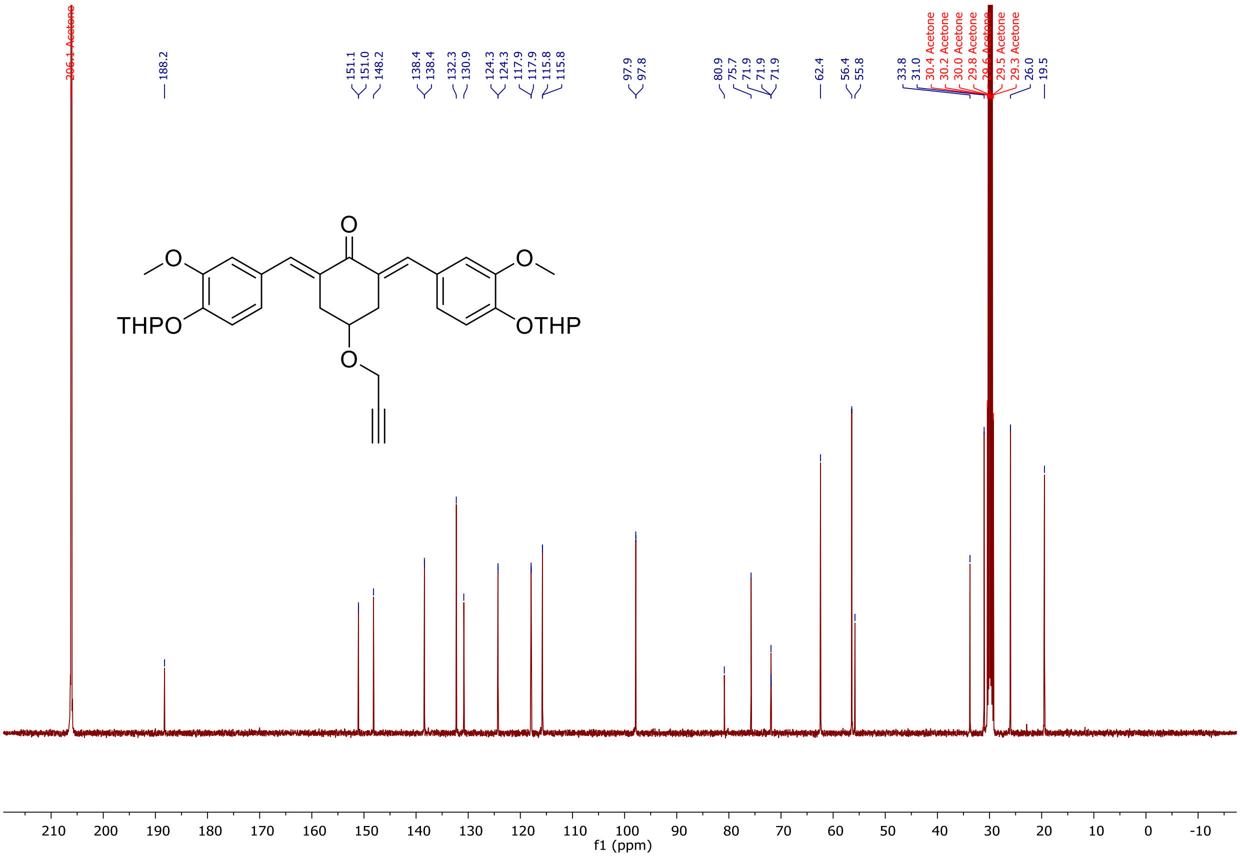

Figure S50. ^{13}C NMR 2,6-bis((E)-3-methoxy-4-((tetrahydro-2H-pyran-2-yl)oxy)benzylidene)-4-(prop-2-yn-1-yloxy) cyclohexan-1-one (**7a**)

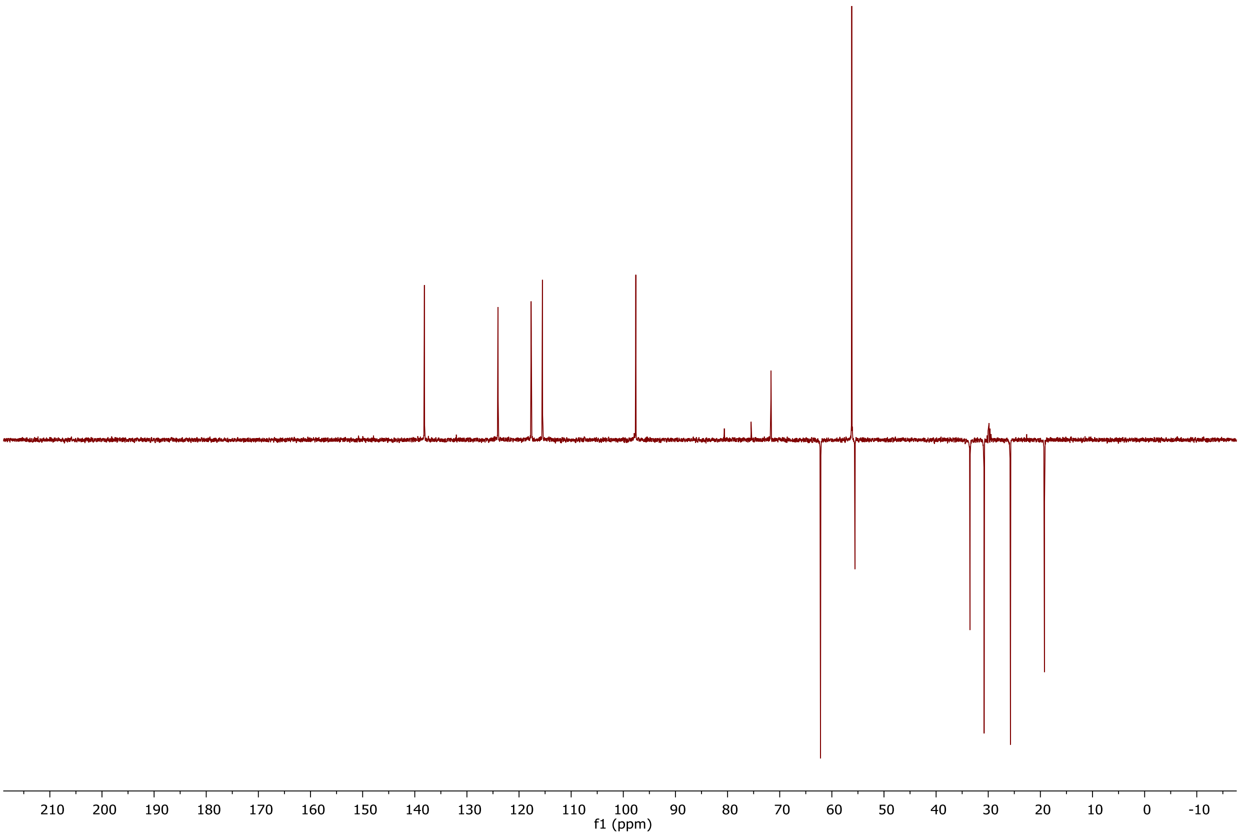

Figure S51. DEPT 135 2,6-bis((E)-3-methoxy-4-((tetrahydro-2H-pyran-2-yl)oxy)benzylidene)-4-(prop-2-yn-1-yloxy) cyclohexan-1-one (**7a**)

*2,6-bis((E)-3,4-bis((tetrahydro-2H-pyran-2-yl)oxy)benzylidene)-4-((1-(2-ethylcarboranyl)-1H-1,2,3-triazol-4-yl)methoxy)cyclohexan-1-one (**8**)*

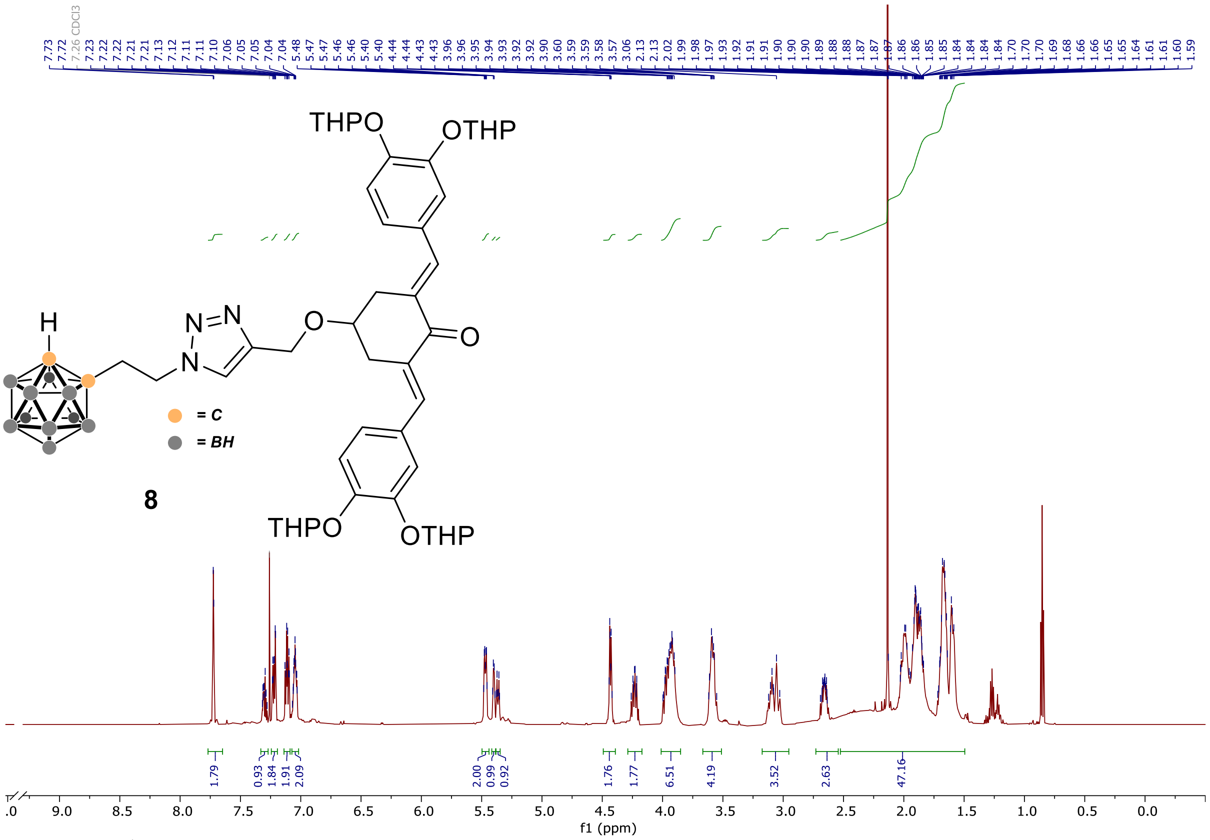

Figure S52. ^{1}H NMR 2,6-bis((E)-3,4-bis((tetrahydro-2H-pyran-2-yl)oxy)benzylidene)-4-((1-(2-ethylcarboranyl)-1H-1,2,3-triazol-4-yl)methoxy)cyclohexan-1-one (**8**)

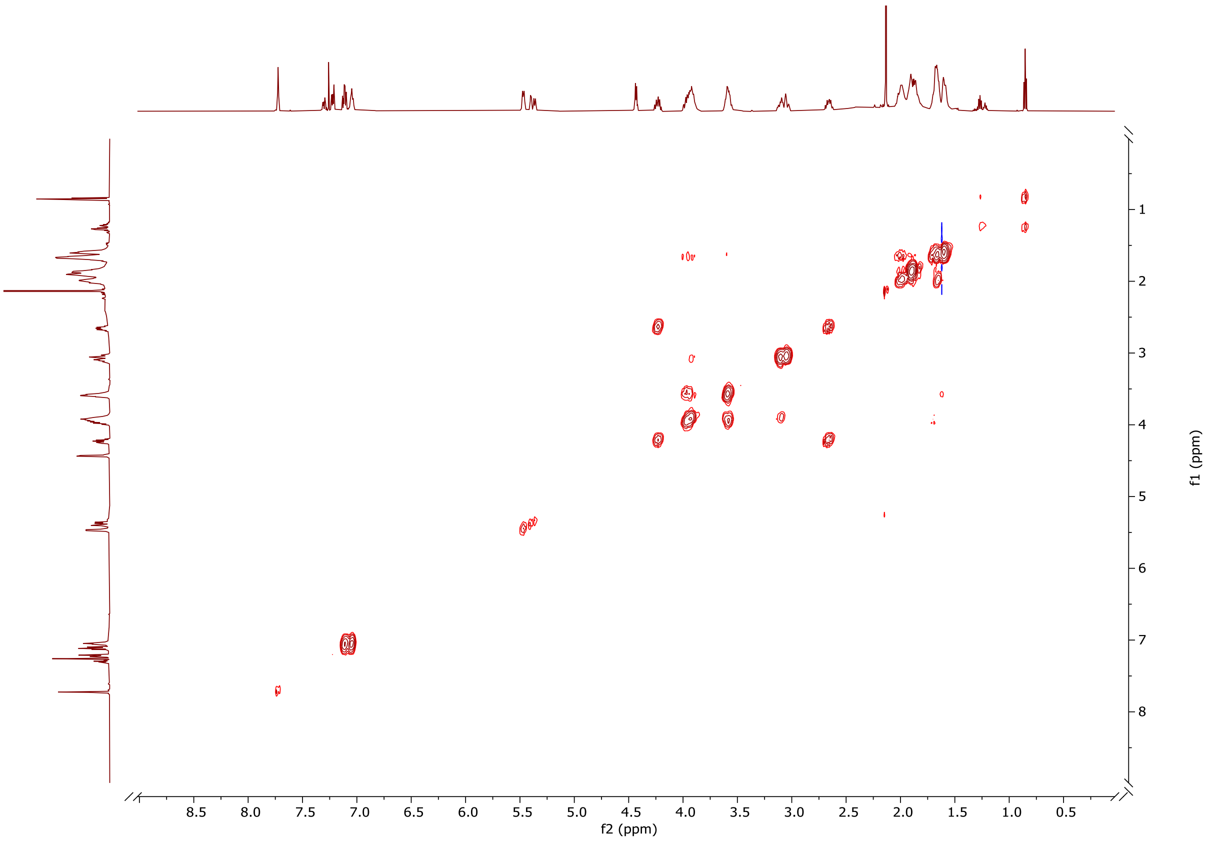

Figure S53. COSY 2,6-bis((E)-3,4-bis((tetrahydro-2H-pyran-2-yl)oxy)benzylidene)-4-((1-(2-ethylcarboranyl)-1H-1,2,3-triazol-4-yl)methoxy)cyclohexan-1-one (**8**)

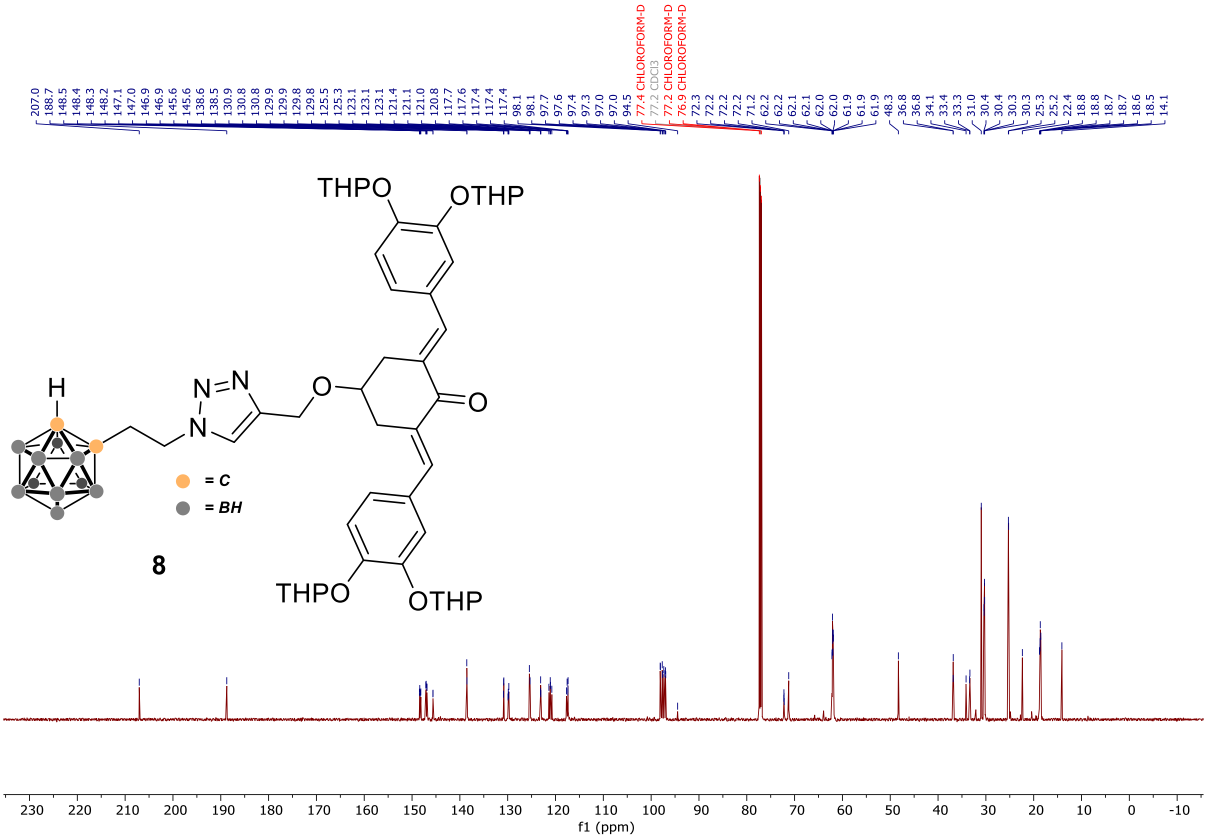

Figure S54. ^{13}C NMR 2,6-bis((E)-3,4-bis((tetrahydro-2H-pyran-2-yl)oxy)benzylidene)-4-((1-(2-ethylcarboranyl)-1H-1,2,3-triazol-4-yl)methoxy)cyclohexan-1-one (**8**)

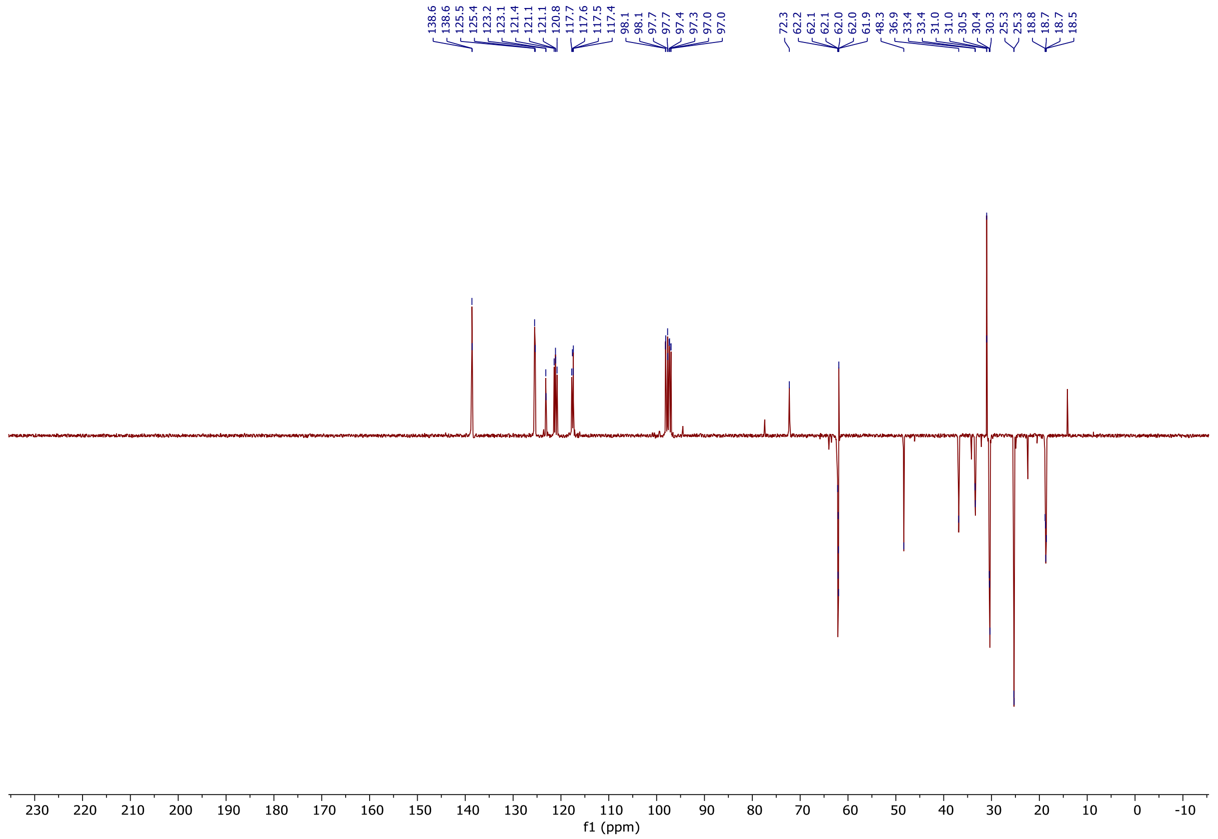

Figure S55. DEPT 135 2,6-bis((E)-3,4-bis((tetrahydro-2H-pyran-2-yl)oxy)benzylidene)-4-((1-(2-ethylcarboranyl)-1H-1,2,3-triazol-4-yl)methoxy)cyclohexan-1-one (**8**)

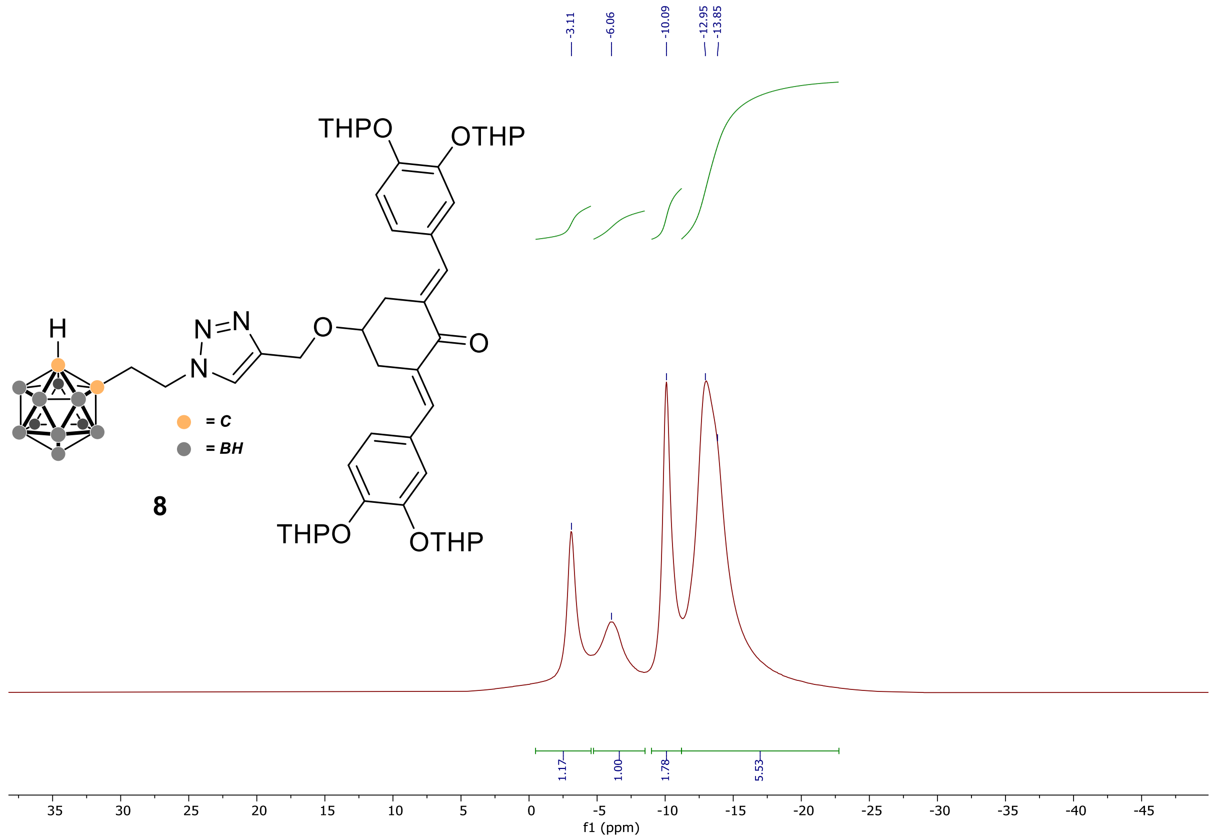

Figure S56. ^{11}B NMR 2,6-bis((E)-3,4-bis((tetrahydro-2H-pyran-2-yl)oxy)benzylidene)-4-((1-(2-ethylcarboranyl)-1H-1,2,3-triazol-4-yl)methoxy)cyclohexan-1-one (**8**)

*[10]B-enriched 2,6-bis((E)-3,4-bis((tetrahydro-2H-pyran-2-yl)oxy)benzylidene)-4-((1-(2-ethylcarboranyl)-1H-1,2,3-triazol-4-yl)methoxy)cyclohexan-1-one (**^{10}B-8**)*

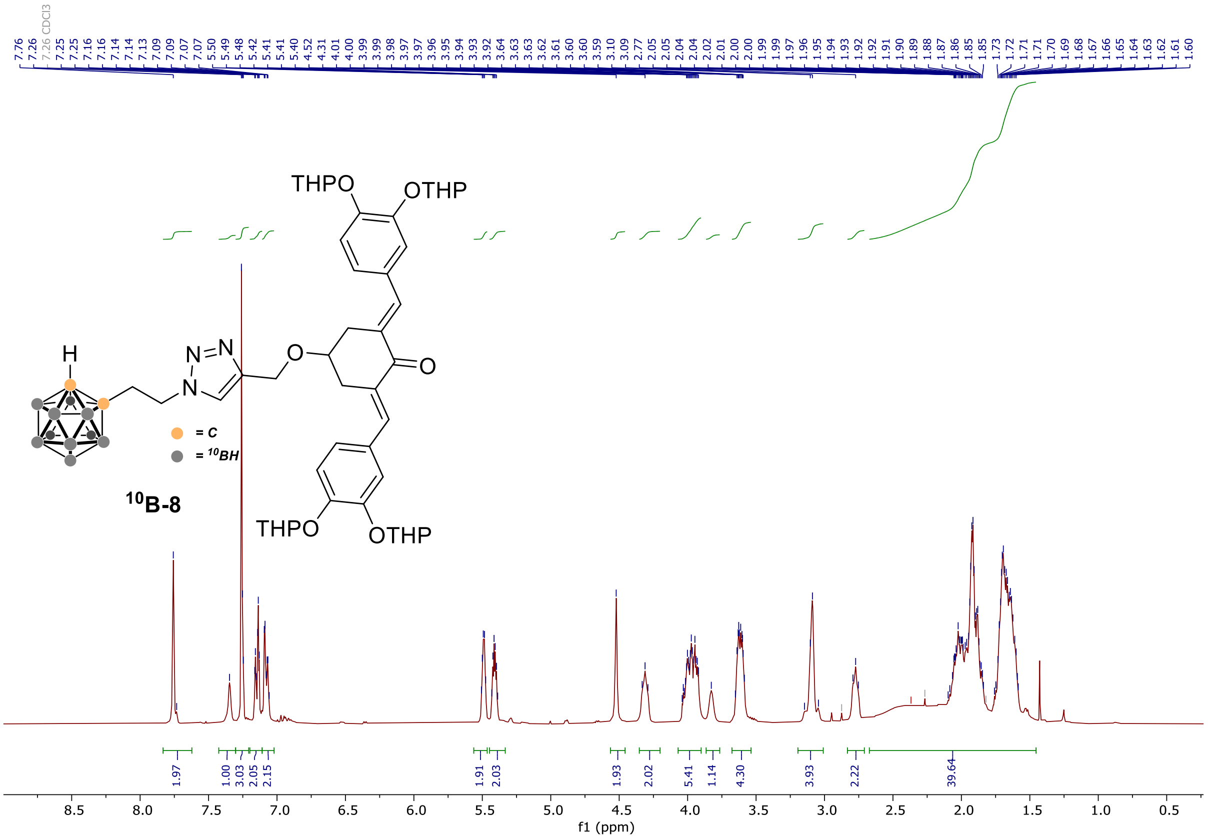

Figure S57. ^{1}H NMR ^{10}B-enriched 2,6-bis((E)-3,4-bis((tetrahydro-2H-pyran-2-yl)oxy)benzylidene)-4-((1-(2-ethylcarboranyl)-1H-1,2,3-triazol-4-yl)methoxy)cyclohexan-1-one (**^{10}B-8**)

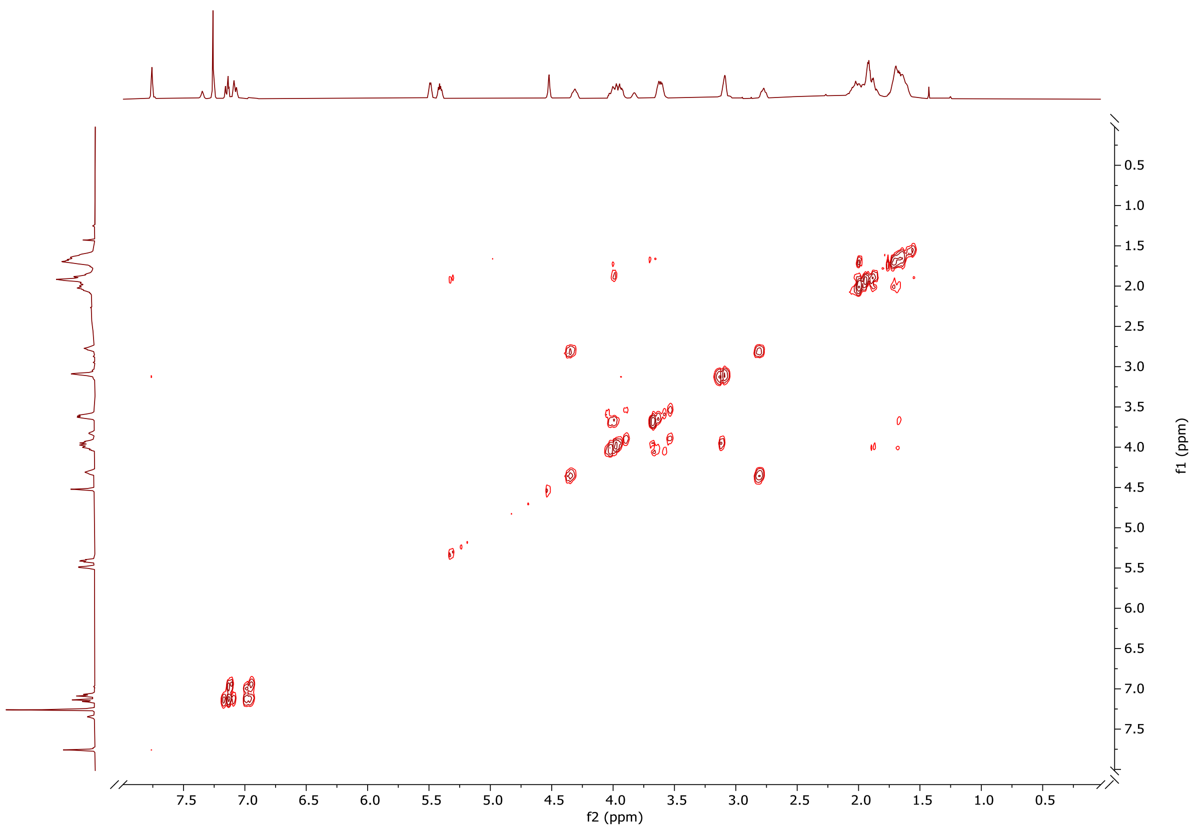

Figure S58. COSY ^{10}B-enriched 2,6-bis((E)-3,4-bis((tetrahydro-2H-pyran-2-yl)oxy)benzylidene)-4-((1-(2-ethylcarboranyl)-1H-1,2,3-triazol-4-yl)methoxy)cyclohexan-1-one (**^{10}B-8**)

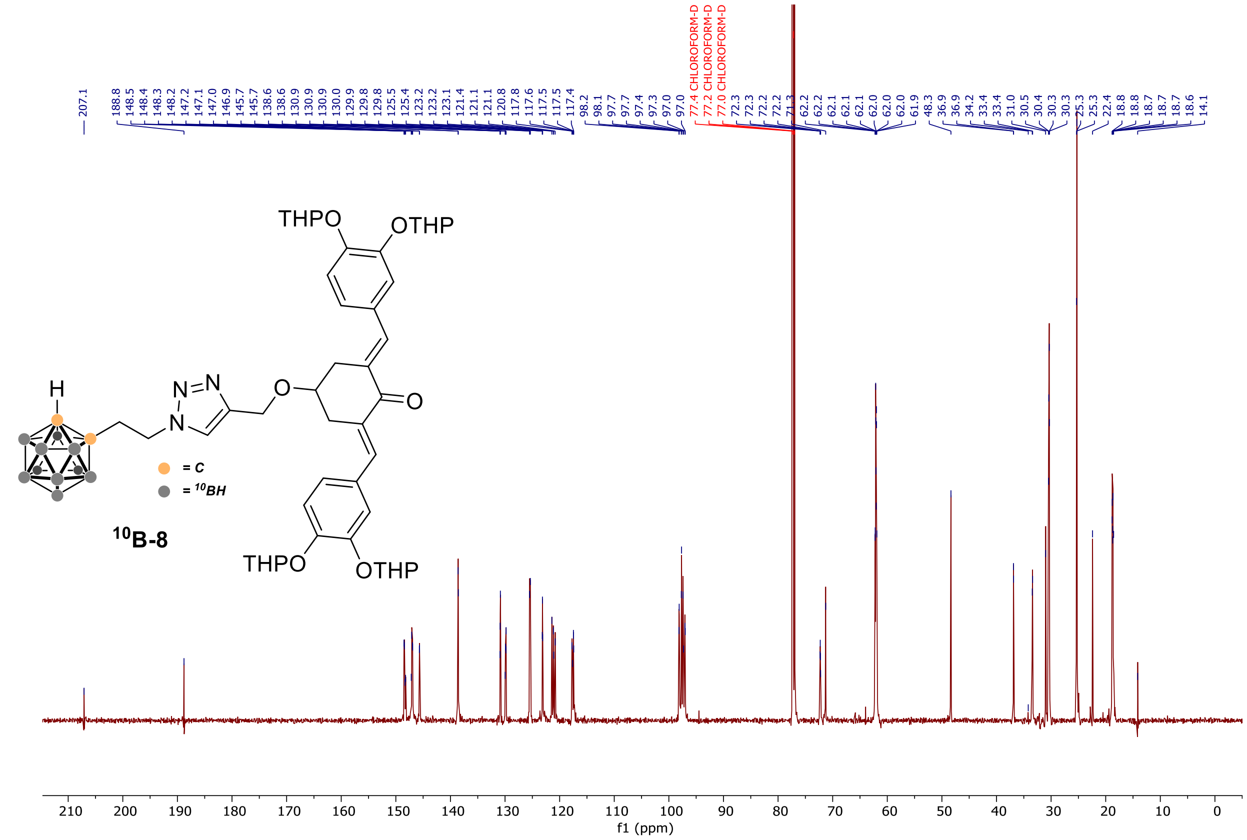

Figure S59. ^{13}C NMR ^{10}B-enriched 2,6-bis((E)-3,4-bis((tetrahydro-2H-pyran-2-yl)oxy)benzylidene)-4-((1-(2-ethylcarboranyl)-1H-1,2,3-triazol-4-yl)methoxy)cyclohexan-1-one (**^{10}B-8**)

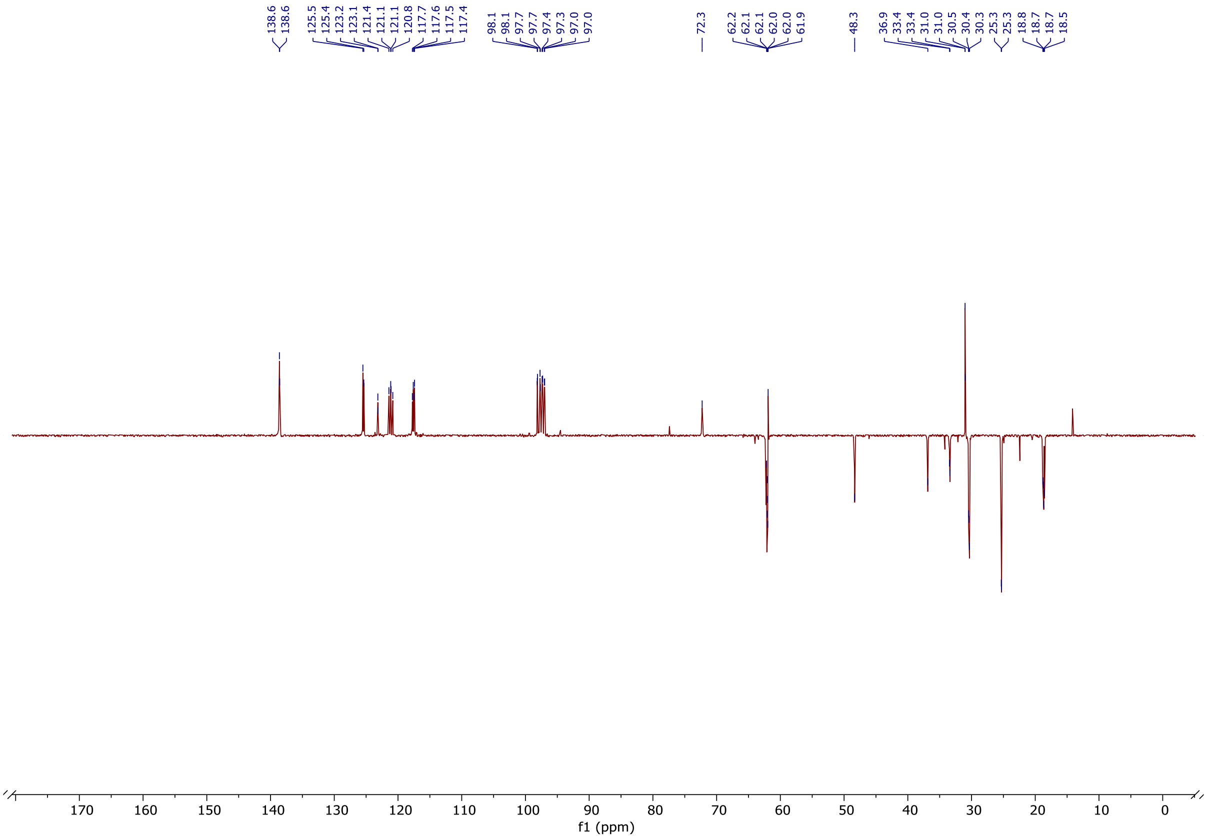

Figure S60. DEPT 135 ^{10}B-enriched 2,6-bis((E)-3,4-bis((tetrahydro-2H-pyran-2-yl)oxy)benzylidene)-4-((1-(2-ethylcarboranyl)-1H-1,2,3-triazol-4-yl)methoxy)cyclohexan-1-one (**^{10}B-8**)

*2,6-bis((E)-3-methoxy-4-(tetrahydro-2H-pyran-2-yl)oxy)benzylidene)-4-((1-(2-ethylcarboranyl)-1H-1,2,3-triazol-4-yl)methoxy)cyclohexan-1-one (**8a**)*

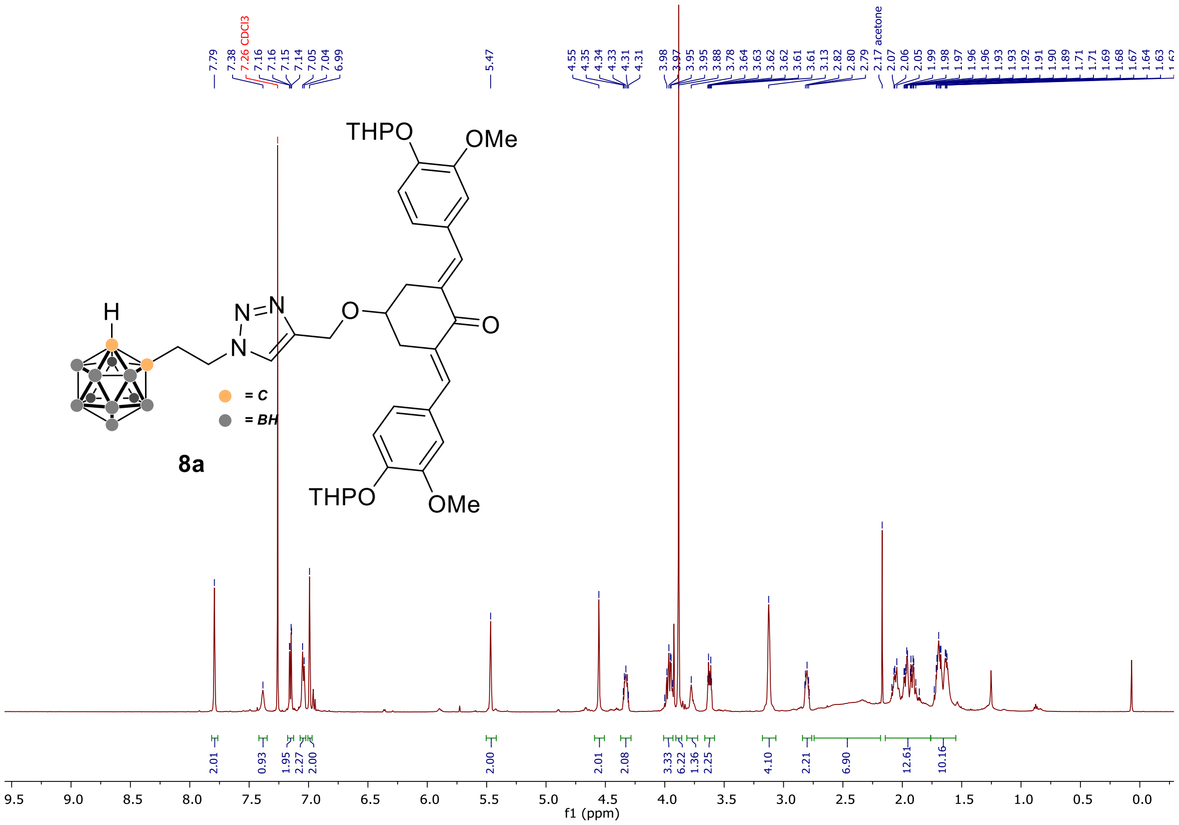

Figure S61. ^{1}H NMR 2,6-bis((E)-3-methoxy-4-(tetrahydro-2H-pyran-2-yl)oxy)benzylidene)-4-((1-(2-ethylcarboranyl)-1H-1,2,3-triazol-4-yl)methoxy)cyclohexan-1-one (**8a**)

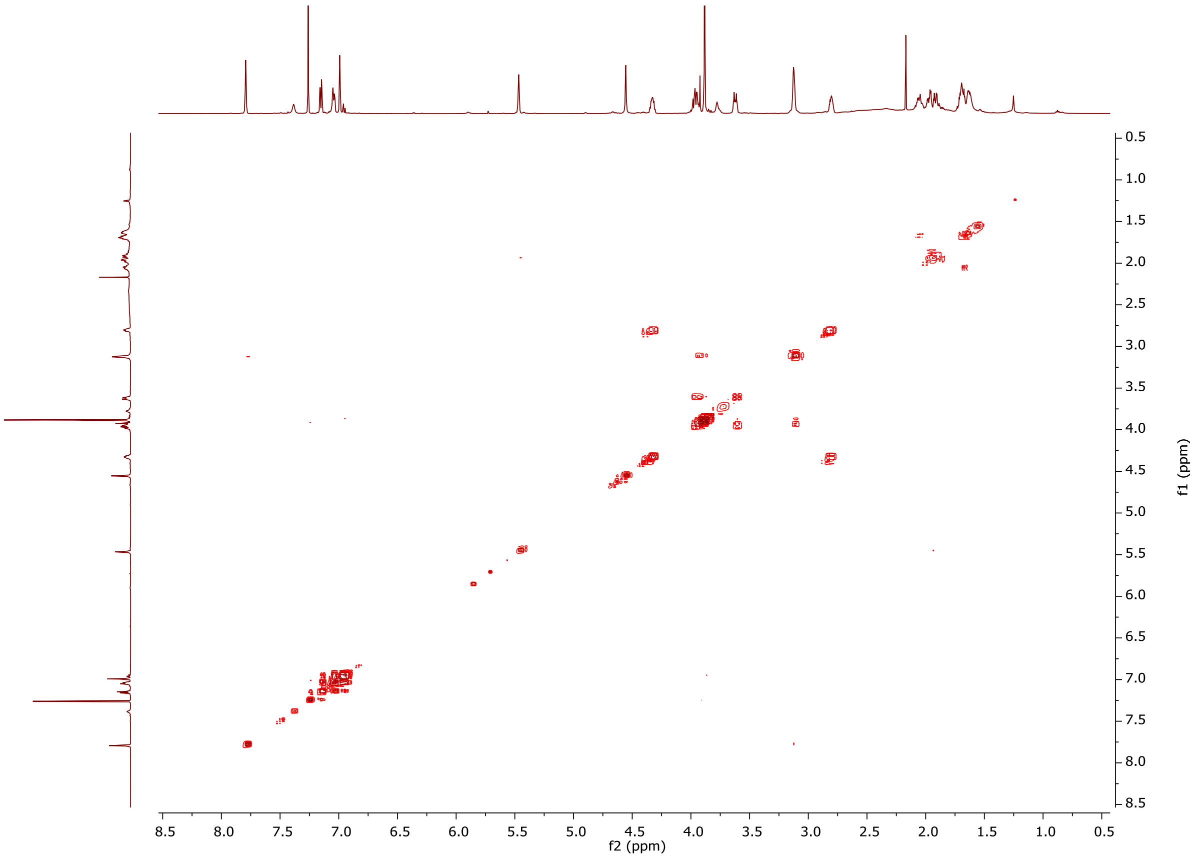

Figure S62. COSY 2,6-bis((E)-3-methoxy-4-(tetrahydro-2H-pyran-2-yl)oxy)benzylidene)-4-((1-(2-ethylcarboranyl)-1H-1,2,3-triazol-4-yl)methoxy)cyclohexan-1-one (**8a**)

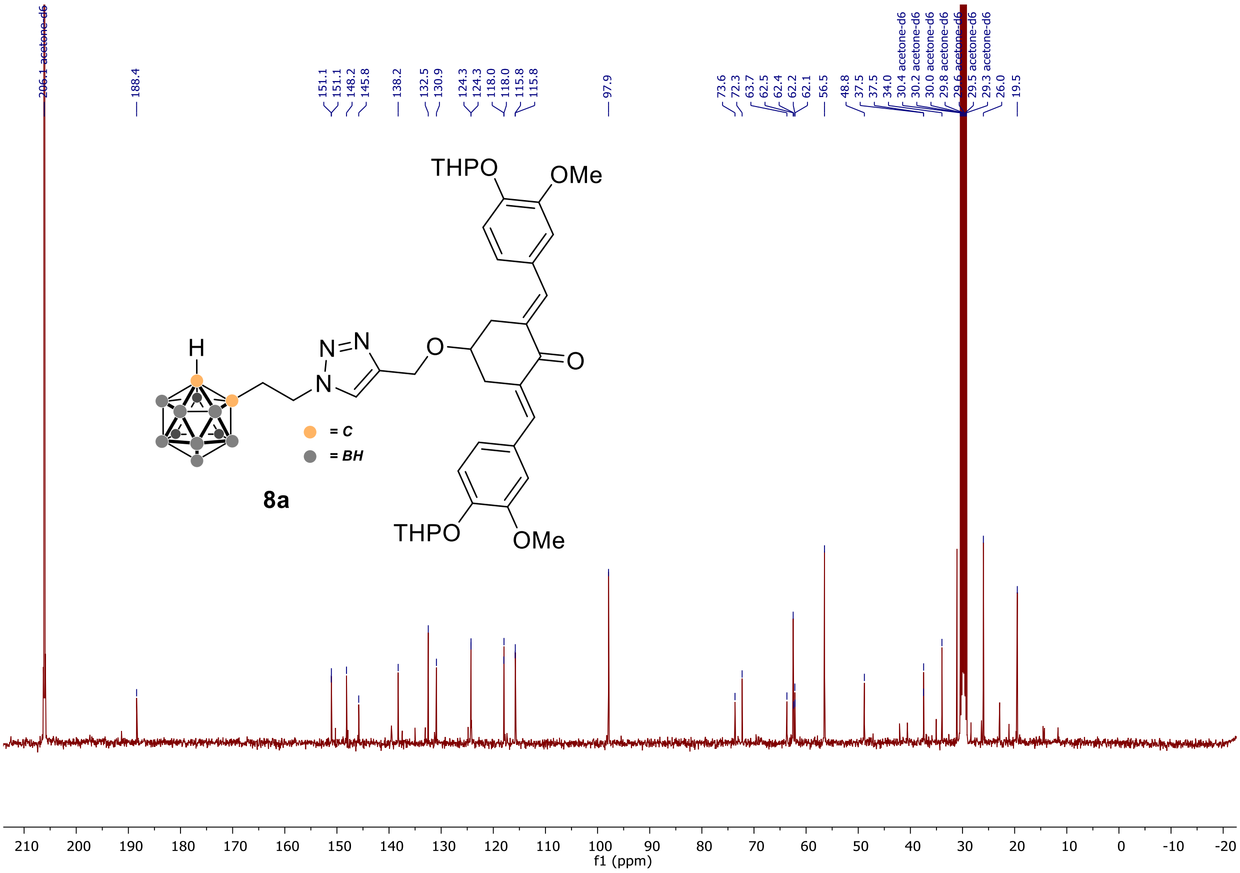

Figure S63. ^{13}C NMR 2,6-bis((E)-3-methoxy-4-(tetrahydro-2H-pyran-2-yl)oxy)benzylidene)-4-((1-(2-ethylcarboranyl)-1H-1,2,3-triazol-4-yl)methoxy)cyclohexan-1-one (**8a**)

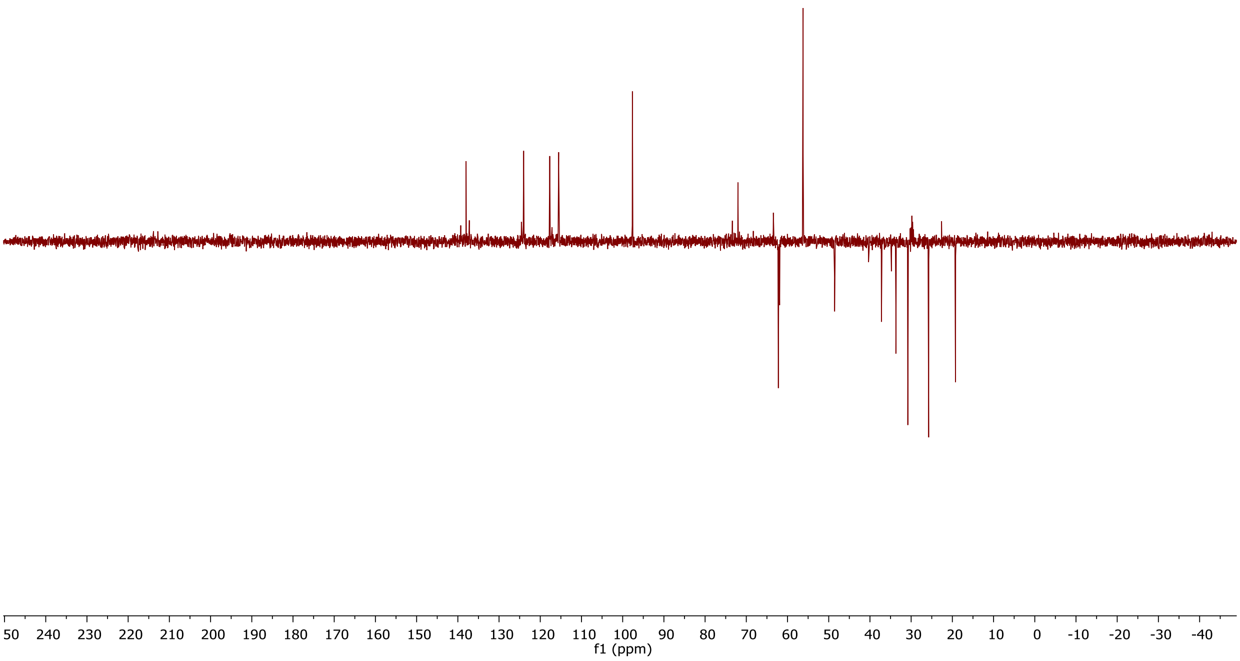

Figure S64. DEPT 135 2,6-bis((E)-3-methoxy-4-(tetrahydro-2H-pyran-2-yl)oxy)benzylidene)-4-((1-(2-ethylcarboranyl)-1H-1,2,3-triazol-4-yl)methoxy)cyclohexan-1-one (**8a**)

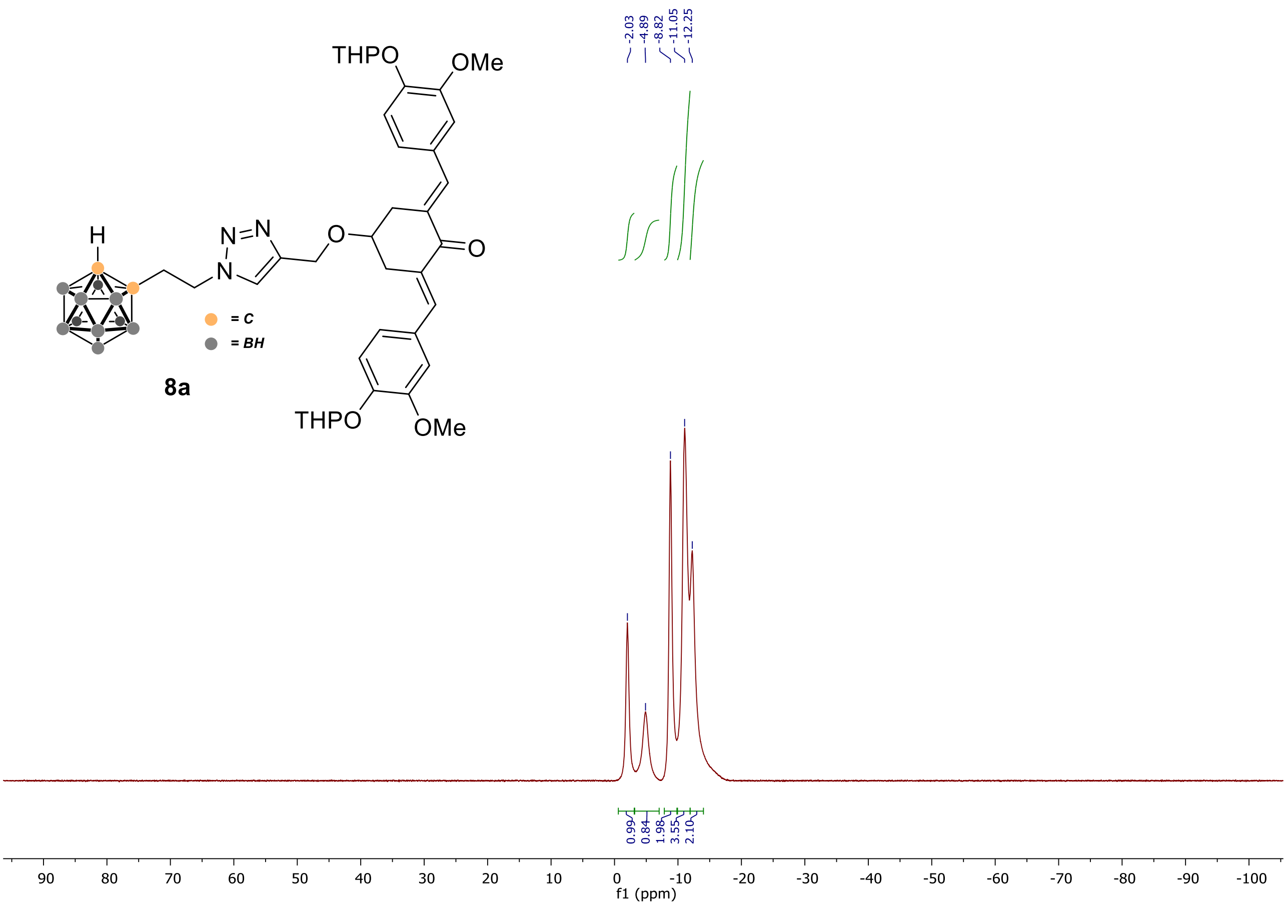

Figure S65. ^{11}B NMR 2,6-bis((E)-3-methoxy-4-(tetrahydro-2H-pyran-2-yl)oxy)benzylidene)-4-((1-(2-ethylcarboranyl)-1H-1,2,3-triazol-4-yl)methoxy)cyclohexan-1-one (**8a**)

*2,6-bis((E)-3,4-bis((tetrahydroxy)benzylidene)-4-((1-(2-ethylcarboranyl)-1H-1,2,3-triazol-4-yl)methoxy)cyclohexan-1-one (**9**)*

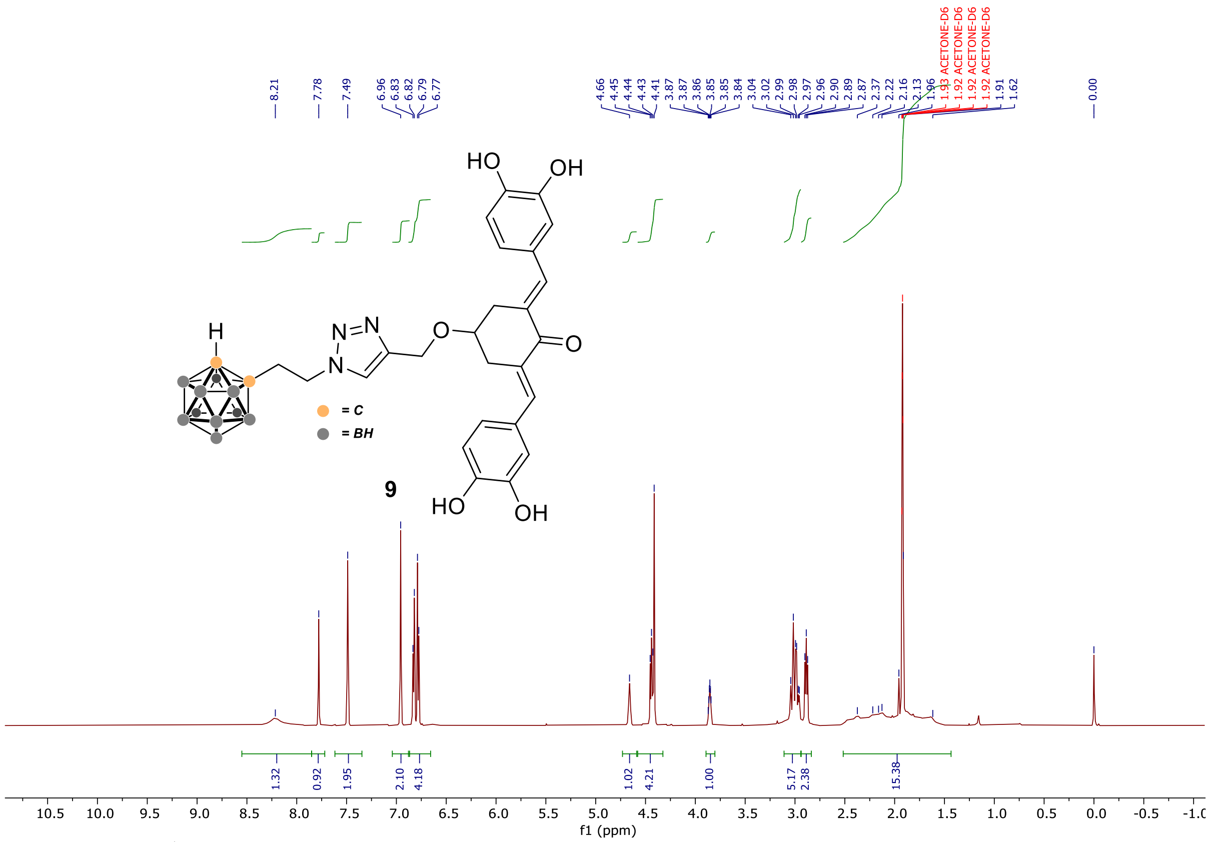

Figure S66. ^{1}H NMR 2,6-bis((E)-3,4-bis((tetrahydroxy)benzylidene)-4-((1-(2-ethylcarboranyl)-1H-1,2,3-triazol-4-yl)methoxy)cyclohexan-1-one (**9**)

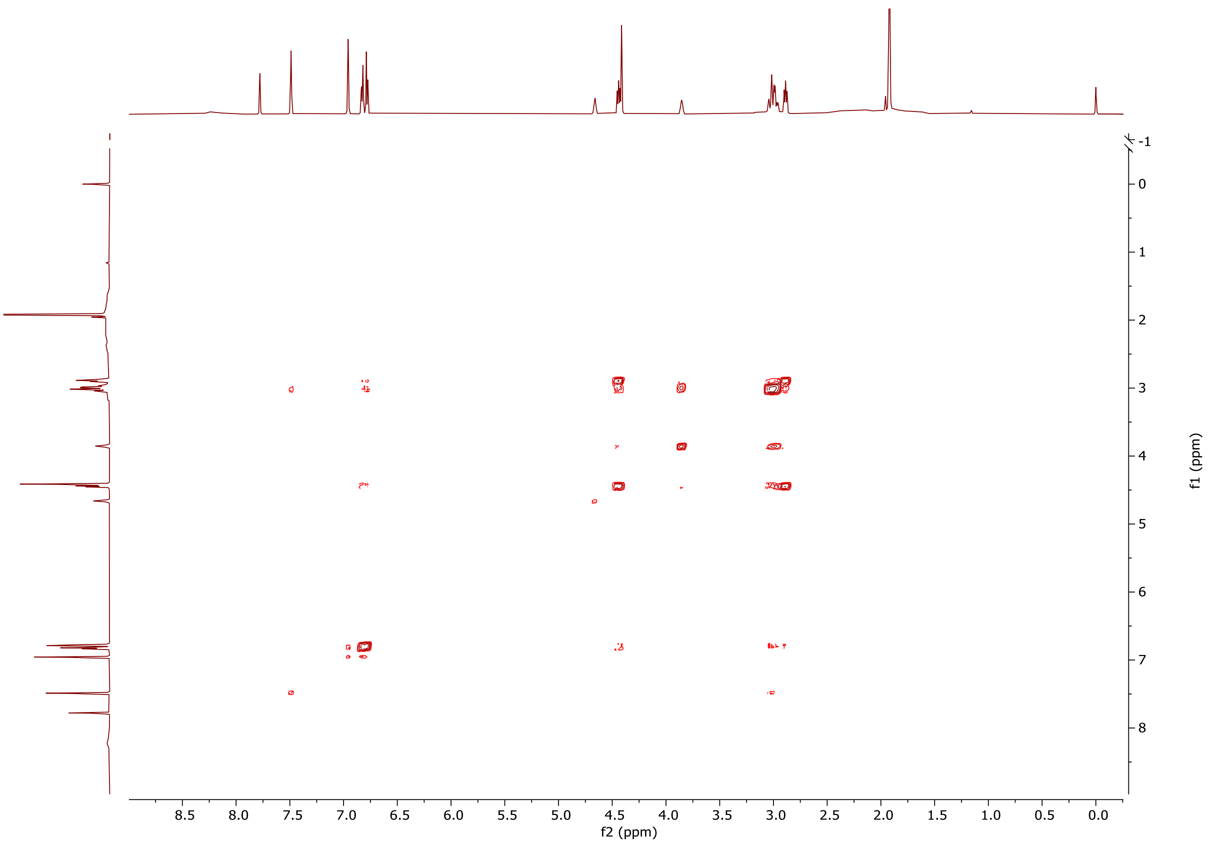

Figure S67. COSY 2,6-bis((E)-3,4-bis((tetrahydroxy)benzylidene)-4-((1-(2-ethylcarboranyl)-1H-1,2,3-triazol-4-yl)methoxy)cyclohexan-1-one (**9**)

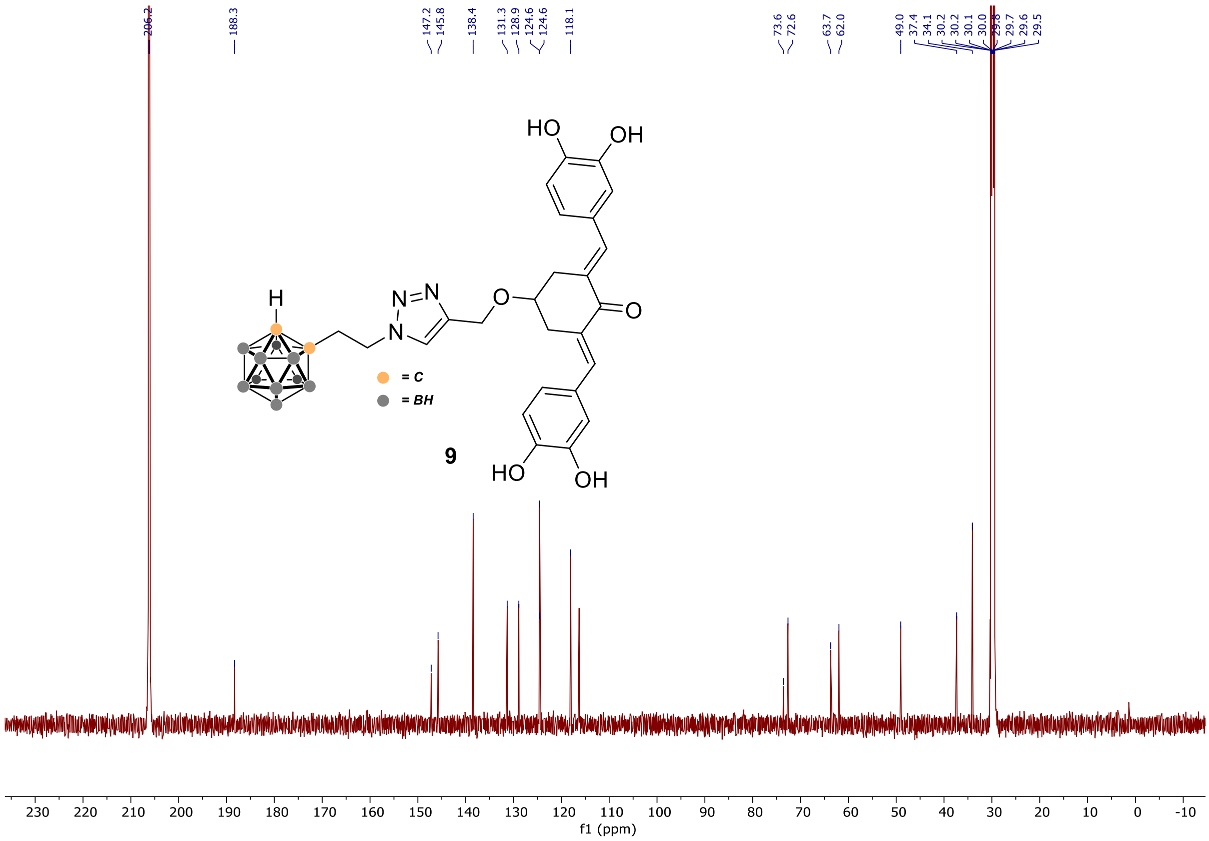

Figure S68. ^{13}C NMR 2,6-bis((E)-3,4-bis((tetrahydroxy)benzylidene)-4-((1-(2-ethylcarboranyl)-1H-1,2,3-triazol-4-yl)methoxy)cyclohexan-1-one (**9**)

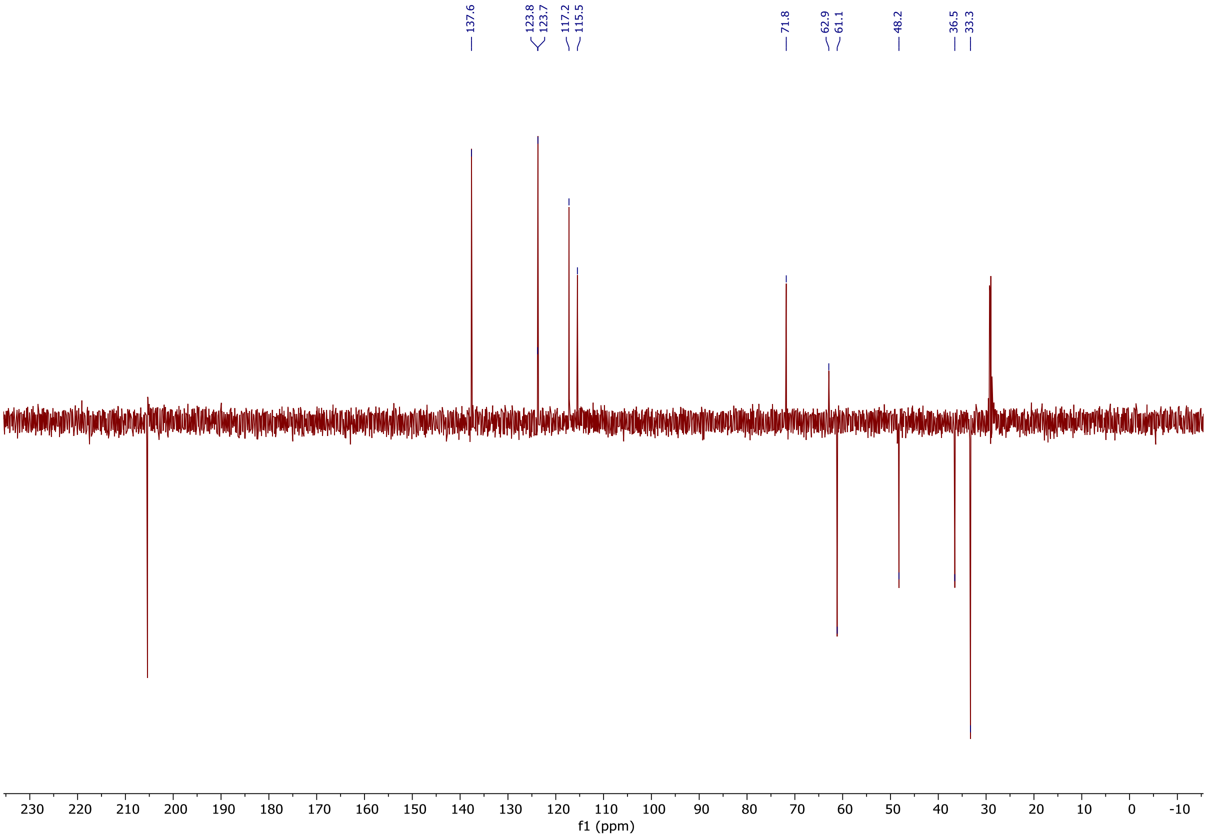

Figure S69. DEPT 135 2,6-bis((E)-3,4-bis((tetrahydroxy)benzylidene)-4-((1-(2-ethylcarboranyl)-1H-1,2,3-triazol-4-yl)methoxy)cyclohexan-1-one (**9**)

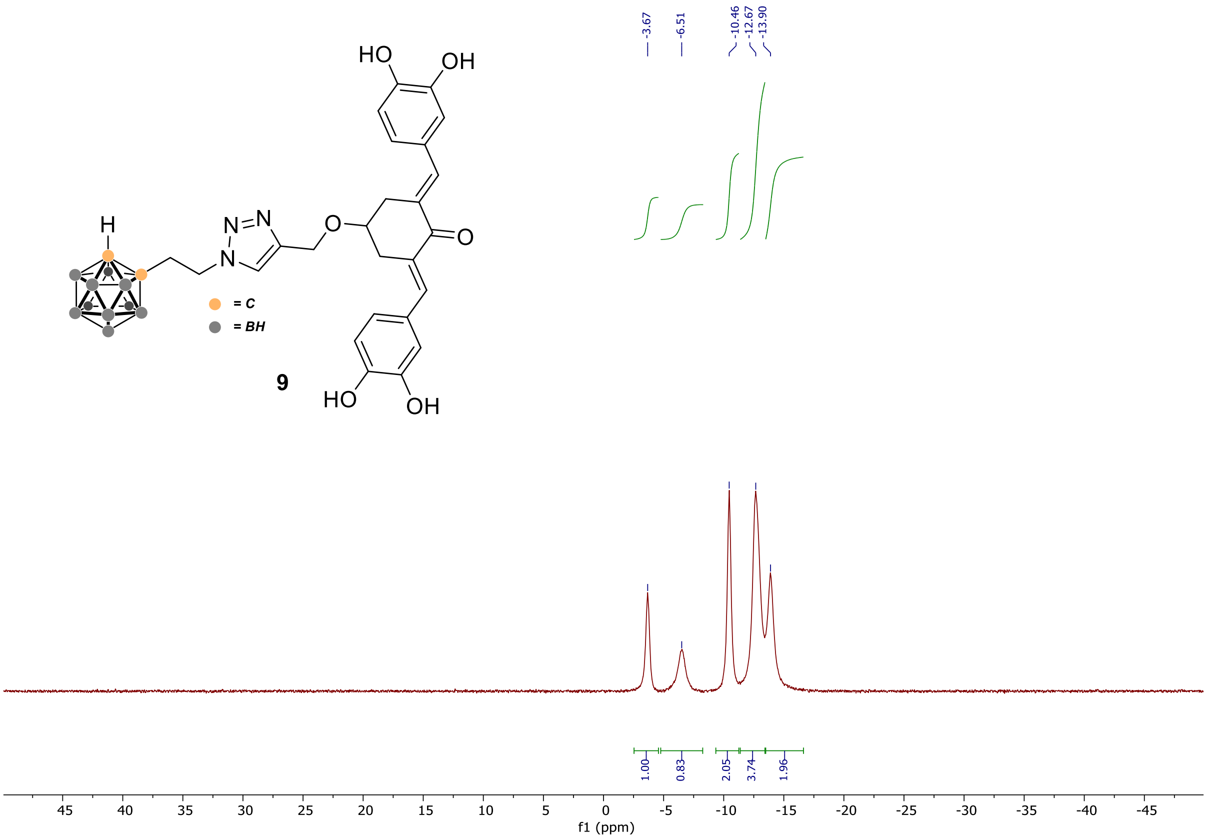

Figure S70. ^{11}B NMR 2,6-bis((E)-3,4-bis((tetrahydroxy)benzylidene)-4-((1-(2-ethylcarboranyl)-1H-1,2,3-triazol-4-yl)methoxy)cyclohexan-1-one (**9**)

*[10]B-enriched 2,6-bis((E)-3,4-bis((tetrahydroxy)benzylidene)-4-((1-(2-ethylcarboranyl)-1H-1,2,3-triazol-4-yl)methoxy)cyclohexan-1-one (**[10]B-9**)*

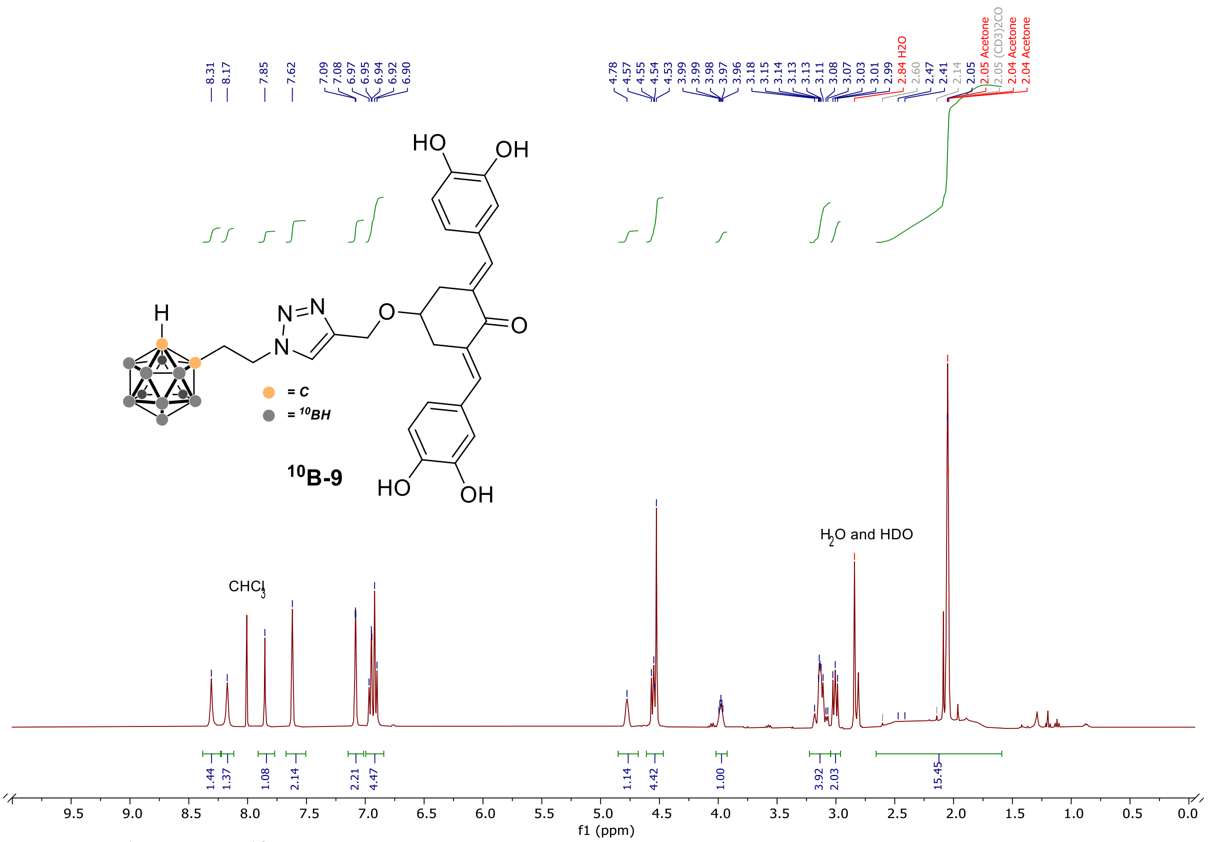

Figure S7*1*. ^{1}H NMR ^{10}B-enriched 2,6-bis((E)-3,4-bis((tetrahydroxy)benzylidene)-4-((1-(2-ethylcarboranyl)-1H-1,2,3-triazol-4-yl)methoxy)cyclohexan-1-one (**[10]B-9**)

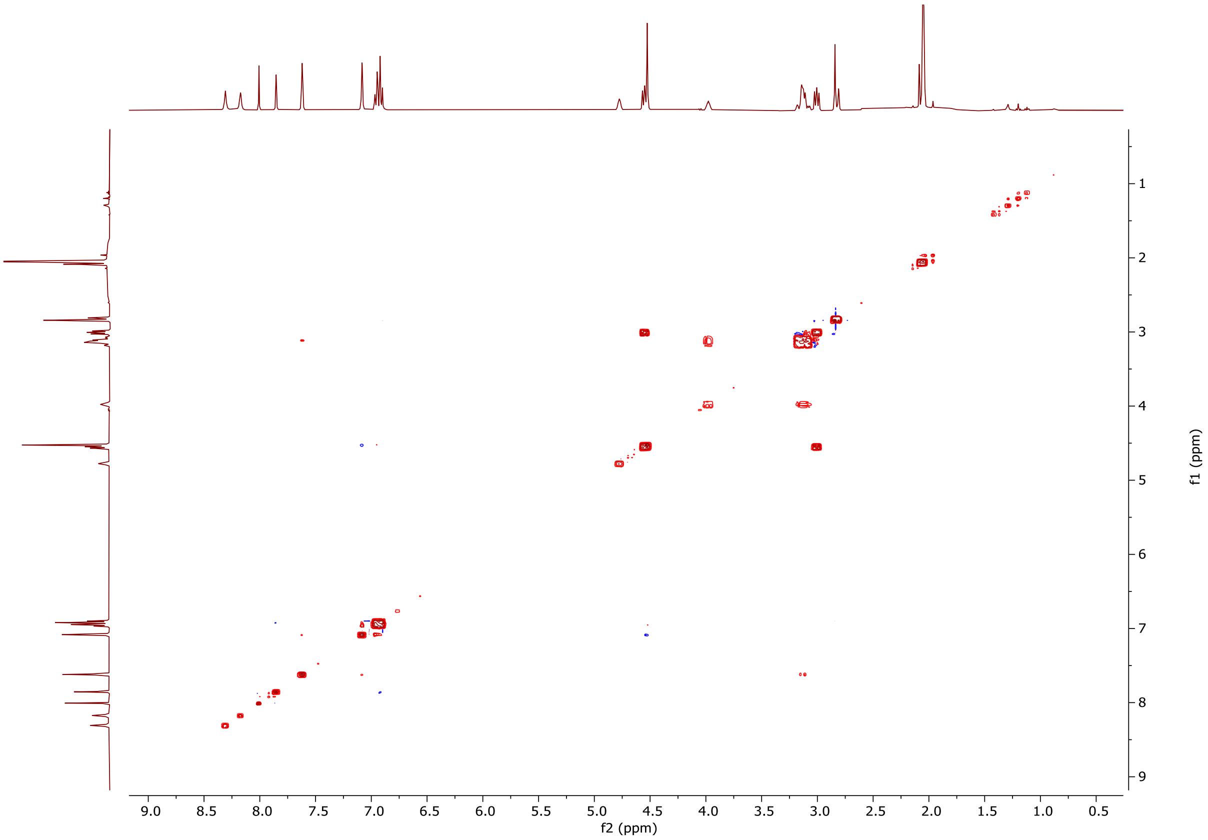

Figure S72. COSY ^{10}B-enriched 2,6-bis((E)-3,4-bis((tetrahydroxy)benzylidene)-4-((1-(2-ethylcarboranyl)-1H-1,2,3-triazol-4-yl)methoxy)cyclohexan-1-one (^{10}**B-9**)

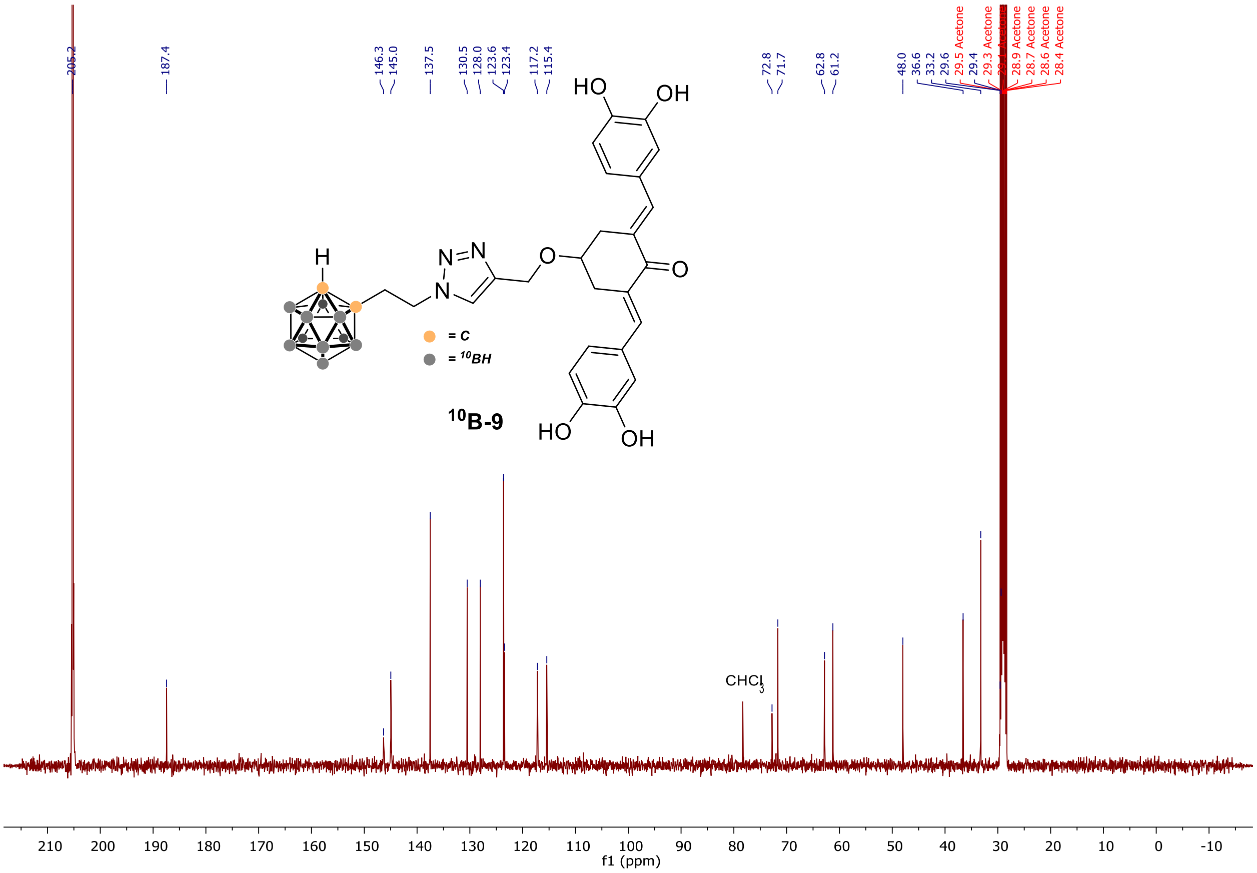

Figure S73. ^{13}C NMR ^{10}B-enriched 2,6-bis((E)-3,4-bis((tetrahydroxy)benzylidene)-4-((1-(2-ethylcarboranyl)-1H-1,2,3-triazol-4-yl)methoxy)cyclohexan-1-one (**^{10}B-9**)

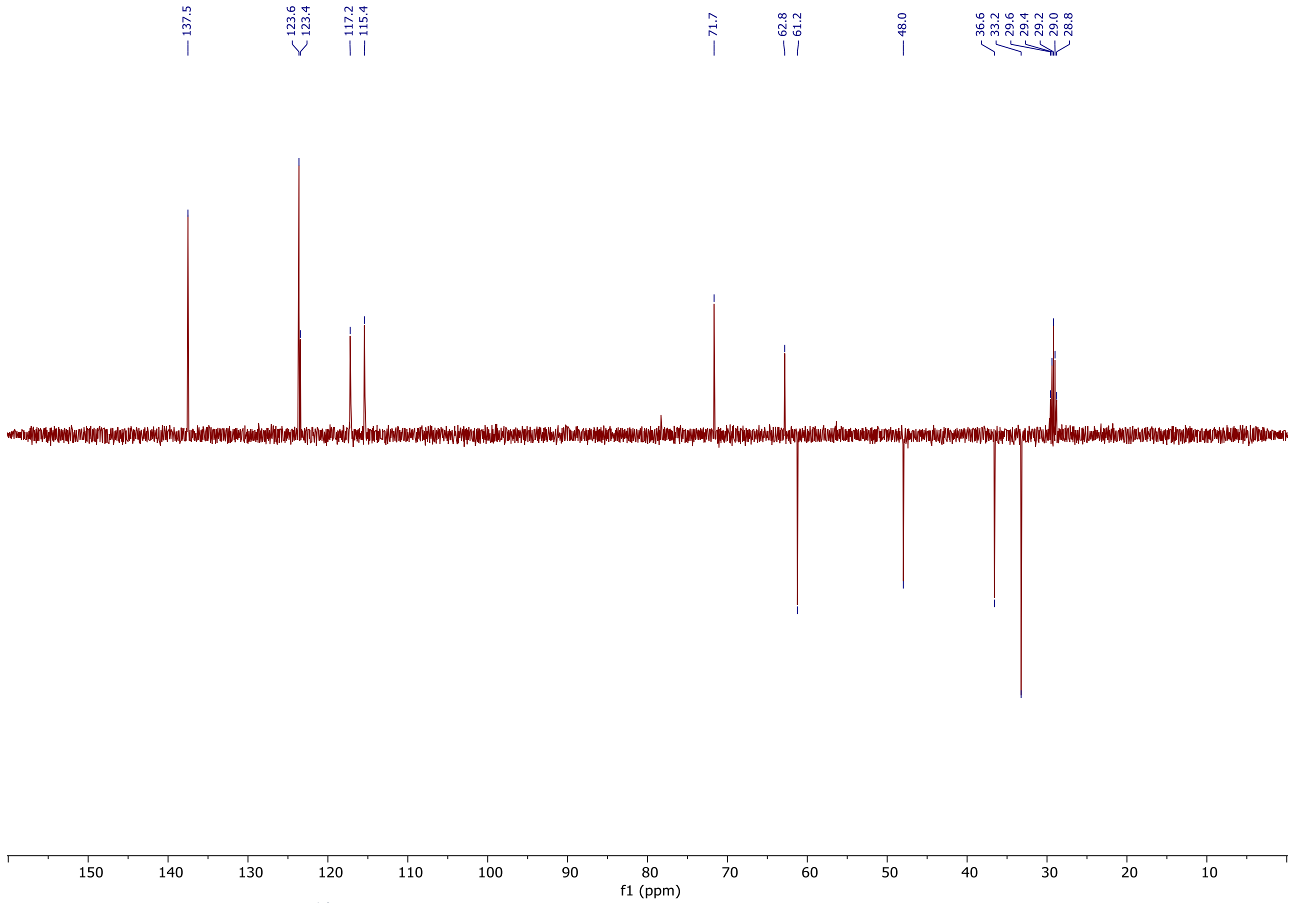

Figure S74. DEPT 135 ^{10}B-enriched 2,6-bis((E)-3,4-bis((tetrahydroxy)benzylidene)-4-((1-(2-ethylcarboranyl)-1H-1,2,3-triazol-4-yl)methoxy)cyclohexan-1-one (**^{10}B-9**)

*2,6-bis((E)-3-methoxy-4-(tetrahydroxy)benzylidene)-4-((1-(2-ethylcarboranyl)-1H-1,2,3-triazol-4-yl)methoxy)cyclohexan-1-on (**9a**)*

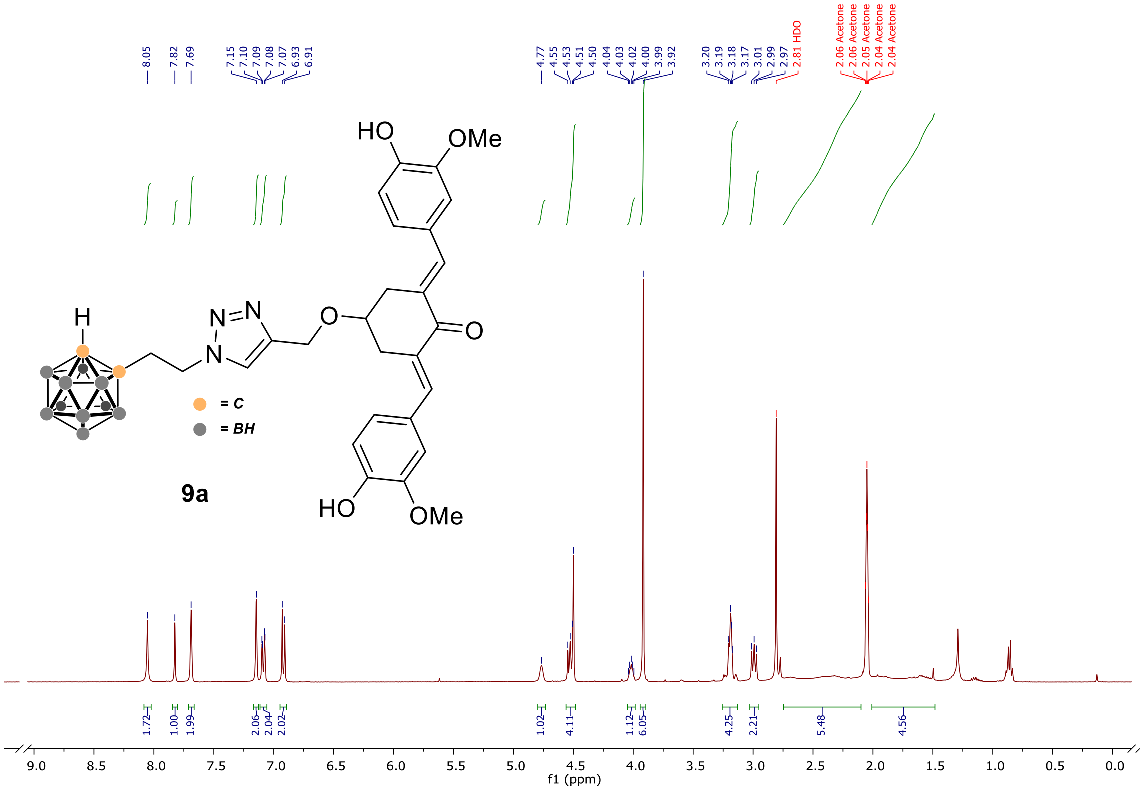

Figure S75. ^{1}H NMR 2,6-bis((E)-3-methoxy-4-(tetrahydroxy)benzylidene)-4-((1-(2-ethylcarboranyl)-1H-1,2,3-triazol-4-yl)methoxy)cyclohexan-1-on (**9a**)

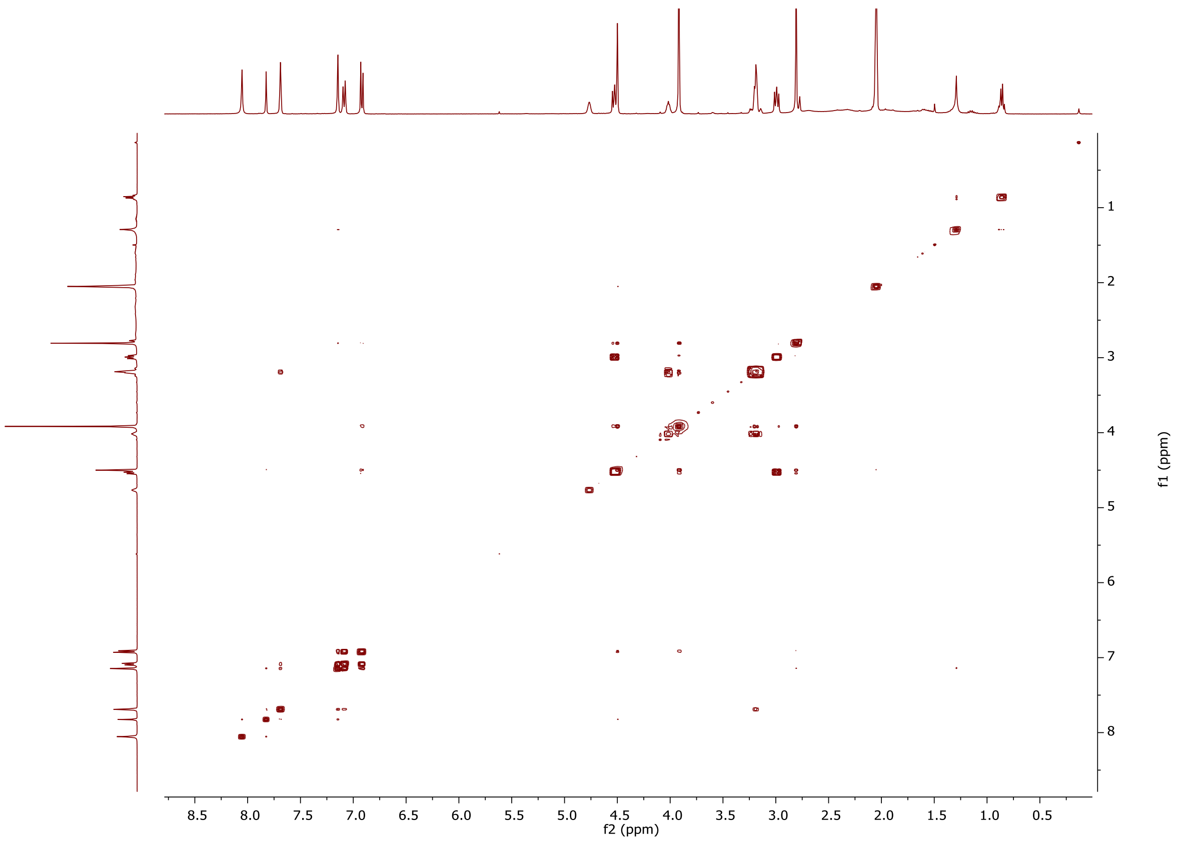

Figure S76. COSY 2,6-bis((E)-3-methoxy-4-(tetrahydroxy)benzylidene)-4-((1-(2-ethylcarboranyl)-1H-1,2,3-triazol-4-yl)methoxy)cyclohexan-1-on (**9a**)

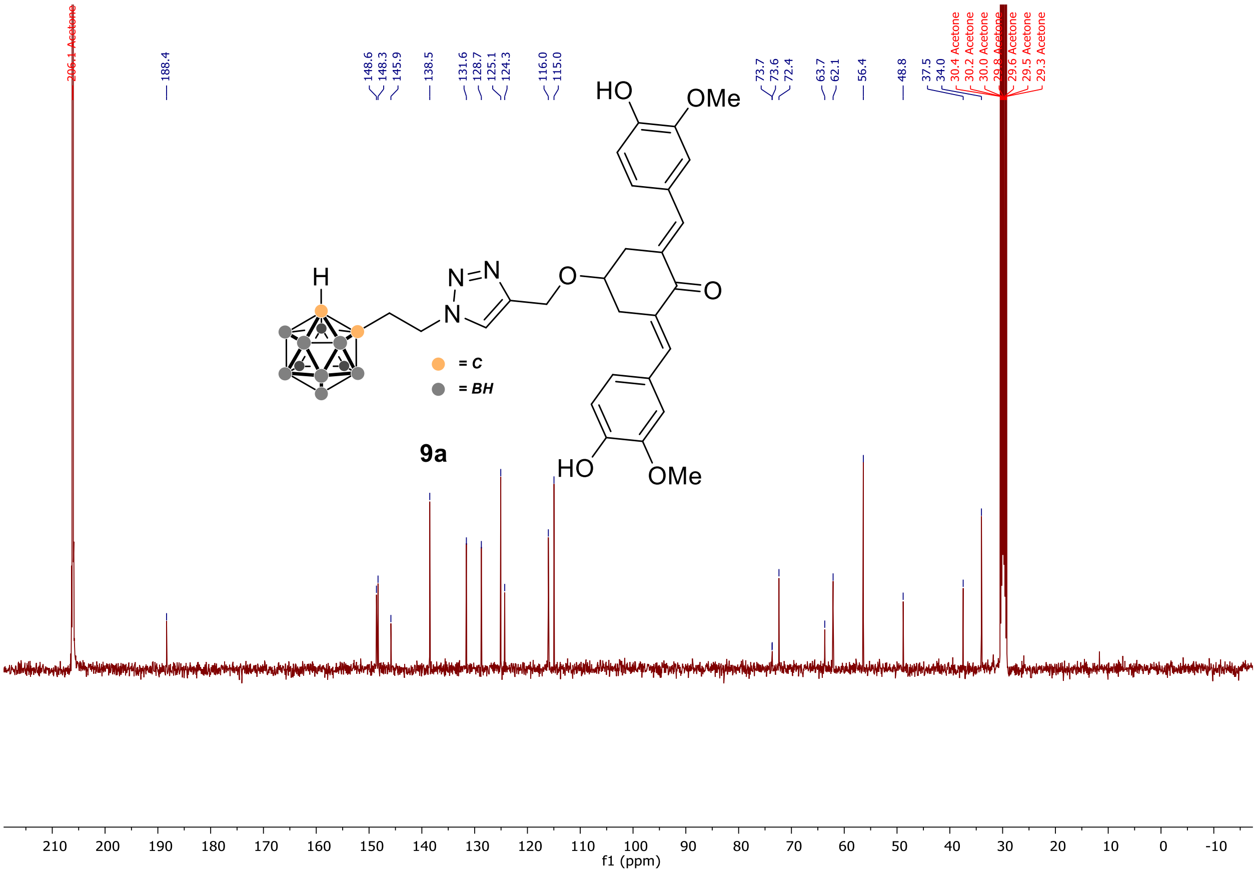

Figure S77. ^{13}C NMR 2,6-bis((E)-3-methoxy-4-(tetrahydroxy)benzylidene)-4-((1-(2-ethylcarboranyl)-1H-1,2,3-triazol-4-yl)methoxy)cyclohexan-1-on (**9a**)

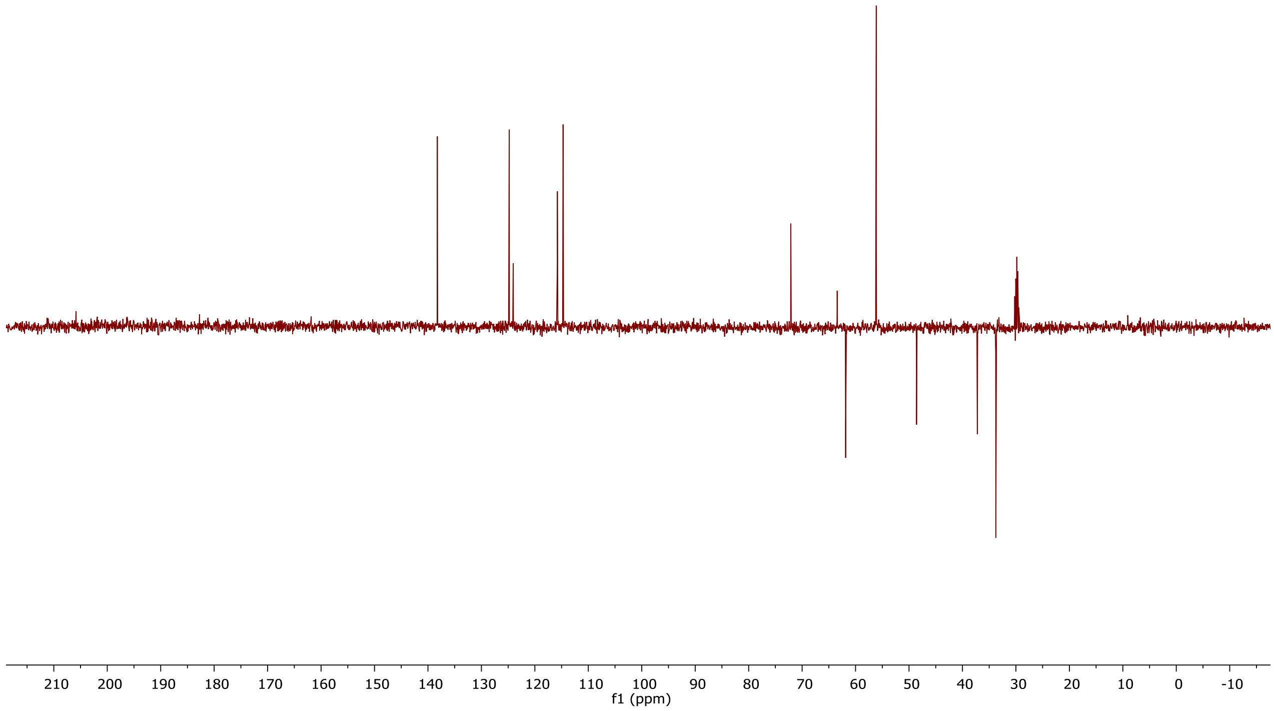

Figure S78. DEPT 135 2,6-bis((E)-3-methoxy-4-(tetrahydroxy)benzylidene)-4-((1-(2-ethylcarboranyl)-1H-1,2,3-triazol-4-yl)methoxy)cyclohexan-1-on (**9a**)

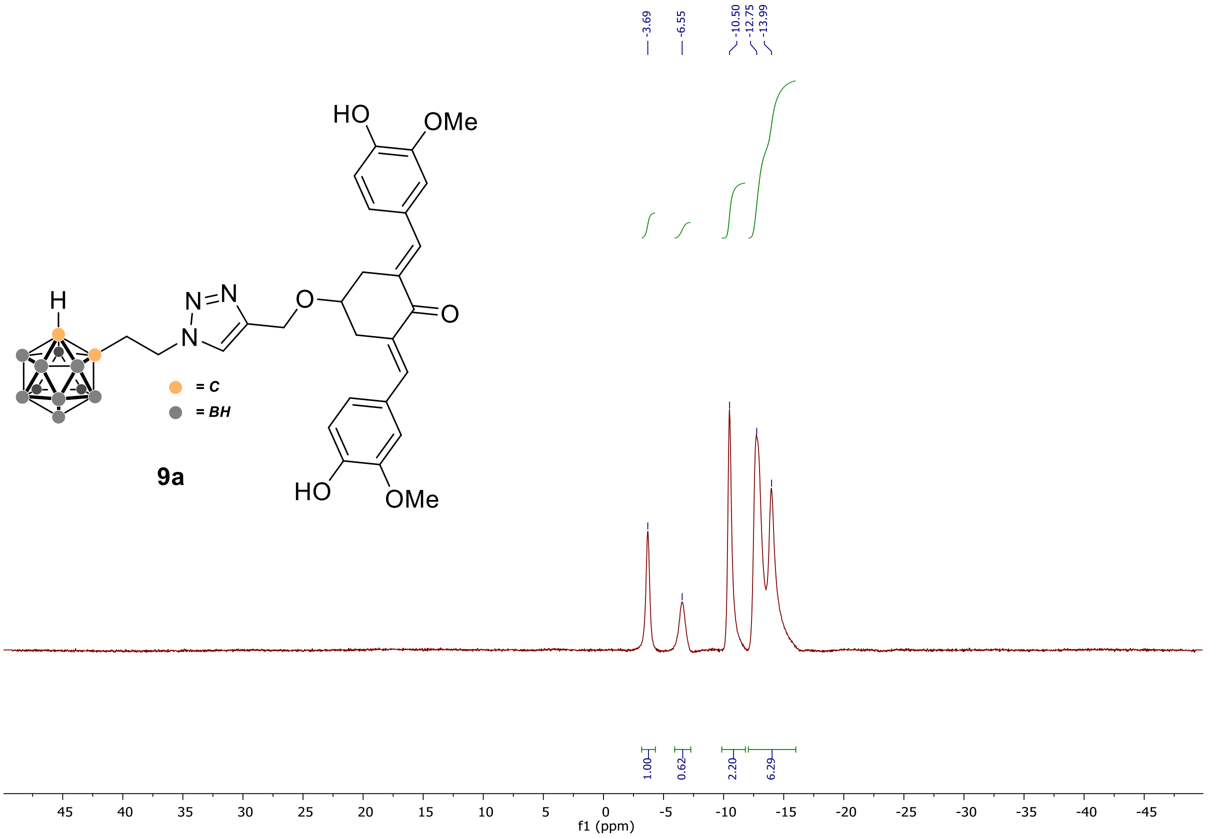

Figure S79. ^{11}B NMR 2,6-bis((E)-3-methoxy-4-(tetrahydroxy)benzylidene)-4-((1-(2-ethylcarboranyl)-1H-1,2,3-triazol-4-yl)methoxy)cyclohexan-1-on (**9a**)

*2,6-bis((E)-3,4-dihydroxybenzylidene)-4-(prop-2-yn-1-yloxy)cyclohexan-1-one (7**b**)*

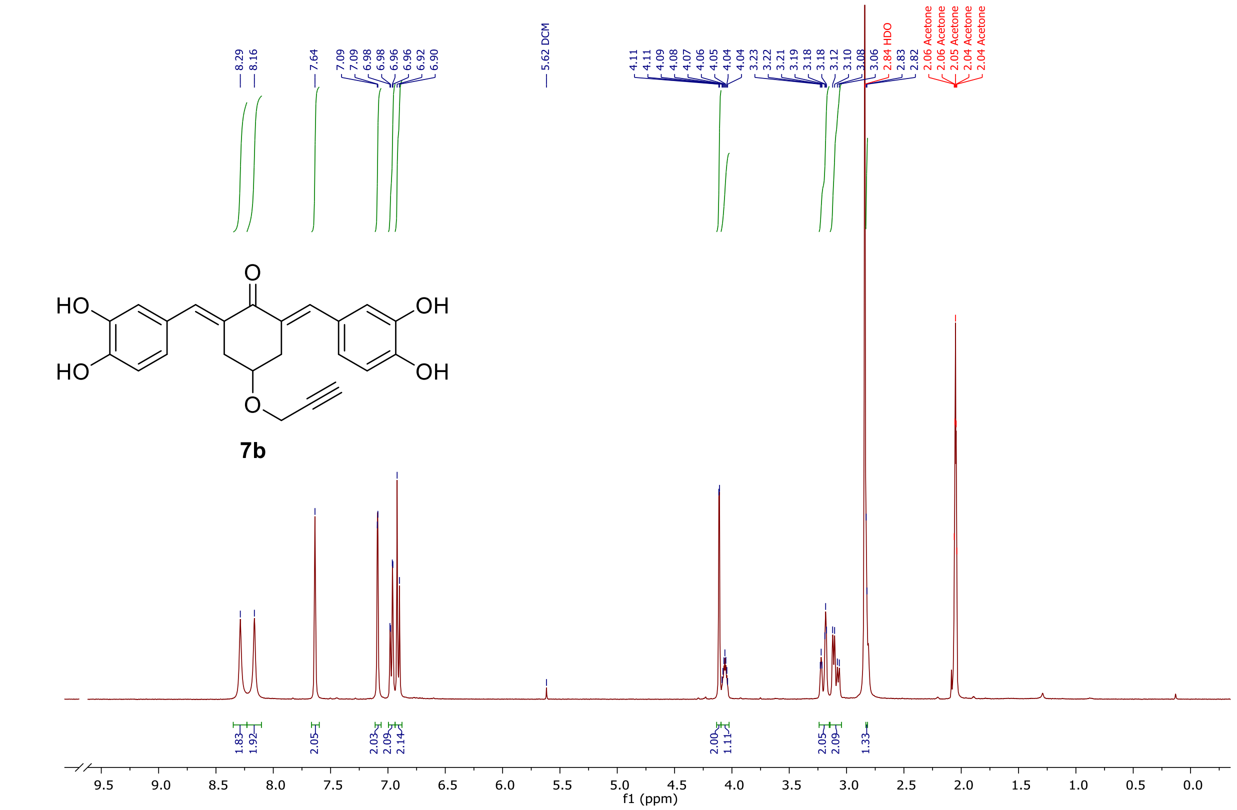

Figure S80. ^{1}H NMR 2,6-bis((*E*)-3,4-dihydroxybenzylidene)-4-(prop-2-yn-1-yloxy)cyclohexan-1-one (**7b**)

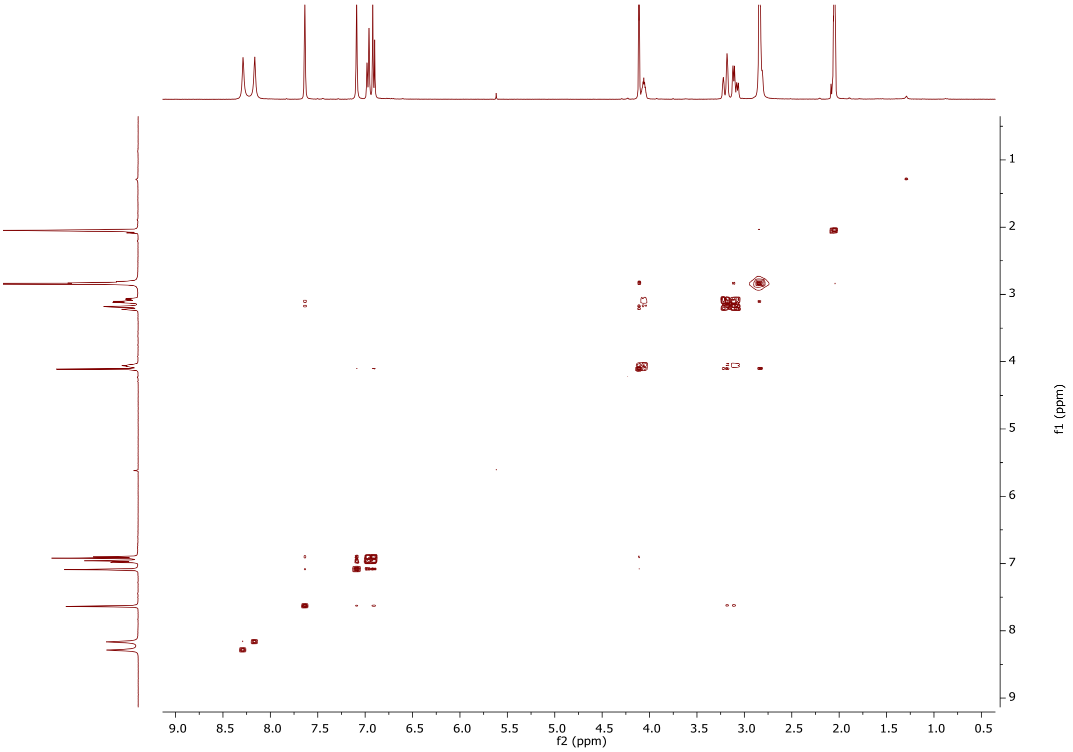

Figure S81. COSY 2,6-bis((*E*)-3,4-dihydroxybenzylidene)-4-(prop-2-yn-1-yloxy)cyclohexan-1-one (**7b**)

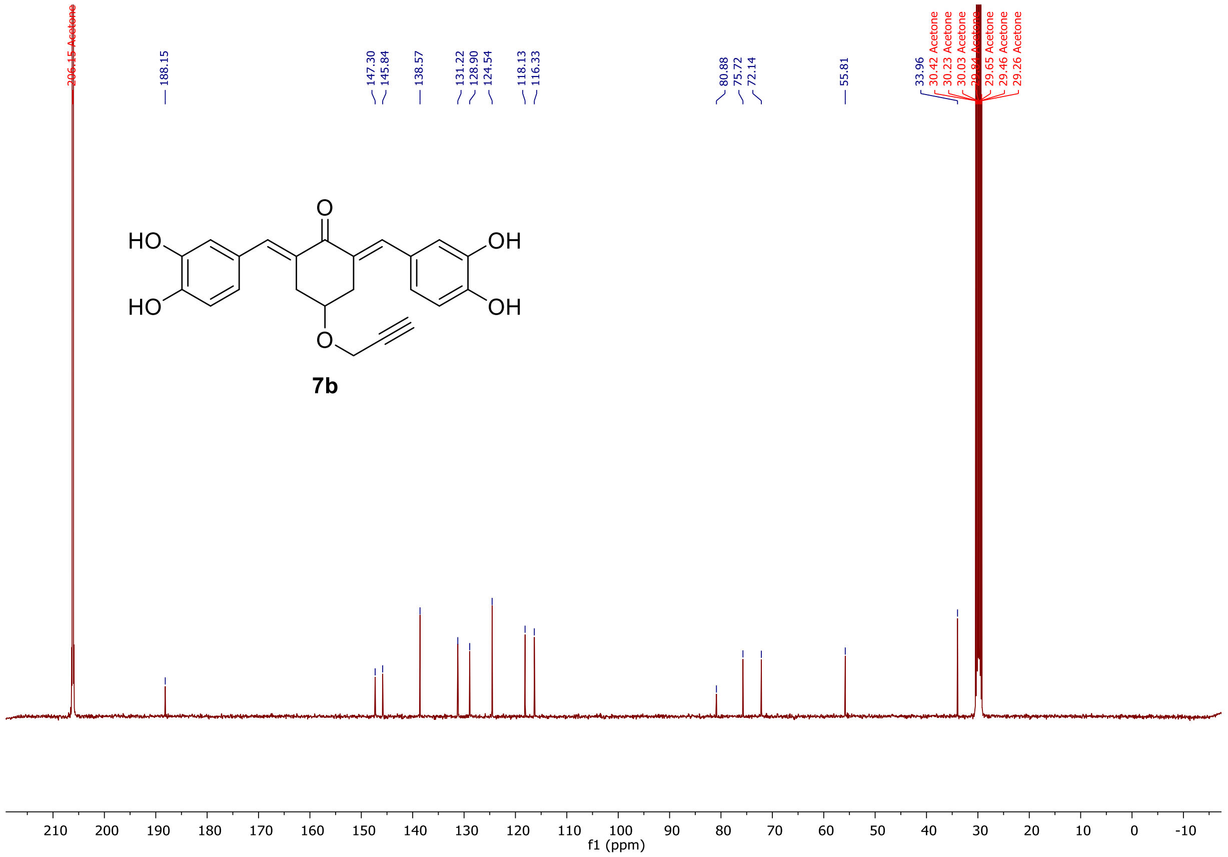

Figure S82. ^{13}C NMR 2,6-bis((*E*)-3,4-dihydroxybenzylidene)-4-(prop-2-yn-1-yloxy)cyclohexan-1-one (**7b**)

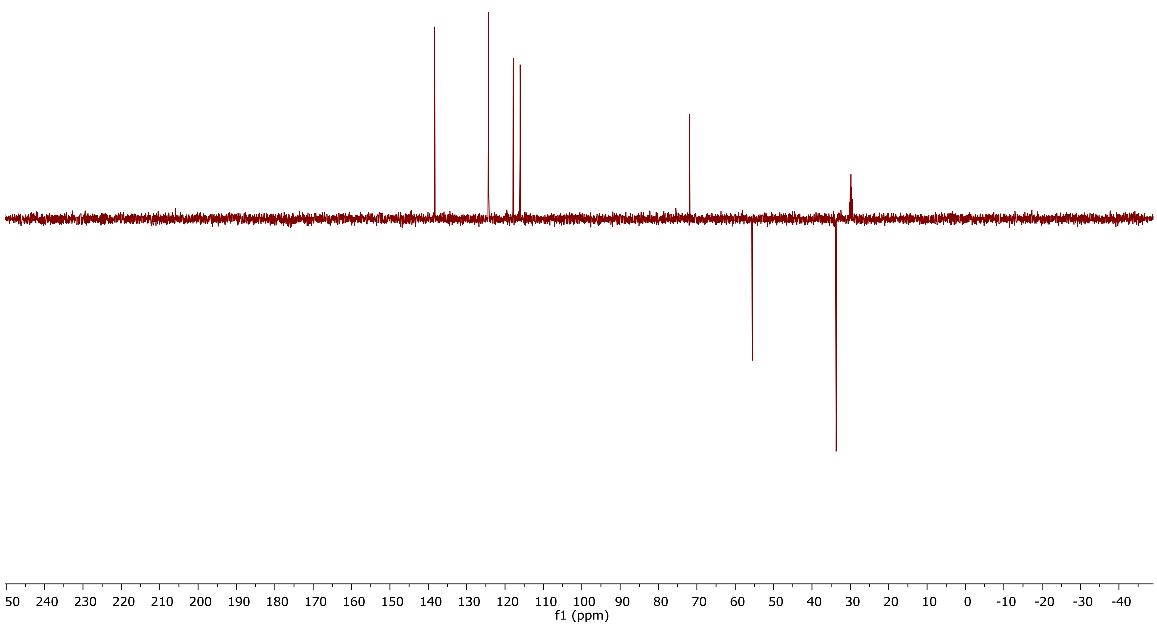

Figure S83. DEPT 135 2,6-bis((*E*)-3,4-dihydroxybenzylidene)-4-(prop-2-yn-1-yloxy)cyclohexan-1-one (**7b**)

UHPLC-MS

2,6-bis((E)-3,4-bis((tetrahydroxy)benzylidene)-4-((1-(2-ethylcarboranyl)-1H-1,2,3-triazol-4-yl)methoxy)cyclohexan-1-one (**9**)

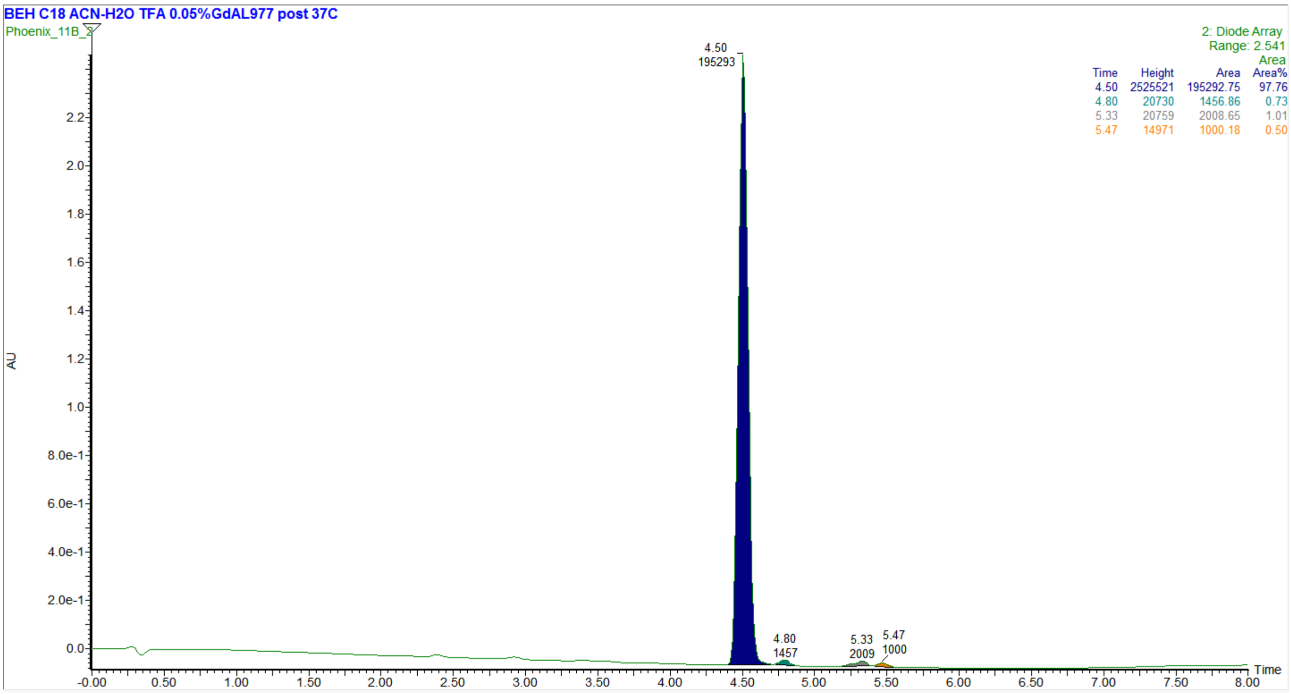

Figure S84. UHPLC chromatogram of 2,6-bis((E)-3,4-bis((tetrahydroxy)benzylidene)-4-((1-(2-ethylcarboranyl)-1H-1,2,3-triazol-4-yl)methoxy)cyclohexan-1-one (**9**)

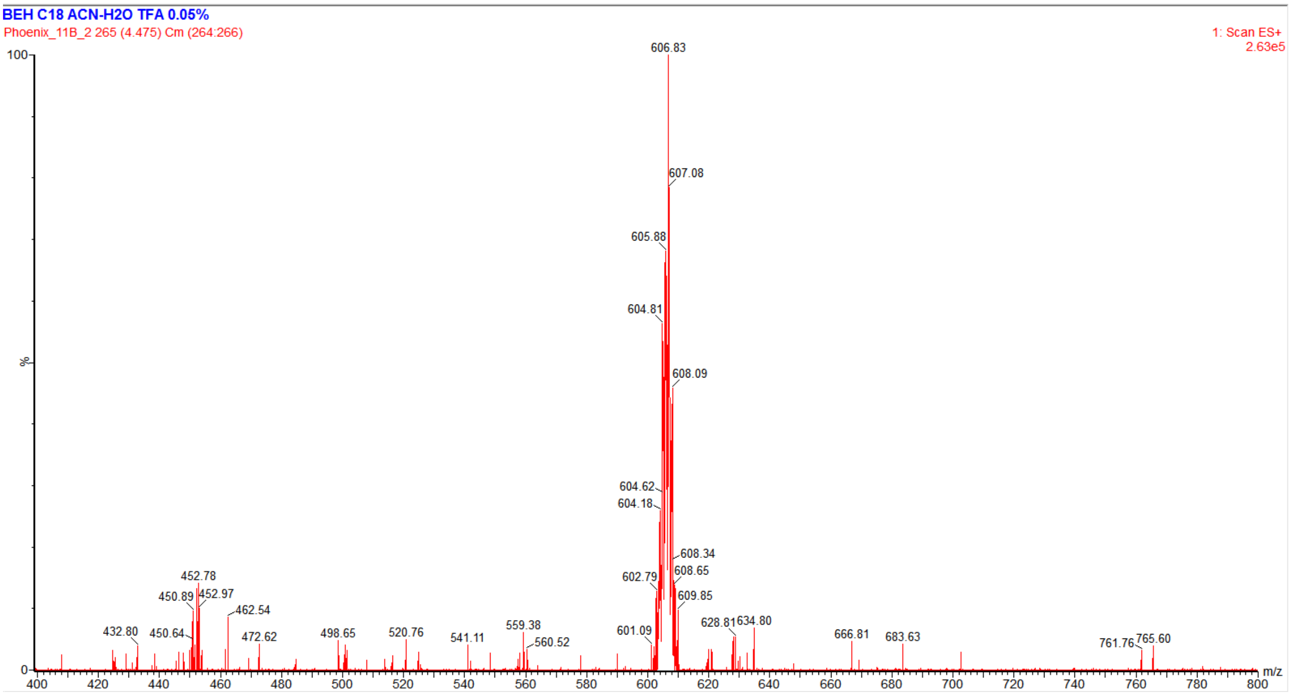

Figure S85. MS spectrum of 2,6-bis((E)-3,4-bis((tetrahydroxy)benzylidene)-4-((1-(2-ethylcarboranyl)-1H-1,2,3-triazol-4-yl)methoxy)cyclohexan-1-one (**9**)

*[10]B-enriched 2,6-bis((E)-3,4-bis((tetrahydroxy)benzylidene)-4-((1-(2-ethylcarboranyl)-1H-1,2,3-triazol-4-yl)methoxy)cyclohexan-1-one (**[10]B-9**)*

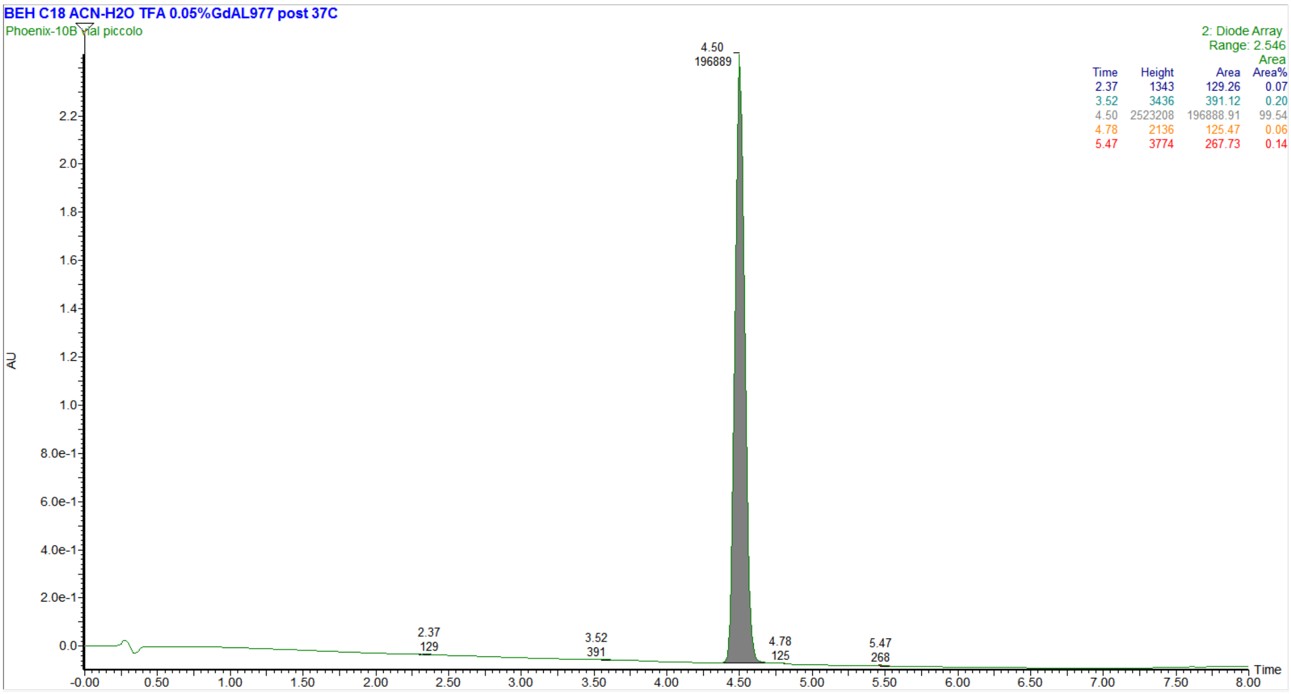

Figure S86. UHPLC chromatogram of [10]B-enriched 2,6-bis((E)-3,4-bis((tetrahydroxy)benzylidene)-4-((1-(2-ethylcarboranyl)-1H-1,2,3-triazol-4-yl)methoxy)cyclohexan-1-one (**[10]B-9**)

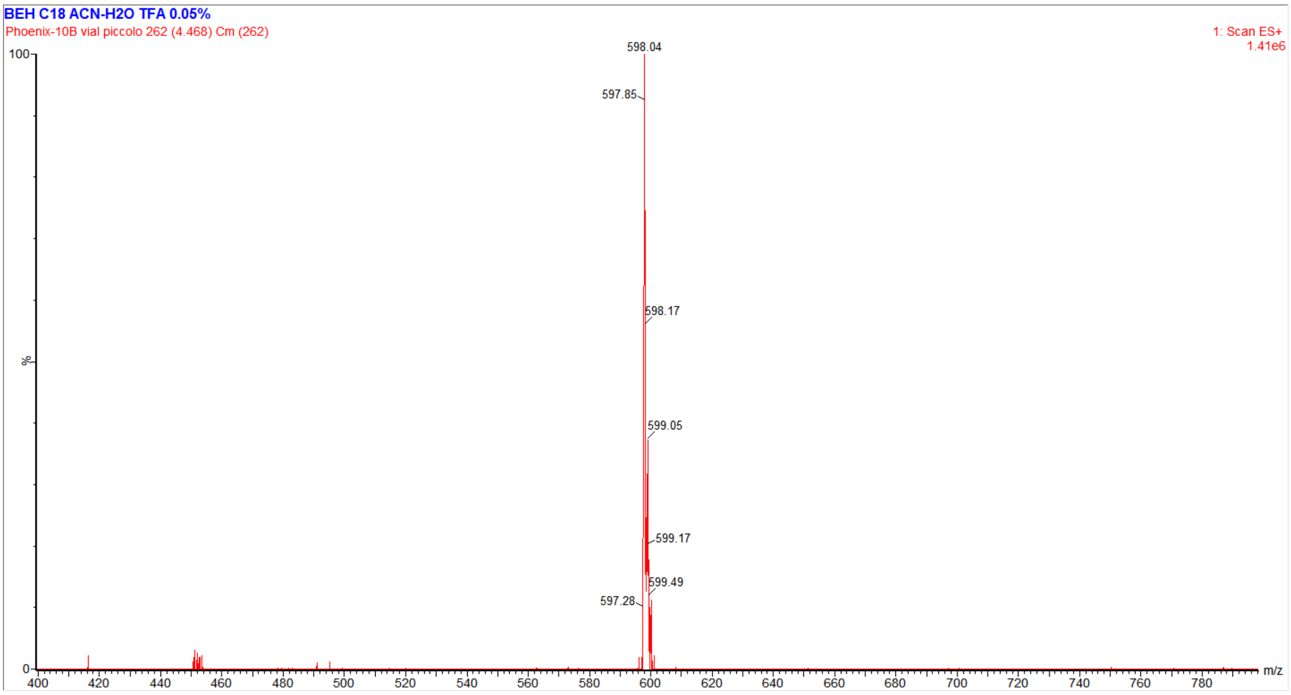

Figure S87. MS spectrum of ^{10}B-enriched 2,6-bis((E)-3,4-bis((tetrahydroxy)benzylidene)-4-((1-(2-ethylcarboranyl)-1H-1,2,3-triazol-4-yl)methoxy)cyclohexan-1-one (**^{10}B-9**)

*2,6-bis((E)-3-methoxy-4-(tetrahydroxy)benzylidene)-4-((1-(2-ethylcarboranyl)-1H-1,2,3-triazol-4-yl)methoxy)cyclohexan-1-on (**9a**)*

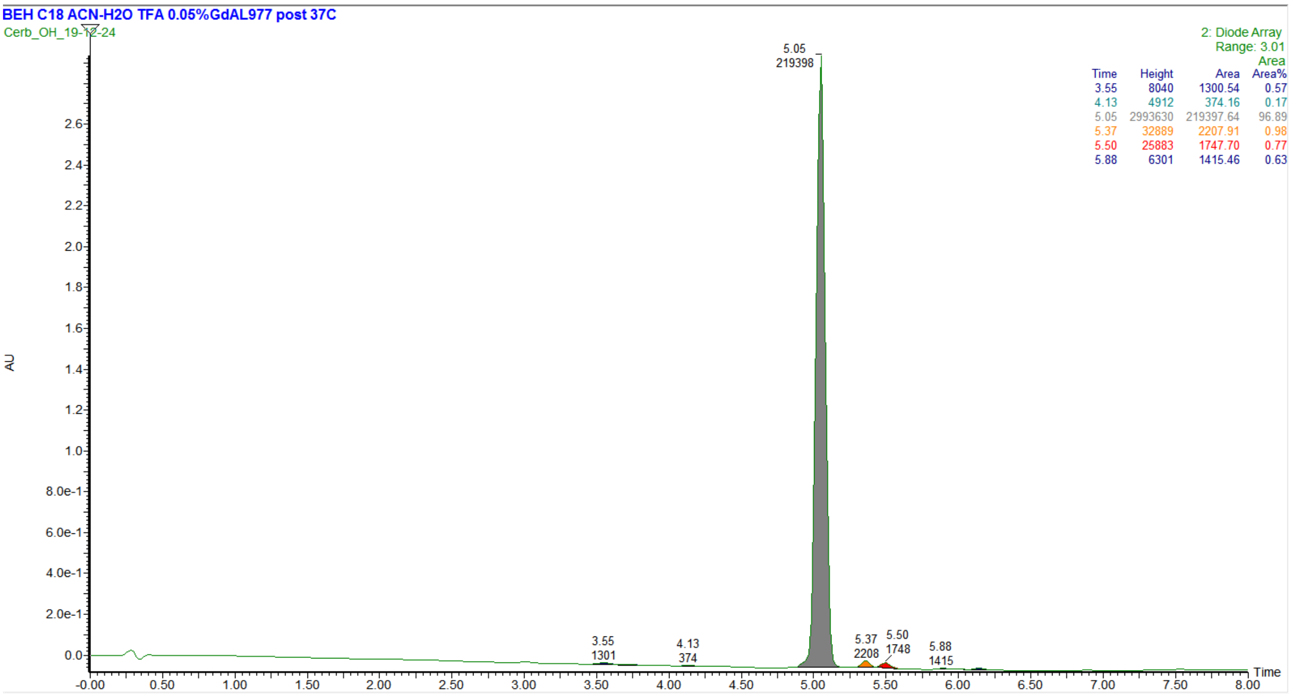

Figure S88. UHPLC chromatogram of 2,6-bis((E)-3-methoxy-4-(tetrahydroxy)benzylidene)-4-((1-(2-ethylcarboranyl)-1H-1,2,3-triazol-4-yl)methoxy)cyclohexan-1-on (**9a**)

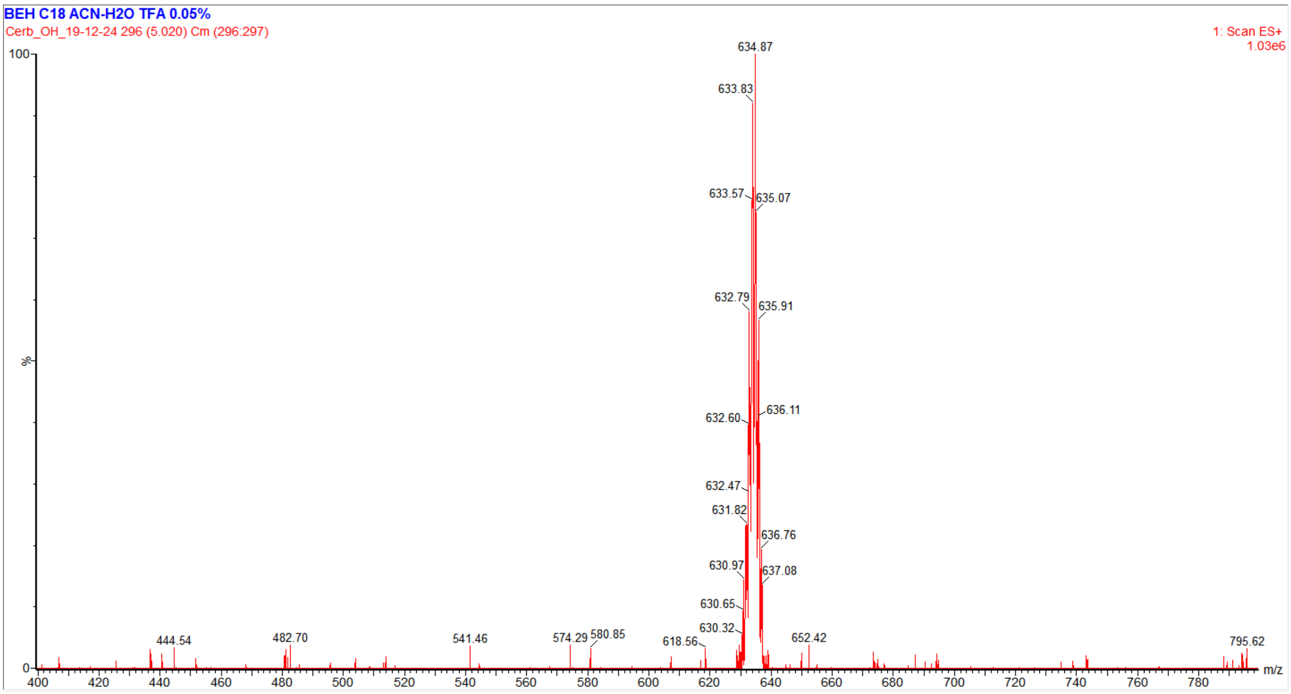

Figure S89. MS spectrum of 2,6-bis((E)-3-methoxy-4-(tetrahydroxy)benzylidene)-4-((1-(2-ethylcarboranyl)-1H-1,2,3-triazol-4-yl)methoxy)cyclohexan-1-on (**9a**)

*2,6-bis((E)-3,4-dihydroxybenzylidene)-4-(prop-2-yn-1-yloxy)cyclohexan-1-one (7**b**)*

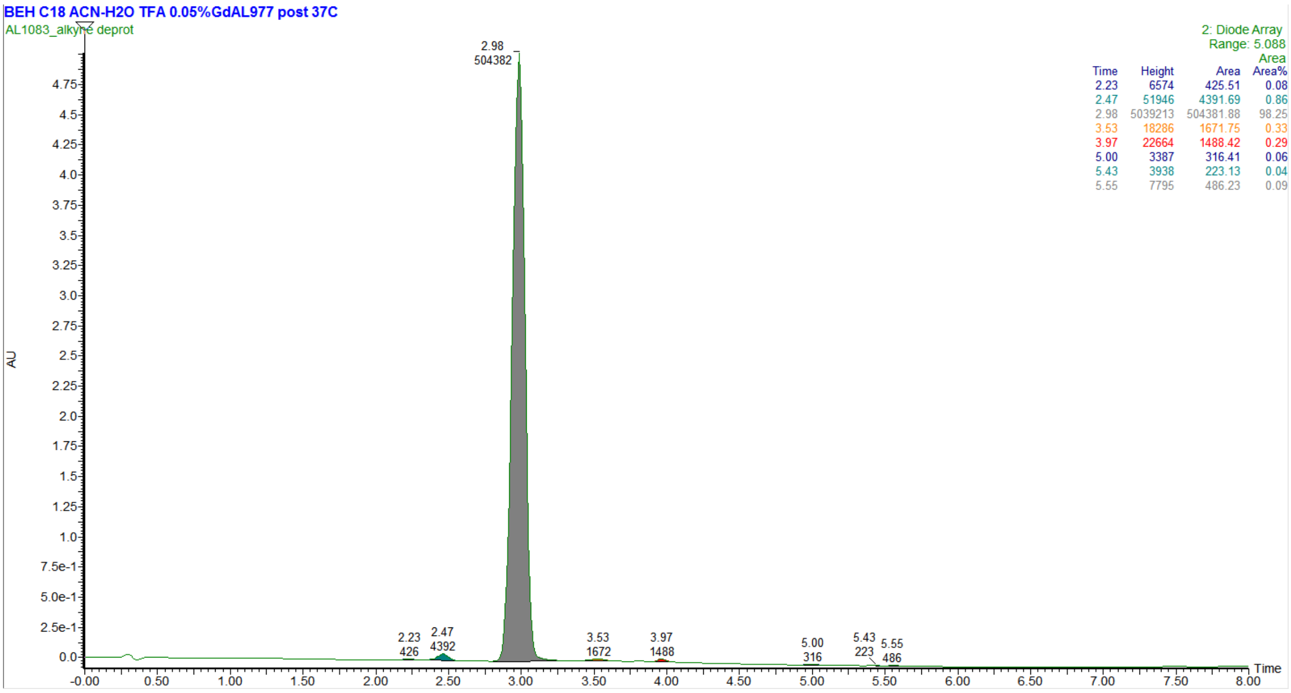

Figure S90. UHPLC chromatogram of 2,6-bis((*E*)-3,4-dihydroxybenzylidene)-4-(prop-2-yn-1-yloxy)cyclohexan-1-one (**7b**)

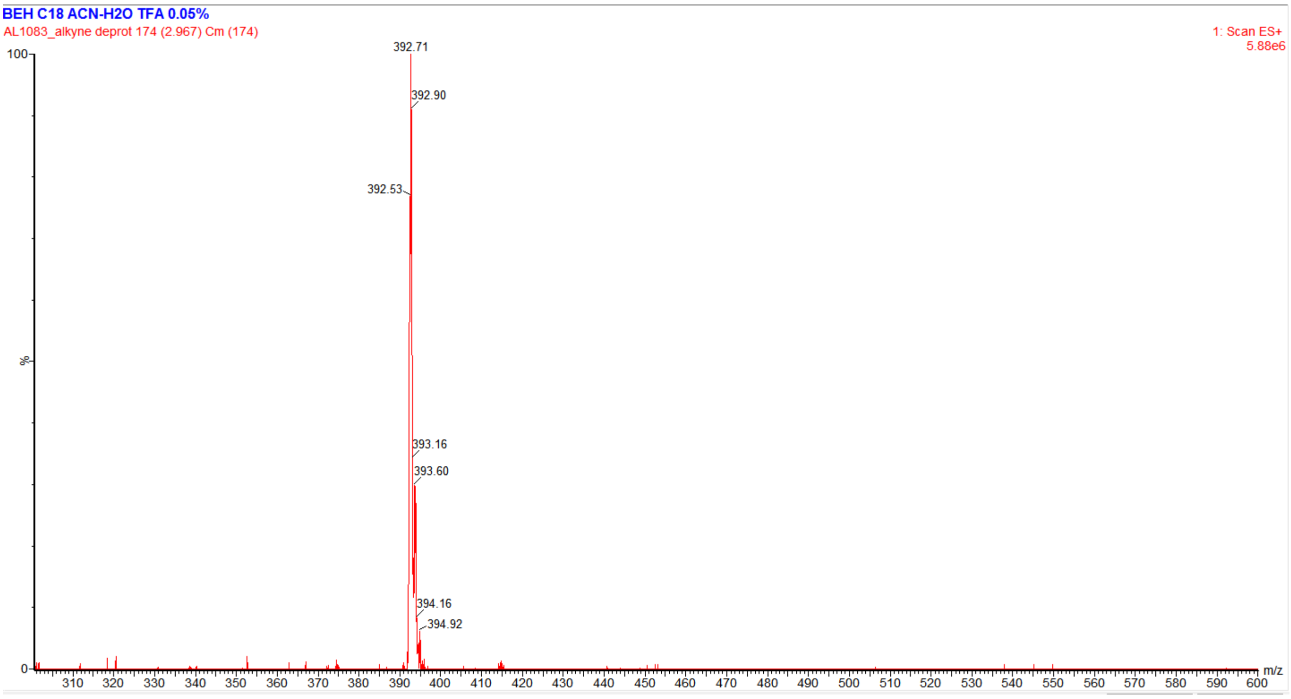

Figure S91. MS spectrum of 2,6-bis((*E*)-3,4-dihydroxybenzylidene)-4-(prop-2-yn-1-yloxy)cyclohexan-1-one (**7b**)
